# Brominated Depsidones with Antibacterial Effects from a Deep-Sea-Derived Fungus *Spiromastix* sp.

**DOI:** 10.3390/md22020078

**Published:** 2024-02-03

**Authors:** Zequan Huang, Dong Liu, Shang Chen, Jinwei Ren, Chenghai Gao, Zhiyong Li, Aili Fan, Wenhan Lin

**Affiliations:** 1 State Key Laboratory of Natural and Biomimetic Drugs, School of Pharmaceutical Sciences, Peking University, Beijing 100191, China; hzq092914@163.com (Z.H.); liudong_1982@126.com (D.L.); chenshang@bjmu.edu.cn (S.C.); 2 Institute of Marine Drugs, Guangxi University of Chinese Medicine, Nanning 530200, China; gaoch@gxtcmu.edu.cn; 3 State Key Laboratory of Mycology, Institute of Microbiology, Chinese Academy of Sciences, Beijing 100101, China; renjw@im.ac.cn; 4 State Key Laboratory of Microbial Metabolism, School of Life Sciences and Biotechnology, Shanghai Jiao Tong University, Shanghai 200240, China; zyli@sjtu.edu.cn; 5 Ningbo Institute of Marine Medicine, Peking University, Ningbo 315832, China

**Keywords:** fungus, *Spiromastix* sp., brominated depsidone, antibacterial effects

## Abstract

Eleven new brominated depsidones, namely spiromastixones U-Z5 (**1**–**11**) along with five known analogues (**12**–**16**), were isolated from a deep-sea-derived fungus *Spiromastix* sp. through the addition of sodium bromide during fermentation. Their structures were elucidated by extensive analysis of the spectroscopic data including high-resolution MS and 1D and 2D NMR data. Compounds **6**–**10** and **16** exhibited significant inhibition against Gram-positive bacteria including methicillin-resistant *Staphylococcus aureus* (MRSA) and vancomycin-resistant *Enterococcus faecium* (VRE) with MIC values ranging from 0.5 to 2.0 μM. Particularly, tribrominated **7** displayed the strongest activity against MRSA and VRE with a MIC of 0.5 and 1.0 μM, respectively, suggesting its potential for further development as a new antibacterial agent.

## 1. Introduction

Halogenated natural products are an important source for drug discovery and development [1,2]. Around 27% of small-molecule drugs and more than 80% of agrochemicals bear halogen atoms [3,4]. Well-known halogenated medicines include fludarabine [5], rebeccamycin [6], and vancomycin [2], which are clinically used for their antitumor and antibacterial effects. Halogenation can facilitate target binding, control metabolism, and improve pharmacological properties such as lipophilicity and permeability, leading natural products to exhibit improved bioactivities [7]. Vancomycin exhibits a remarkable drop in antibacterial bioactivity when removing one or both of the chlorine atoms, proving the significance of halogenation [2]. Due to the high concentrations of chloride and bromine ions in seawater, marine natural products become the main source of halogenated compounds [8]. Since chlorine concentrations are nearly three orders of magnitude higher than bromide concentrations in seawater, the chlorinated compounds are about nine times the frequency of brominated compounds found in the ocean [8]. Halogen types could affect the bioactivities of halogenated compounds [9]. For example, by varying the amounts of chloride and bromide salts in fermentation media, vancomycin-type glycopeptides balhimycins with bromine substitution were isolated and bromobalhimycins greatly altered the antimicrobial potency and profile [10].

**Figure 1:**
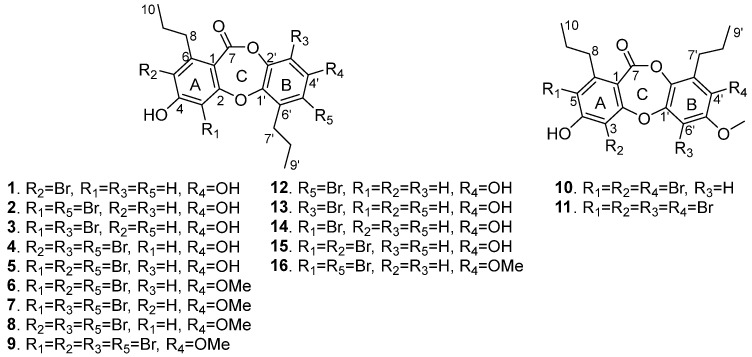
Structures of the isolated compounds **1**–**16**.

Fungi represent one of the most diverse groups of microorganisms on Earth, displaying a wide range of physiological plasticity that allows numerous taxa to thrive across both freshwater and marine ecosystems [11]. Deep-sea organisms survive in a multitude of extreme environmental conditions, such as the absence of sunlight, severely depleted oxygen concentrations, and crushing pressures. These stress factors may force marine-derived fungi to evolve to new mutants. Despite the fact that a substantial number of diverse natural products with distinct structures have been discovered from deep-sea flora and fauna, the exploration of secondary metabolites sourced from fungi dwelling beneath 1000 m in the oceanic abyss remains relatively uncharted territory [12]. During our search for bioactive secondary metabolites from the deep-sea-derived fungus *Spiromastix* sp. MCCC 3A00308, a series of chlorinated compounds were characterized, including the antibacterial depsidones spiromastixones A–S [12,13] and polyphenols spiromastols A–K [14], as well as the antiviral phenolic lactones spiromastilactones A–M [15]. Among the antibacterial chlorinated analogues, spiromastixone J exhibits significant antibacterial activity against methicillin-resistant *Staphylococcus aureus* (MRSA) and vancomycin-resistant *Enterococcus faecium* (VRE), comparable to the positive control penicillin. Structure—activity relationships (SARs) showed that the antimicrobial activity of the tetrachlorinated spiromastixone J is much stronger than its precursors [12]. To investigate the effect of bromination on the antibacterial activities of spiromastixones, we fermented the fungus *Spiromastix* sp. MCCC 3A00308 using rice media supplemented with sodium bromide. As a result, eleven new bromated depsidones, spiromastixones U-Z5 (**1**–**11**), together with spiromastixones P-T (**12**–**16**) (Figure 1) [12,16], were isolated and identified. Herein, we report the isolation, structure elucidation, and antibacterial activity of these brominated depsidones.

## 2. Results and Discussion

The EtOAc extract of the fermentation broth of *Spiromastix* sp. was separated by column chromatography followed by semipreparative HPLC purification to give compounds **1**–**16**.

**Figure 2:**
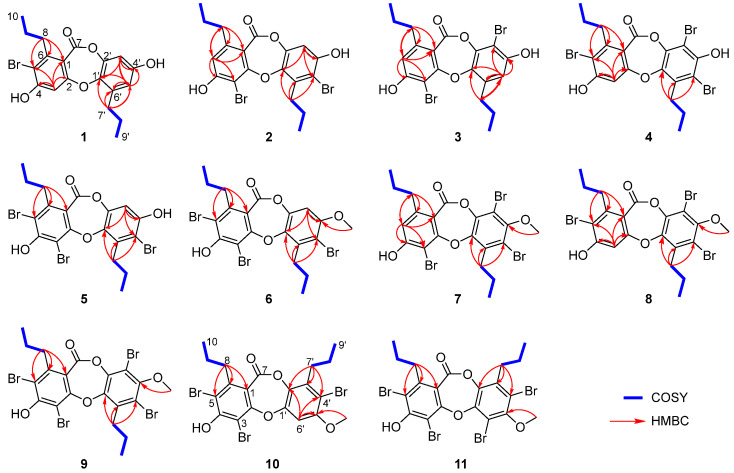
^1^H-^1^H COSY and HMBC correlations of **1**–**11**.

Spiromastixone U (**1**) was obtained as a white powder, and its molecular formula was established as C_19_H_19_BrO_5_ on the basis of the HRESIMS and NMR data. The ESI-MS spectrum displayed the molecular ions [M − H]^−^ at *m*/*z* 405 and 407 with a ratio of 1:1, indicating the presence of one bromine atom. In the ^1^H NMR spectrum, three aromatic protons were observed at *δ*_H_ 6.80 (1H, s, H-3), 6.50 (1H, d, *J* = 2.7 Hz, H-3′), and 6.47 (1H, d, *J* = 2.7 Hz, H-5′) (Table 1). The APT and HSQC data exhibited twelve aromatic carbons for two phenyl units, along with two methyls, four methylenes, and a carbonyl carbon (Figures S2 and S3). The NMR data featured a depsidone-type derivative, structurally related to spiromastixone B [12] from the same fungal strain. The ^1^H-^1^H COSY and HMBC correlations (Figure 2) led to the assignment of two n-propyl units, which directly connected to C-6 (*δ*_C_ 146.9) and C-6′ (*δ*_C_ 135.8) according to the HMBC correlations from H_2_-8 (*δ*_H_ 2.81) to C-1 (*δ*_C_ 111.1), C-5 (*δ*_C_ 113.6), and C-6, as well as from H_2_-7′ (*δ*_H_ 2.65) to C-1′ (*δ*_C_ 141.3), C-5′ (*δ*_C_ 113.2), and C-6′ (Figures S4 and S5). Furthermore, the HMBC correlations (Figure 2) from H-3 to C-1, C-2 (*δ*_C_ 161.7), C-4 (*δ*_C_ 159.7), C-5, and a carbonyl carbon at *δ*_C_ 162.9, H-3′ to C-1′ and C-5′, and H-5′ to C-1′, C-3′ (*δ*_C_ 105.5), C-4′ (*δ*_C_ 155.2), and C-7′ (*δ*_C_ 31.4) indicated **1** as a homologue of spiromastixone B [12]. The similar NMR data with the exception of the chemical shift at *δ*_C_ 113.6 for C-5 of **1** instead of *δ*_C_ 119.2 of the latter supported the location of a Br atom at C-5 (Figure 2) [12].

Spiromastixones V and W (**2** and **3**) were determined to have the same molecular formula of C_19_H_18_Br_2_O_5_ on the basis of the HRESIMS data, which were characterized by the presence of an isotopic cluster of ions [M − H]^−^ at *m*/*z* 483, 485 and 487 with a ratio of 1:2:1, supporting two bromine atoms in the molecules. The NMR data of **2** resembled those of spiromastixone D except for the shielded C-3 (*δ*_C_ 98.9) and C-5′ (*δ*_C_ 108.8) compared to those (*δ*_C_ 108.9 for C-3, and *δ*_C_ 117.3 for C-5′) of the latter suggested **2** to be a 3,5′-brominated analogue of spiromastixone D [12]. Spiromastixone W (**3**) was identified as an homologue of **2** with 3′-bromination rather than 5′-bromination due to the HMBC correlations from H-5′ (*δ*_H_ 6.69) to C-1′ (*δ*_C_ 142.7), C-3′ (*δ*_C_ 99.5), and from H_2_-7′ (*δ*_H_ 2.90) to C-1′, C-5′ (*δ*_C_ 113.3), and C-6′ (*δ*_C_ 134.8) to position the methylene carbon of an n-propyl unit at C-6′.

Spiromastixones X and Y (**4** and **5**) had the same molecular formula of C_19_H_16_Br_3_O_5_ with three bromine atoms as determined by the molecular ions [M − H]^−^ at *m*/*z* 560.8550 and 560.8542, respectively. The comparable NMR data suggested that both **4** and **5** were structurally related to **1–3**. Diagnostic 2D NMR data identified the partial structure regarding ring A of **4** to be the same as that of **1**, while C-3′ and C-5′ of ring B were brominated. Analogue **5** was determined as 3, 5, 5′-tribrominated spiromastixone by the comparison of its NMR data with those of **4** and the HMBC correlations from H-3′ (*δ*_H_ 6.80) to C-1′ (*δ*_C_ 142.0), C-2′ (*δ*_C_ 143.4), C-4′ (*δ*_C_ 153.0), and C-5′ (*δ*_C_ 108.9) (Figure 2).

Spriomastixone Z (**6**) was identified as a 4′-methoxylated analogue of **5** based on the comparable NMR data, except for the presence of a methoxy group whose methyl protons (*δ*_H_ 3.84, s) showed an HMBC correlation to C-4′ (*δ*_C_ 154.4). Spriomastixone Z2 (**8**) was determined as a 4′-methoxylated analogue of **4** due to the similar NMR data of both analogues but with the presence of a methoxy group in **8** and the HMBC correlation between the methoxy protons (*δ*_H_ 3.76) and C-4′ (*δ*_C_ 152.5).

Spriomastixone Z1 (**7**) had the same molecular composition as that of **8**, and the NMR data of both **7** and **8** were similar. The distinction was found in ring A, where an aromatic proton H-5 (*δ*_H_ 6.84, s) of **7** showed HMBC correlations to C-3 (*δ*_C_ 99.2), C-4 (*δ*_C_ 160.0), C-1 (*δ*_C_ 111.5), and C-8 (*δ*_C_ 35.7), implying C-3 to be brominated (Figure 2 and Figure S53).

Spiromastixone Z3 (**9**) was determined as a 3′-brominated analogue of **8** on the basis of the HRESIMS and NMR data (Figures S65–S70).

The molecular formula of spiromastixone Z4 (**10**) was the same as that of **6** as determined by the HRESIMS data. Analysis of the COSY and HMBC correlations established the partial structure regarding ring A to be identical to that of **6**. However, the unshielded C-1′ (*δ*_C_ 150.1) and a shielded C-2′ (*δ*_C_ 136.5) were observed in comparison with the corresponding resonances of **6** (Table 2). These findings indicated an alternate fusion of ring B through the ether and ester bonds [12].

The HRESIMS and NMR data indicated the molecular formula of spiromastixone Z5 (**11**) was the same as that of **9**. The alternate fusion of ring B was also indicated by an unshielded C-1′ (*δ*_C_ 149.1) and a shielded C-2′ (*δ*_C_ 141.9) compared to those of **9** (Table 2). These findings assigned an ester bond across C-7/C-2′ instead of C-7/C-1′ [12].

In addition, five known brominated depsidones were identical to spiromastixones P (**12**), Q (**13**), R (**14**), S (**15**), and T (**16**) based on the spectroscopic data in comparison with those reported in the literature (Tables S12 and S13) [16].

The biosynthetic pathways of depsidones have been extensively investigated [17,18,19], and a halogenase is identified in vitro to catalyze the triple halogenation [17]. The presence of tetrabrominated spiromastixones in this study suggests that the halogenase is able to process multiple steps for the halogenation utilizing NaBr as a halide source in addition to NaCl during biosynthesis.

Antibacterial bioassays demonstrated that most brominated spiromastixones exhibited significant inhibition toward the Gram-positive bacteria MRSA T144, MRSA 1530 (*cfr*), VRE CAU360 (*vanA*), and VRE CAU378 (*vanA*) with MIC values ranging from 0.5 to 8.0 μM (Table 3). However, none of the tested spiromastixones had any significant growth inhibition against the Gram-negative bacterium *E. coli*, indicating the selective effects of these analogues against Gram-positive bacteria. Analogue **7** inhibited the growth of MRSA with similar MIC values to the positive control vancomycin and displayed potent inhibition against VRE with a 1.5- to 12-fold lower MIC than that of the clinical medicine linezolid.

A primary analysis of the structure—activity relationships (SAR) revealed that the inhibitory effects of the spiromastixones depend on the number of bromine atoms. As shown in Table 3, tribrominated analogues (**4**–**8**) exhibited potent inhibition against a panel of the drug-resistant bacteria, while the analogues with a dibrominated ring B showed more activity than those with a dibrominated ring A such as **7**/**8** vs. **6**. In addition, analogues bearing a 4′-MeO group significantly enhanced the inhibitory effects in comparison with those with a 4′-OH group (**8** vs. **4**), and the analogues with the location of a bromine atom at C-3 improved the activity compared with 5-brominated analogues (**7** vs. **8**). Dibrominated analogues also showed significant inhibition against bacteria but were attenuated in comparison with tribrominated analogues. Similar to the results of tribrominated depsidones, analogues with a 4′-methoxy group and dibrominated ring B exhibited more effects than those bearing a 4′-hydroxy group and dibrominated ring A (**16** vs. **2**, **3** vs. **15**). Apparently, mono-brominated depsidones exhibited weaker effects than di- or tribrominated analogues. Tetrabrominated depsidones or those with an alternative fusion of ring B seem unlikely to further improve the antibacterial activities (**9** vs. **7**/**8**, **10** vs. **6**). The similar antibacterial activities between **9** and spiromastixone J suggested that both bromine and chlorine substitutions are favored for the structure modification. Therefore, depsidones characterized by the substitution of a 4′-methoxy group and dibromination in ring B commonly enhanced inhibitory properties in comparison to their counterparts with other substitution patterns.

MRSA is one of the most common antibiotic-resistant bacterial pathogens, causing approximately 171,000 invasive infections each year in Europe alone, and the World Health Organization now considers MRSA to be an important threat to human health [20]. Meanwhile, VRE was estimated to cause 5400 deaths in 2017 in USA [21]. Antimicrobial-resistant infections lead to substantial healthcare costs [22]. The inhibitory effects of **7** against MRSA and VRE suggest its potential for further development as an agent to treat multidrug-resistant bacterial infections.

## 3. Materials and Methods

### 3.1. General Experimental Procedures

UV spectra were detected on an Alltech UVIS-200 detector. IR spectra were determined on a Thermo Nicolet Nexus Is50 FT-IR spectrometer. The NMR spectra were acquired on a 400, 500, or 600 MHz Bruker FTNMR spectrometer. Chemical shifts are referenced to the solvent peaks at *δ*_H_ 2.50 and *δ*_C_ 39.52 for DMSO-*d*_6_. Mass spectra were obtained from a Bruker APEX IV 70 eV FT-MS spectrometer. The chromatographic (CC) substrates included silica gel (100−200 and 200−300 mesh) and HF254 silica gel for thin-layer chromatography (TLC) (Qingdao Marine Chemistry Co., Ltd., Qingdao, China). High-performance liquid chromatography (HPLC) was performed with an Alltech 426 pump equipped with an Alltech UVIS-200 detector (210 nm) and using a semipreparative reversed-phase column (YMC-packed, C_18_, 5 μM, 10 × 250 mm). UPLC was performed on an Agilent UPLC series 1200 (Agilent Technologies, Santa Clara, CA, USA) equipped with an Agilent Eclipse XDB-C18 column (5 μm, 4.6 × 150 mm). LC-MS analysis was carried out on an Agilent HPLC 1260 series system equipped with a Bruker microTOF QIII mass spectrometer by using an Agilent Eclipse XDB C18 column (5 μm, 4.6 × 150 mm) or Waters UPLC-MS system. The chemicals used in this study were obtained from Beijing Tongguang Fine Chemicals Company of the highest available purity.

### 3.2. Fungal Material

Fungus *Spiromastix* sp. MCCC 3A00308 was isolated from a deep-sea sediment collected from the South Atlantic Ocean (GPS13.7501 W, 15.1668 S) at a depth of 2869 m in June 2011. It was identified as *Spiromastix* sp. by a gene sequence analysis of the ITSregion of the rDNA (GenBank accession number KJ010057) [12]. The voucher specimen was deposited in the Marine Culture Collection of China (Xiamen, China).

### 3.3. Fermentation and Extraction

The *Spiromastix* sp. fungus was cultured in PDB medium at 28 °C on a rotary shaker at 200 r·min^−1^ for 12 days. The scale-up fermentation was carried out in Erlenmeyer flasks (30 × 500 mL) containing 60 g of rice per flask. A total of 1.833 g NaBr was added to each flask, and the contents were autoclaved at 121 °C for 20 min. After cooling to room temperature, each flask was inoculated with 5.0 mL PDB culture and incubated at 28 °C for 50 days. The fermentation broth was extracted with EtOAc and then was concentrated under reduced pressure to give an extract (32.58 g).

### 3.4. Isolation and Purification

The EtOAc extract (32.6 g) was subjected to a silica gel column, eluting with a gradient of cyclohexane–acetone (from 10:1 to 1:1) to obtain 9 fractions (FA−FI). FC (466.2 mg) was separated by semipreparative HPLC with a mobile phase of MeCN−H_2_O (19:20) containing 0.01% trifluoroacetic acid (TFA) to yield **6** (22.5 mg), **9** (6.3 mg), and **11** (3.3 mg). FD (1.558 g) was chromatographed on an ODS column eluting with MeOH−H_2_O (from 3:5 to MeOH) to afford 16 fractions (FD1−FD16). FD14 (576.8 mg) was separated by semipreparative HPLC with a mobile phase of MeCN−H_2_O (17:20) containing 0.01% TFA to yield **16** (15.8 mg). FD16 (426.5 mg) was subjected to Sephadex LH-20 using isocratic elution with a mixture of CH_2_Cl_2_: MeOH (1:1) to remove fatty acids. Then, the residue (113.3 mg) was separated by semipreparative HPLC with a mobile phase of MeCN−H_2_O (83:100) containing 0.01% TFA to yield **7** (18.1 mg), **8** (3.8 mg), and **10** (5.3 mg). FE (4.0 g) was chromatographed on an ODS column eluting with MeOH−H_2_O (from 11:20 to MeOH) to afford 7 fractions (FE1−FE7). FE3 (398.2 mg) and FE6 (840.1 mg) were separated by semipreparative HPLC with a mobile phase of MeCN−H_2_O (11:20) containing 0.01% TFA to yield **1** (4.7 mg), **4** (3.4 mg), **5** (4.8 mg), **12** (10.2 mg), and **14** (8.1 mg). FE5 (1.3 g) extraction followed the same protocol as for FE3 to yield **2** (6.0 mg) and **15** (8.1 mg). FF (1.4 g) was fractionated on an ODS column eluting with MeOH−H_2_O (11:20) to collect 11 portions (FF1−FF11). FF6 (193.5 mg) and FF8 (38.7 mg) were separated by semipreparative HPLC eluting with MeCN−H_2_O (11:20) containing 0.01% TFA to give **3** (22.8 mg) and **13** (6.7 mg).

Spiromastixone U (**1**): white powder; UV (MeOH) λ_max_ 264 nm; IR (KBr) *v*_max_ 3193, 2963, 2873, 1732, 1688, 1597, 1565, 1465, 1345, 1239 cm^−1^; ^1^H and ^13^C NMR data, see Table 1 and Table 2. HRESIMS *m*/*z* 405.0342 [M − H]^−^ (calcd for C_19_H_19_^79^BrO_5_, 405.0338).

Spiromastixone V (**2**): red powder; UV (MeOH) λ_max_ 203, 273 nm; IR (KBr) *v*_max_ 3257, 2958, 1727, 1684, 1587, 1489, 1377, 1299 cm^−1^; ^1^H and ^13^C NMR data, see Table 1 and Table 2. HRESIMS *m*/*z* 482.9462 [M − H]^−^ (calcd for C_19_H_17_^79^Br_2_O_5_, 482.9443).

Spiromastixone W (**3**): white powder; UV (MeOH) λ_max_ 205, 272 nm; IR (KBr) *v*_max_ 3191, 2963, 1736, 1590, 1490, 1250 cm^−1^; ^1^H and ^13^C NMR data, see Table 2 and Table 3. HRESIMS *m*/*z* 482.9443 [M − H]^−^ (calcd for C_19_H_17_^79^Br_2_O_5_, 482.9443).

Spiromastixone X (**4**): white powder; UV (MeOH) λ_max_ 210 nm; IR (KBr) *v*_max_ 3184, 2962, 1742, 1597, 1565, 1419, 1330, 1234 cm^−1^; ^1^H and ^13^C NMR data, see Table 1 and Table 2. HRESIMS *m*/*z* 560.8550 [M − H]^−^ (calcd for C_19_H_16_^79^Br_3_O_5_, 560.8548).

Spiromastixone Y (**5**): red powder; UV (MeOH) λ_max_ 224 nm; IR (KBr) *v*_max_ 3181, 2961, 2930, 1731, 1685, 1584, 1563, 1428, 1241 cm^−1^; ^1^H and ^13^C NMR data, see Table 1 and Table 2. HRESIMS *m*/*z* 560.8542 [M − H]^−^ (calcd for C_19_H_16_^79^Br_3_O_5_, 560.8548).

Spiromastixone Z (**6**): white powder; UV (MeOH) λ_max_ 205, 224 nm; IR (KBr) *v*_max_ 3326, 2961, 1723, 1586, 1572, 1539, 1464, 1439, 1422, 1285 cm^−1^; ^1^H and ^13^C NMR data, see Table 1 and Table 2. HRESIMS *m*/*z* 574.8701 [M − H]^−^ (calcd for C_20_H_18_^79^Br_3_O_5_, 574.8704).

Spiromastixone Z1 (**7**): red powder; UV (MeOH) λ_max_ 211, 271 nm; IR (KBr) *v*_max_ 3281, 2961, 1742, 1591, 1490, 1453, 1407, 1343, 1249 cm^−1^; ^1^H and ^13^C NMR data, see Table 1 and Table 2. HRESIMS *m*/*z* 574.8717 [M − H]^−^ (calcd for C_20_H_18_^79^Br_3_O_5_, 574.8704).

Spiromastixone Z2 (**8**): red powder; UV (MeOH) λ_max_ 210 nm; IR (KBr) *v*_max_ 3184, 2962, 1745, 1690, 1599, 1564, 1453, 1409, 1342 cm^−1^; ^1^H and ^13^C NMR data, see Table 1 and Table 2. HRESIMS *m*/*z* 574.8713 [M − H]^−^ (calcd for C_20_H_18_^79^Br_3_O_5_, 574.8704).

Spiromastixone Z3 (**9**): white powder; UV (MeOH) λ_max_ 212 nm; IR (KBr) *v*_max_ 3325, 2963, 1751, 1563, 1453, 1237 cm^−1^; ^1^H and ^13^C NMR data, see Table 1 and Table 2. HRESIMS *m*/*z* 652.7812 [M − H]^−^ (calcd for C_20_H_17_^79^Br_4_O_5_, 652.7809).

Spiromastixone Z4 (**10**): white powder; UV (MeOH) λ_max_ 204, 223 nm; IR (KBr) *v*_max_ 3350, 2962, 1689, 1569, 1462, 1438, 1345 cm^−1^; ^1^H and ^13^C NMR data, see Table 1 and Table 2. HRESIMS *m*/*z* 574.8712 [M − H]^−^ (calcd for C_20_H_18_^79^Br_3_O_5_, 574.8704).

Spiromastixone Z5 (**11**): white powder; UV (MeOH) (log ε) λ_max_ 211 nm; IR (KBr) *v*_max_ 3399, 2961, 1690, 1551, 1452, 1339 cm^−1^; ^1^H and ^13^C NMR data, see Table 1 and Table 2. HRESIMS *m*/*z* 652.7812 [M − H]^−^ (calcd for C_20_H_17_^79^Br_4_O_5_, 652.7809).

### 3.5. Antibacterial Assays

Antimicrobial activities were measured against six bacterial strains including MRSA T144, MRSA 1530, VRE CAU360, VRE CAU378, *E. coli* ATCC 25922, and *E. coli* B2 (mcr-1 + blaNDM-5) by the standard broth microdilution method according to the CLSI M100 guideline (https://clsi.org/standards/products/microbiology/documents/m100/, accessed on 10 December 2023) [22]. Vancomycin, linezolid, and colistin were used as positive controls for MRSA, VRE, and *E. coli*, respectively. The antibiotic stock solutions were dissolved also according to the CLSI M100 guideline. Mueller–Hinton broth (MHB) containing 6.4% DMSO was used as a negative control. Compounds were dissolved in DMSO with a final concentration of 10 mM as stock solutions. The MIC values were obtained by a gradient of concentrations beginning with 64 μM and then diluted 2-fold in MHB and mixed with equal volumes of bacterial suspensions in MHB containing approximately 1.5 × 10^6^ colony-forming units (CFUs)/mL in a clear UV-sterilized 96-well microtiter plate. After 16–20 h incubation at 37 °C, the MIC values were defined as the lowest concentrations of compounds with no visible growth of bacteria. Experiments were performed in two biological replicates and the MIC values are calculated by the data obtained from the two biological replicates. *E. coli* ATCC 25922 is utilized as the standard strain to indicate the quality of the MIC value. The MIC value of colistin against *E. coli* ATCC 25922 of this study is 0.25 μg/mL, consistent with the CLSI guideline, indicating that the experiments are trustworthy.

## 4. Conclusions

In summary, eleven new brominated depsidones (**1**–**11**) together with five known analogues were isolated from the fermentation broth of a deep-sea-derived fungus *Spiromastix* sp. These findings provide additional evidence that OSMAC by altering the cultural medium could be an efficacious method to produce novel bioactive halogenated compounds. Although numerous depsidones have been isolated from natural sources with diverse bioactivities, the present study together with our previous work suggests that halogenation plays a critical role for the antibacterial effects of depsidones. Similar to chlorinated spiromastixones, brominated depsidones also exhibit significant inhibition against drug-resistant Gram-positive bacteria. Among the antibacterial brominated depsidones, spiromastixone Z1 (**7**) displays the highest inhibitory activity against MRSA and VRE with MIC values of 0.5 and 1.0 μM, respectively. These findings highlight the promising lead of **7** as an agent in combating multidrug-resistant bacterial infections.

## Figures and Tables

**Table 1:** ^1^H NMR data of **1**–**11** in DMSO-*d*6 (*δ*_H_ ppm, *J* in Hz).

Position	1 ^a^	2 ^a^	3 ^a^	4 ^a^	5 ^a^	6 ^b^	7 ^a^	8 ^a^	9 ^b^	10 ^a^	11 ^c^
3	6.80, s			6.71, s				6.84, s			
5		6.79, s	6.80, s				6.84, s				
8	2.81, t (7.9)	2.66, t (7.8)	2.70, t (7.6)	2.86, t (7.7)	3.11, t (8.0)	2.78, t (7.9)	2.68, t (7.7)	2.85, m	2.81, t (7.7)	2.79, m	2.72, t (8.0)
9	1.54, m	1.49, m	1.49, m	1.54, m	1.59, m	1.47, m	1.48, m	1.55, m	1.58, m	1.53, m	1.56, m
10	0.89, t (7.3)	0.86, t (7.3)	0.83, t (7.2)	0.86, t (7.3)	0.88, t (7.3)	0.90, t (7.3)	0.83, t (7.3)	0.88, t (7.2)	0.87, t (7.3)	0.90, t (7.2)	0.87, t (7.2)
3′	6.50, d (2.7)	6.77, s			6.80, s	7.12, s					
5′	6.47, d (2.7)		6.69, s								
6′										7.07, s	
7′	2.65, t (7.8)	3.13, t (8.0)	2.90, t (7.8)	2.81, t (8.1)	2.76, t (7.8)	3.14, t (7.5)	3.16, t (8.0)	2.87, m	3.15, t (7.9)	2.81, m	2.83, t (7.5)
8′	1.57, m	1.48, m	1.07, m	1.51, m	1.48, m	1.61, m	1.50, m	1.55, m	1.50, m	1.50, m	1.54, m
9′	0.97, t (7.3)	1.00, t (7.3)	0.95, t (7.2)	0.98, t (7.3)	1.01, t (7.3)	1.01, t (7.3)	1.00, t (7.2)	1.04, t (7.2)	1.02, t (7.3)	0.90, t (7.2)	0.92, t (7.2)
OMe						3.84, s	3.78, s	3.76, s	3.78, s	3.85, s	3.78, s

^a^ 500 MHz; ^b^ 400 MHz; ^c^ 600 MHz.

**Table 2:** ^13^C NMR data of **1–11** in DMSO-*d*6 (*δ*_C_ ppm).

Position	1 ^a^	2 ^a^	3 ^a^	4 ^a^	5 ^a^	6 ^b^	7 ^a^	8 ^a^	9 ^b^	10 ^a^	11 ^c^
1	111.1, C	112.5, C	112.3, C	112.9, C	113.6, C	113.2, C	111.5, C	111.7, C	112.7, C	112.6, C	-
2	161.7, C	160.4, C	160.6, C	161.7, C	159.3, C	159.2, C	160.2, C	161.0, C	158.8, C	157.6, C	158.9, C
3	105.3, CH	98.9, C	99.0, C	105.2, CH	101.3, C	101.3, C	99.2, C	105.7, CH	101.5, C	102.5, C	102.2, C
4	159.7, C	159.6, C	159.7, C	161.7, C	159.3, C	159.2, C	160.0, C	161.0, C	158.8, C	157.6, C	158.9, C
5	113.6, C	115.1, CH	115.3, C	110.3, C	112.8, C	114.1, C	115.6, C	112.4, C	113.8, C	114.1, C	116.2, C
6	146.9, C	148.5, C	148.1, C	146.9, C	145.8, C	146.1, C	148.8, C	147.3, C	146.1, C	144.6, C	145.7, C
7	162.9, C	162.1, C	161.8, C	161.9, C	162.0, C	161.7, C	160.7, C	161.2, C	160.6, C	161.9, C	161.6, C
8	36.0, CH_2_	35.7, CH_2_	35.5, CH_2_	35.7, CH_2_	36.9, CH_2_	27.1, CH_2_	35.7, CH_2_	35.7, CH_2_	36.6, CH_2_	36.1, CH_2_	36.9, CH_2_
9	23.0, CH_2_	24.5, CH_2_	24.6, CH_2_	22.9, CH_2_	22.9, CH_2_	23.1, CH_2_	24.5, CH_2_	22.8, CH_2_	22.7, CH_2_	23.0, CH_2_	23.0, CH_2_
10	14.3, CH_3_	14.3, CH_3_	14.1, CH_3_	14.2, CH_3_	14.3, CH_3_	14.4, CH_3_	14.2, CH_3_	14.2, CH_3_	14.1, CH_3_	14.1, CH_3_	14.5, CH_3_
1′	141.3, C	142.0, C	142.7, C	142.0, C	142.0, C	143.0, C	146.7, C	146.1, C	146.6, C	150.1, C	149.1, C
2′	144.6, C	143.4, C	142.9, C	142.1, C	143.4, C	143.7, C	142.2, C	142.5, C	142.2, C	136.5, C	141.9, C
3′	105.5, CH	105.6, CH	99.5, C	102.2, C	105.6, C	103.5, CH	108.3, C	108.0, C	108.3, C	135.2, C	134.0, C
4′	155.2, C	152.9, C	153.0, C	151.0, C	153.0, C	154.4, C	152.8, C	152.5, C	152.9, C	110.8, C	117.5, C
5′	113.2, CH	108.8, C	113.3, CH	111.1, C	108.9, C	110.0, C	117.2, C	116.6, C	117.2, C	153.9, C	152.0, C
6′	135.8, C	136.0, C	134.8, C	134.0, C	135.9, C	136.1, C	135.5, C	135.2, C	135.5, C	103.6, C	108.3, C
7′	31.4, CH_2_	33.0, CH_2_	32.7, CH_2_	32.6, CH_2_	33.0, CH_2_	33.0, CH_2_	33.2, CH_2_	32.5, CH_2_	33.2, CH_2_	32.3, CH_2_	32.5, CH_2_
8′	23.7, CH_2_	23.1, CH_2_	24.2, CH_2_	22.5, CH_2_	23.1, CH_2_	22.9, CH_2_	22.8, CH_2_	22.5, CH_2_	22.9, CH_2_	22.1, CH_2_	22.0, CH_2_
9′	14.4, CH_3_	14.4, CH_3_	14.1, CH_3_	14.9, CH_3_	14.4, CH_3_	14.4, CH_3_	14.3, CH_3_	14.5, CH_3_	14.3, CH_3_	14.4, CH_3_	14.0, CH_3_
OMe						57.6, CH_3_	60.9, CH_3_	60.9, CH_3_	60.8, CH_3_	57.3, CH_3_	60.8, CH_3_

^a^ 125 MHz; ^b^ 100 MHz; ^c^ 150 MHz.

**Table 3:** Antibacterial activities of **1**–**16** and spiromastixone J (MIC in μM).

Compounds	MRSA T144	MRSA 1530	VRE CAU360	VRE CAU378	*E. coli* ATCC 25922	*E. coli* B2 (mcr-1 + blaNDM-5)
**1**	32	32	64	64	>64	>64
**2**	8	8	8	8	>64	>64
**3**	16	16	32	32	>64	>64
**4**	4	8	16	16	>64	>64
**5**	4	4	8	8	>64	>64
**6**	2	2	2	2	>64	>64
**7**	0.5	0.5	1	1	>64	>64
**8**	1	1	2	2	>64	>64
**9**	2	2	1	1	>64	>64
**10**	2	2	2	2	>64	>64
**11**	8	8	4	4	>64	>64
**12**	8	8	8	8	>64	>64
**13**	16	16	16	16	>64	>64
**14**	64	64	64	64	>64	>64
**15**	64	64	>64	>64	>64	>64
**16**	2	2	2	2	>64	>64
Spiromastixone J	2	2	2	2	>64	>64
Vancomycin *	0.35	0.35	/	/	/	/
Linezolid *	/	/	5.93	11.86	/	/
Colistin *	/	/	/	/	0.22	6.92

**/**: not determined; *: positive control.

## Data Availability

The data presented in this study are available on request from the corresponding author.

## Supplementary Materials

The following supporting information can be downloaded at: https://www.mdpi.com/article/10.3390/md22020078/s1, Figures S1–S128 and Tables S1–S13: Spectra of compounds **1**–**16**.
