# Supplementary material for: Brominated Depsidones with Antibacterial Effects from a Deep-Sea-Derived Fungus Spiromastix sp
Source: Mar Drugs. 2024 Feb 3;22(2):78. doi: 10.3390/md22020078 (PMC10890614; doi:10.3390/md22020078)
Supplement: Supplementary file 1 [file marinedrugs-22-00078-s001.zip › marinedrugs-2848042-supplementary.pdf]

Supplementary data

Brominated Depsidones with Antibacterial Effects from a Deep-Sea-Derived Fungus *Spiromastix* sp.

Zequan Huang[1,2,†], Dong Liu[1,†], Shang Chen[1], Jinwei Ren[3], Chenghai Gao[2], Zhiyong Li[4], Aili Fan[1,*] and Wenhan Lin[1,5,*]

[1] State Key Laboratory of Natural and Biomimetic Drugs, School of Pharmaceutical Sciences, Peking University, Beijing 100191, China; hzq092914@163.com (Z.H.); liudong_1982@126.com (D.L.); chenshang@bjmu.edu.cn (S.C.)
[2] Institute of Marine Drugs, Guangxi University of Chinese Medicine, Nanning 530200, China; gaoch@gxtcmu.edu.cn
[3] State Key Laboratory of Mycology, Institute of Microbiology, Chinese Academy of Sciences, Beijing 100101, China; renjw@im.ac.cn
[4] State Key Laboratory of Microbial Metabolism, School of Life Sciences and Biotechnology, Shanghai Jiao Tong University, Shanghai 200240, China; zyli@sjtu.edu.cn
[5] Ningbo Institute of Marine Medicine, Peking University, Ningbo 315832 Zhejiang, China
* Correspondence: fanaili@bjmu.edu.cn (A.F.); whlin@bjmu.edu.cn (W.L.)
† These authors contributed equally to this work.

Table of Contents

Table S1. ^{1}H and ^{13}C NMR data and key COSY and HMBC correlations of **1** 6
Figure S1. ^{1}H-NMR spectrum of **1** in DMSO-d_6 (500 MHz) 7
Figure S2. APT spectrum of **1** in DMSO-d_6 (125 MHz) 8
Figure S3. HSQC spectrum of **1** in DMSO-d_6 9
Figure S4. ^{1}H-^{1}H COSY spectrum of **1** in DMSO-d_6 10
Figure S5. HMBC spectrum of **1** in DMSO-d_6 11
Figure S6. HRESIMS spectrum of **1** 12
Figure S7. UV spectrum of **1** 13
Figure S8. IR spectrum of **1** 14
Table S2. ^{1}H and ^{13}C NMR data and key COSY and HMBC correlations of **2** 15
Figure S9. ^{1}H-NMR spectrum of **2** in DMSO-d_6 (500 MHz) 16
Figure S10. APT spectrum of **2** in DMSO-d_6 (125 MHz) 17
Figure S11. HSQC spectrum of **2** in DMSO-d_6 18
Figure S12. ^{1}H-^{1}H COSY spectrum of **2** in DMSO-d_6 19
Figure S13. HMBC spectrum of **2** in DMSO-d_6 20
Figure S14. HRESIMS spectrum of **2** 21
Figure S15. UV spectrum of **2** 22
Figure S16. IR spectrum of **2** 23
Table S3. ^{1}H and ^{13}C NMR data and key COSY and HMBC correlations of **3** 24
Figure S17. ^{1}H-NMR spectrum of **3** in DMSO-d_6 (500 MHz) 25
Figure S18. APT spectrum of **3** in DMSO-d_6 (125 MHz) 26
Figure S19. HSQC spectrum of **3** in DMSO-d_6 27
Figure S20. ^{1}H-^{1}H COSY spectrum of **3** in DMSO-d_6 28
Figure S21. HMBC spectrum of **3** in DMSO-d_6 29
Figure S22. HRESIMS spectrum of **3** 30
Figure S23. UV spectrum of **3** 31
Figure S24. IR spectrum of **3** 32
Table S4. ^{1}H and ^{13}C NMR data and key COSY and HMBC correlations of **4** 33
Figure S25. ^{1}H-NMR spectrum of **4** in DMSO-d_6 (500 MHz) 34
Figure S26. APT spectrum of **4** in DMSO-d_6 (125 MHz) 35
Figure S27. HSQC spectrum of **4** in DMSO-d_6 36
Figure S28. ^{1}H-^{1}H COSY spectrum of **4** in DMSO-d_6 37
Figure S29. HMBC spectrum of **4** in DMSO-d_6 38
Figure S30. HRESIMS spectrum of **4** 39
Figure S31. UV spectrum of **4** 40
Figure S32. IR spectrum of **4** 41
Table S5. ^{1}H and ^{13}C NMR data and key COSY and HMBC correlations of **5** 42
Figure S33. ^{1}H-NMR spectrum of **5** in DMSO-d_6 (500 MHz) 43
Figure S34. APT spectrum of **5** in DMSO-d_6 (125 MHz) 44
Figure S35. HSQC spectrum of **5** in DMSO-d_6 45
Figure S36. ^{1}H-^{1}H COSY spectrum of **5** in DMSO-d_6 46

Figure S37. HMBC spectrum of **5** in DMSO-d_6 47
Figure S38. HRESIMS spectrum of **5** 48
Figure S39. UV spectrum of **5** 49
Figure S40. IR spectrum of **5** 50
Table S6. ^{1}H and ^{13}C NMR data and key COSY and HMBC correlations of **6** 51
Figure S41. ^{1}H-NMR spectrum of **6** in DMSO-d_6 (400 MHz) 52
Figure S42. ^{13}C-NMR spectrum of **6** in DMSO-d_6 (100 MHz) 53
Figure S43. HSQC spectrum of **6** in DMSO-d_6 54
Figure S44. ^{1}H-^{1}H COSY spectrum of **6** in DMSO-d_6 55
Figure S45. HMBC spectrum of **6** in DMSO-d_6 56
Figure S46. HRESIMS spectrum of **6** 57
Figure S47. UV spectrum of **6** 58
Figure S48. IR spectrum of **6** 59
Figure S49. ^{1}H-NMR spectrum of **7** in DMSO-d_6 (500 MHz) 60
Figure S50. APT spectrum of **7** in DMSO-d_6 (125 MHz) 61
Figure S51. HSQC spectrum of **7** in DMSO-d_6 62
Figure S52. ^{1}H-^{1}H COSY spectrum of **7** in DMSO-d_6 63
Figure S53. HMBC spectrum of **7** in DMSO-d_6 64
Figure S54. HRESIMS spectrum of **7** 65
Figure S55. UV spectrum of **7** 66
Figure S56. IR spectrum of **7** 67
Table S7. ^{1}H and ^{13}C NMR data and key COSY and HMBC correlations of **7** 68
Figure S57. ^{1}H-NMR spectrum of **8** in DMSO-d_6 (500 MHz) 69
Figure S58. APT spectrum of **8** in DMSO-d_6 (125 MHz) 70
Figure S59. HSQC spectrum of **8** in DMSO-d_6 71
Figure S60. ^{1}H-^{1}H COSY spectrum of **8** in DMSO-d_6 72
Figure S61. HMBC spectrum of **8** in DMSO-d_6 73
Figure S62. HRESIMS spectrum of **8** 74
Figure S63. UV spectrum of **8** 75
Figure S64. IR spectrum of **8** 76
Table S8. ^{1}H and ^{13}C NMR data and key COSY and HMBC correlations of **8** 77
Figure S65. ^{1}H-NMR spectrum of **9** in DMSO-d_6 (400 MHz) 78
Figure S66. ^{13}C-NMR spectrum of **9** in DMSO-d_6 (100 MHz) 79
Figure S67. HSQC spectrum of **9** in DMSO-d_6 80
Figure S68. ^{1}H-^{1}H COSY spectrum of **9** in DMSO-d_6 81
Figure S69. HMBC spectrum of **9** in DMSO-d_6 82
Figure S70. HRESIMS spectrum of **9** 83
Figure S71. UV spectrum of **9** 84
Figure S72. IR spectrum of **9** 85
Table S9. ^{1}H and ^{13}C NMR data and key COSY and HMBC correlations of **9** 86
Figure S73. ^{1}H-NMR spectrum of **10** in DMSO-d_6 (500 MHz) 87
Figure S74. ^{13}C-NMR spectrum of **10** in DMSO-d_6 (125 MHz) 88

Figure S75. HSQC spectrum of **10** in DMSO-d_6 89
Figure S76. ^{1}H-^{1}H COSY spectrum of **10** in DMSO-d_6 90
Figure S77. HMBC spectrum of **10** in DMSO-d_6 91
Figure S78. HRESIMS spectrum of **10** 92
Figure S79. UV spectrum of **10** 93
Figure S80. IR spectrum of **10** 94
Table S10. ^{1}H and ^{13}C NMR data and key COSY and HMBC correlations of **10** 95
Figure S81. ^{1}H-NMR spectrum of **11** in DMSO-d_6 (600 MHz) 96
Figure S82. ^{13}C-NMR spectrum of **11** in DMSO-d_6 (150 MHz) 97
Figure S83. HSQC spectrum of **11** in DMSO-d_6 98
Figure S84. ^{1}H-^{1}H COSY spectrum of **11** in DMSO-d_6 99
Figure S85. HMBC spectrum of **11** in DMSO-d_6 100
Figure S86. HRESIMS spectrum of **11** 101
Figure S87. UV spectrum of **11** 102
Figure S88. IR spectrum of **11** 103
Table S11. ^{1}H and ^{13}C NMR data and key COSY and HMBC correlations of **11** 104
Figure S89. ^{1}H-NMR spectrum of **12** in DMSO-d_6 (500 MHz) 105
Figure S90. APT spectrum of **12** in DMSO-d_6 (125 MHz) 106
Figure S91. HSQC spectrum of **12** in DMSO-d_6 107
Figure S92. ^{1}H-^{1}H COSY spectrum of **12** in DMSO-d_6 108
Figure S93. HMBC spectrum of **12** in DMSO-d_6 109
Figure S94. HRESIMS spectrum of **12** 110
Figure S95. UV spectrum of **12** 111
Figure S96. IR spectrum of **12** 112
Figure S97. ^{1}H-NMR spectrum of **13** in DMSO-d_6 (500 MHz) 113
Figure S98. APT spectrum of **13** in DMSO-d_6 (125 MHz) 114
Figure S99. HSQC spectrum of **13** in DMSO-d_6 115
Figure S100. ^{1}H-^{1}H COSY spectrum of **13** in DMSO-d_6 116
Figure S101. HMBC spectrum of **13** in DMSO-d_6 117
Figure S102. HRESIMS spectrum of **13** 118
Figure S103. UV spectrum of **13** 119
Figure S104. IR spectrum of **13** 120
Figure S105. ^{1}H-NMR spectrum of **14** in DMSO-d_6 (500 MHz) 121
Figure S106. APT spectrum of **14** in DMSO-d_6 (125 MHz) 122
Figure S107. HSQC spectrum of **14** in DMSO-d_6 123
Figure S108. ^{1}H-^{1}H COSY spectrum of **14** in DMSO-d_6 124
Figure S109. HMBC spectrum of **14** in DMSO-d_6 125
Figure S110. HRESIMS spectrum of **14** 126
Figure S111. UV spectrum of **14** 127
Figure S112. IR spectrum of **14** 128
Figure S113. ^{1}H-NMR spectrum of **15** in DMSO-d_6 (500 MHz) 129
Figure S114. APT spectrum of **15** in DMSO-d_6 (125 MHz) 130

Figure S115. HSQC spectrum of **15** in DMSO-d_6 131
Figure S116. ^{1}H-^{1}H COSY spectrum of **15** in DMSO-d_6 132
Figure S117. HMBC spectrum of **15** in DMSO-d_6 133
Figure S118. HRESIMS spectrum of **15** 134
Figure S119. UV spectrum of **15** 135
Figure S120. IR spectrum of **15** 136
Figure S121. ^{1}H-NMR spectrum of **16** in DMSO-d_6 (500 MHz) 137
Figure S122. ^{13}C-NMR spectrum of **16** in DMSO-d_6 (125 MHz) 138
Figure S123. HSQC spectrum of **16** in DMSO-d_6 139
Figure S124. ^{1}H-^{1}H COSY spectrum of **16** in DMSO-d_6 140
Figure S125. HMBC spectrum of **16** in DMSO-d_6 141
Figure S126. HRESIMS spectrum of **16** 142
Figure S127. UV spectrum of **16** 143
Figure S128. IR spectrum of **16** 144
Table S12. Comparison of ^{1}H NMR data of **12-16** with spiromastixones P-T 145
Table S13. Comparison of ^{13}C NMR data of **12-16** with spiromastixones P-T 146

Table S1. ^{1}H and ^{13}C NMR data and key COSY and HMBC correlations of 1

No.	δ_H	δ_C	COSY	HMBC
1		111.1, qC		
2		161.7, qC		
3	6.80, s	105.3, CH		C-1, C-2, C-4, C-5
4		159.7, qC		
5		113.6, qC		
6		146.9, qC		
7		162.9, qC		
8	2.81, t (7.9)	36.0, CH_2	H_2-9	C-1, C-5, C-6, C-9, C-10
9	1.54, m	23.0, CH_2	H_2-8, H_3-10	C-6, C-8, C-10
10	0.89, t (7.3)	14.3, CH_3	H_2-9	C-8, C-9
1′		141.3, C		
2′		144.6, C		
3′	6.50, d (2.7)	105.5, CH	H-5'	C-1', C-2', C-4', C-5'
4′		155.2, C		
5′	6.47, d (2.7)	113.2, CH	H-3'	C-1', C-3', C-4', C-7'
6′		135.8, C		
7′	2.65, t (7.8)	31.4, CH_2	H_2-8'	C-1', C-5', C-6', C-8', C-9'
8′	1.57, m	23.7, CH_2	H_2-7', H_3-9'	C-6', C-7', C-9'
9′	0.97, t (7.3)	14.4, CH_3	H_2-8'	C-7', C-8'

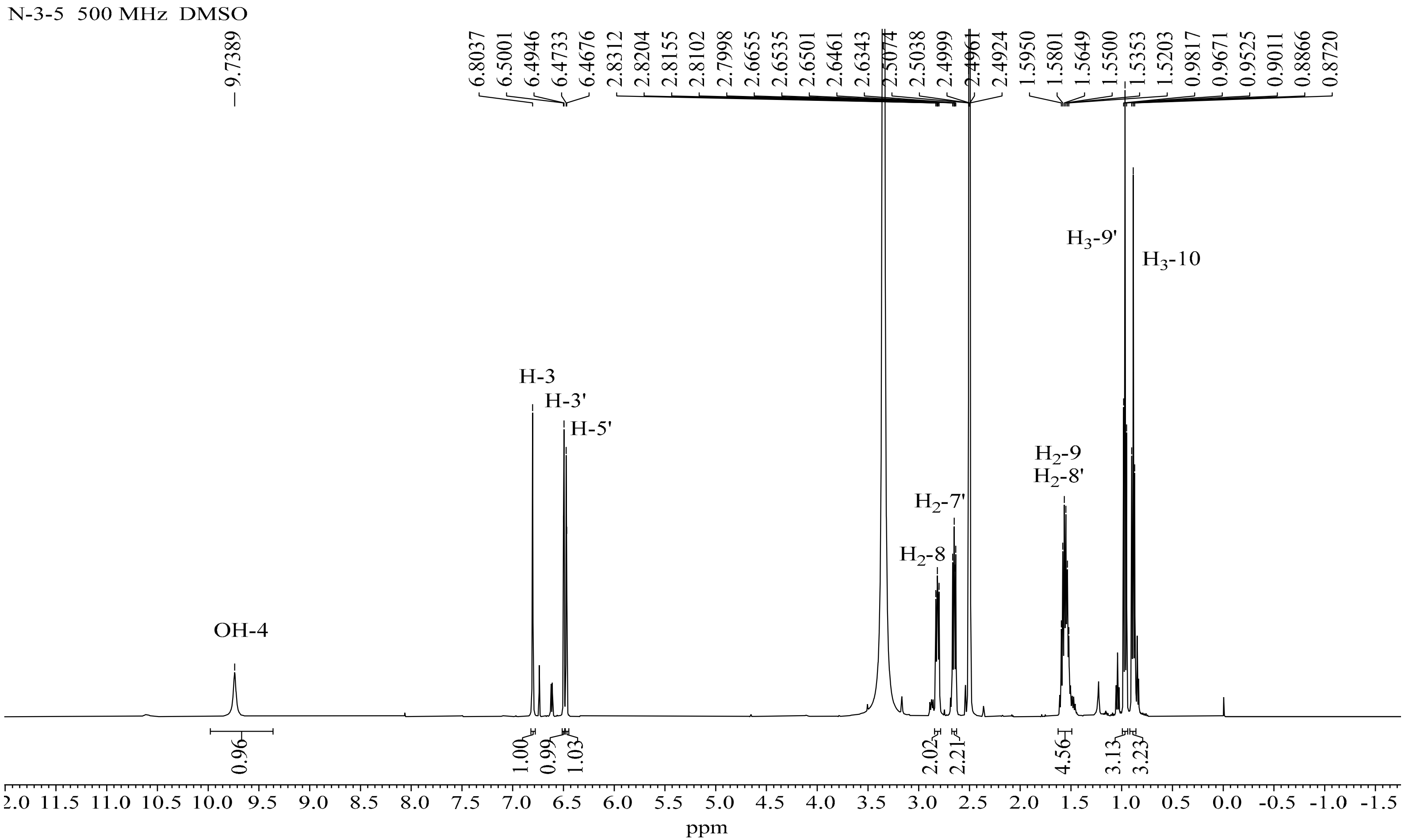

Figure S1. ^{1}H-NMR spectrum of **1** in DMSO-d_6 (500 MHz)

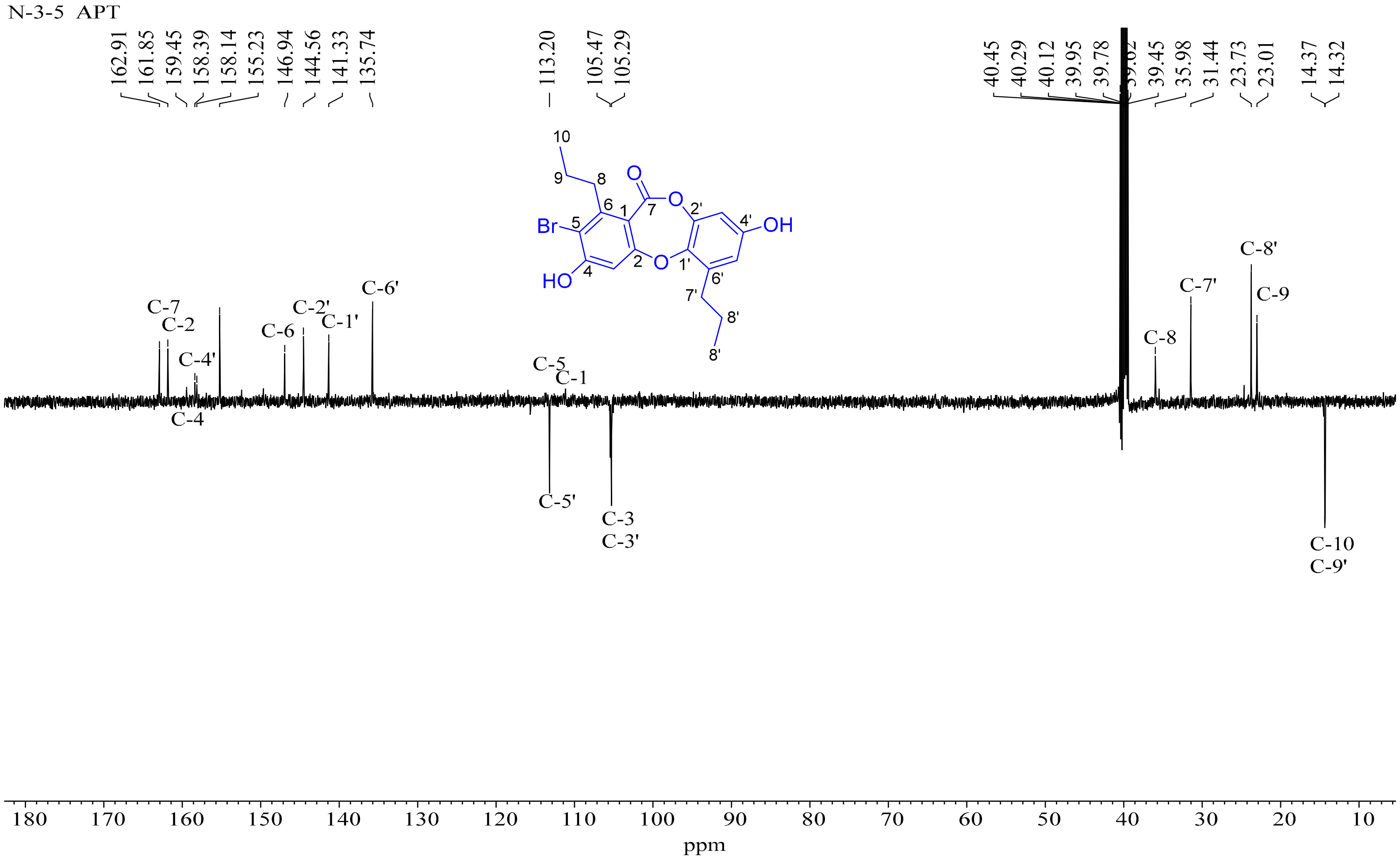

Figure S2. APT spectrum of **1** in DMSO-d_6 (125 MHz)

N-3-5 HSQC

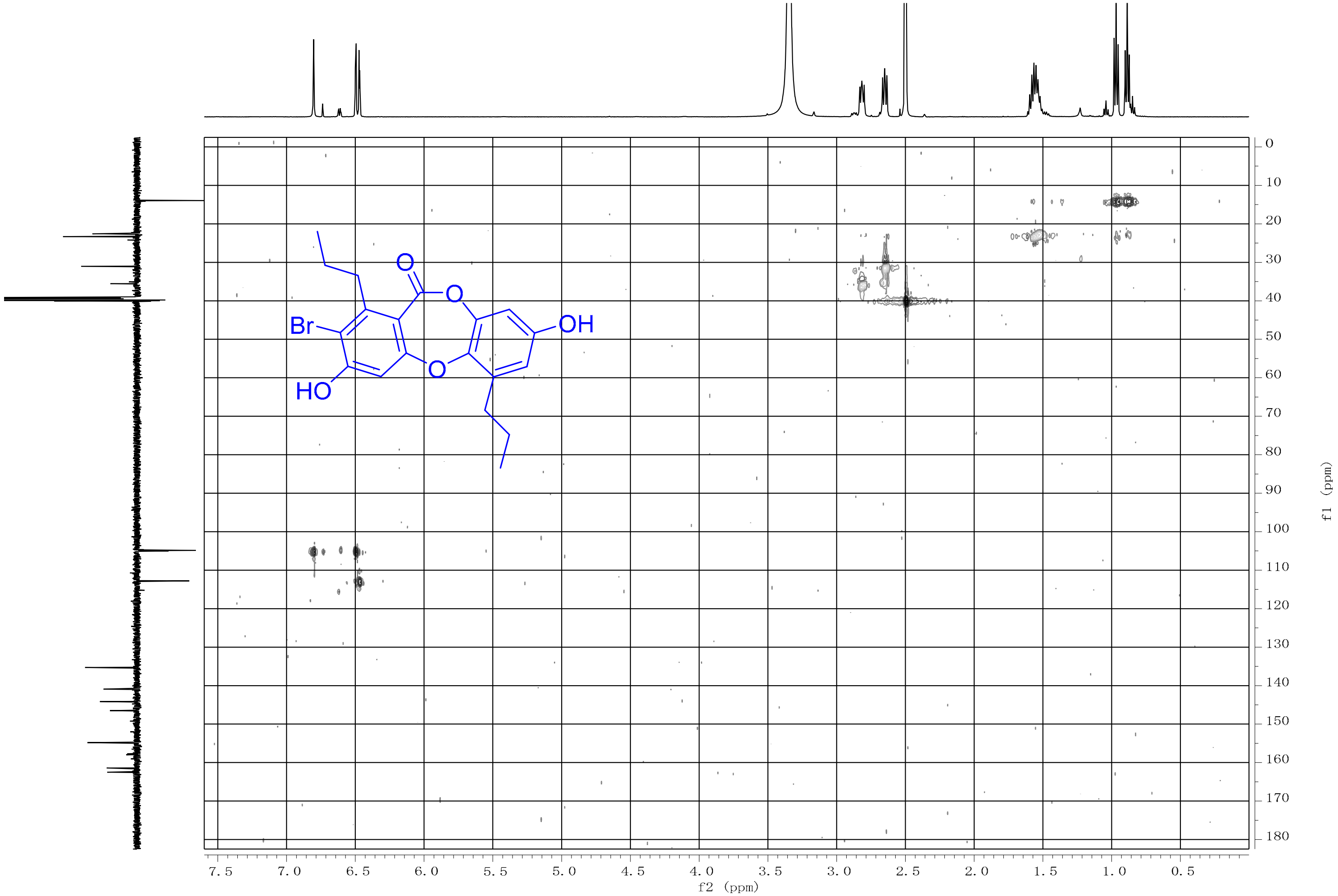

Figure S3. HSQC spectrum of **1** in DMSO-d_6

N-3-5 COSY

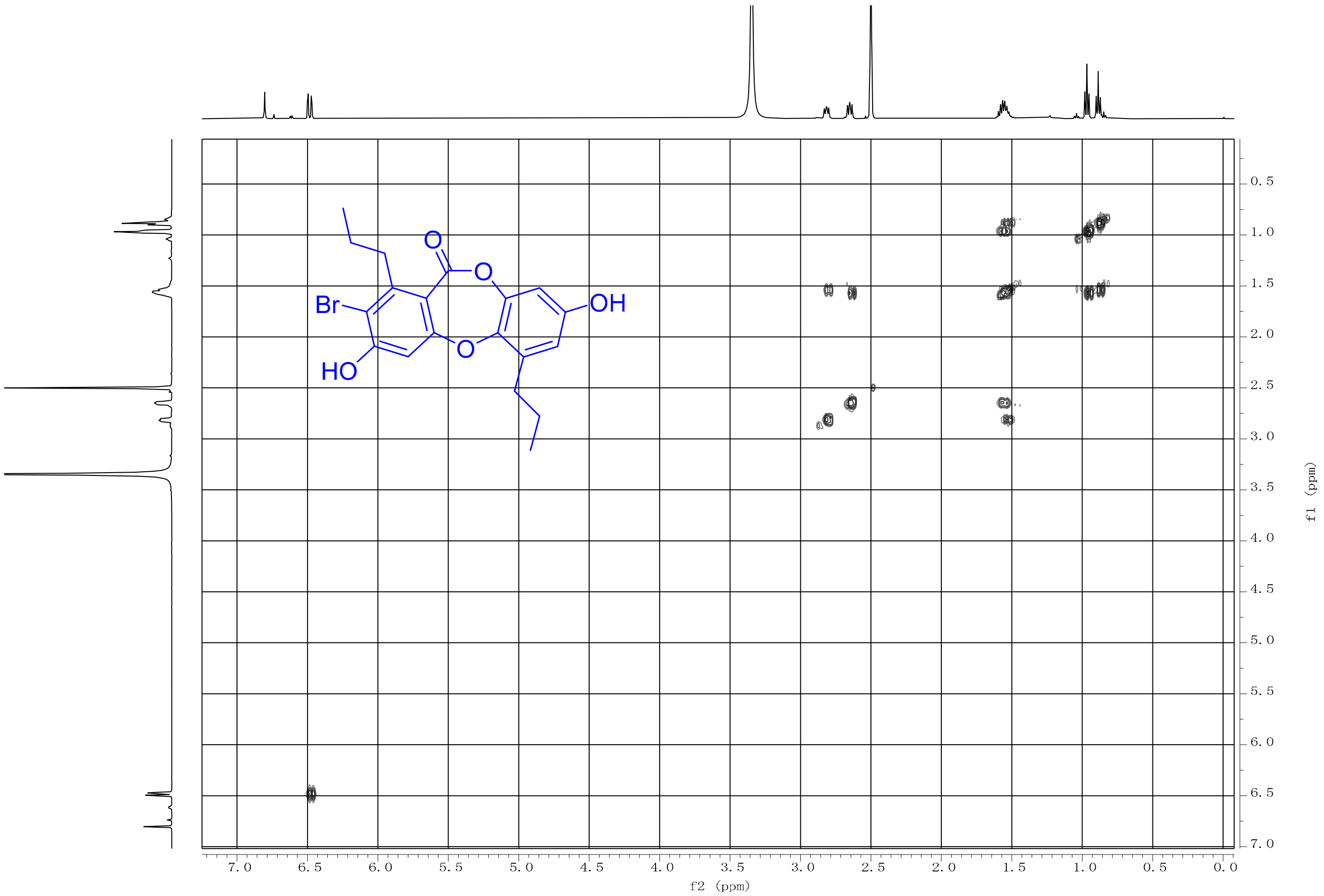

Figure S4. ^{1}H-^{1}H COSY spectrum of **1** in DMSO-d_6

N-3-5 HMBC

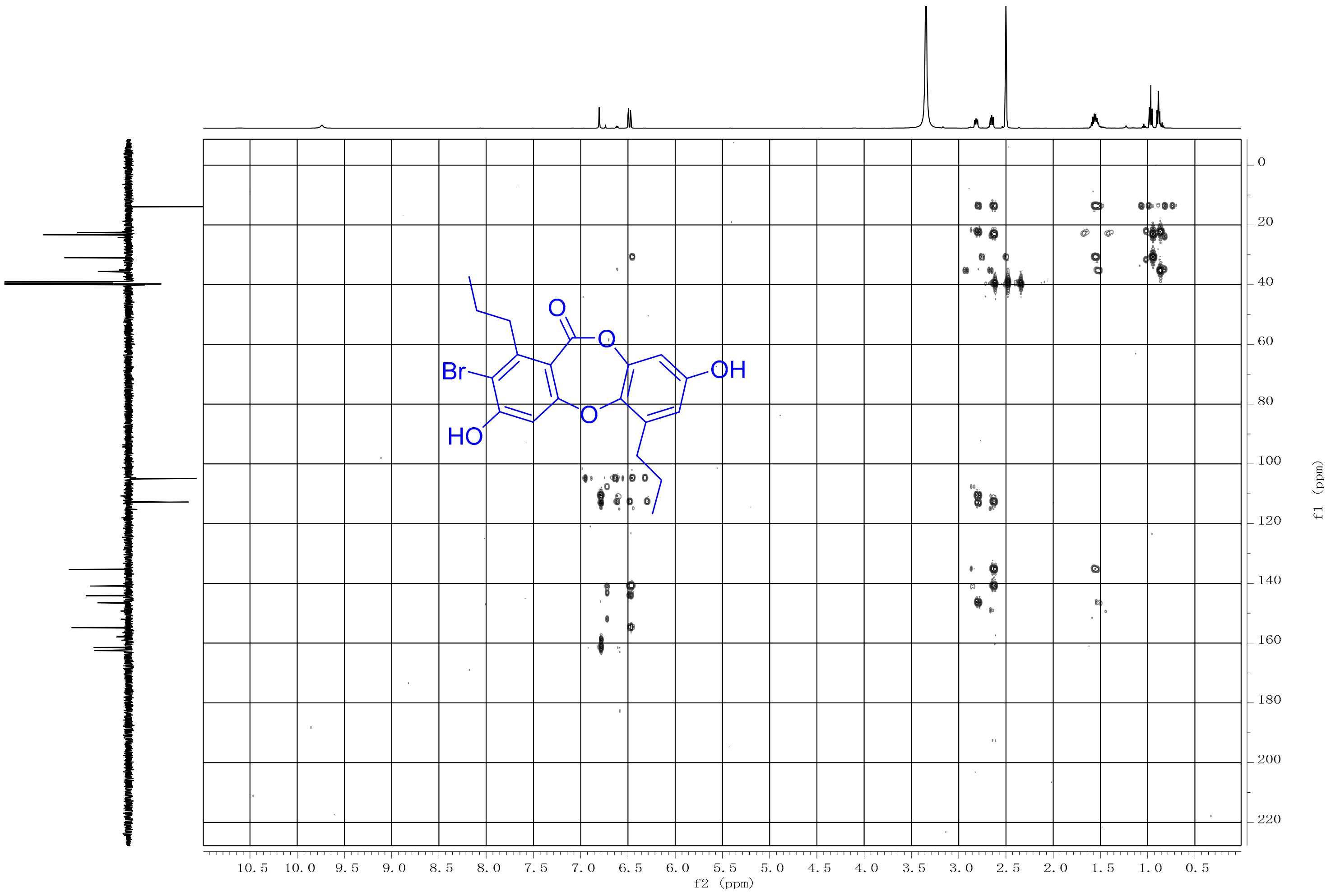

Figure S5. HMBC spectrum of **1** in DMSO-d_6

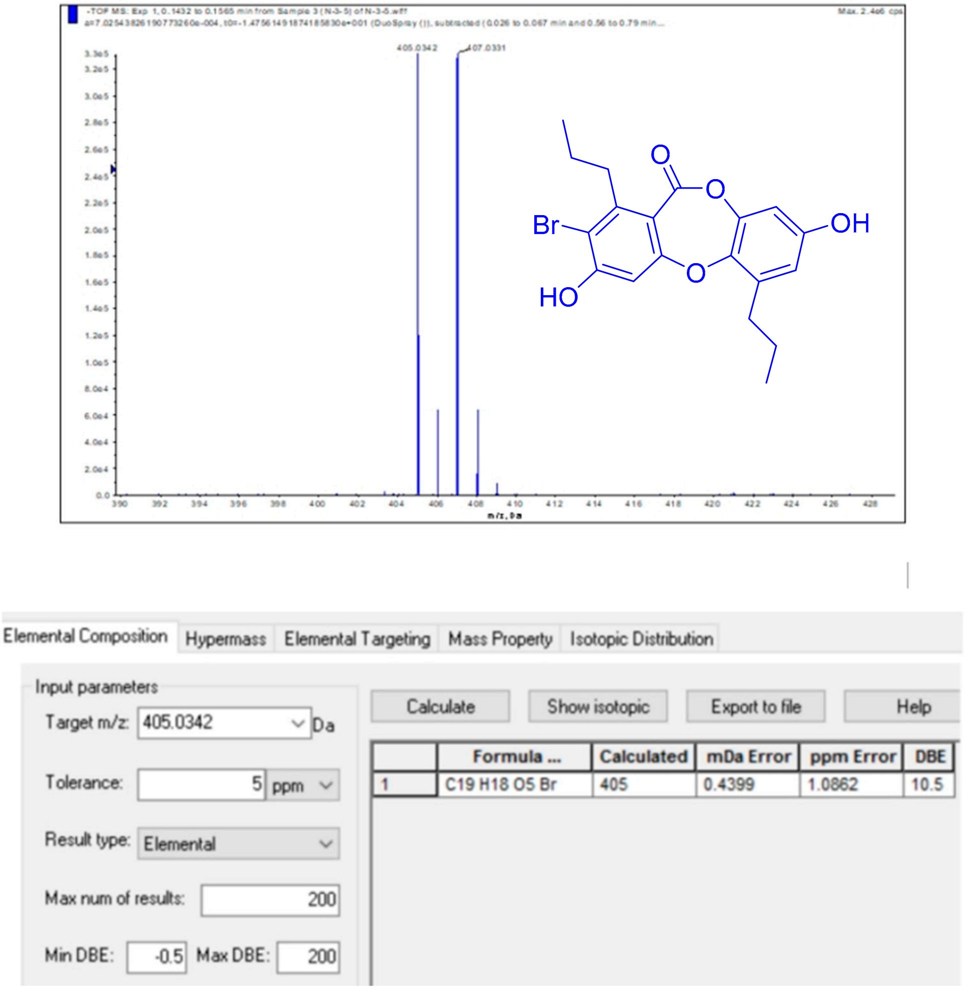

Figure S6. HRESIMS spectrum of **1**

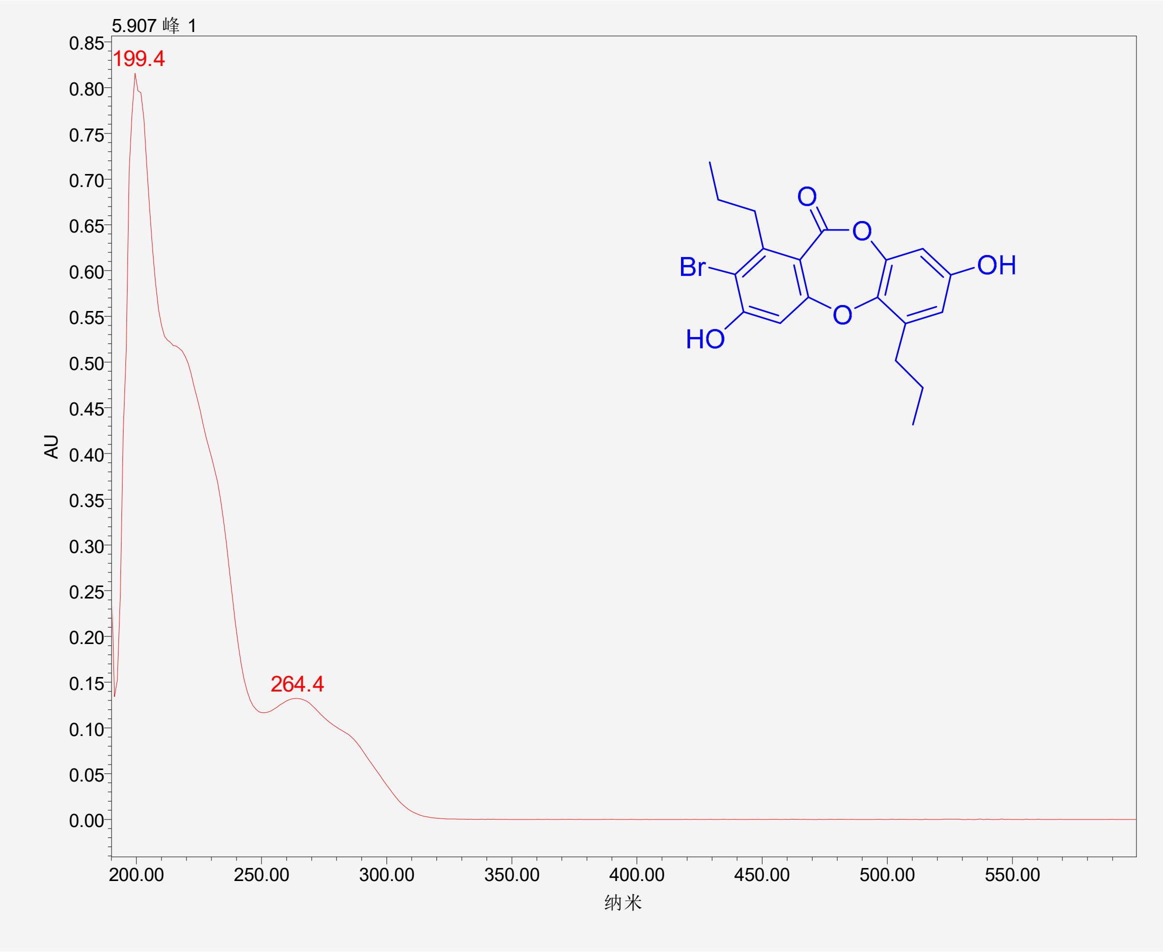

Figure S7. UV spectrum of **1**

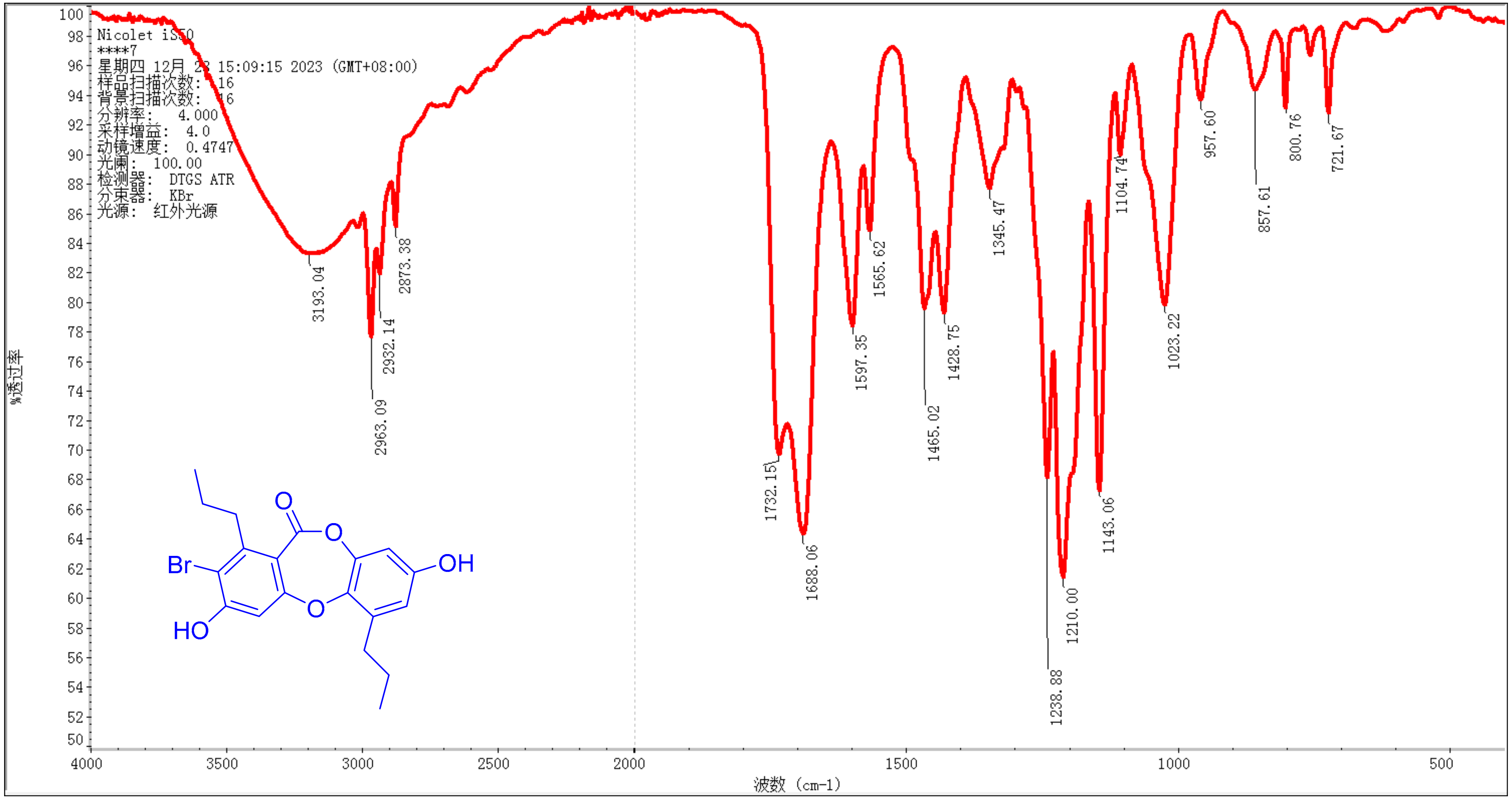

Figure S8. IR spectrum of **1**

Table S2. ^{1}H and ^{13}C NMR data and key COSY and HMBC correlations of 2

No.	δ_H	δ_C	COSY	HMBC
1		112.5, C		
2		160.4, C		
3		98.9, C		
4		159.6, C		
5	6.79, s	115.1, CH		C-1, C-3, C-4, C-8
6		148.5, C		
7		162.1, C		
8	2.66, t (7.8)	35.7, CH_2		C-1, C-5, C-6, C-9, C-10
9	1.49, m	24.5, CH_2		C-6, C-8, C-10
10	0.86, t (7.3)	14.3, CH_3		C-8, C-9
1′		142.0, C		
2′		143.4, C		
3′	6.77, s	105.6, CH		C-1', C-2', C-4', C-5'
4′		152.9, C		
5′		108.8, C		
6′		136.0, C		
7′	3.13, t (8.0)	33.0, CH_2		C-1', C-5', C-6', C-8', C-9'
8′	1.48, m	23.1, CH_2		C-6', C-7', C-9'
9′	1.00, t (7.3)	14.4, CH_3		C-7', C-8'

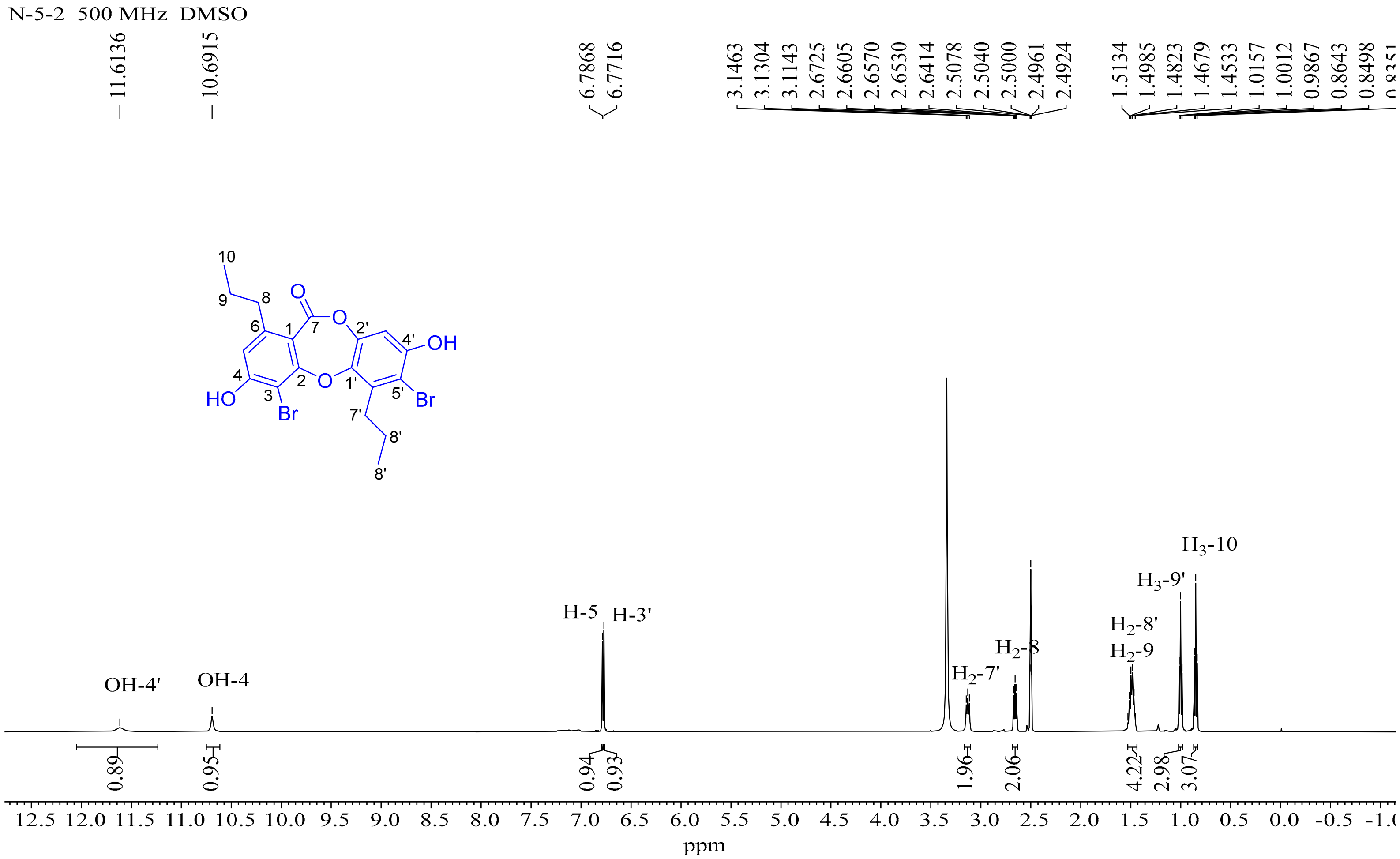

Figure S9. ^{1}H-NMR spectrum of **2** in DMSO-d_6 (500 MHz)

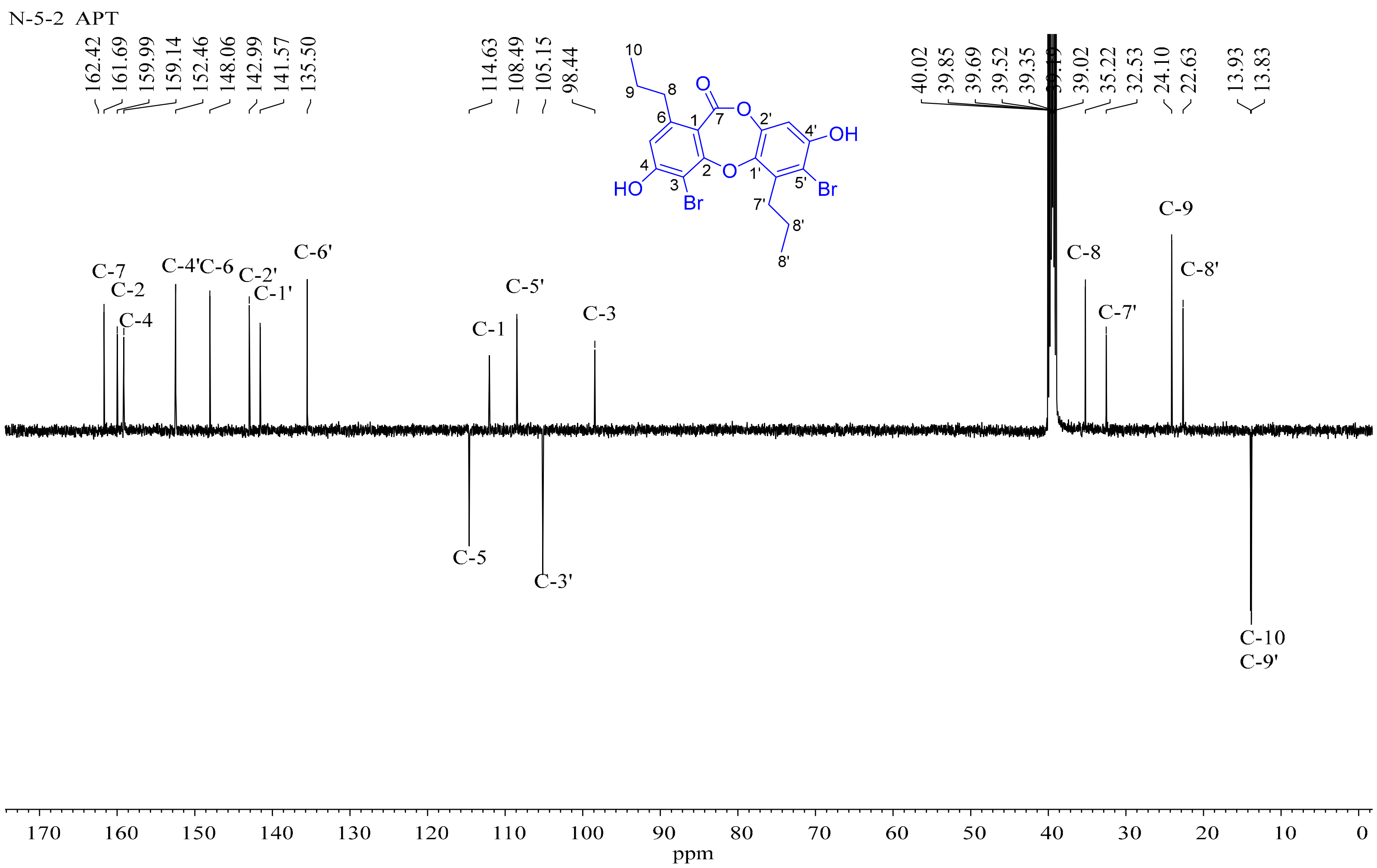

Figure S10. APT spectrum of **2** in DMSO-d_6 (125 MHz)

N-5-2 HSQC

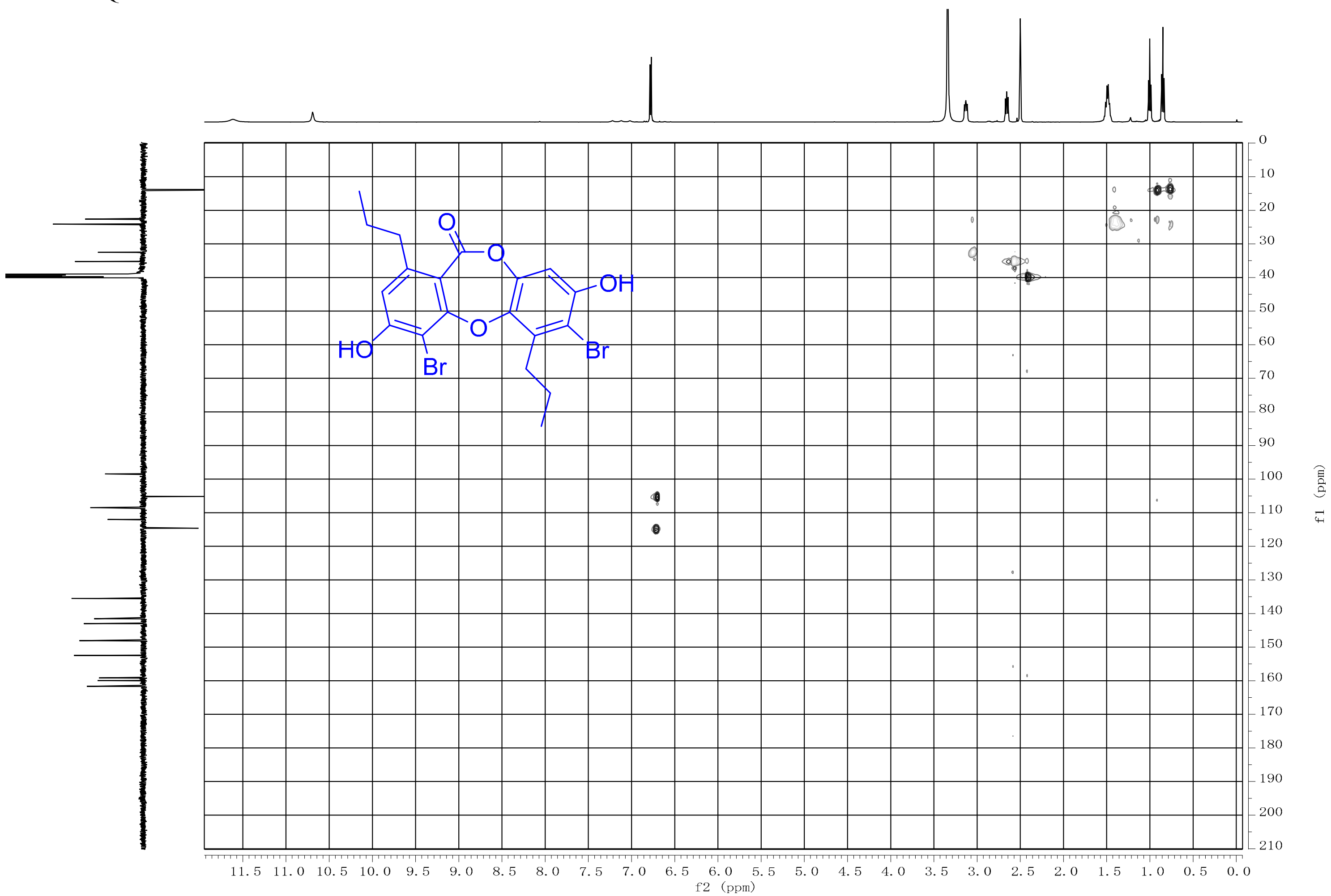

Figure S11. HSQC spectrum of **2** in DMSO-d_6

N-5-2 COSY

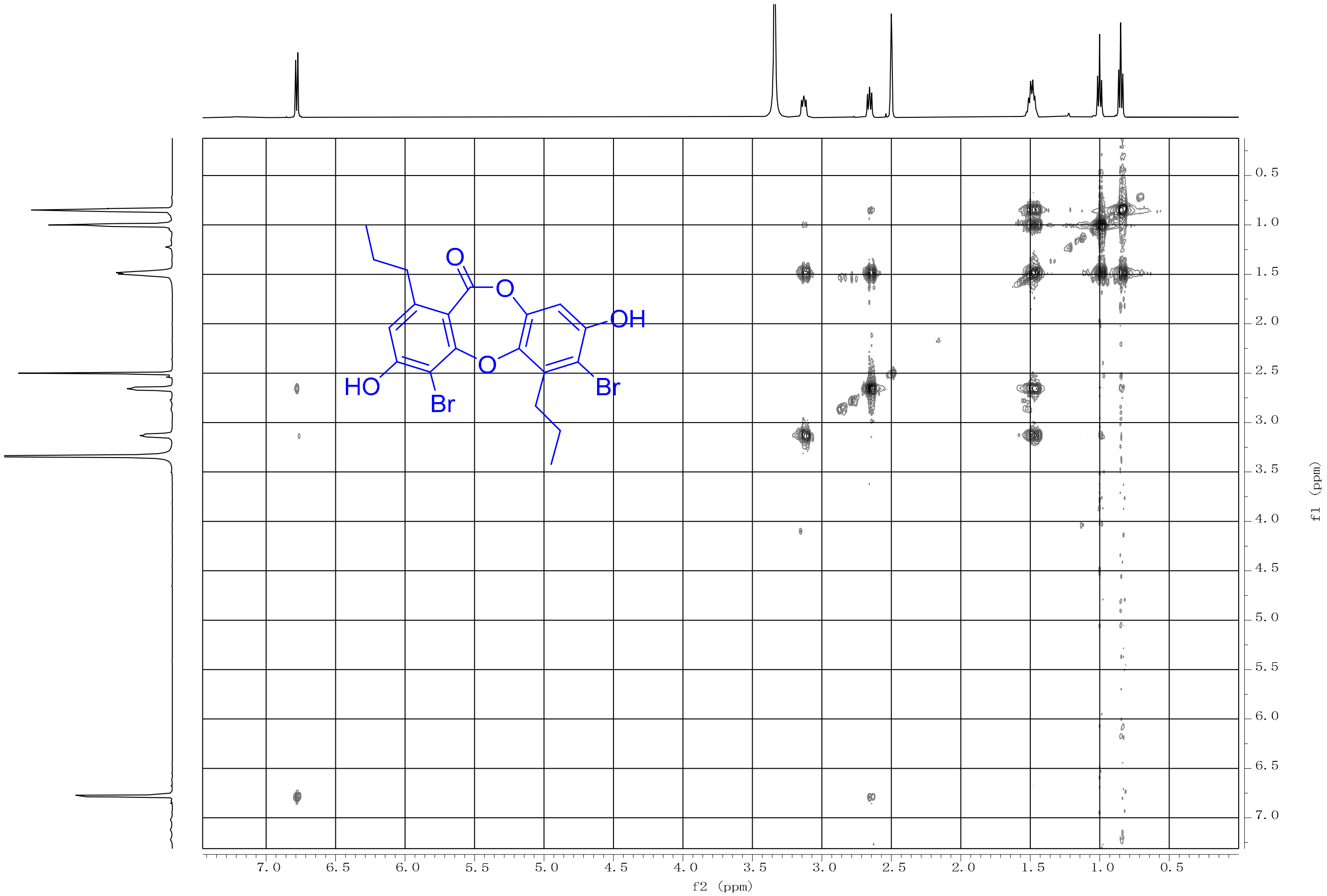

Figure S12. ^{1}H-^{1}H COSY spectrum of **2** in DMSO-d_6

N-5-2 HMBC

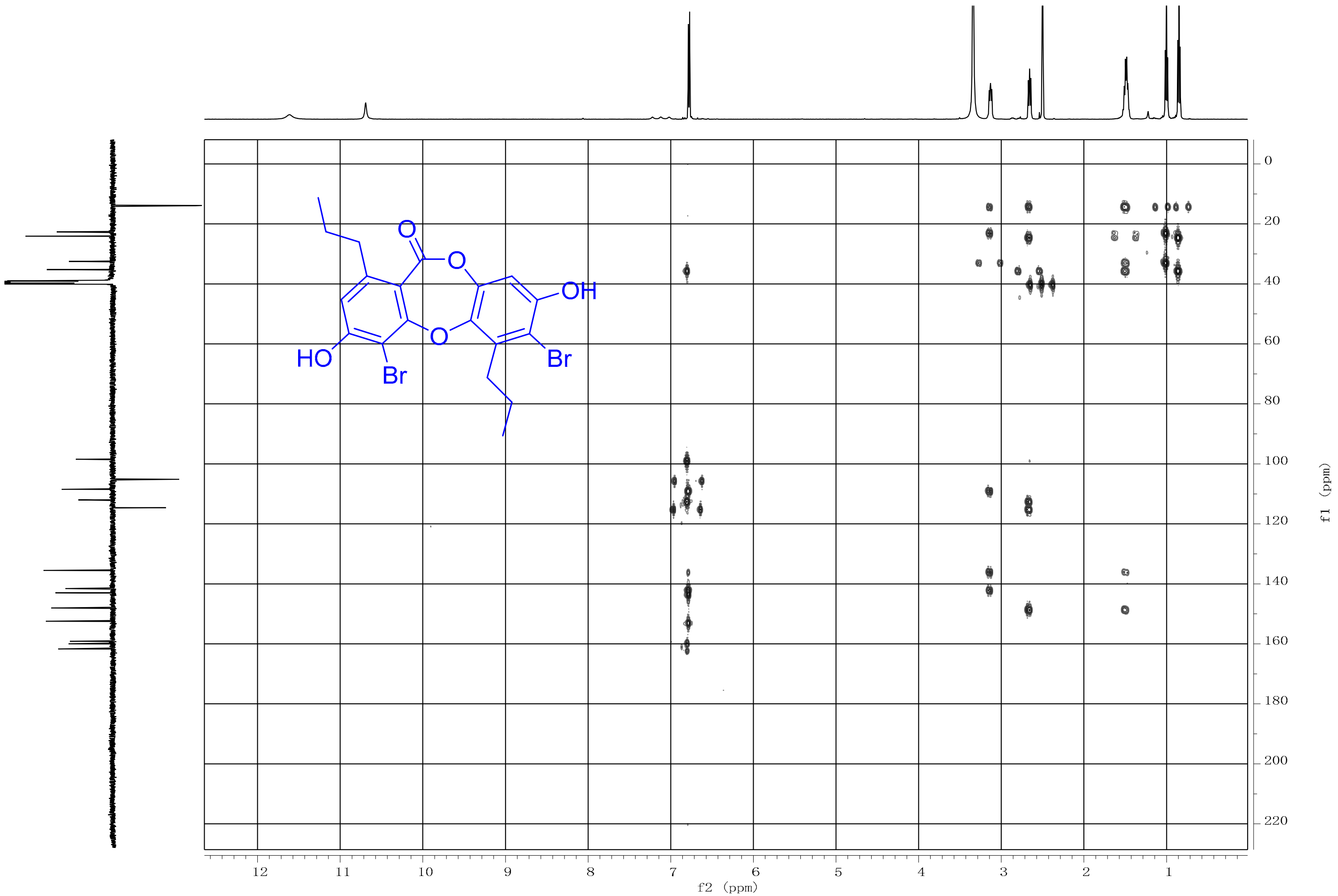

Figure S13. HMBC spectrum of **2** in DMSO-d_6

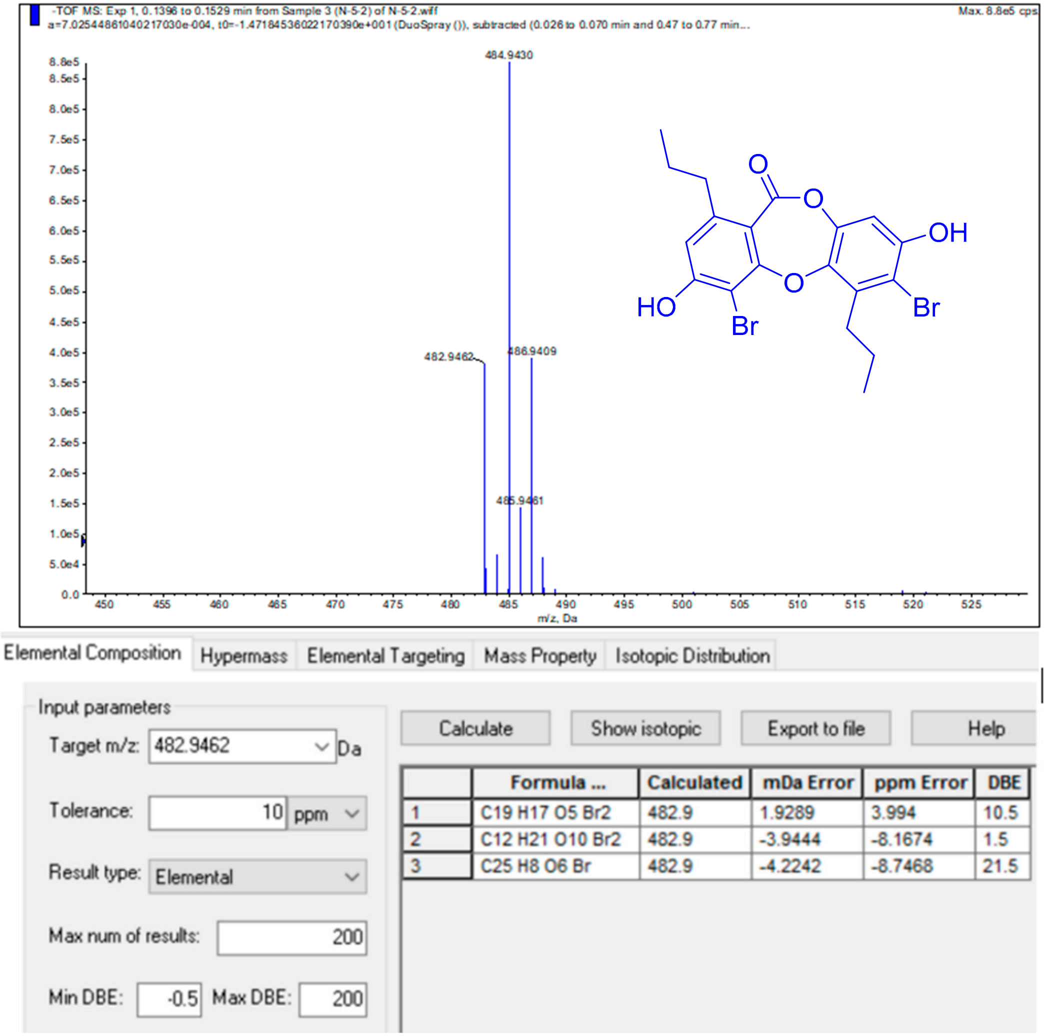

	Formula ...	Calculated	mDa Error	ppm Error	DBE
1	C19 H17 O5 Br2	482.9	1.9289	3.994	10.5
2	C12 H21 O10 Br2	482.9	-3.9444	-8.1674	1.5
3	C25 H8 O6 Br	482.9	-4.2242	-8.7468	21.5

Figure S14. HRESIMS spectrum of **2**

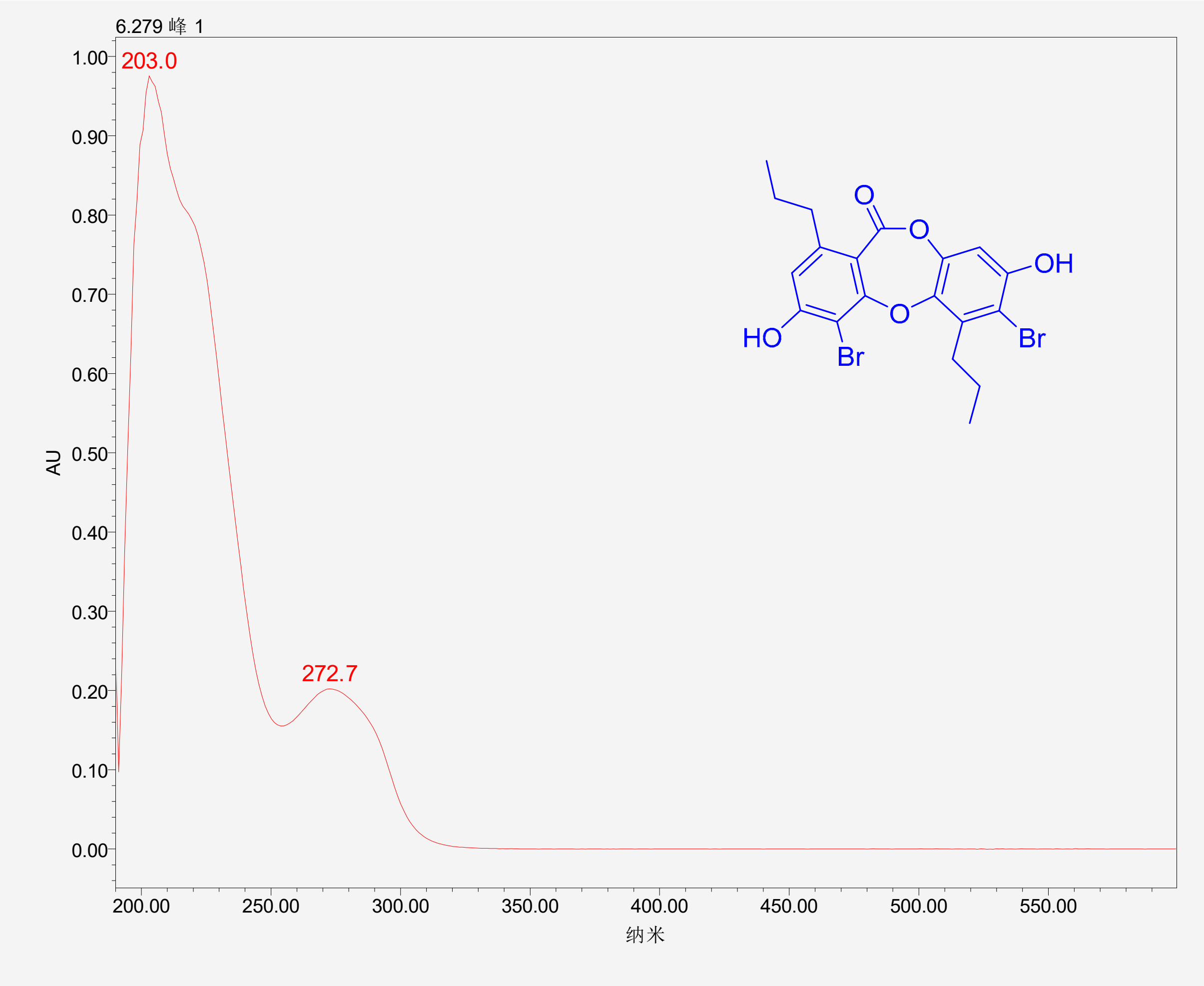

Figure S15. UV spectrum of **2**

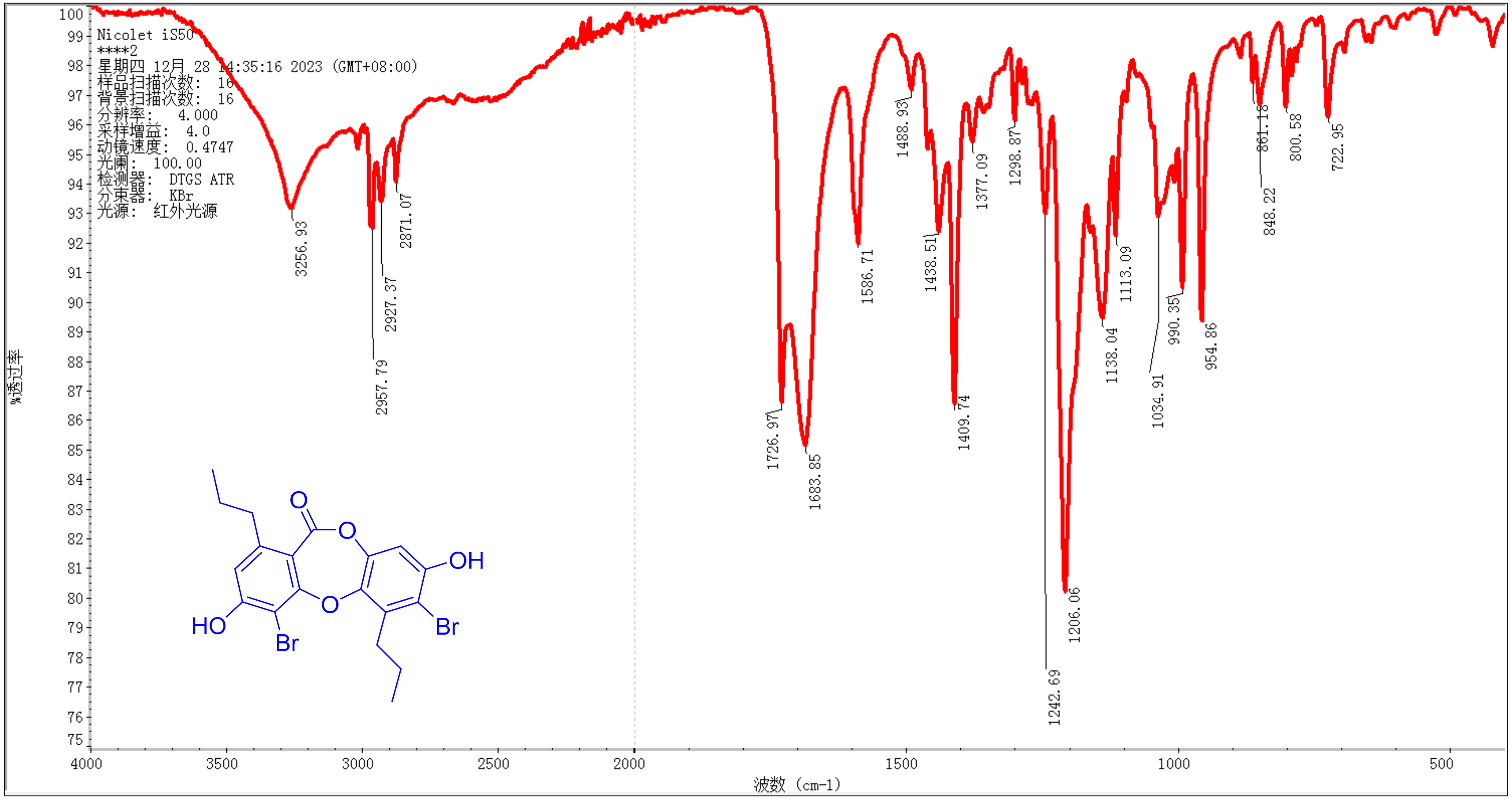

Figure S16. IR spectrum of **2**

Table S3. ^{1}H and ^{13}C NMR data and key COSY and HMBC correlations of 3

No.	δ_H	δ_C	COSY	HMBC
1		112.3, C		
2		160.6, C		
3		99.0, C		
4		159.7, C		
5	6.80, s	115.3, C		C-1, C-3, C-4, C-8
6		148.1, C		
7		161.8, C		
8	2.70, t (7.6)	35.5, CH_2		C-1, C-5, C-6, C-9, C-10
9	1.49, m	24.6, CH_2		C-6, C-8, C-10
10	0.83, t (7.2)	14.1, CH_3		C-8, C-9
1′		142.7, C		
2′		142.9, C		
3′		99.5, C		
4′		153.0, C		
5′	6.69, s	113.3, CH		C-1', C-4', C-5', C-7’
6′		134.8, C		
7′	2.90, t (7.8)	32.7, CH_2		C-1', C-5', C-6', C-8', C-9'
8′	1.47, m	24.2, CH_2		C-6', C-7', C-9'
9′	0.95, t (7.2)	14.1, CH_3		C-7', C-8'

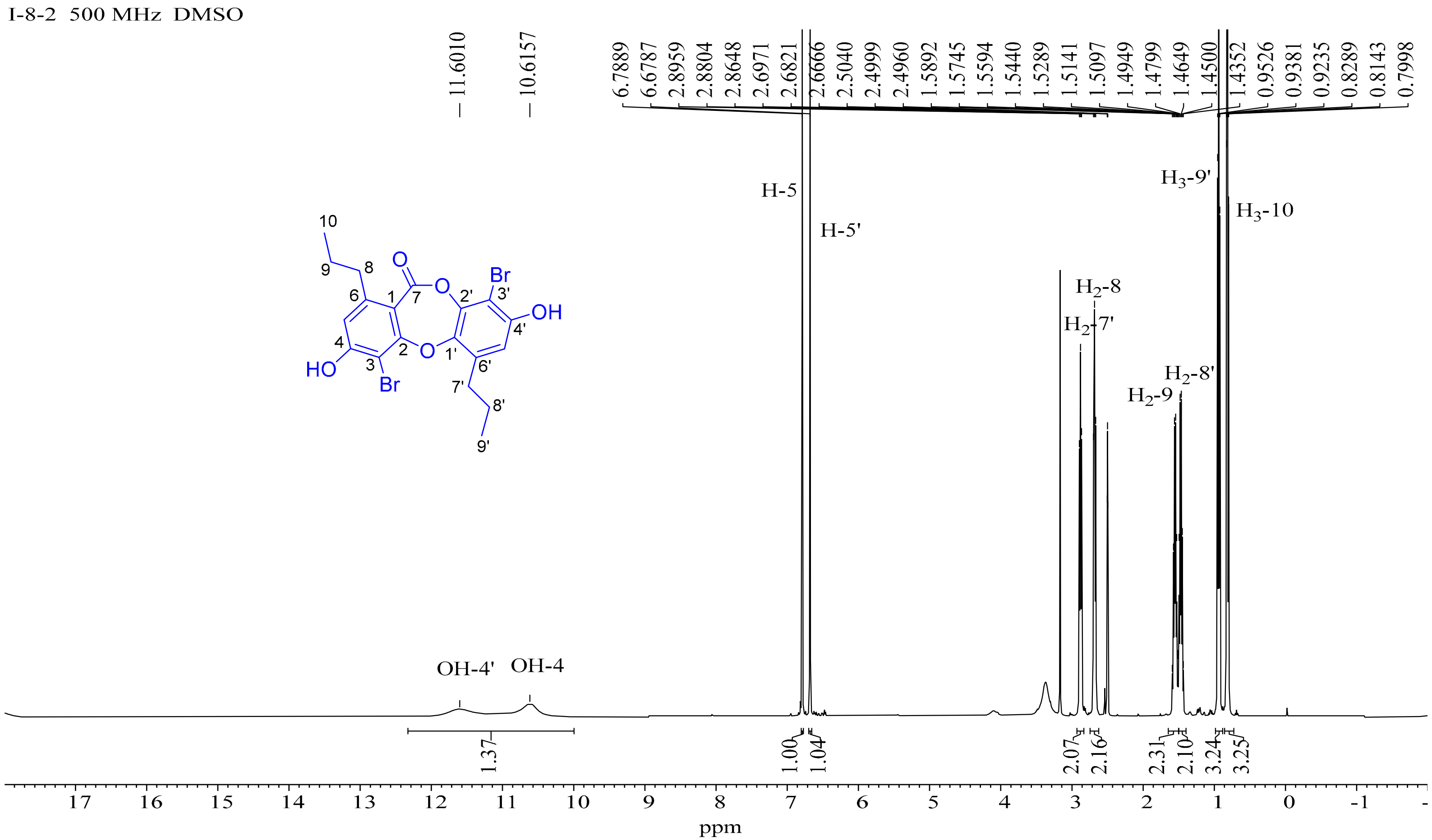

Figure S17. ^{1}H-NMR spectrum of **3** in DMSO-d_6 (500 MHz)

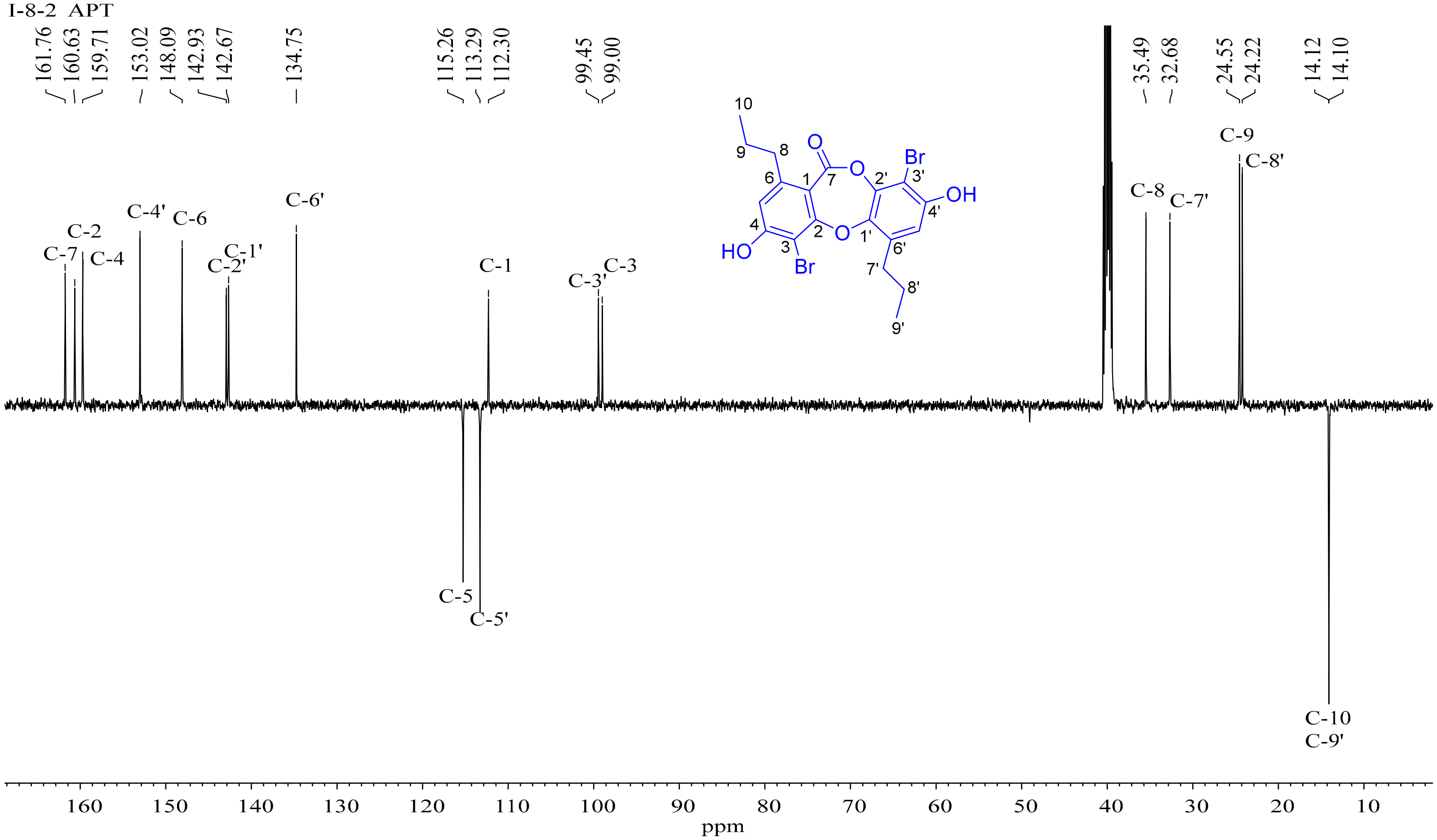

Figure S18. APT spectrum of **3** in DMSO-d_6 (125 MHz)

I-8-2 HSQC

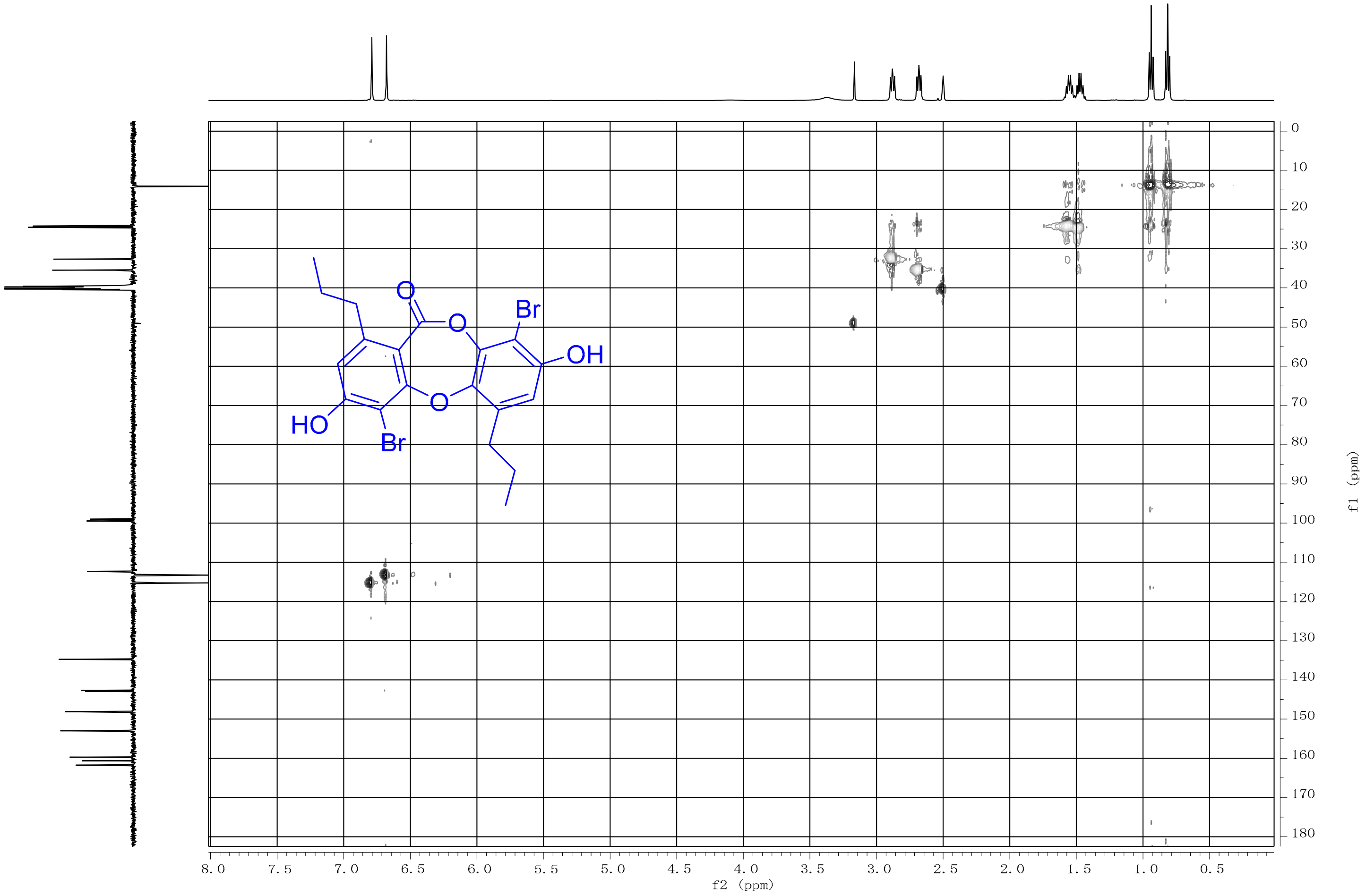

Figure S19. HSQC spectrum of **3** in DMSO-d_6

I-8-2 COSY

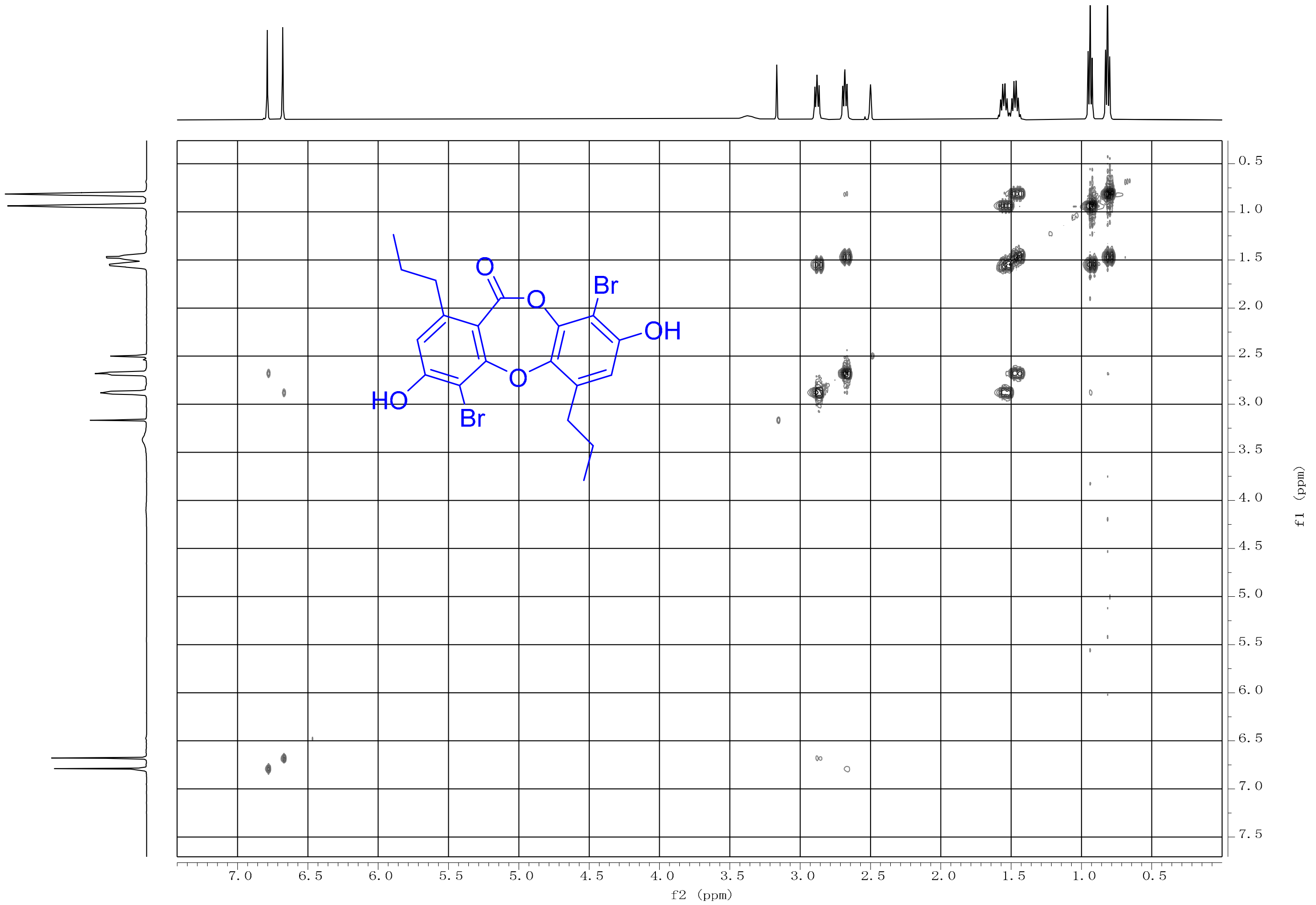

Figure S20. ^{1}H-^{1}H COSY spectrum of **3** in DMSO-d_6

I-8-2 HMBC

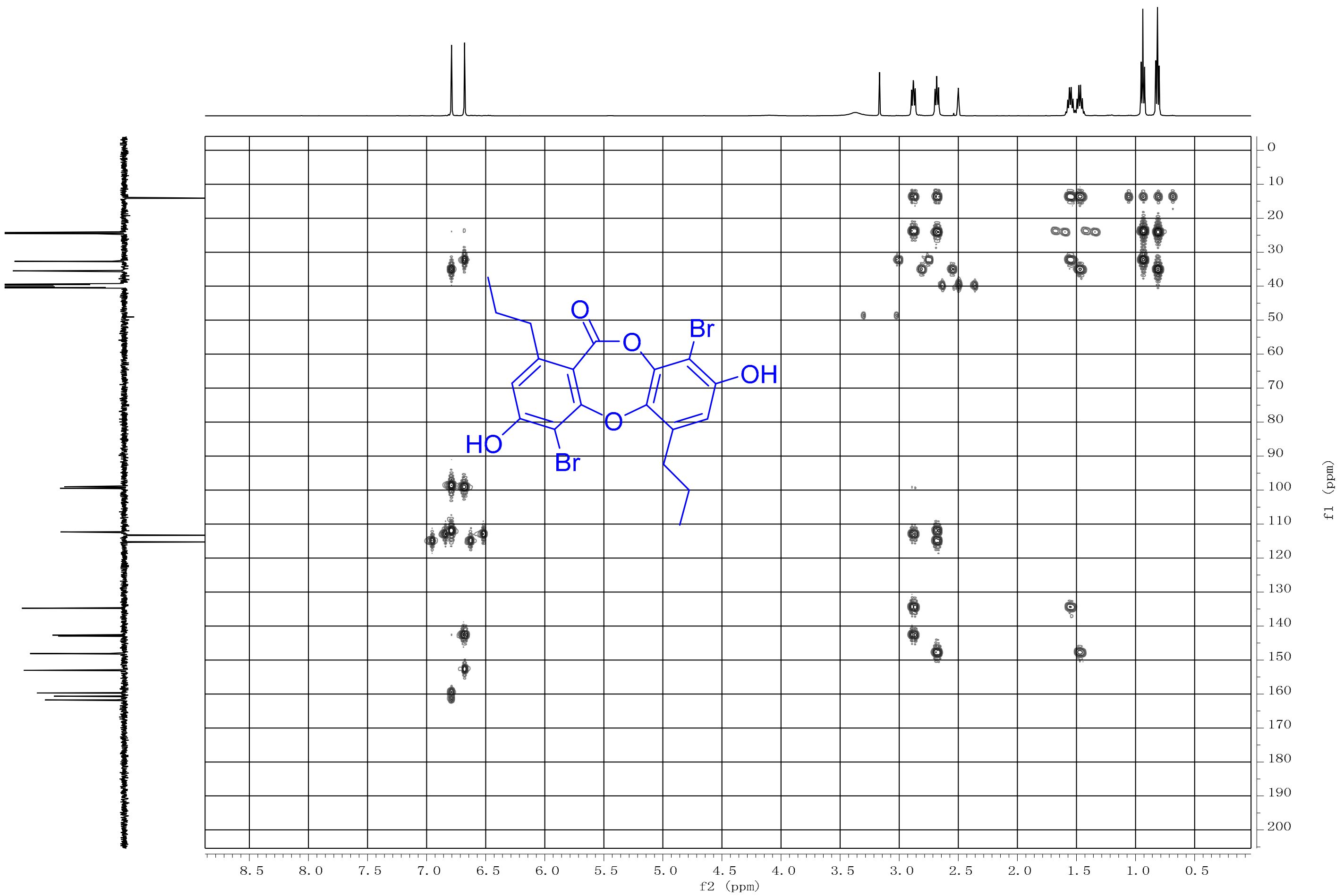

Figure S21. HMBC spectrum of **3** in DMSO-d_6

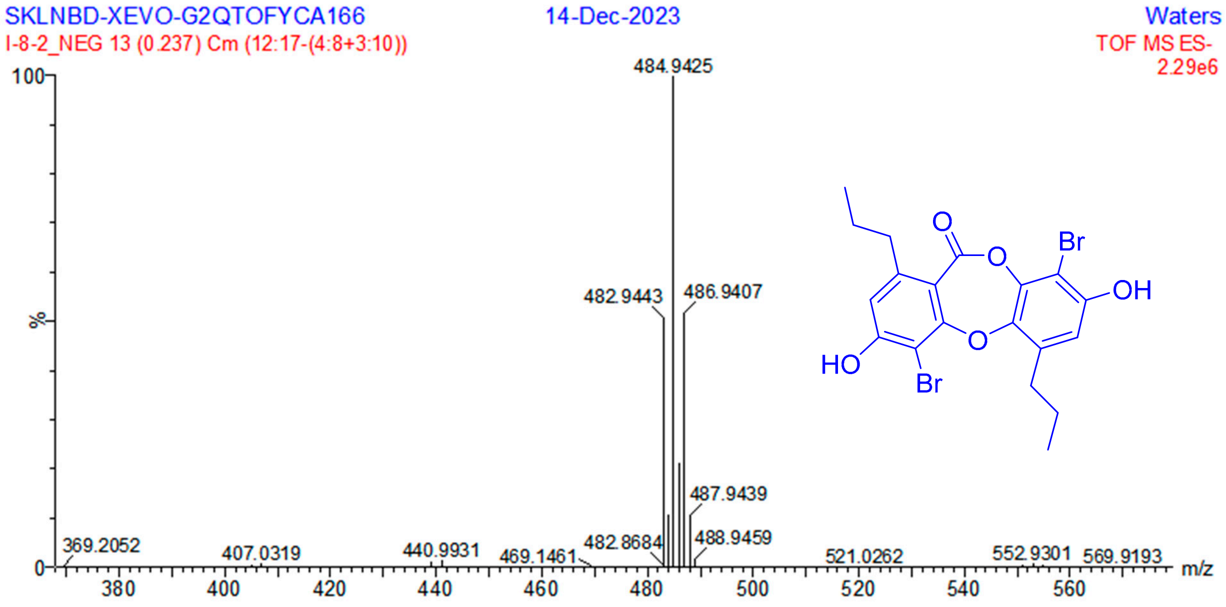

Maximum:		3.0	5.0	120.0				
Mass	Calc. Mass	mDa	PPM	DBE	i-FIT	Norm	Conf(%)	Formula
482.9443	482.9443	0.0	0.0	10.5	647.6	0.000	100.00	$C_{19}H_{17}O_5Br_2$
	482.9446	-0.3	-0.6	30.5	669.0	21.462	0.00	$C_{32}H_4OBr$

Figure S22. HRESIMS spectrum of **3**

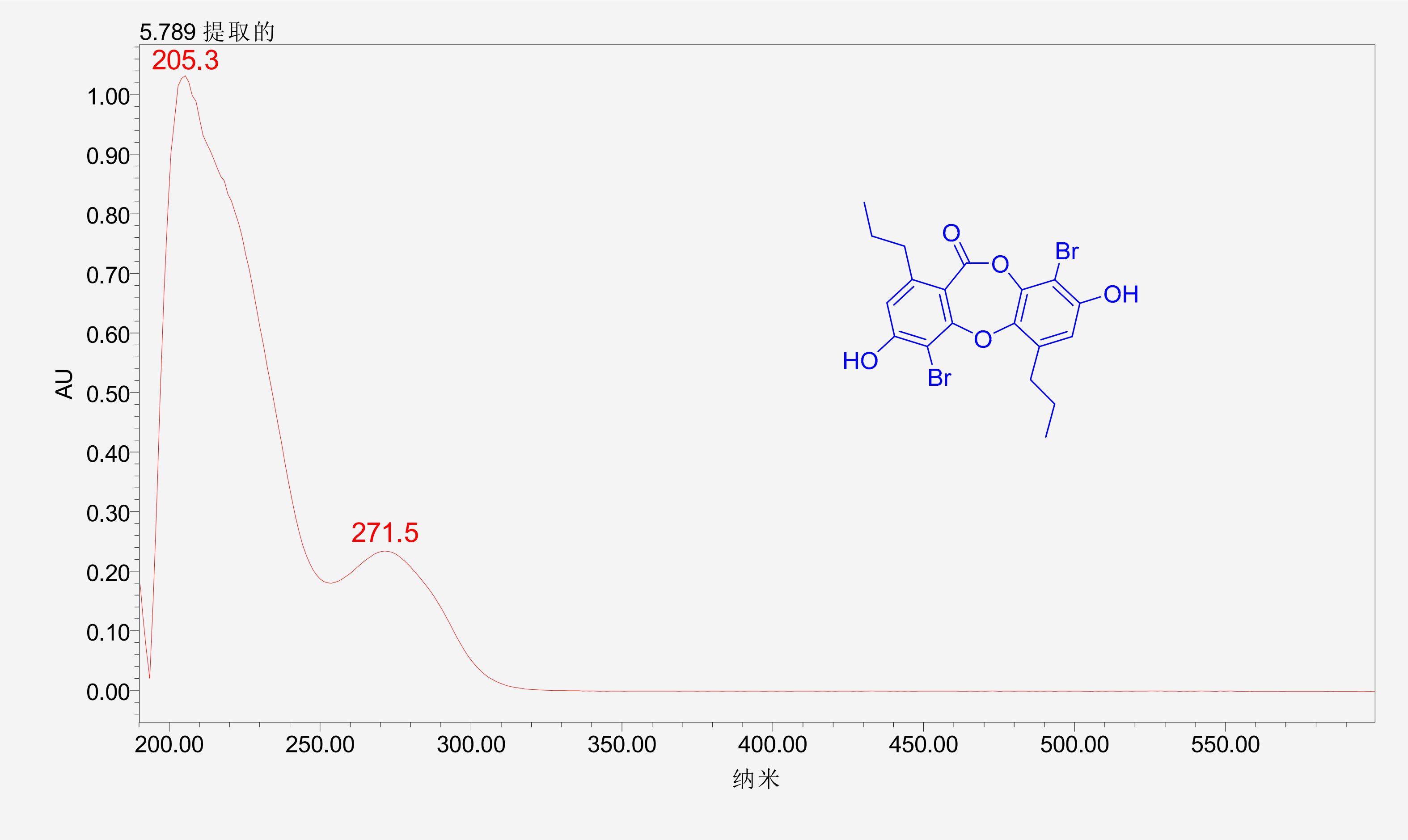

Figure S23. UV spectrum of **3**

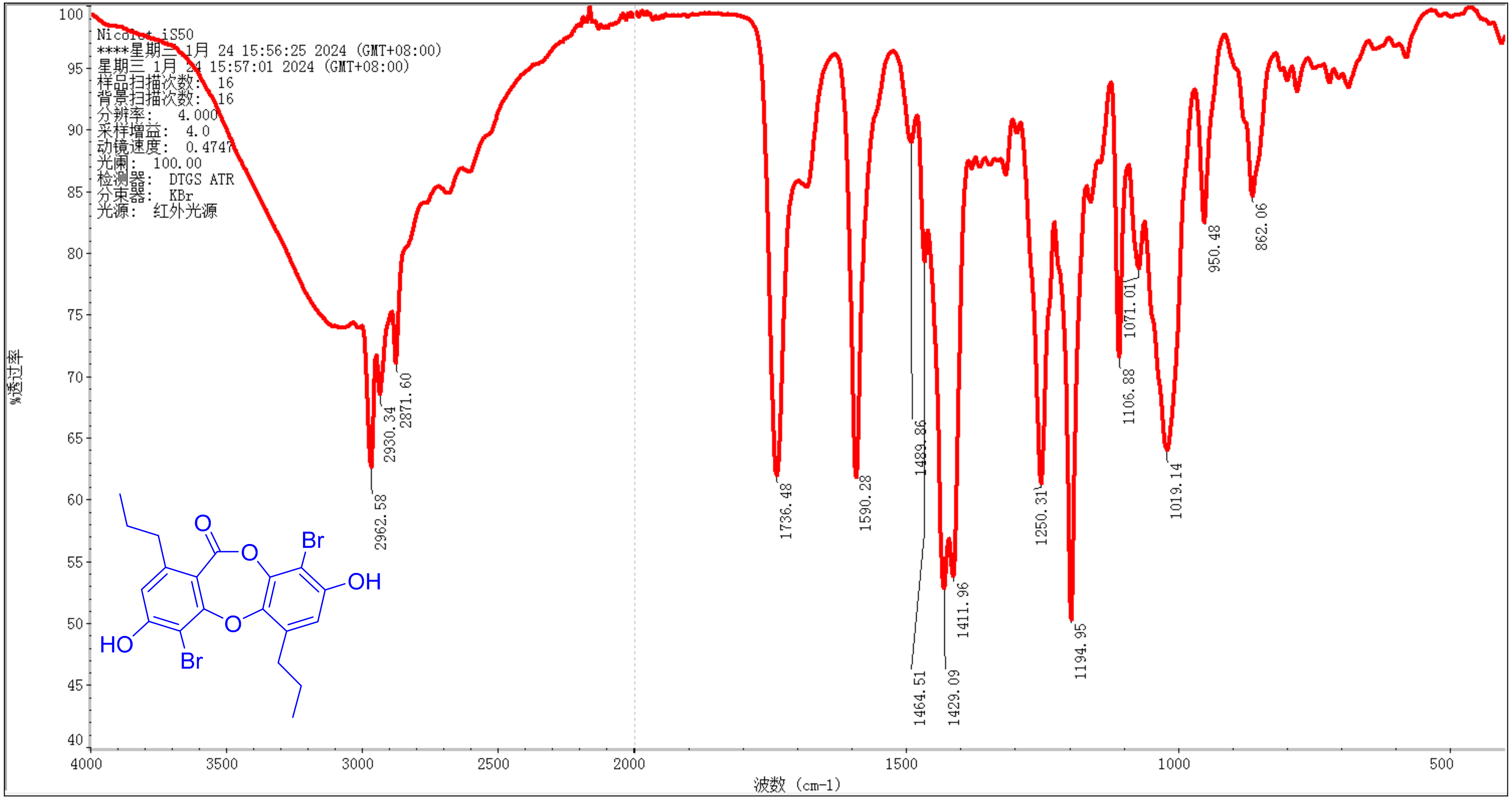

Figure S24. IR spectrum of **3**

Table S4. ^{1}H and ^{13}C NMR data and key COSY and HMBC correlations of 4

No.	δ_H	δ_C	COSY	HMBC
1		112.9, C		
2		161.7, C		
3	6.71, s	105.2, CH		C-1, C-2 C-4, C-5
4		161.7, C		
5		110.3, C		
6		146.9, C		
7		161.9, C		
8	2.86, t (7.7)	35.7, CH_2	H_2-9	C-1, C-5, C-6, C-9, C-10
9	1.54, m	22.9, CH_2	H_2-8, H3-10	C-6, C-8, C-10
10	0.86, t (7.3)	14.2, CH_3	H_2-9	C-8, C-9
1′		142.0, C		
2′		142.1, C		
3′		102.2, C		
4′		151.0, C		
5′		111.1, C		
6′		134.0, C		
7′	2.81, t (8.1)	32.6, CH_2	H_2-8’	C-1', C-5', C-6', C-8', C-9'
8′	1.51, m	22.5, CH_2	H_2-7’, H_3-9’	C-6', C-7', C-9'
9′	0.98, t (7.3)	14.9, CH_3	H_2-8’	C-7', C-8'

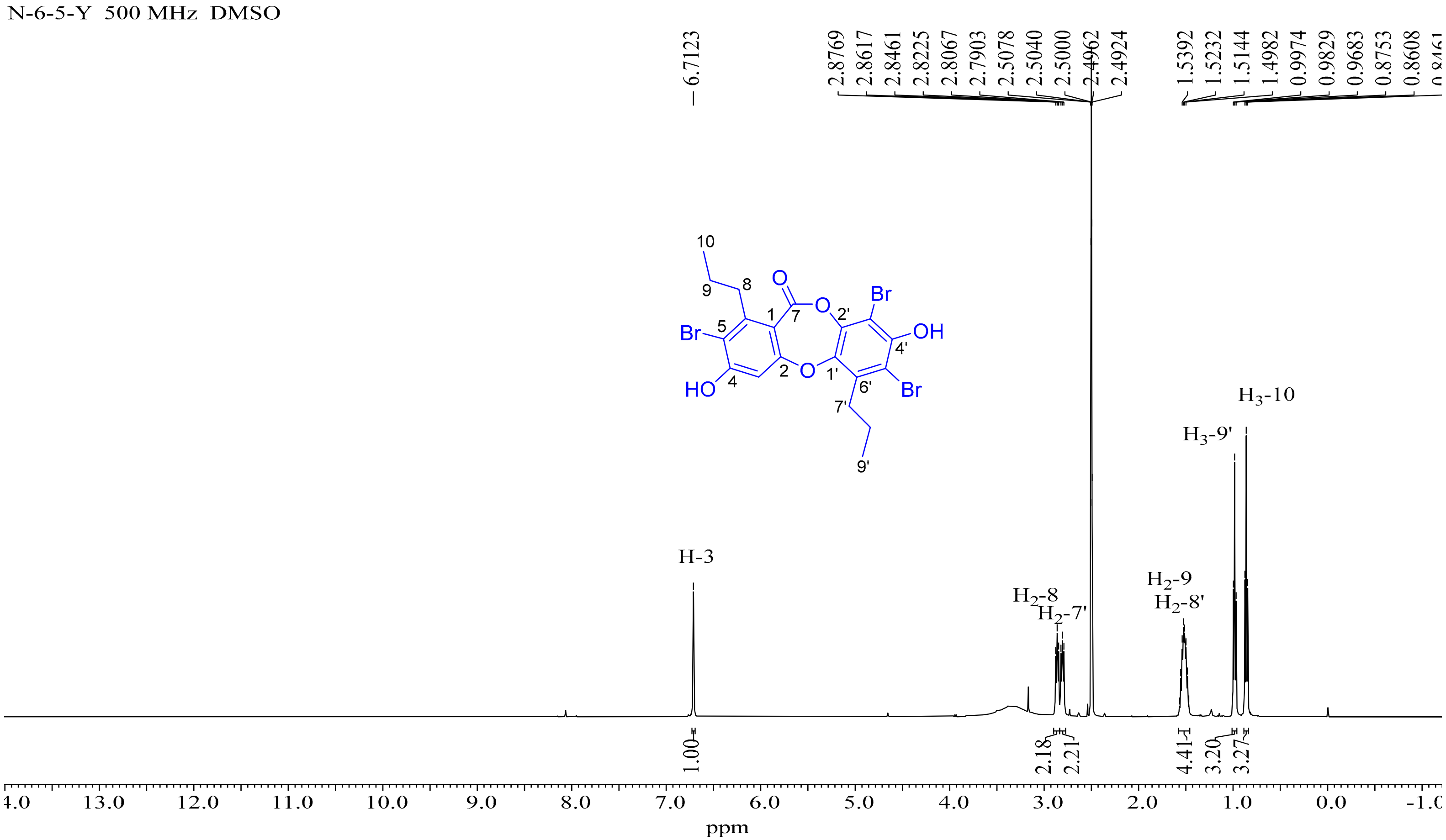

Figure S25. ¹H-NMR spectrum of **4** in DMSO-d_6 (500 MHz)

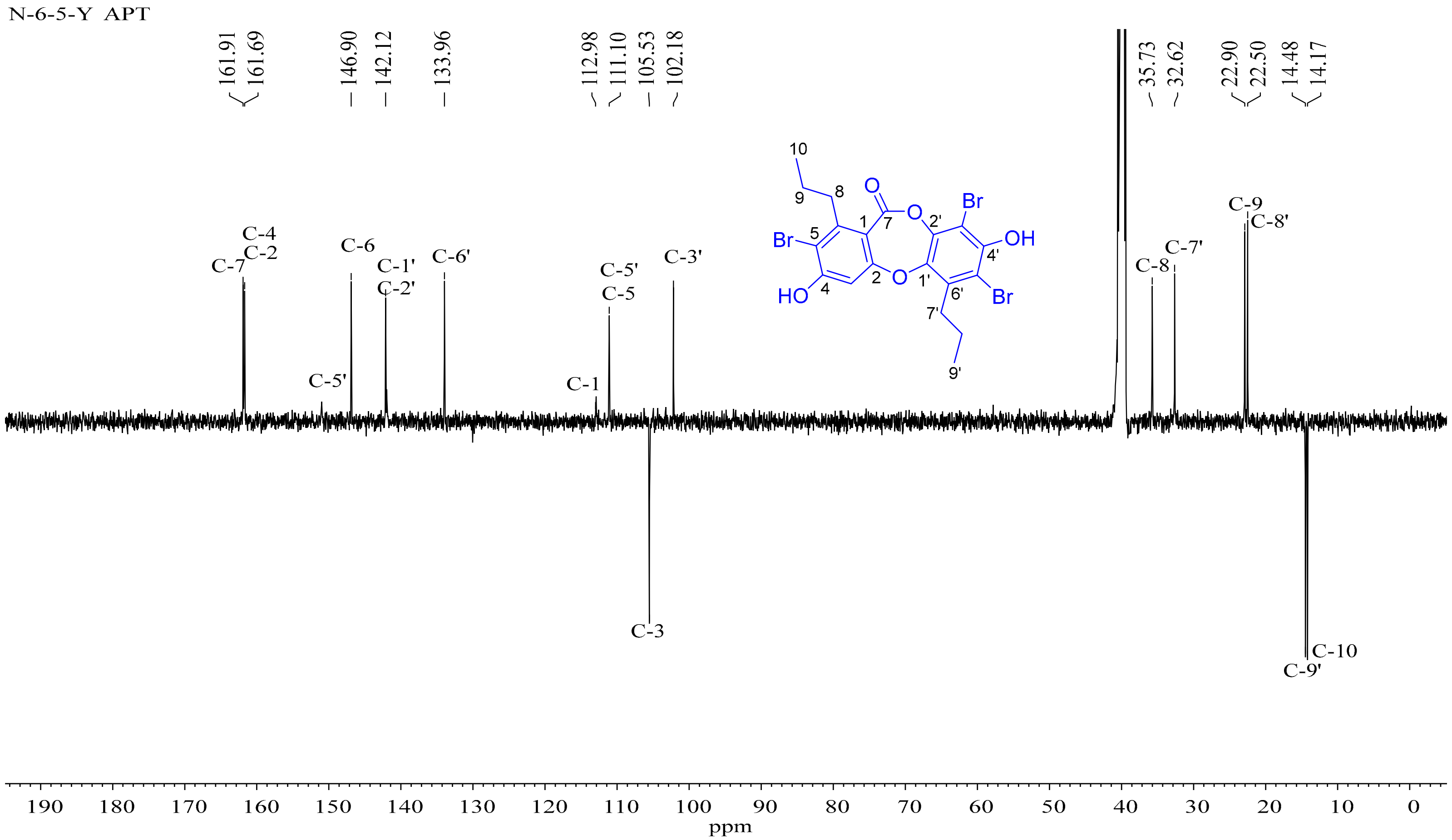

Figure S26. APT spectrum of **4** in DMSO-*d*6 (125 MHz)

N-6-5-Y HSQC

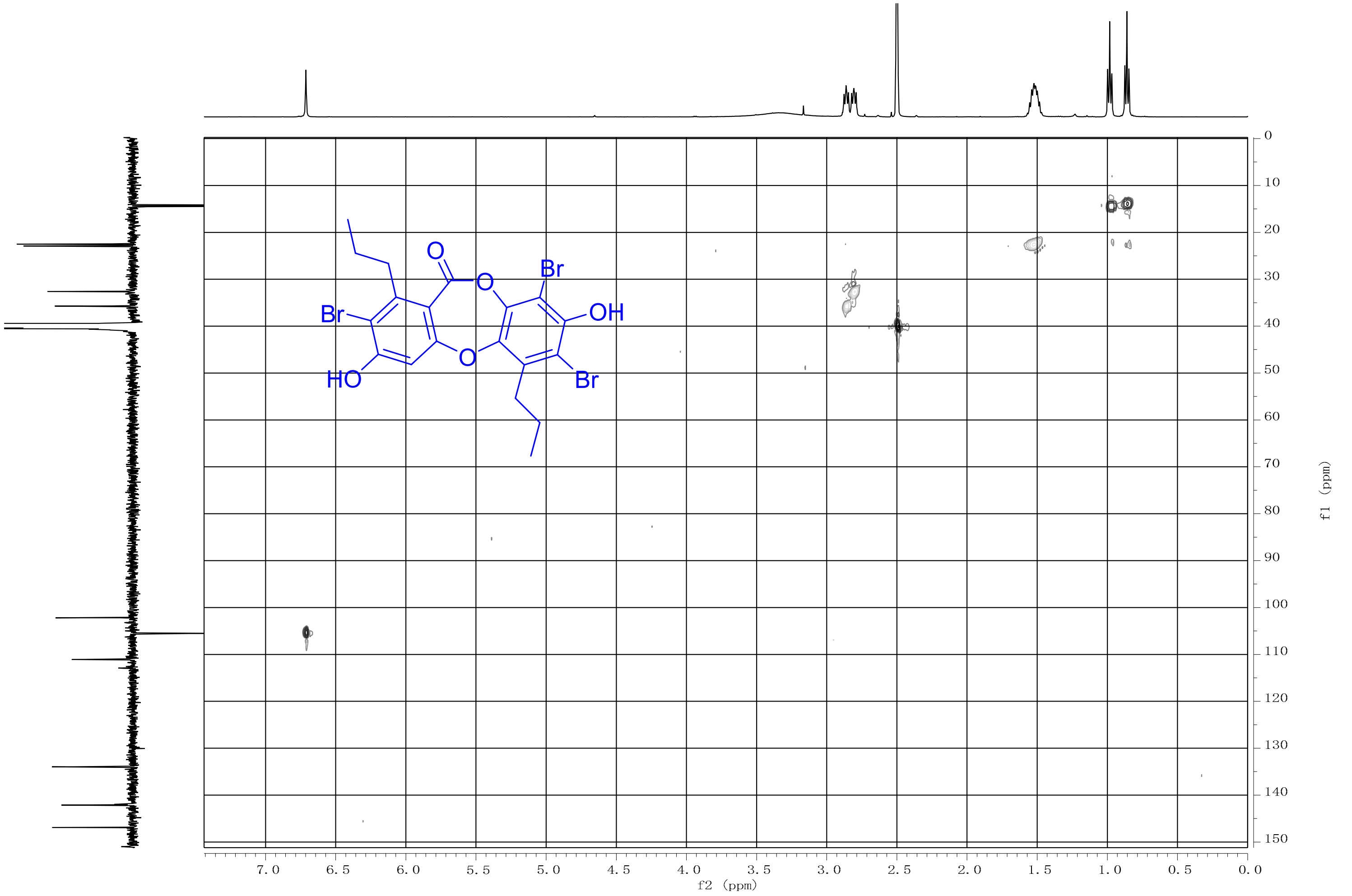

Figure S27. HSQC spectrum of **4** in DMSO-d_6

N-6-5-Y COSY

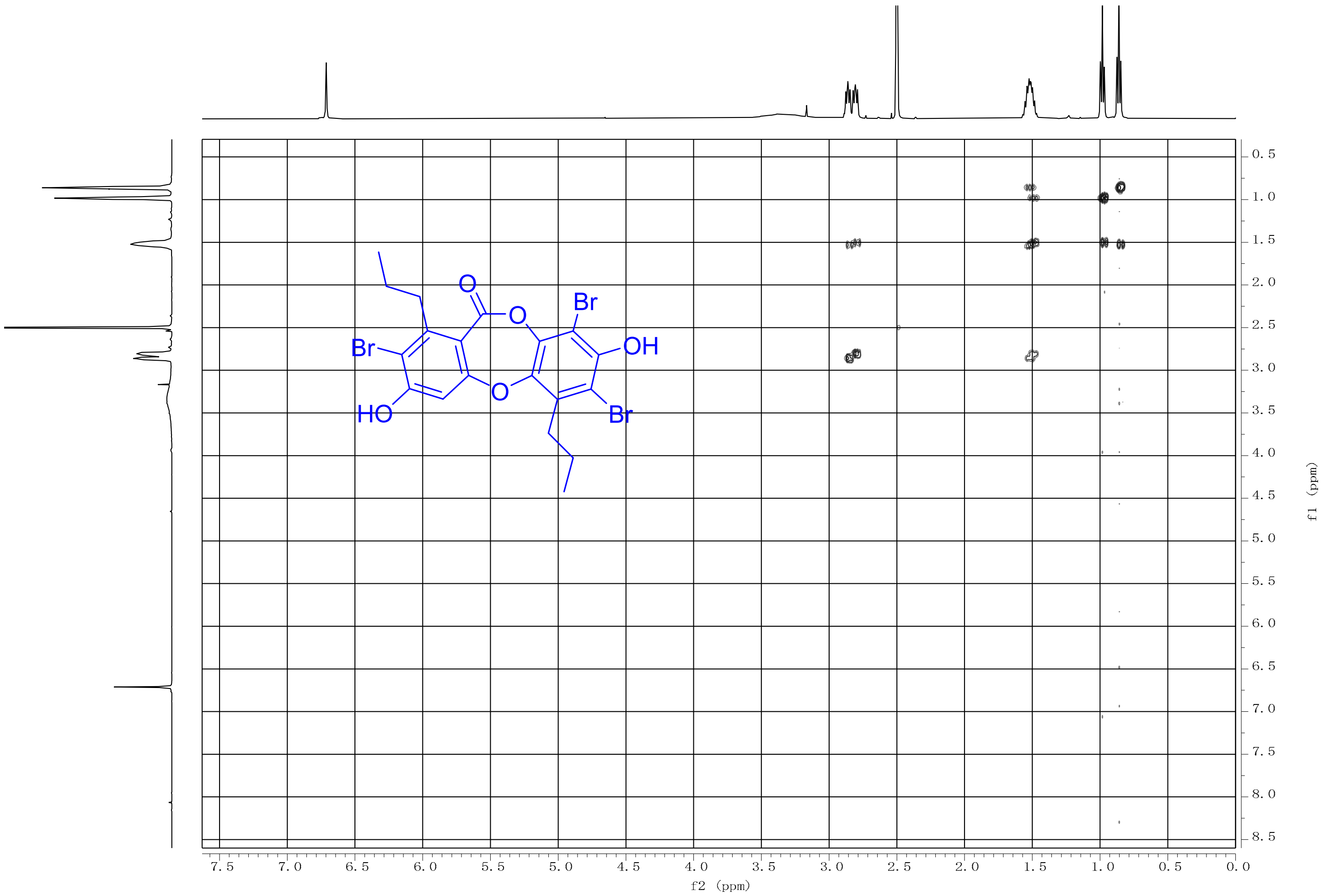

Figure S28. ^1H-^1H COSY spectrum of **4** in DMSO-d_6

N-6-5-Y HMBC

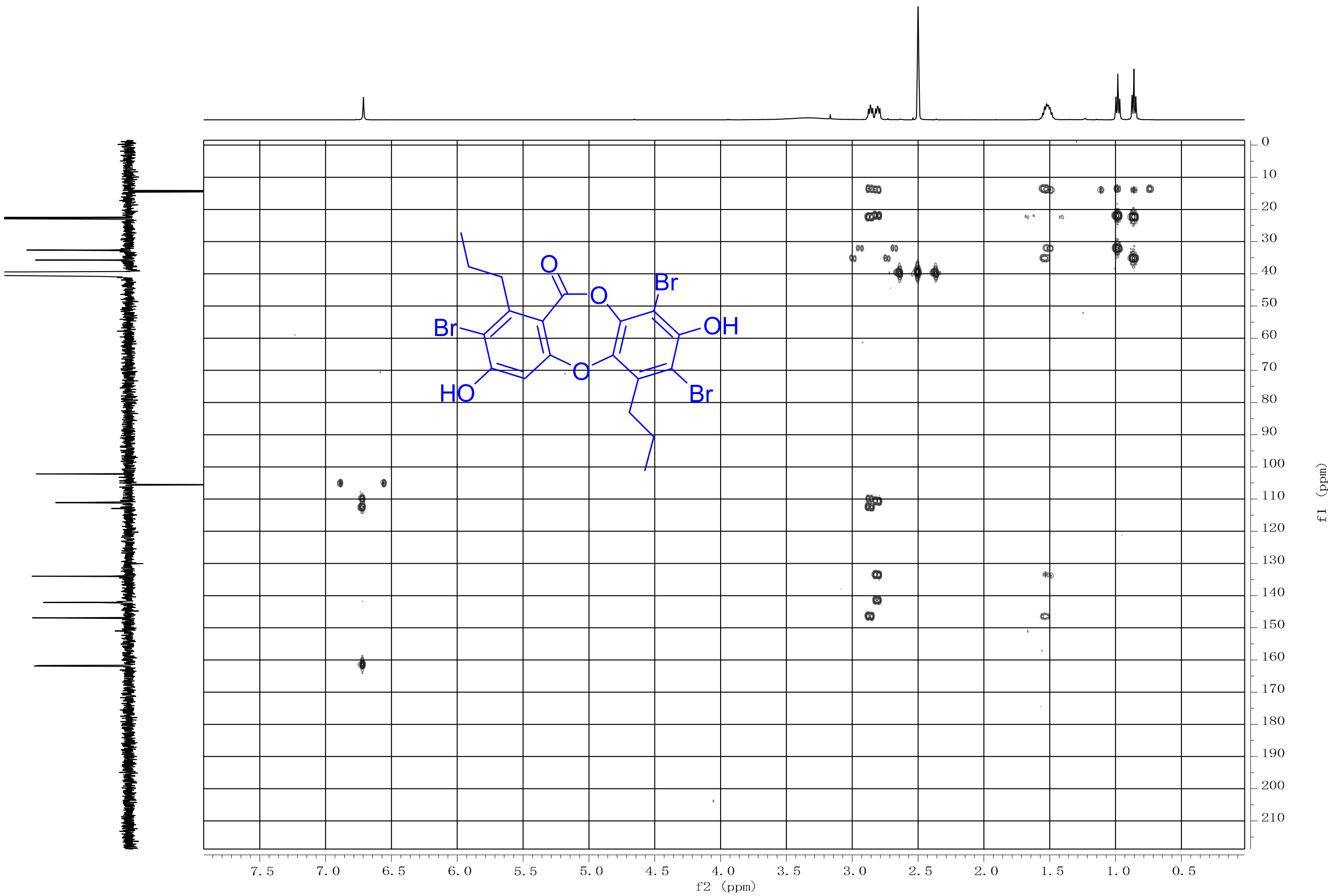

Figure S29. HMBC spectrum of **4** in DMSO-d_6

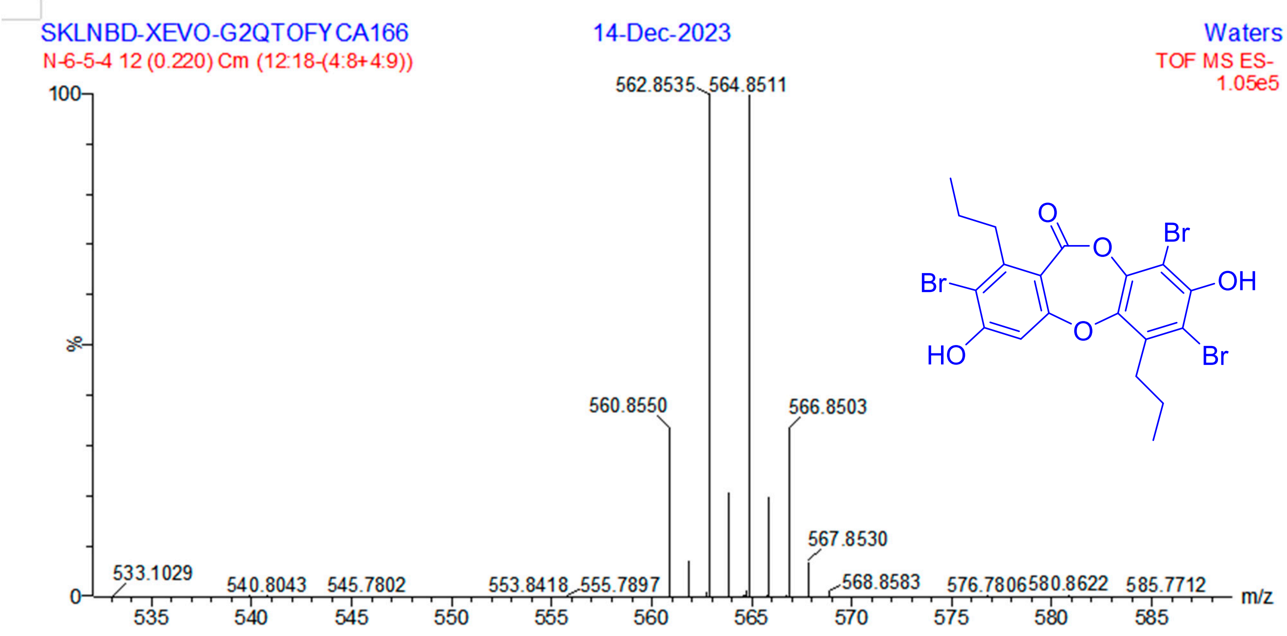

Mass	Calc. Mass	mDa	PPM	DBE	i-FIT	Norm	Conf(%)	Formula
560.8550	560.8551	-0.1	-0.2	30.5	253.8	14.560	0.00	$C_{32}H_3OBr_2$
	560.8548	0.2	0.4	10.5	239.2	0.000	100.00	$C_{19}H_{16}O_5Br_3$
	560.8574	-2.4	-4.3	-0.5	254.7	15.479	0.00	$C_7H_{15}O_{19}Br_2$
	560.8577	-2.7	-4.8	19.5	258.7	19.441	0.00	$C_{20}H_2O_{15}Br$

Figure S30. HRESIMS spectrum of **4**

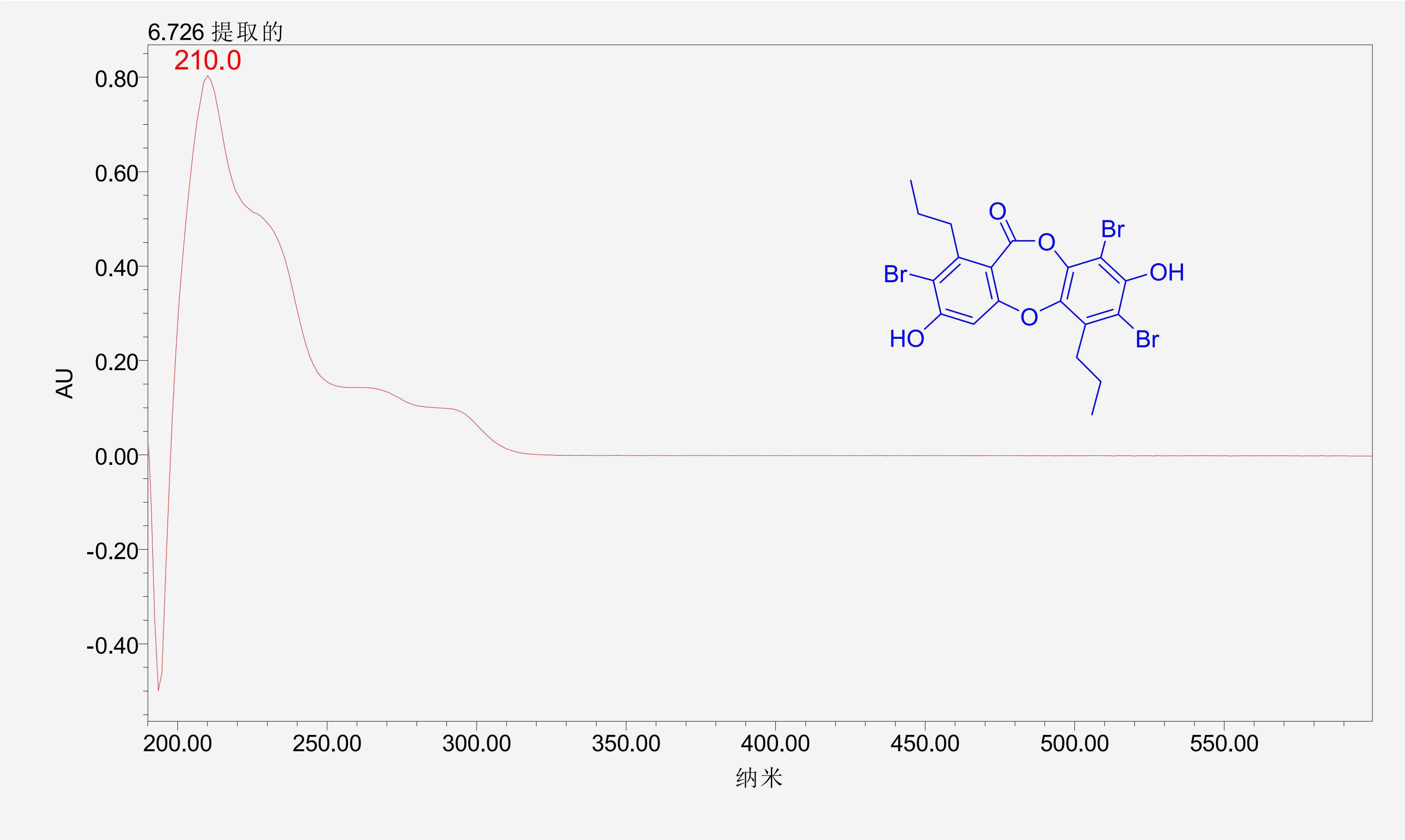

Figure S31. UV spectrum of **4**

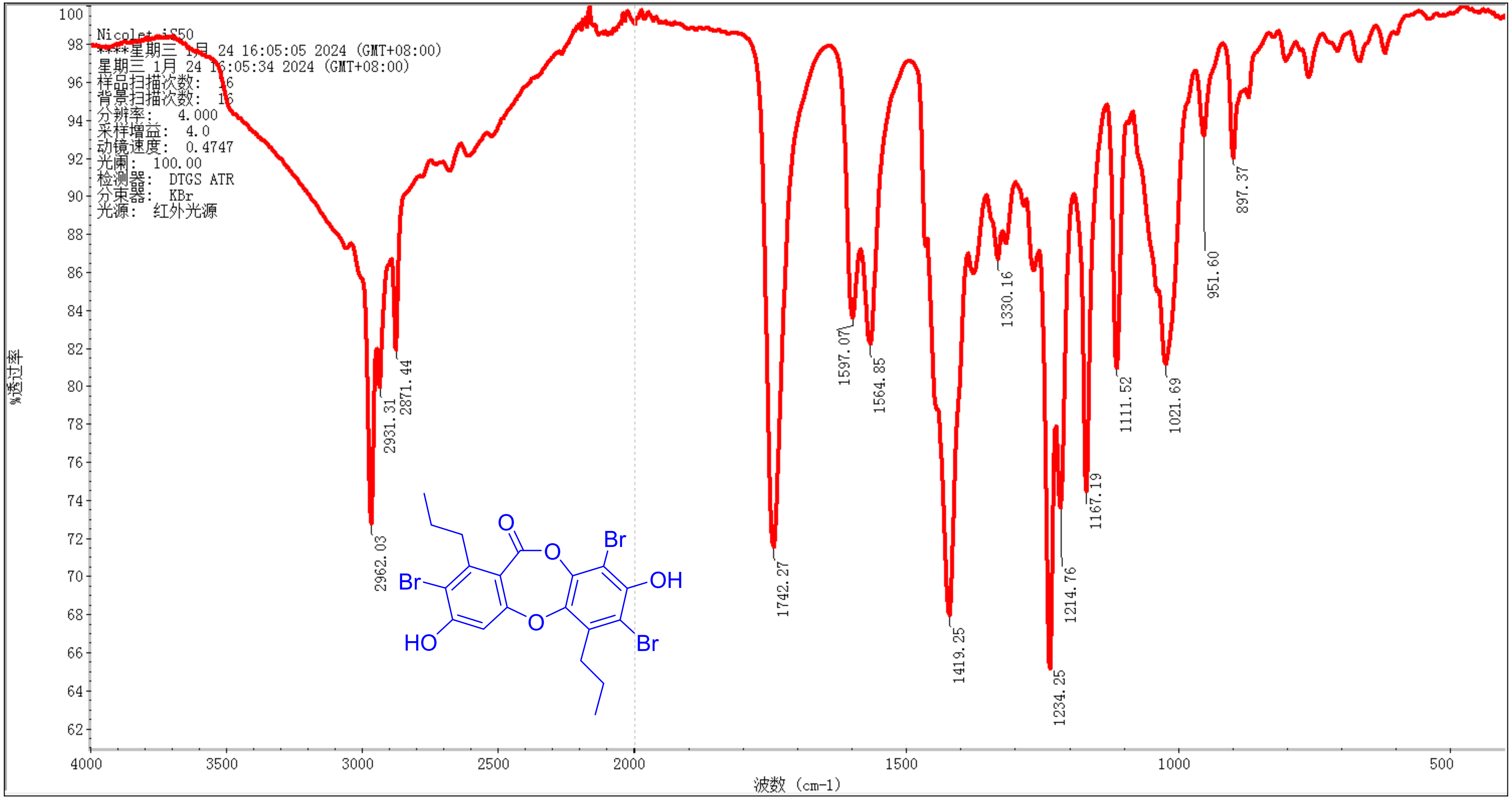

Figure S32. IR spectrum of **4**

Table S5. ^1H and ^{13}C NMR data and key COSY and HMBC correlations of 5

No.	δ_H	δ_C	COSY	HMBC
1		113.6, C		
2		159.3, C		
3		101.3, C		
4		159.3, C		
5		112.8, C		
6		145.8, C		
7		162.0, C		
8	3.11, t (8.0)	36.9, CH_2		C-1, C-5, C-6, C-9, C-10
9	1.59, m	22.9, CH_2		C-6, C-8, C-10
10	0.88, t (7.3)	14.3, CH_3		C-8, C-9
1′		142.0, C		
2′		143.4, C		
3′	6.80, s	105.6, C		C-1, C-2, C-4, C-5
4′		153.0, C		
5′		108.9, C		
6′		135.9, C		
7′	2.76, t (7.8)	33.0, CH_2		C-1', C-5', C-6', C-8', C-9'
8′	1.48, m	23.1, CH_2		C-6', C-7', C-9'
9′	1.01, t (7.3)	14.4, CH_3		C-7', C-8'

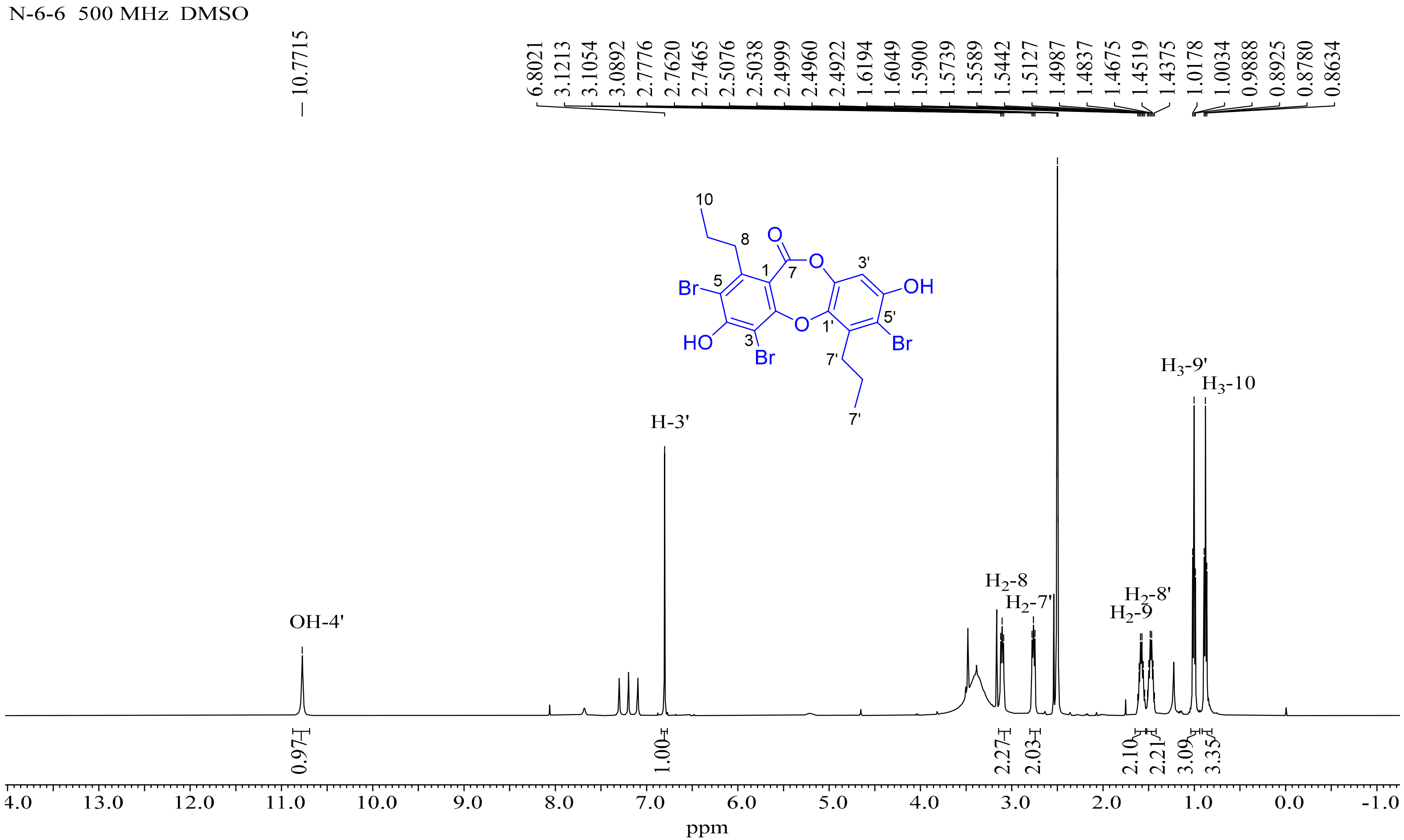

Figure S33. ^{1}H-NMR spectrum of **5** in DMSO-*d*6 (500 MHz)

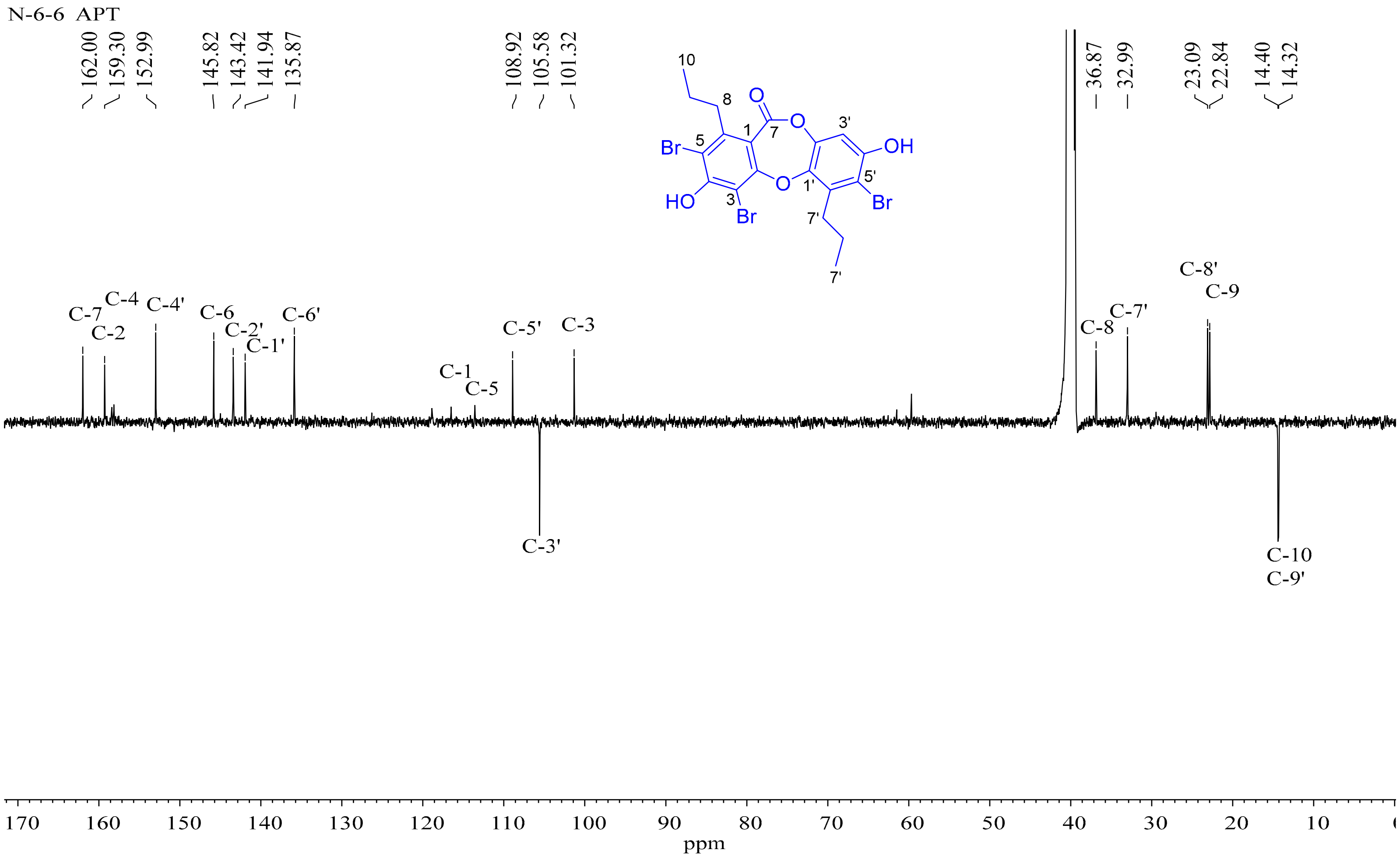

Figure S34. APT spectrum of **5** in DMSO-d_6 (125 MHz)

N-6-6 HSQC

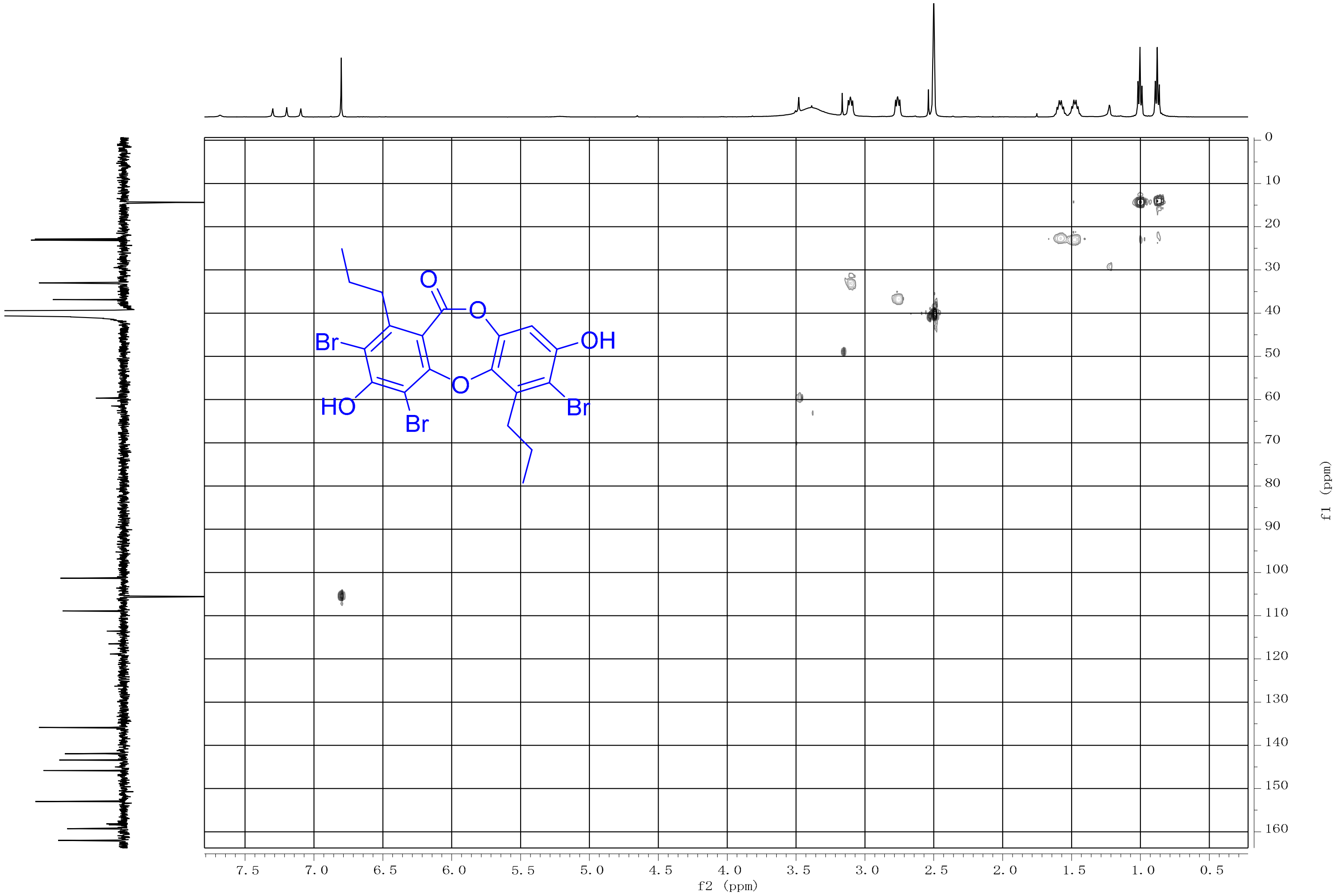

Figure S35. HSQC spectrum of **5** in DMSO-d_6

N-6-6 COSY

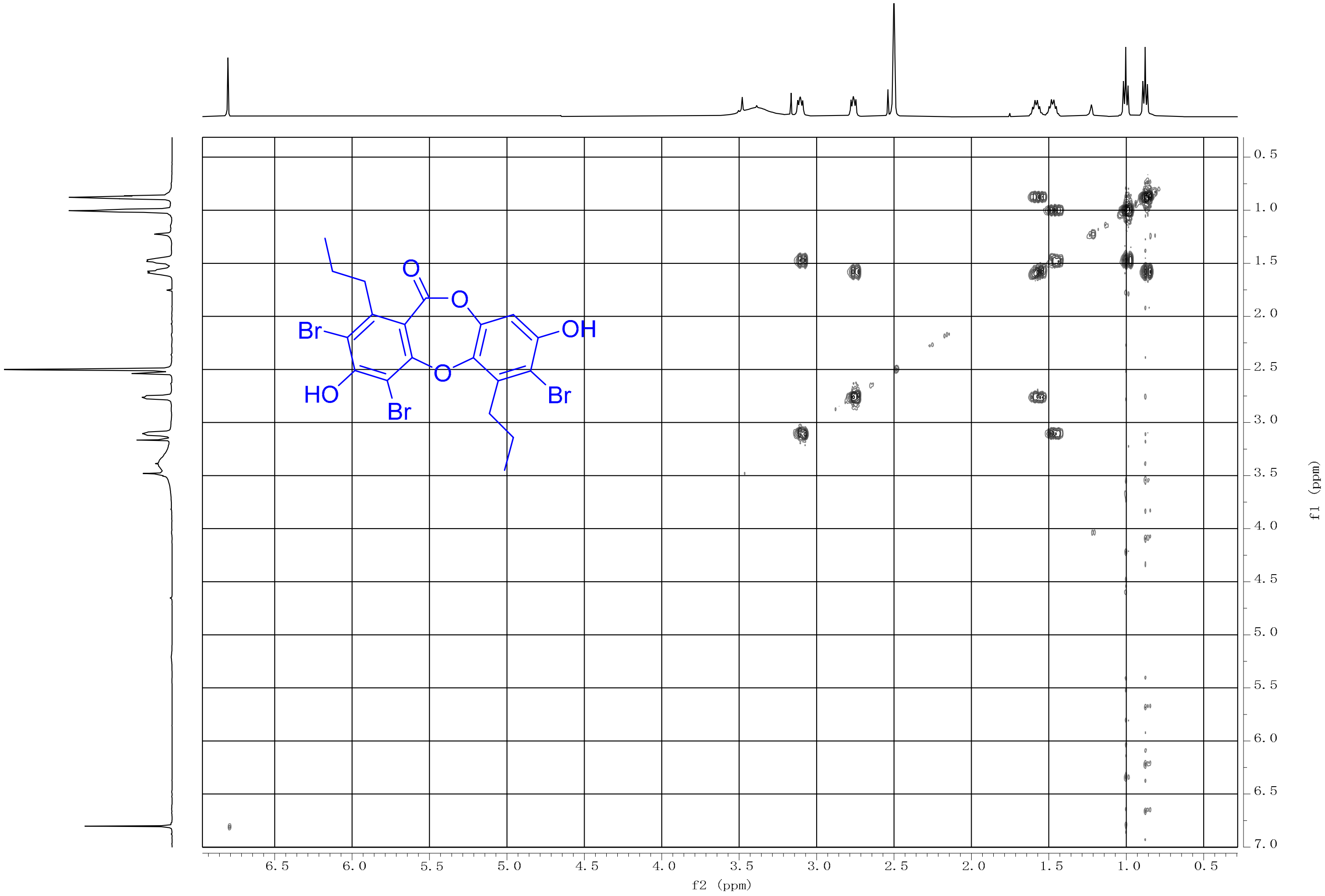

Figure S36. ^{1}H-^{1}H COSY spectrum of **5** in DMSO-d_6

N-6-6 HMBC

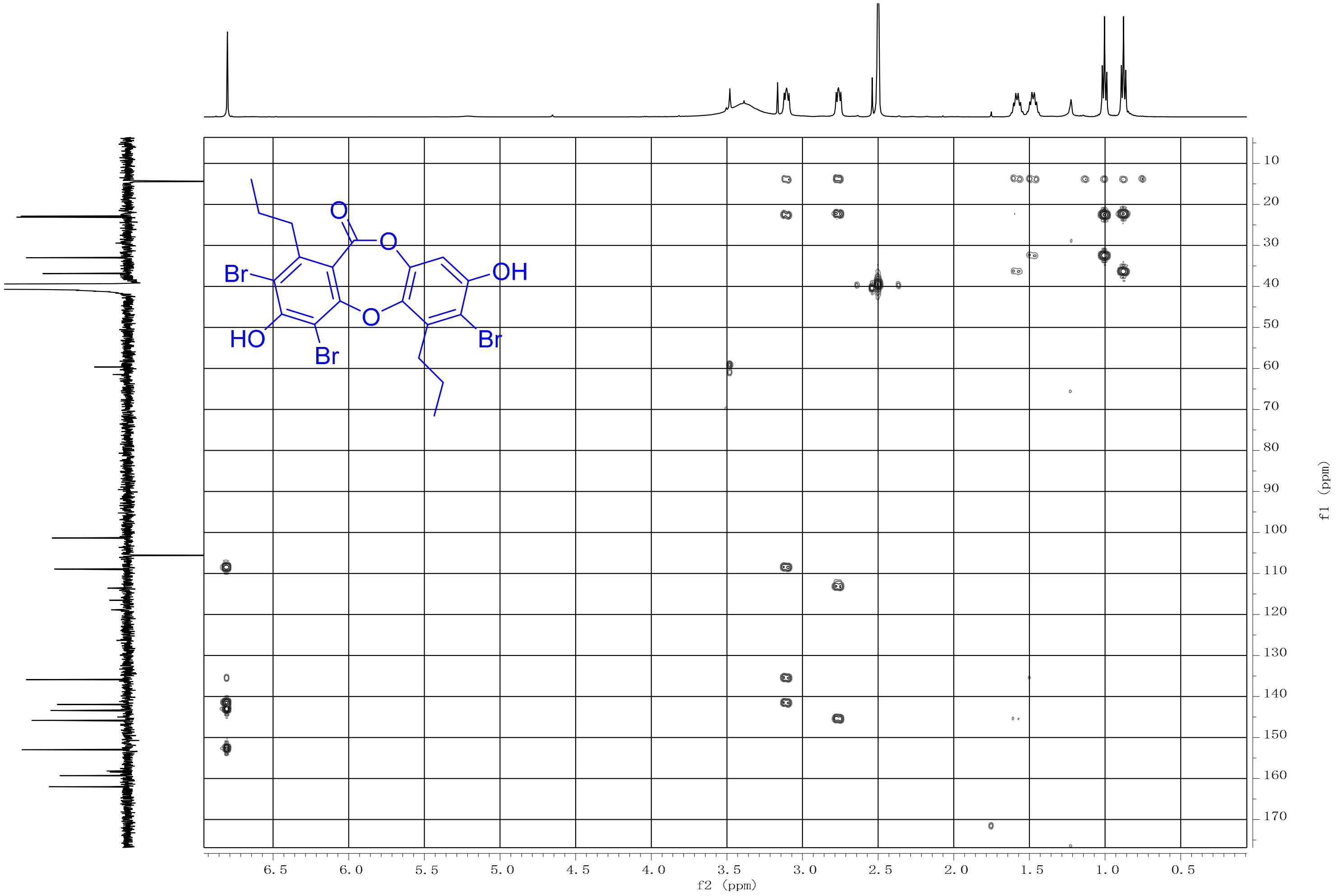

Figure S37. HMBC spectrum of **5** in DMSO-d_6

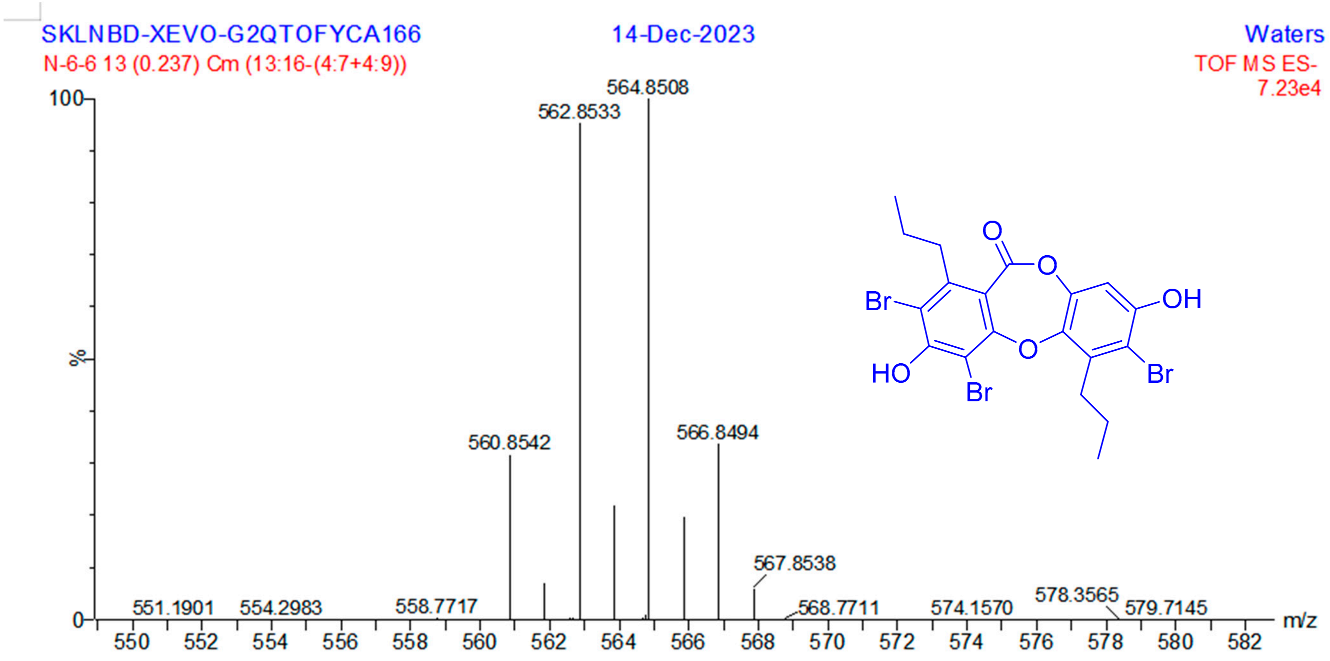

Mass	Calc. Mass	mDa	PPM	DBE	i-FIT	Norm	Conf(%)	Formula
560.8542	560.8548	-0.6	-1.1	10.5	216.0	0.000	100.00	$C_{19}H_{16}O_5Br_3$
	560.8551	-0.9	-1.6	30.5	229.1	13.105	0.00	$C_{32}H_3OBr_2$
	560.8516	2.6	4.6	8.5	229.9	13.979	0.00	$C_{14}H_{11}O_{14}Br_2$

Figure S38. HRESIMS spectrum of **5**

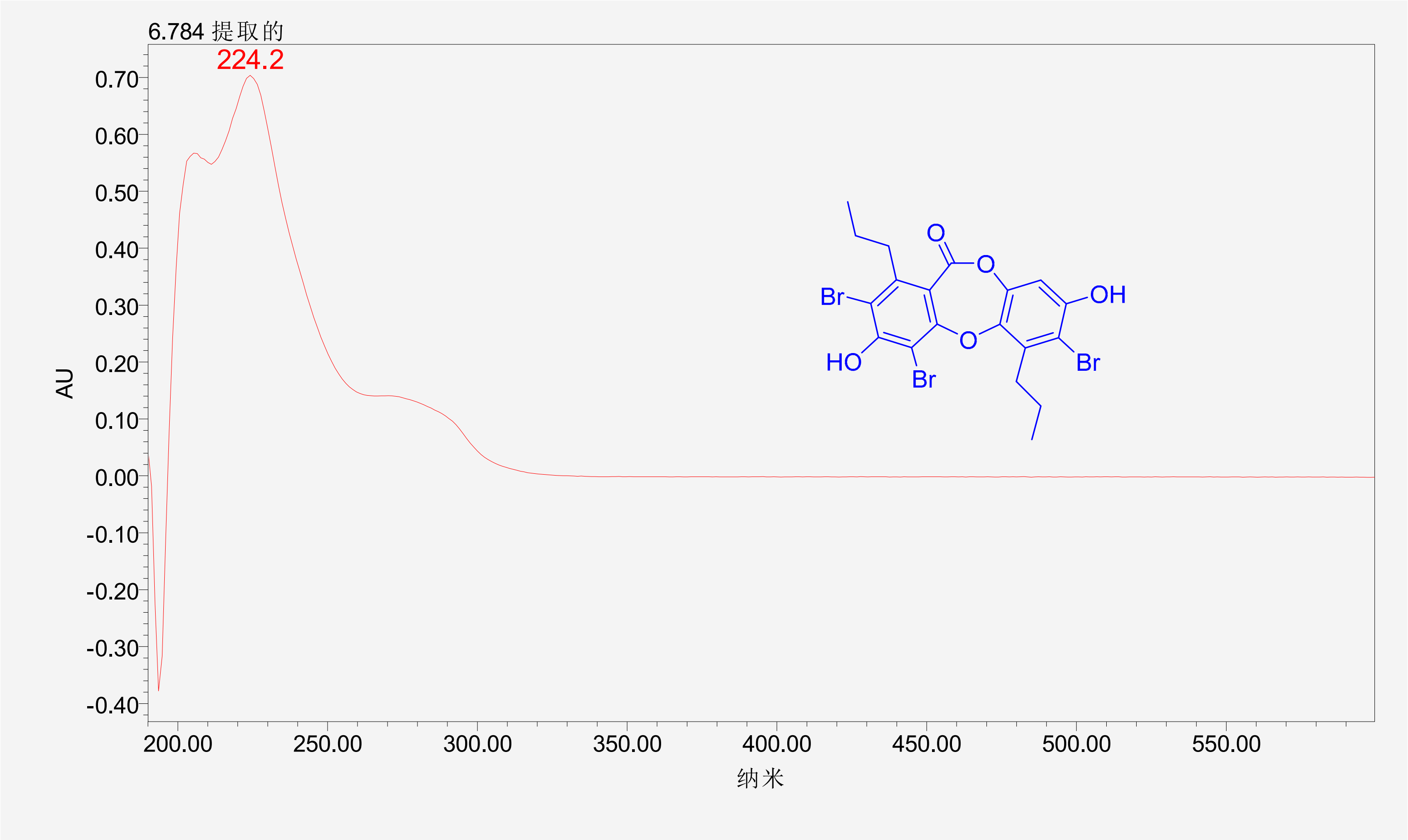

Figure S39. UV spectrum of **5**

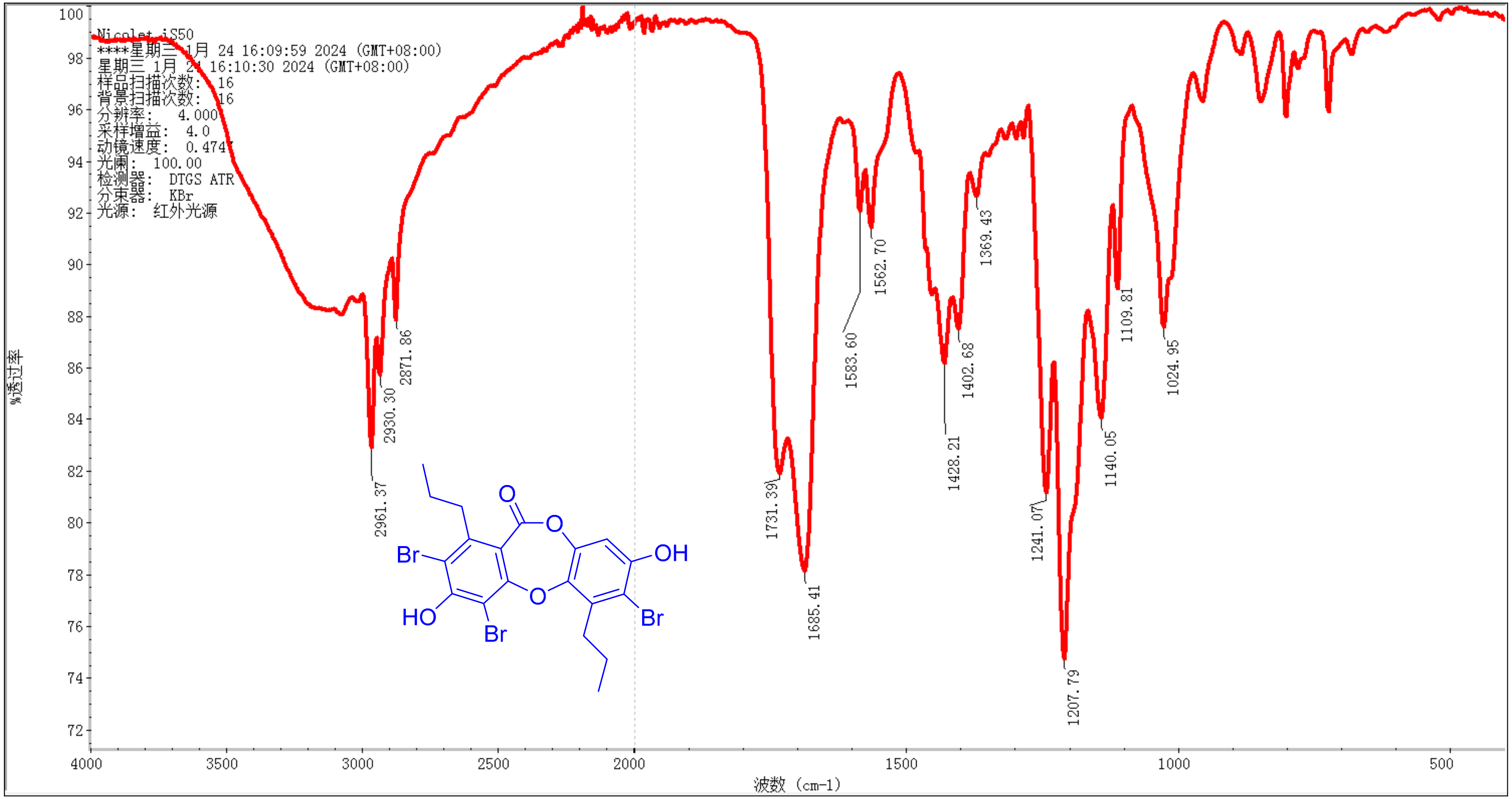

Figure S40. IR spectrum of **5**

Table S6. ^{1}H and ^{13}C NMR data and key COSY and HMBC correlations of 6

No.	δ_H	δ_C	COSY	HMBC
1		113.2, C		
2		159.2, C		
3		101.3, C		
4		159.2, C		
5		114.1, C		
6		146.1, C		
7		161.7, C		
8	2.78, t (7.9)	27.1, CH_2		C-1, C-5, C-6, C-9, C-10
9	1.47, m	23.1, CH_2		C-6, C-8, C-10
10	0.90, t (7.3)	14.4, CH_3		C-8, C-9
1′		143.0, C		
2′		143.7, C		
3′	7.12, s	103.5, CH		C-1, C-2, C-4, C-5
4′		154.4, C		
5′		110.0, C		
6′		136.1, C		
7′	3.14, t (7.5)	33.0, CH_2		C-1', C-5', C-6', C-8', C-9'
8′	1.61, m	22.9, CH_2		C-6', C-7', C-9'
9′	1.01, t (7.3)	14.4, CH_3		C-7', C-8'
MeO	3.84, s	57.6, CH_3		C-4’

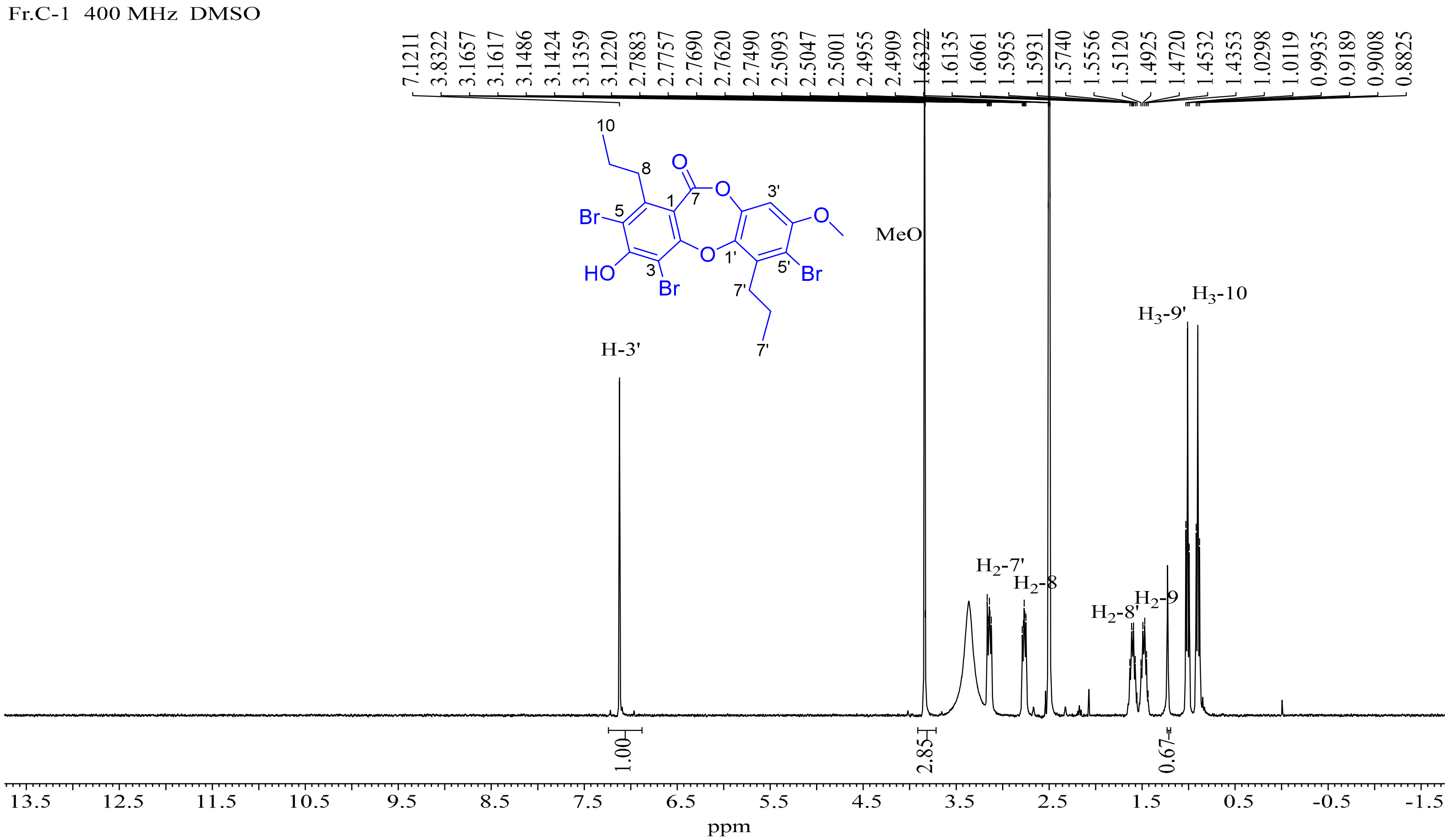

Figure S41. ^{1}H-NMR spectrum of **6** in DMSO-d_6 (400 MHz)

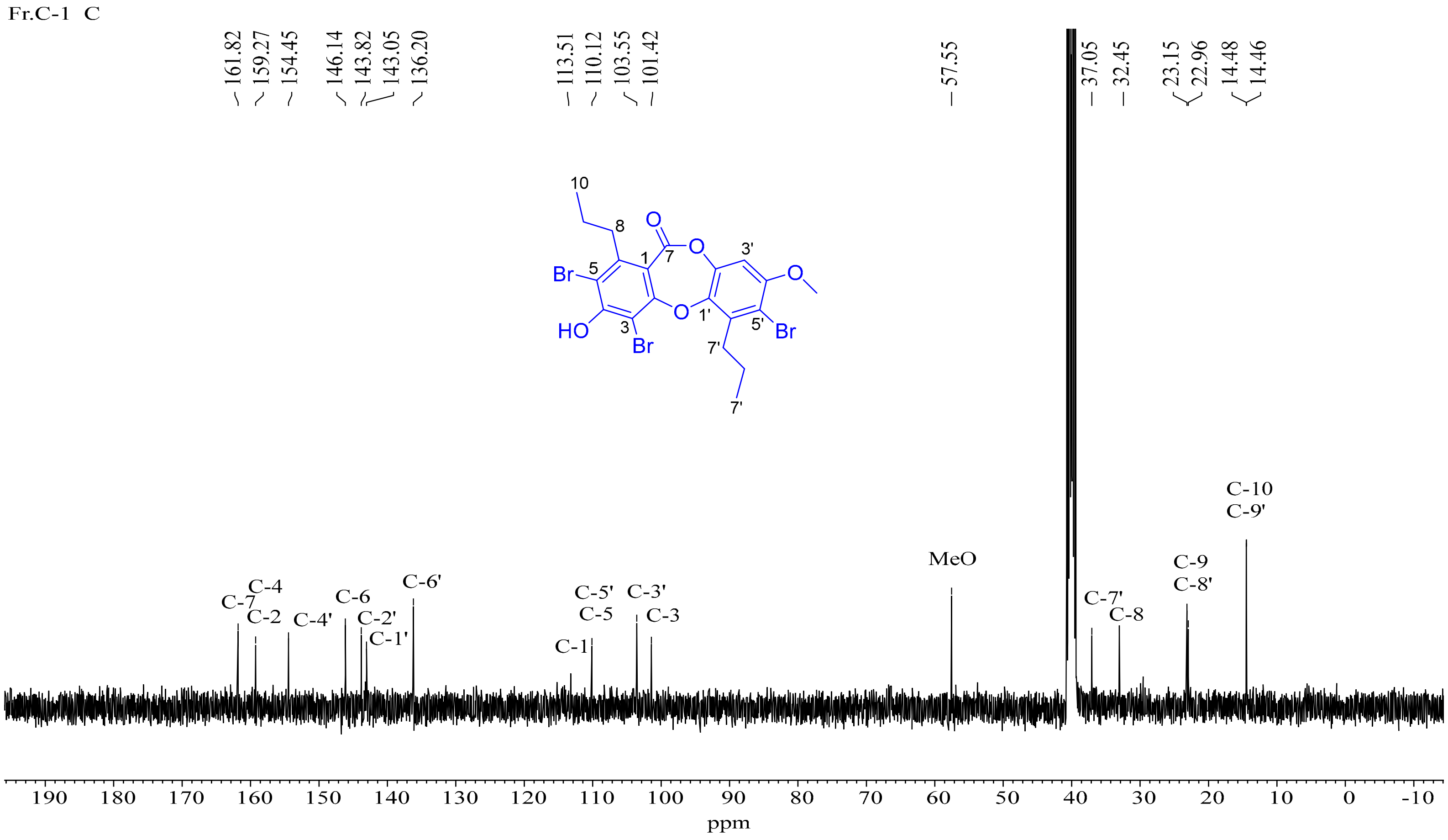

Figure S42. ^{13}C-NMR spectrum of **6** in DMSO-d_6 (100 MHz)

Fr.C-1 HSQC

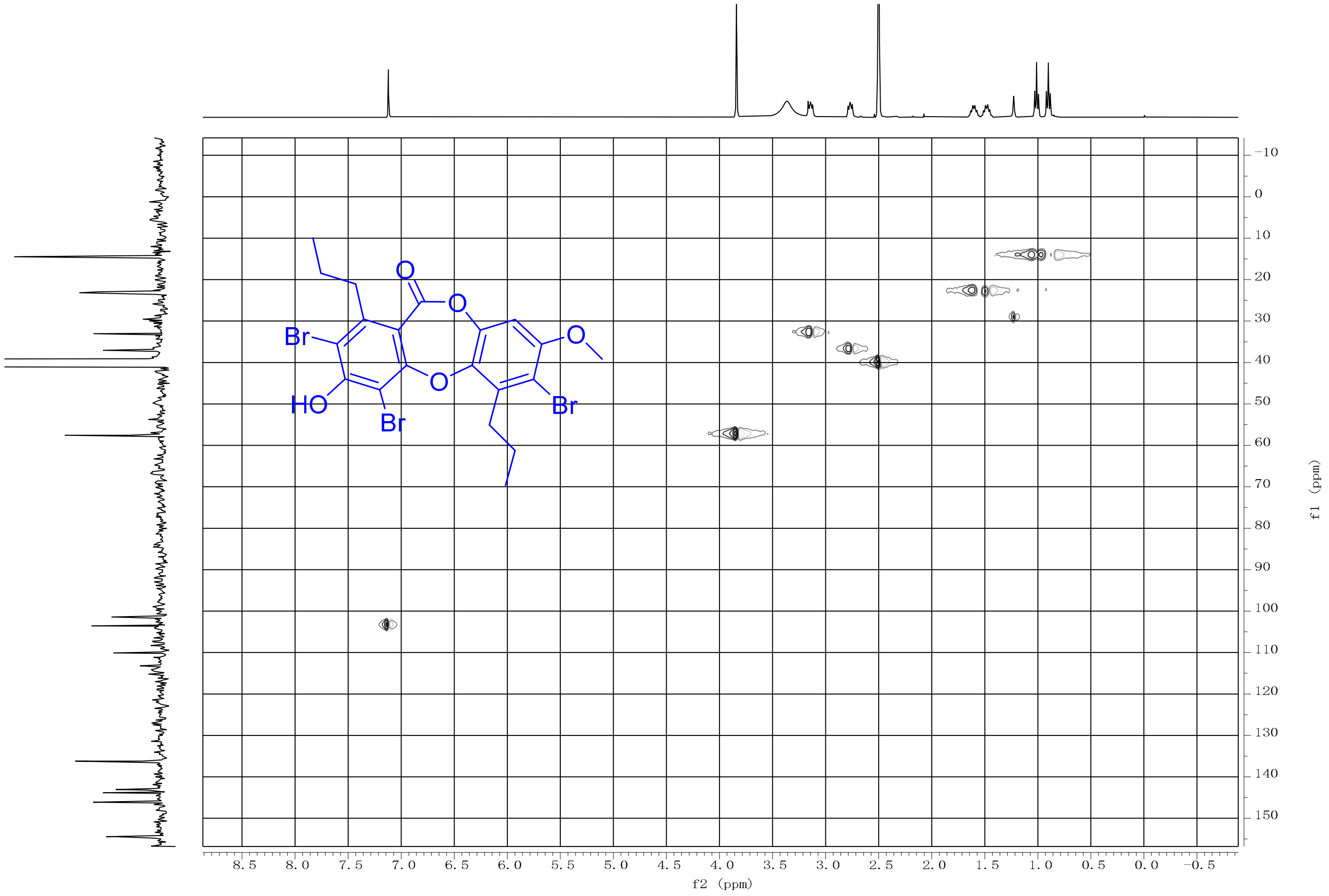

Figure S43. HSQC spectrum of **6** in DMSO-d_6

Fr.C-1 COSY

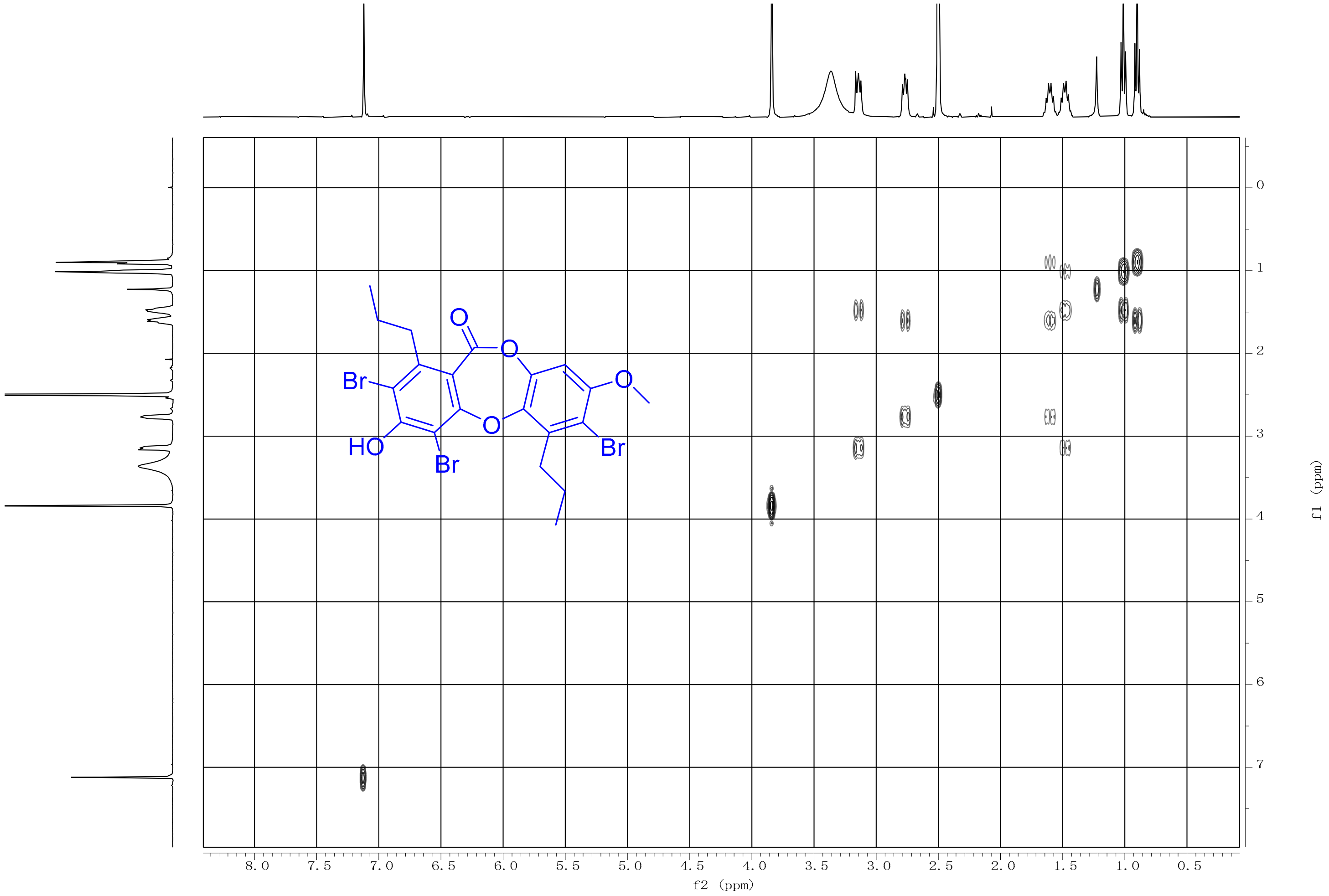

Figure S44. ^{1}H-^{1}H COSY spectrum of **6** in DMSO-d_6

Fr.C-1 HMBC

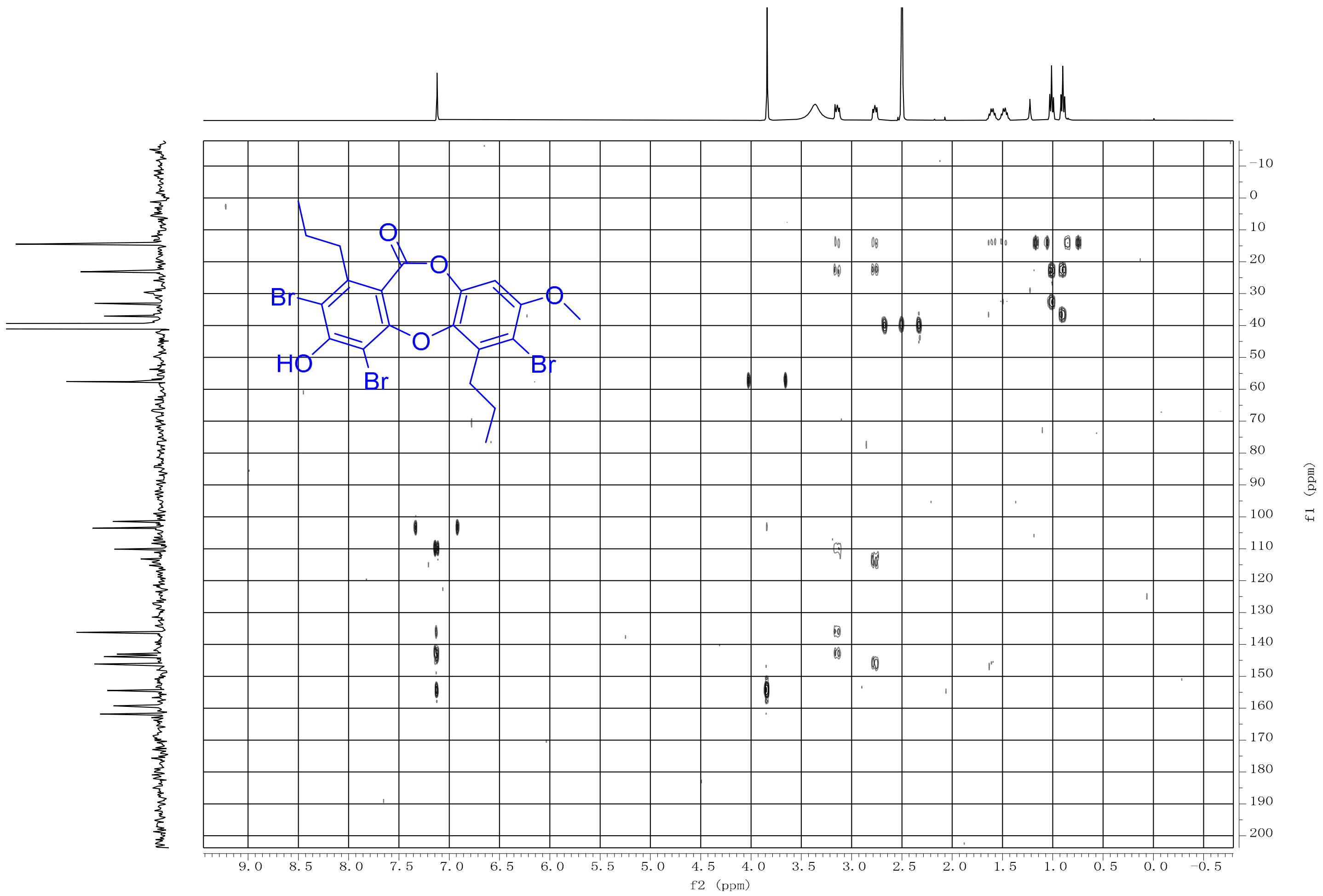

Figure S45. HMBC spectrum of **6** in DMSO-d_6

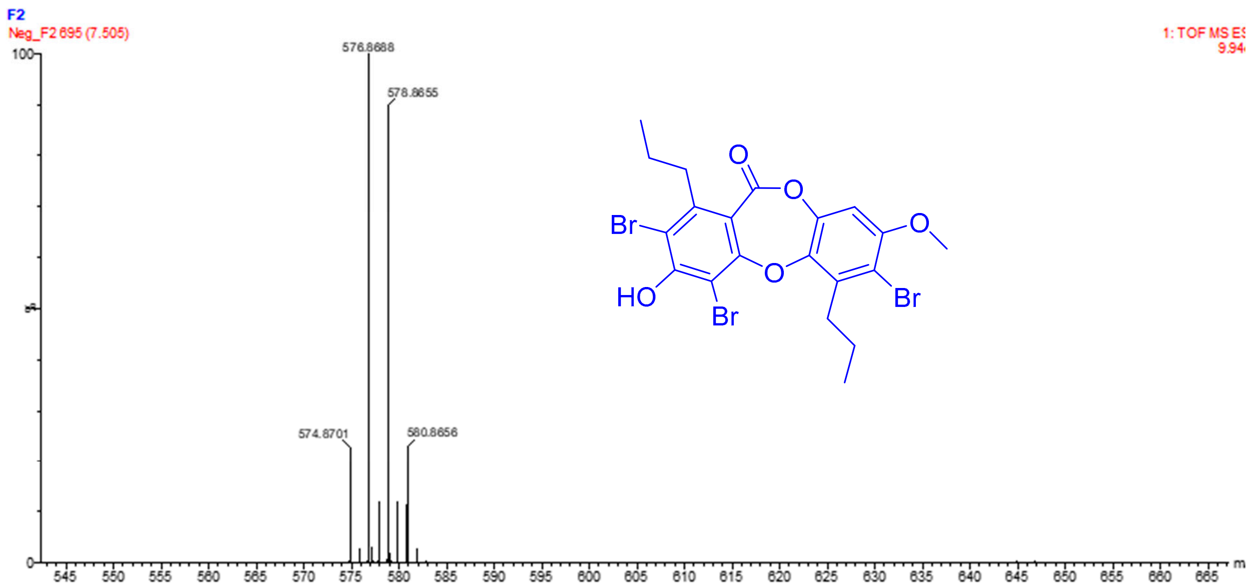

Single Mass Analysis

Tolerance = 5.0 mDa / DBE: min = -1.5, max = 50.0

Element prediction: Off

Monoisotopic Mass, Even Electron Ions

510 formula(e) evaluated with 6 results within limits (up to 50 closest results for each mass)

Elements Used:

C: 0-500 H: 0-1000 O: 0-200 Br: 0-8

Mass	Calc. Mass	mDa	PPM	DBE	Formula	C	H	O	Br
574.8701	574.8704	-0.3	-0.5	10.5	C20 H18 O5 Br3	20	18	5	3
	[illegible]	[illegible]	[illegible]	[illegible]	[illegible]	[illegible]	[illegible]	[illegible]	[illegible]

Figure S46. HRESIMS spectrum of **6**

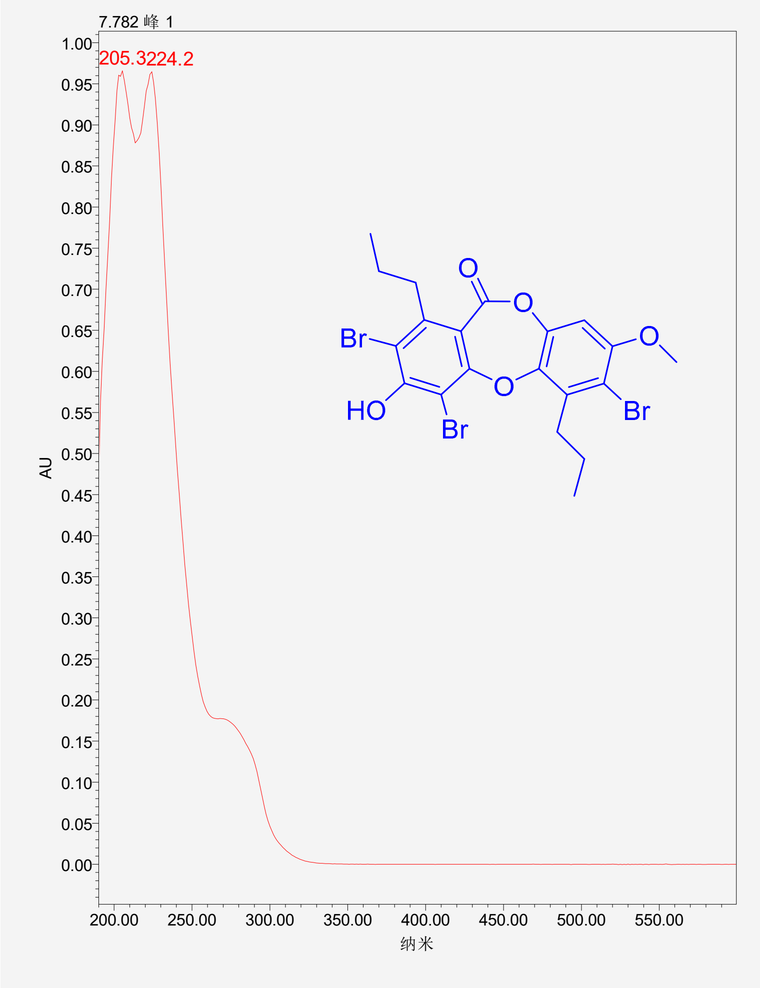

Figure S47. UV spectrum of **6**

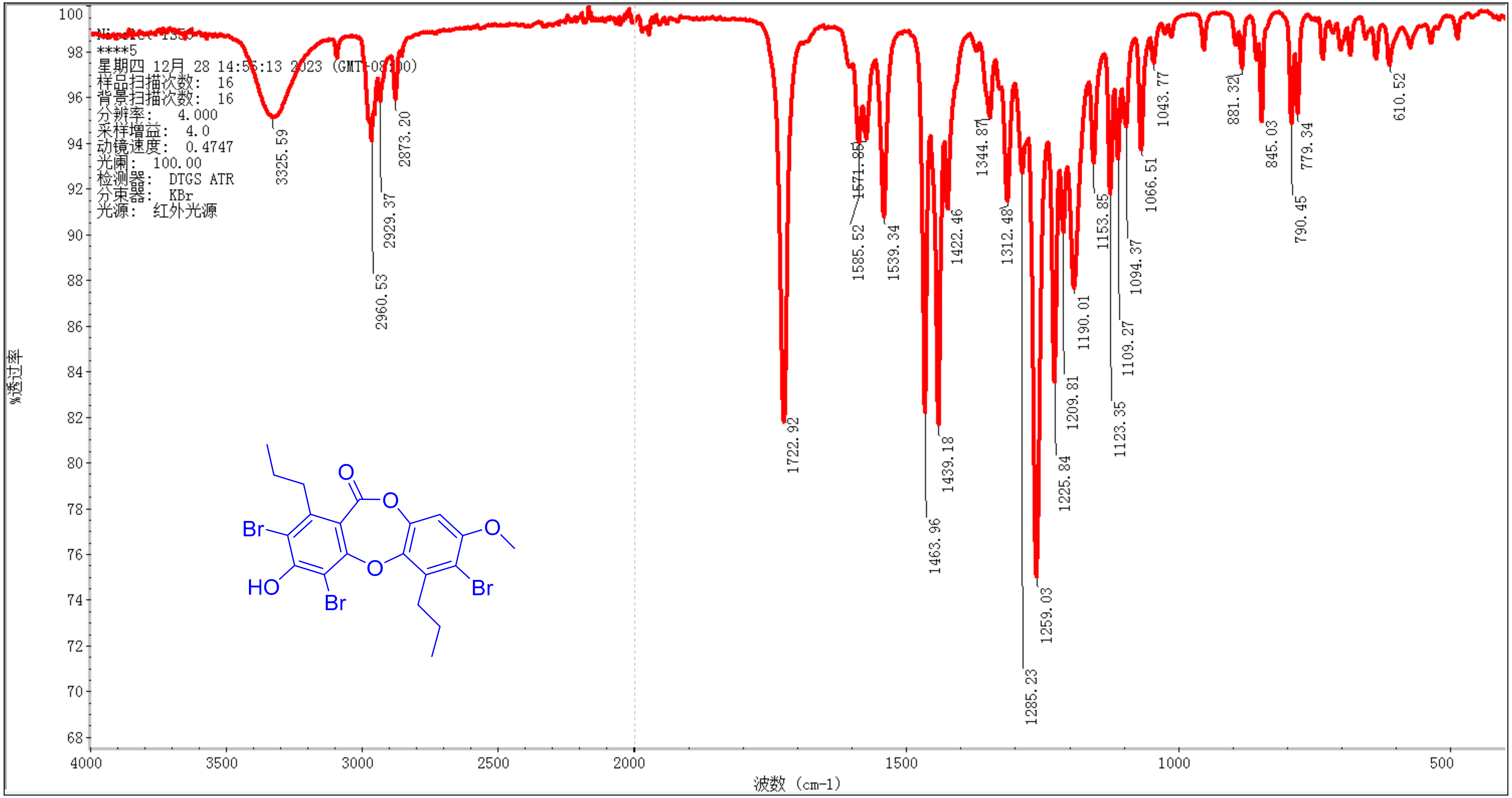

Figure S48. IR spectrum of **6**

Table S7. ^{1}H and ^{13}C NMR data and key COSY and HMBC correlations of 7

No.	δ_H	δ_C	COSY	HMBC
1		111.5, C		
2		160.2, C		
3		99.2, C		
4		160.0, C		
5	6.84, s	115.6, C		C-1, C-3, C-4, C-8
6		148.8, C		
7		160.7, C		
8	2.68, t (7.7)	35.7, CH_2	H_2-9	C-1, C-5, C-6, C-9, C-10
9	1.48, m	24.5, CH_2	H_2-8, H3-10	C-6, C-8, C-10
10	0.83, t (7.3)	14.2, CH_3	H_2-9	C-8, C-9
1′		146.7, C		
2′		142.2, C		
3′		108.3, C		
4′		152.8, C		
5′		117.2, C		
6′		135.5, C		
7′	3.16, t (8.0)	33.2, CH_2	H_2-8’	C-1', C-5', C-6', C-8', C-9'
8′	1.50, m	22.8, CH_2	H_2-7’, H3-9’	C-6', C-7', C-9'
9′	1.00, t (7.2)	14.3, CH_3	H_2-8’	C-7', C-8'
MeO	3.78, s	60.9, CH_3		C-4’

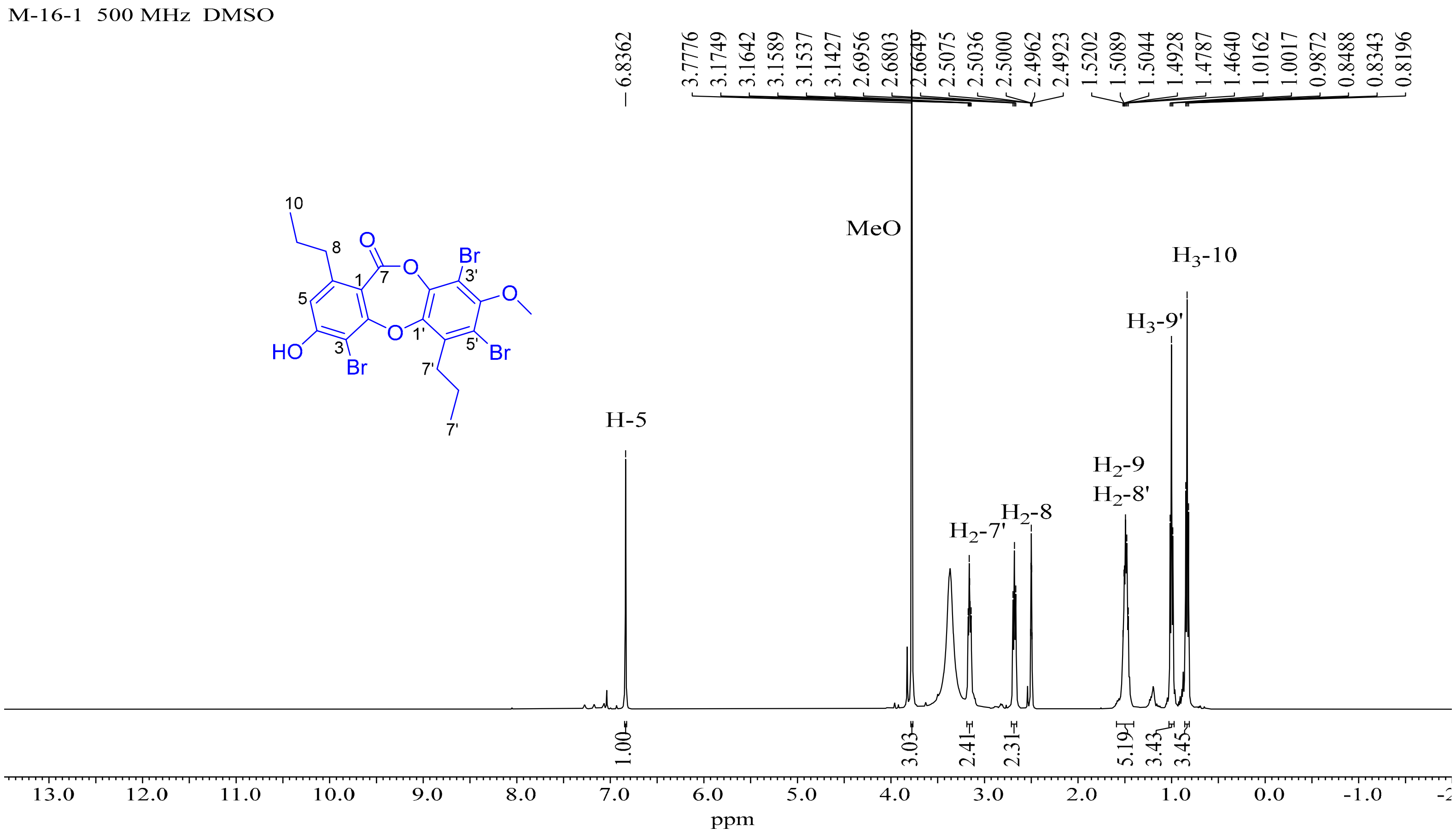

Figure S49. ^{1}H-NMR spectrum of **7** in DMSO-d_6 (500 MHz)

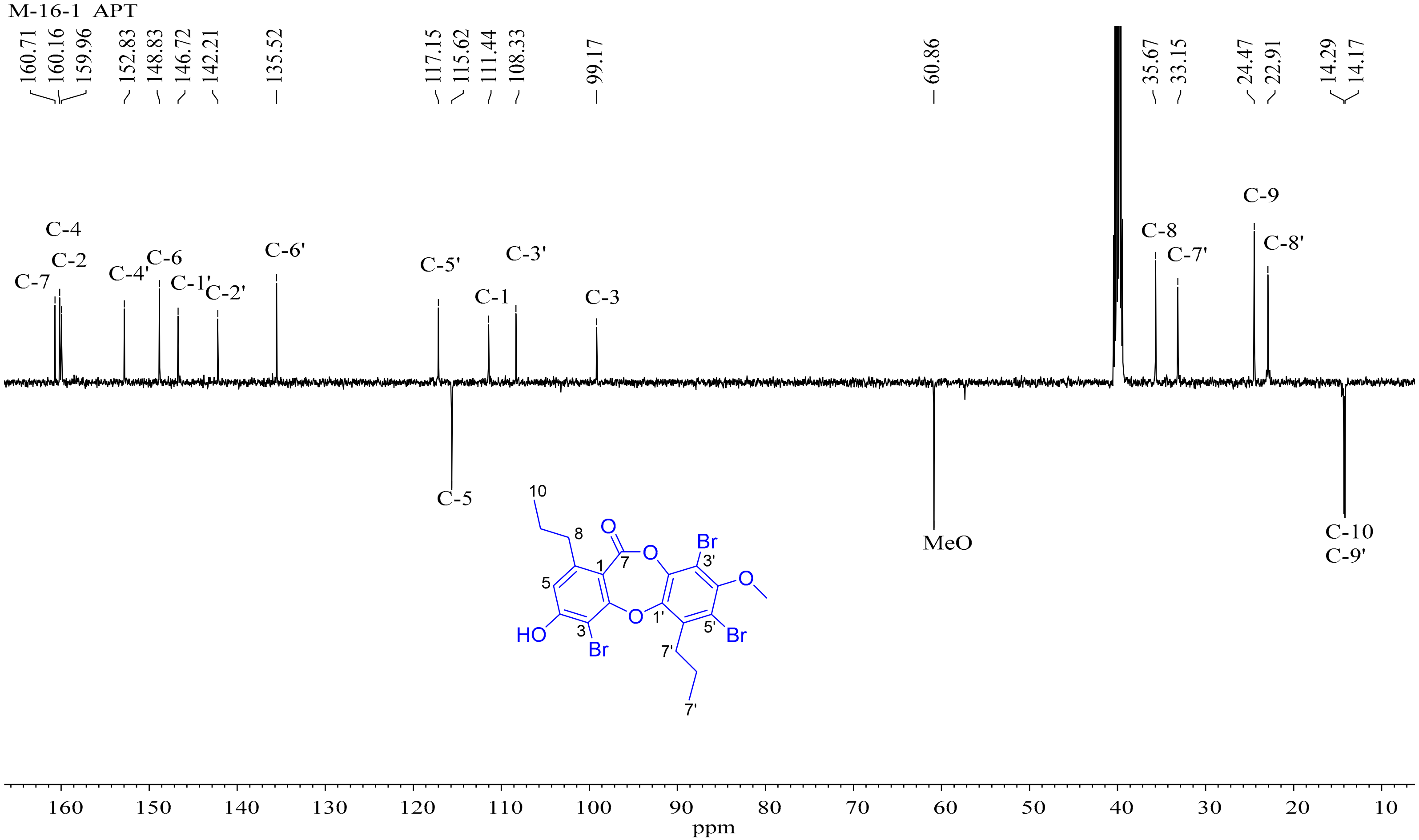

Figure S50. APT spectrum of **7** in DMSO-d_6 (125 MHz)

M-16-1 HSQC

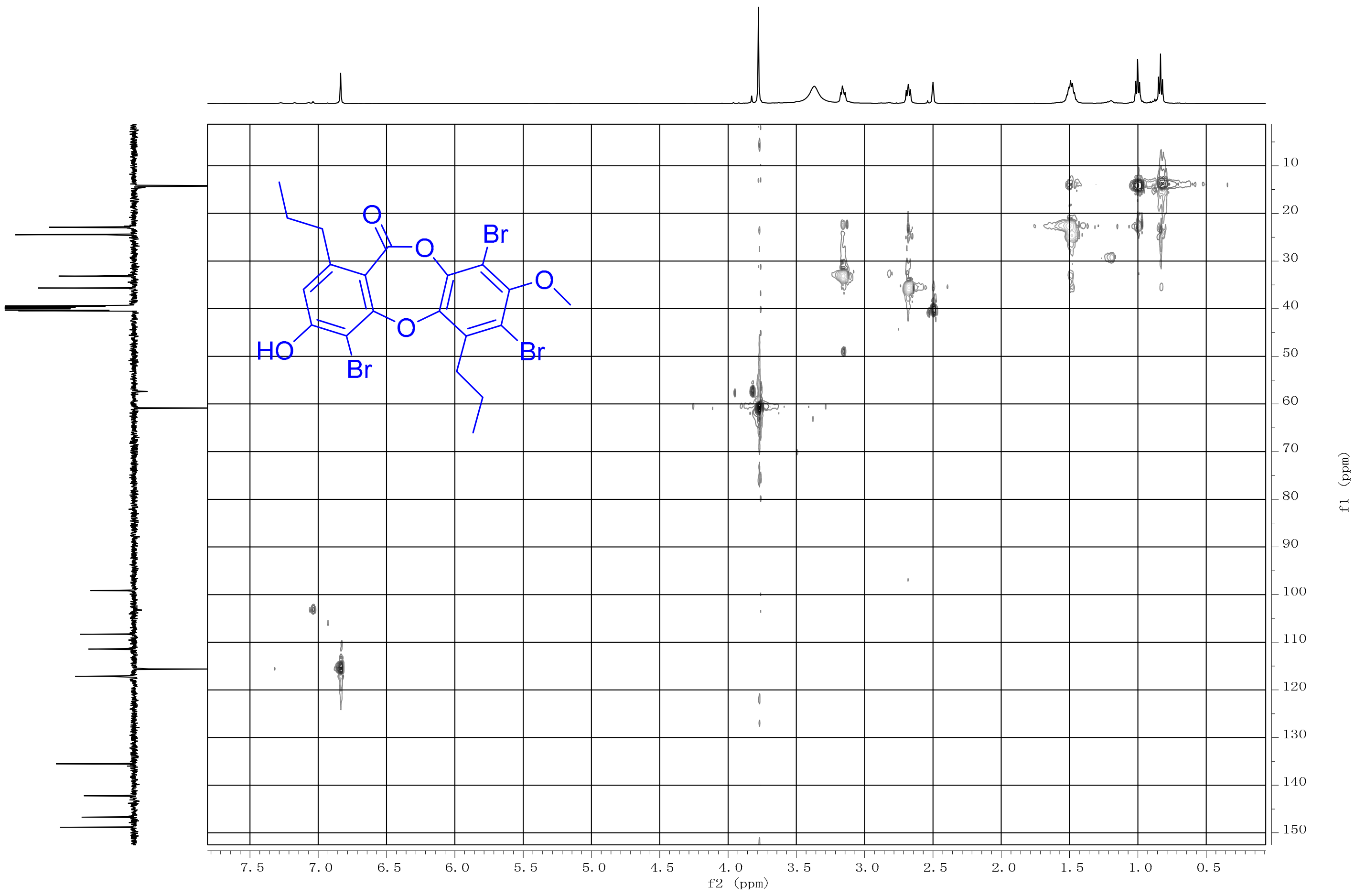

Figure S51. HSQC spectrum of **7** in DMSO-d_6

M-16-1 COSY

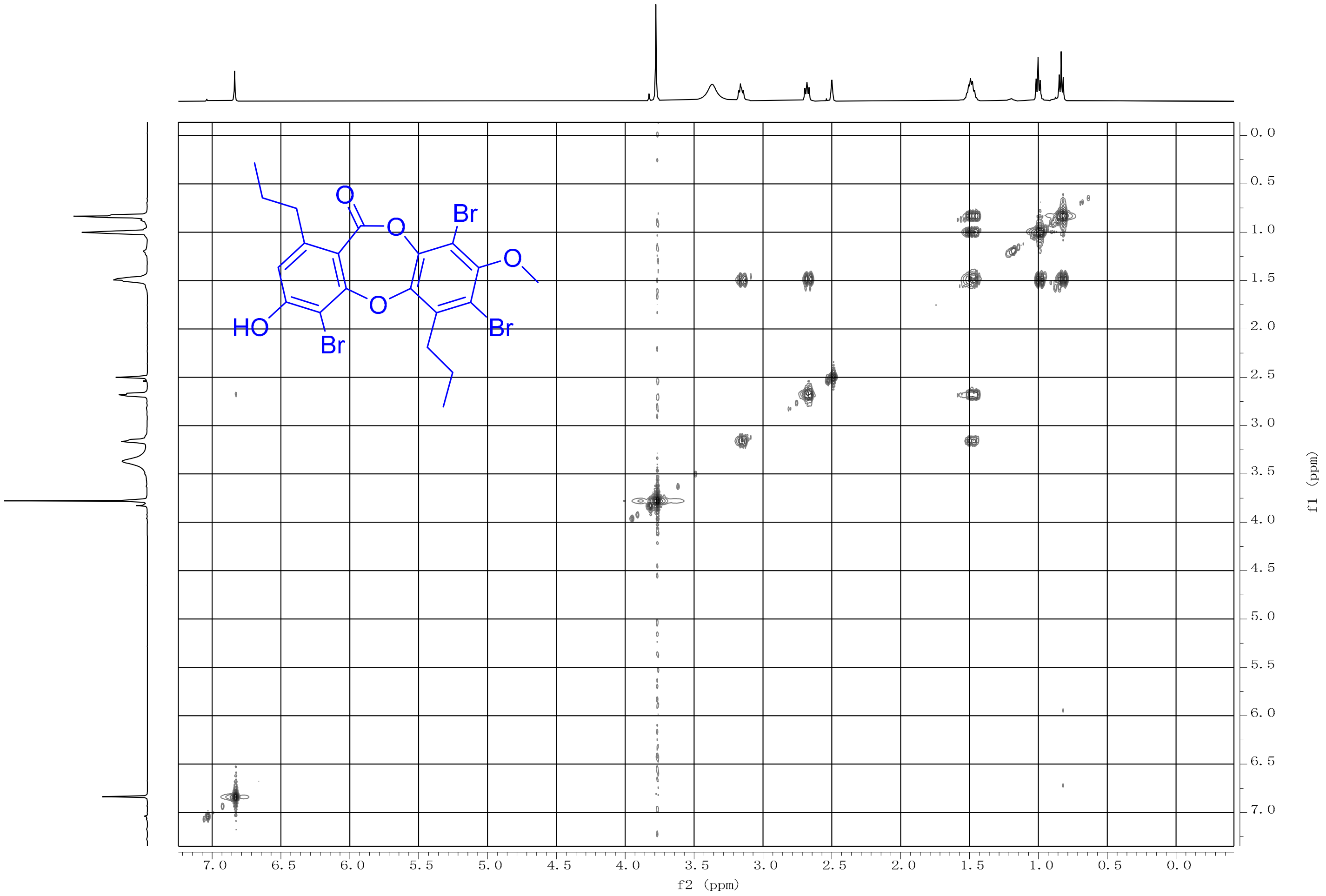

Figure S52. ^{1}H-^{1}H COSY spectrum of **7** in DMSO-d_6

M-16-1 HMBC

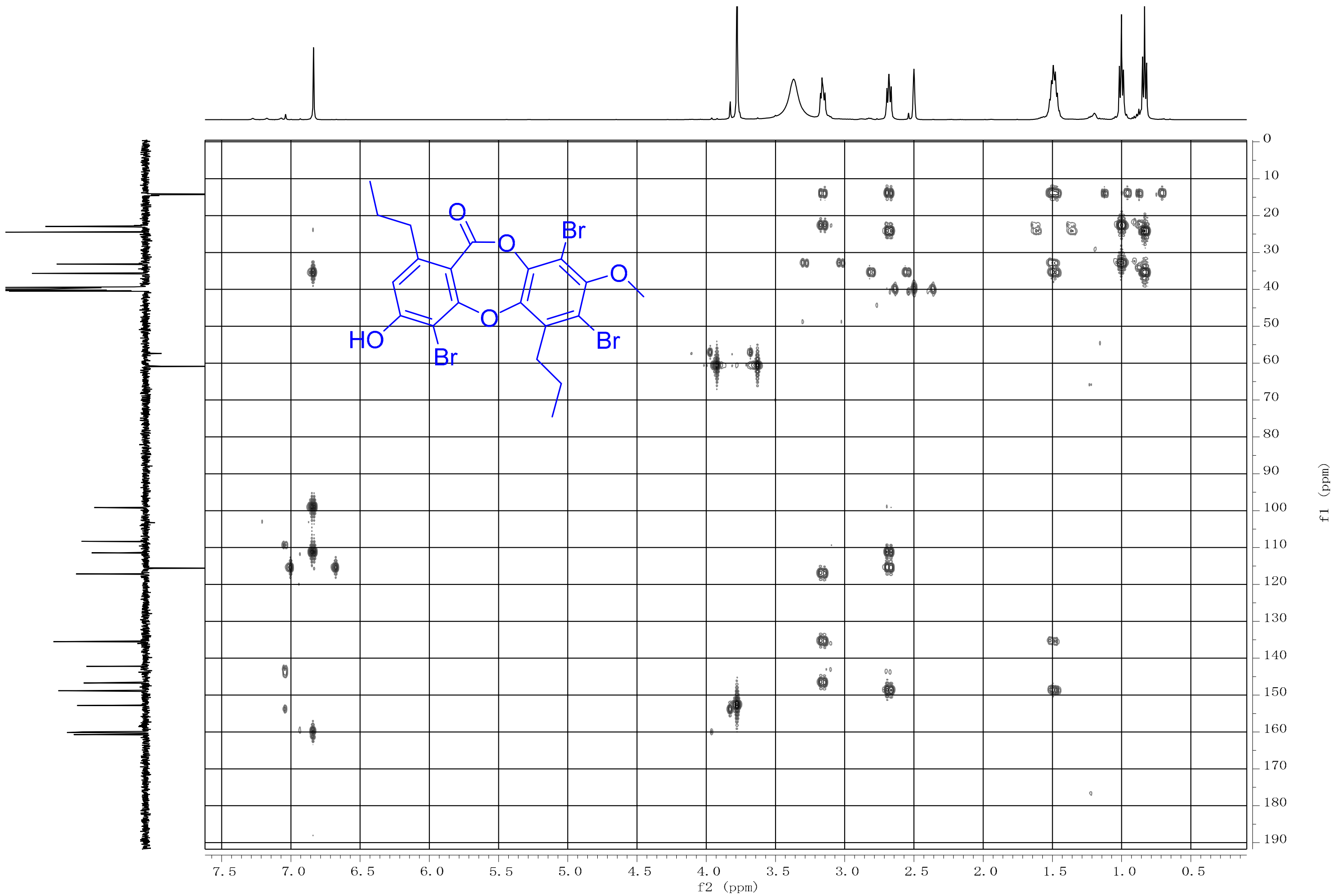

Figure S53. HMBC spectrum of **7** in DMSO-d_6

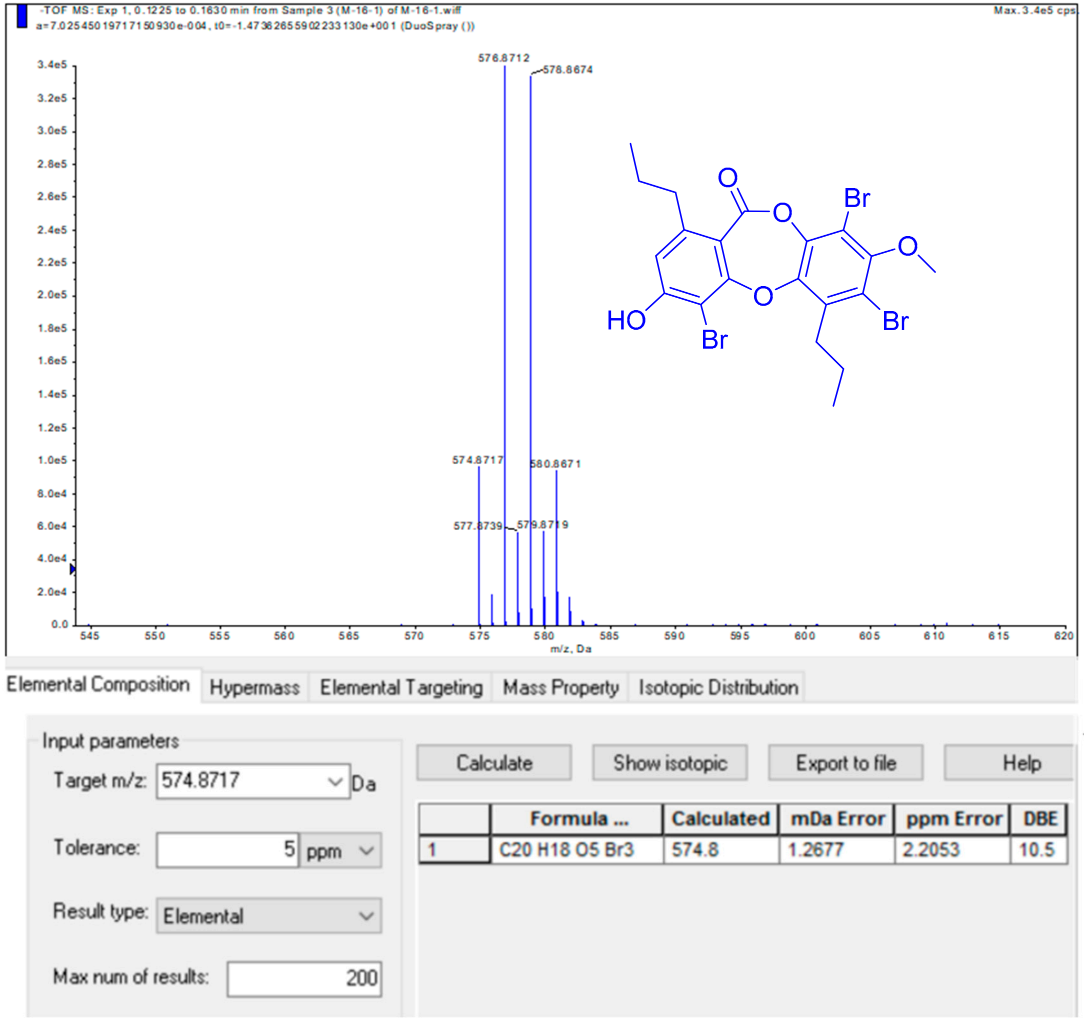

Figure S54. HRESIMS spectrum of **7**

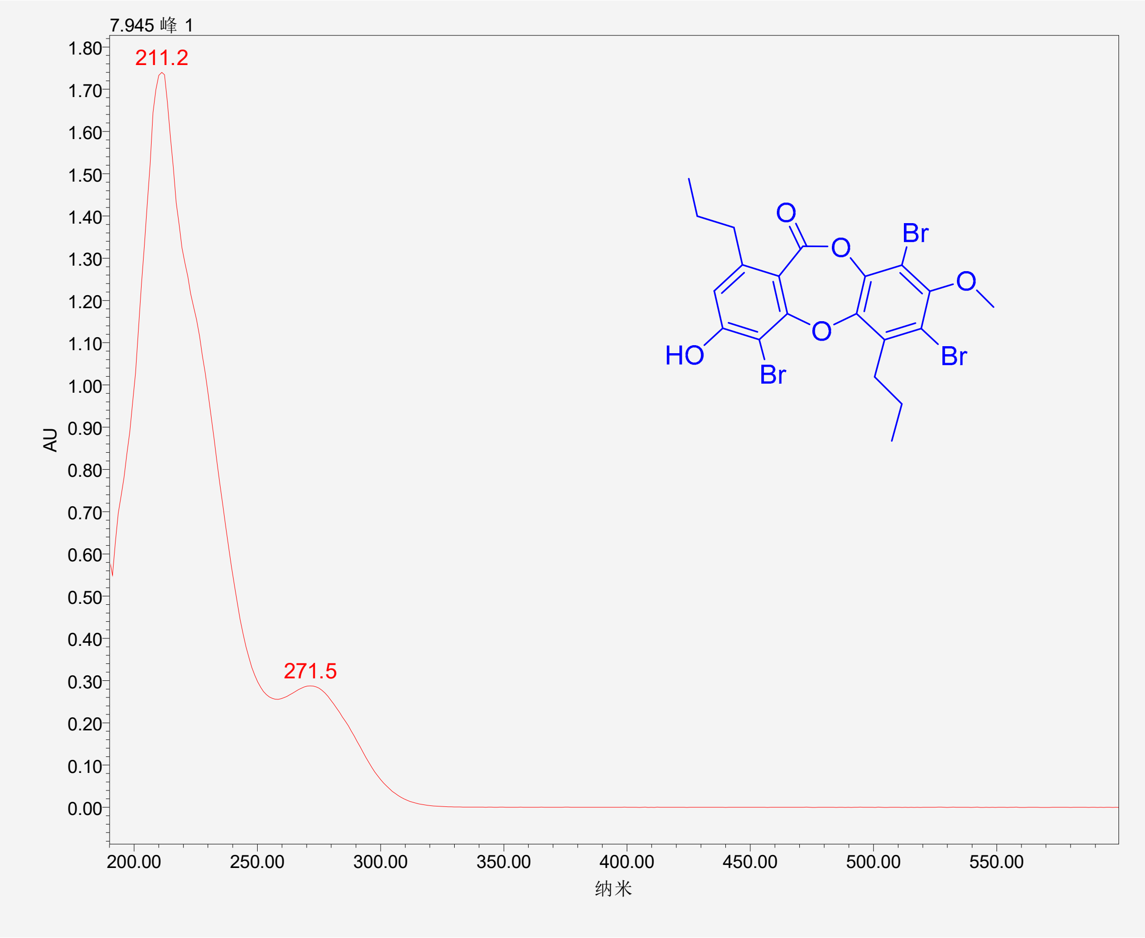

Figure S55. UV spectrum of **7**

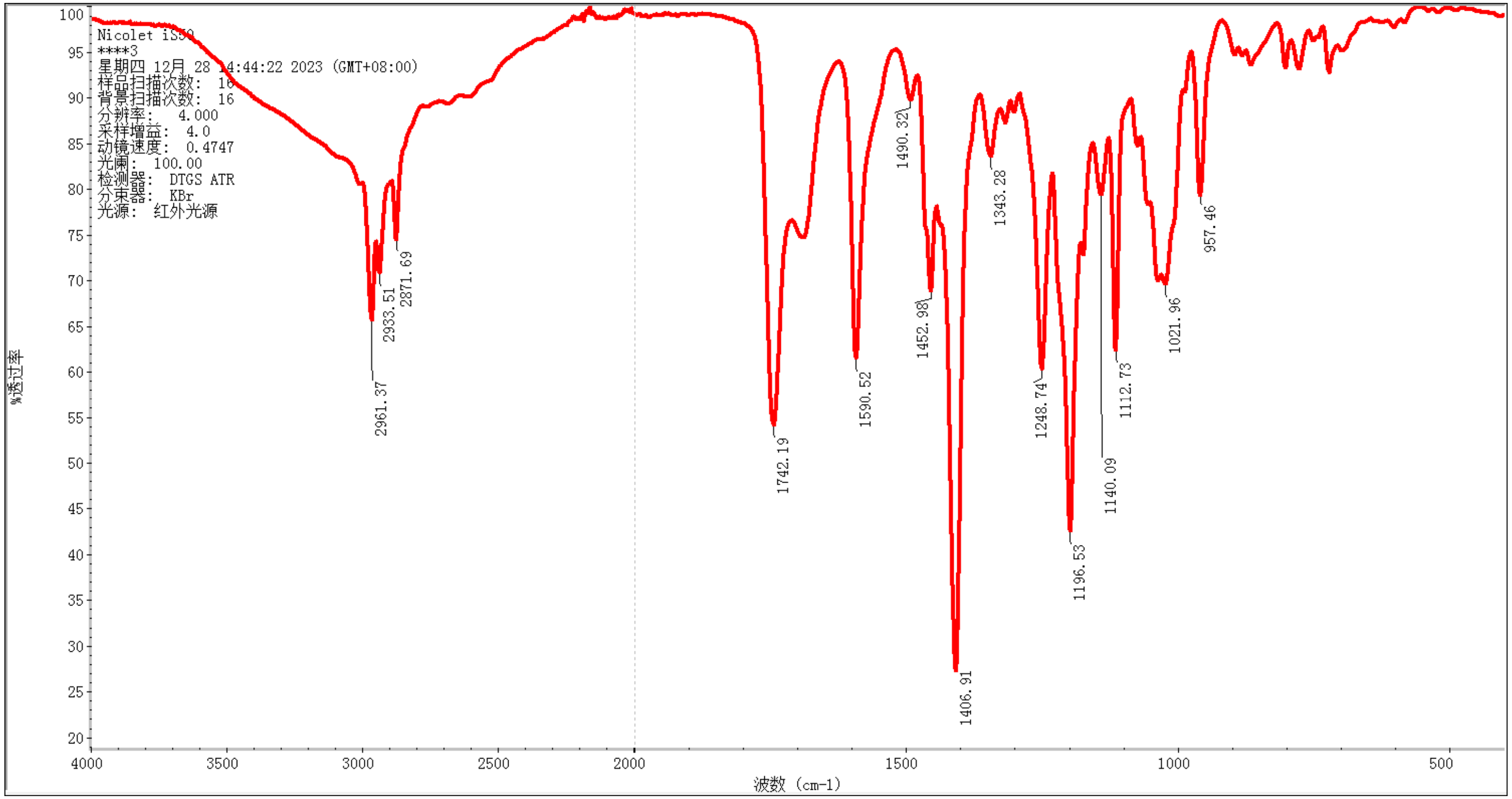

Figure S56. IR spectrum of **7**

Table S8. ^{1}H and ^{13}C NMR data and key COSY and HMBC correlations of 8

No.	δ_H	δ_C	COSY	HMBC
1		111.7, C		
2		161.0, C		
3	6.84, s	105.7, CH		C-1, C-2, C-3, C-4
4		158.4, C		
5		112.4, C		
6		147.3, C		
7		161.2, C		
8	2.85, m	35.7, CH_2	H_2-9	C-1, C-5, C-6, C-9, C-10
9	1.55, m	22.8, CH_2	H_2-8, H_3-10	C-6, C-8, C-10
10	0.88, t (7.2)	14.2, CH_3	H_2-9	C-8, C-9
1′		146.1, C		
2′		142.5, C		
3′		108.0, C		
4′		152.5, C		
5′		116.6, C		
6′		135.2, C		
7′	2.87, m	32.5, CH_2	H_2-8’	C-1', C-5', C-6', C-8', C-9'
8′	1.55, m	22.5, CH_2	H_2-7’, H_3-9’	C-6', C-7', C-9'
9′	1.04, t (7.2)	14.5, CH_3	H_2-8’	C-7', C-8'
MeO	3.76, s	60.9, CH_3		C-4’

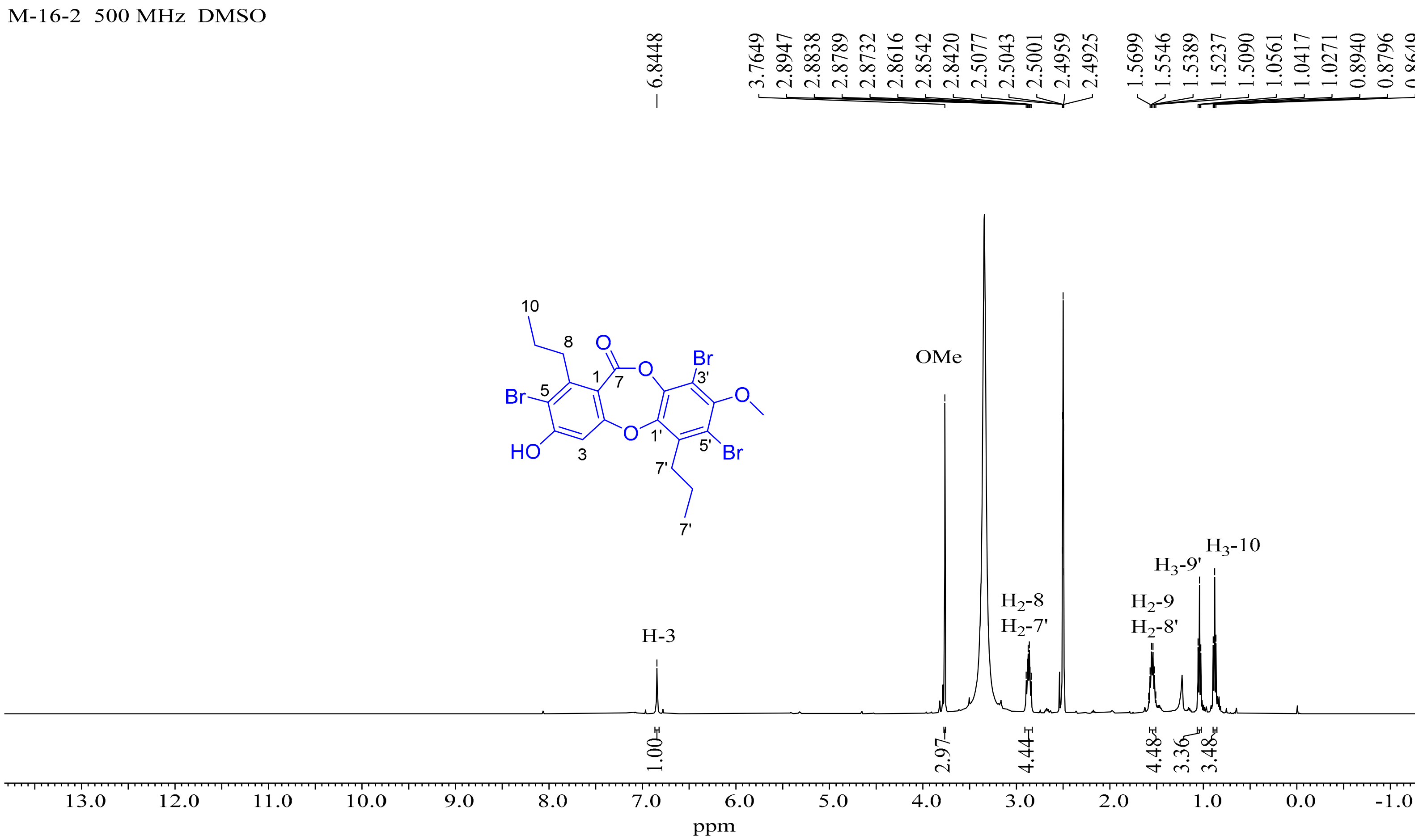

Figure S57. ^{1}H-NMR spectrum of **8** in DMSO-d_6 (500 MHz)

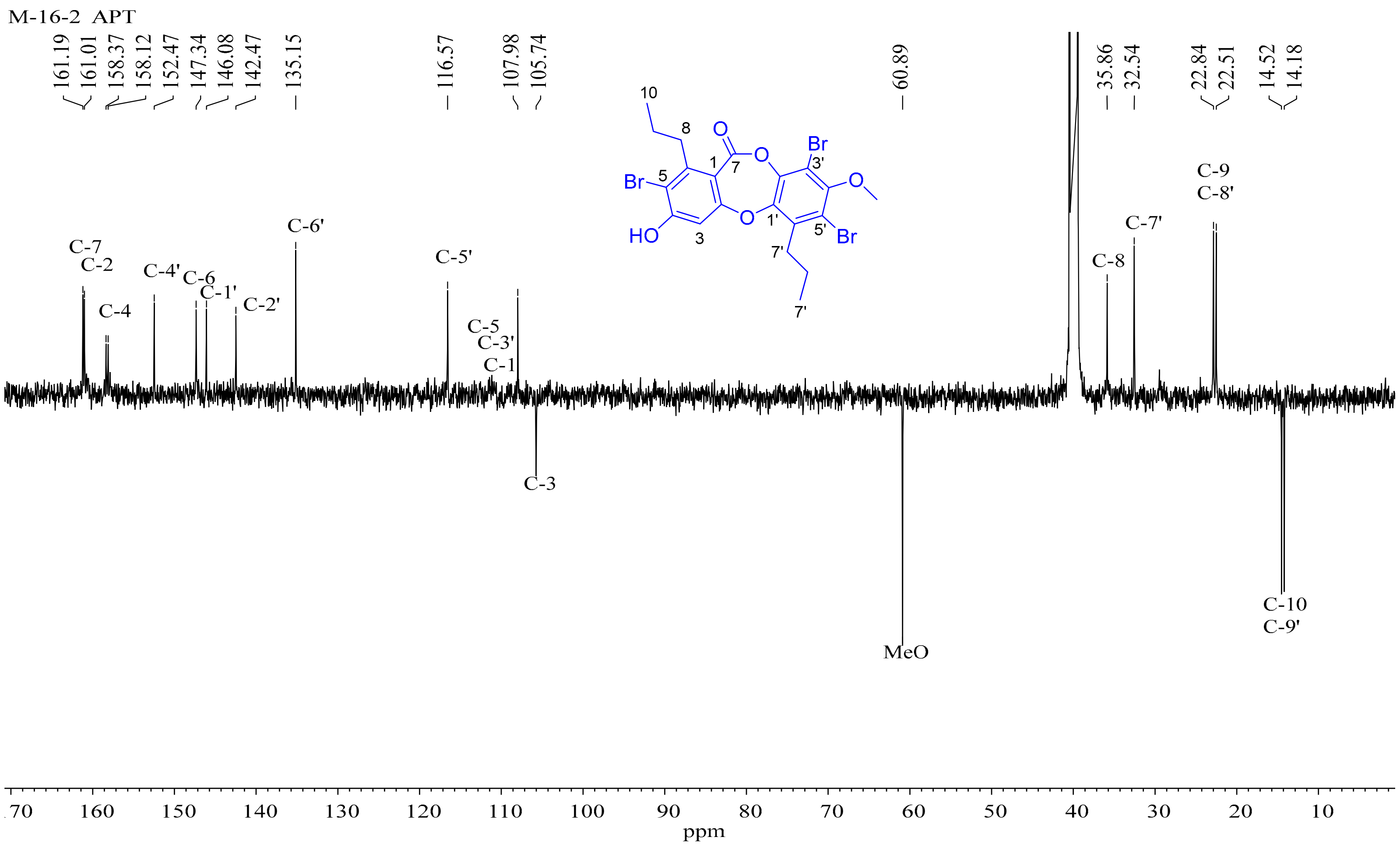

Figure S58. APT spectrum of **8** in DMSO-d_6 (125 MHz)

M-16-2 HSQC

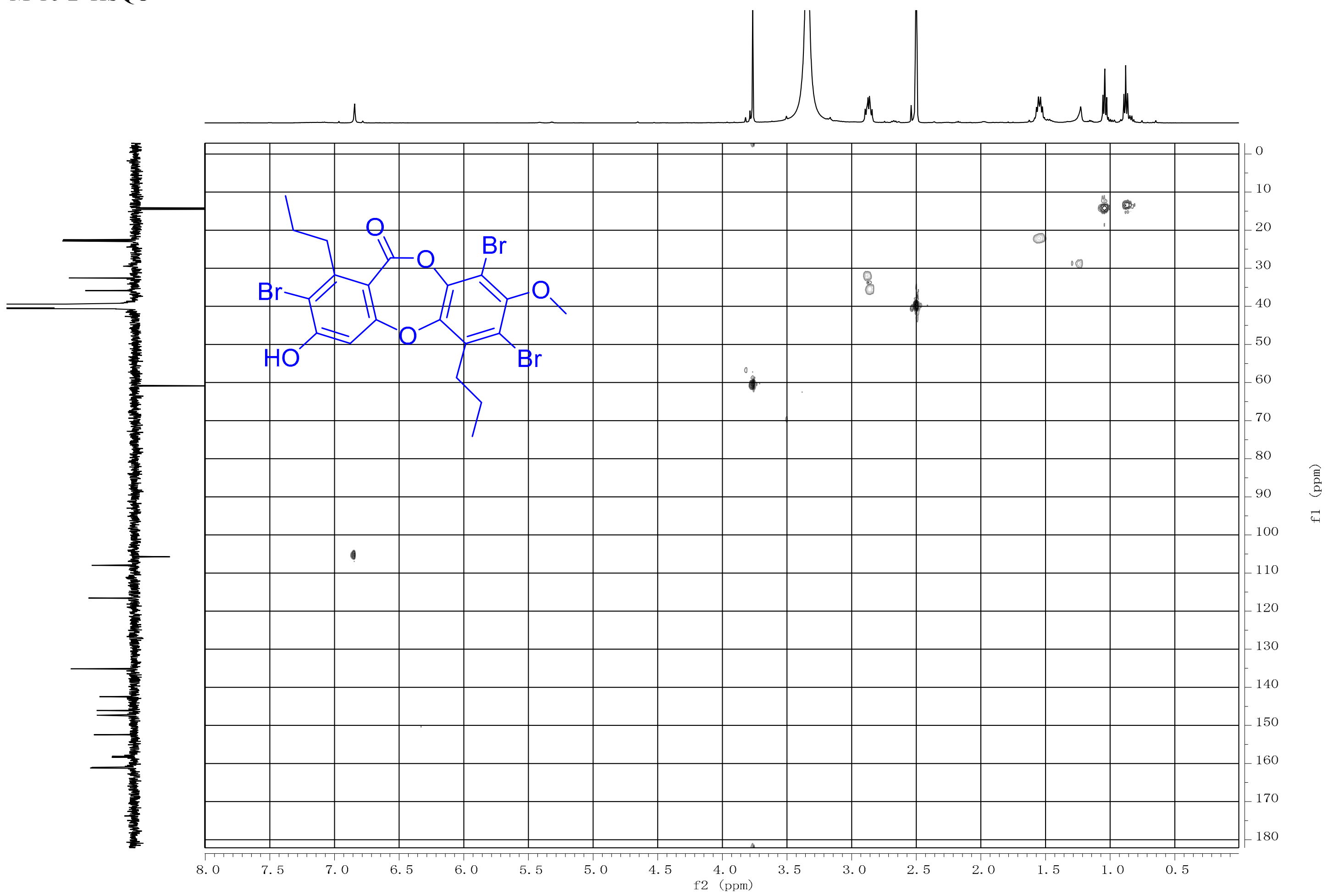

Figure S59. HSQC spectrum of **8** in DMSO-d_6

M-16-2 COSY

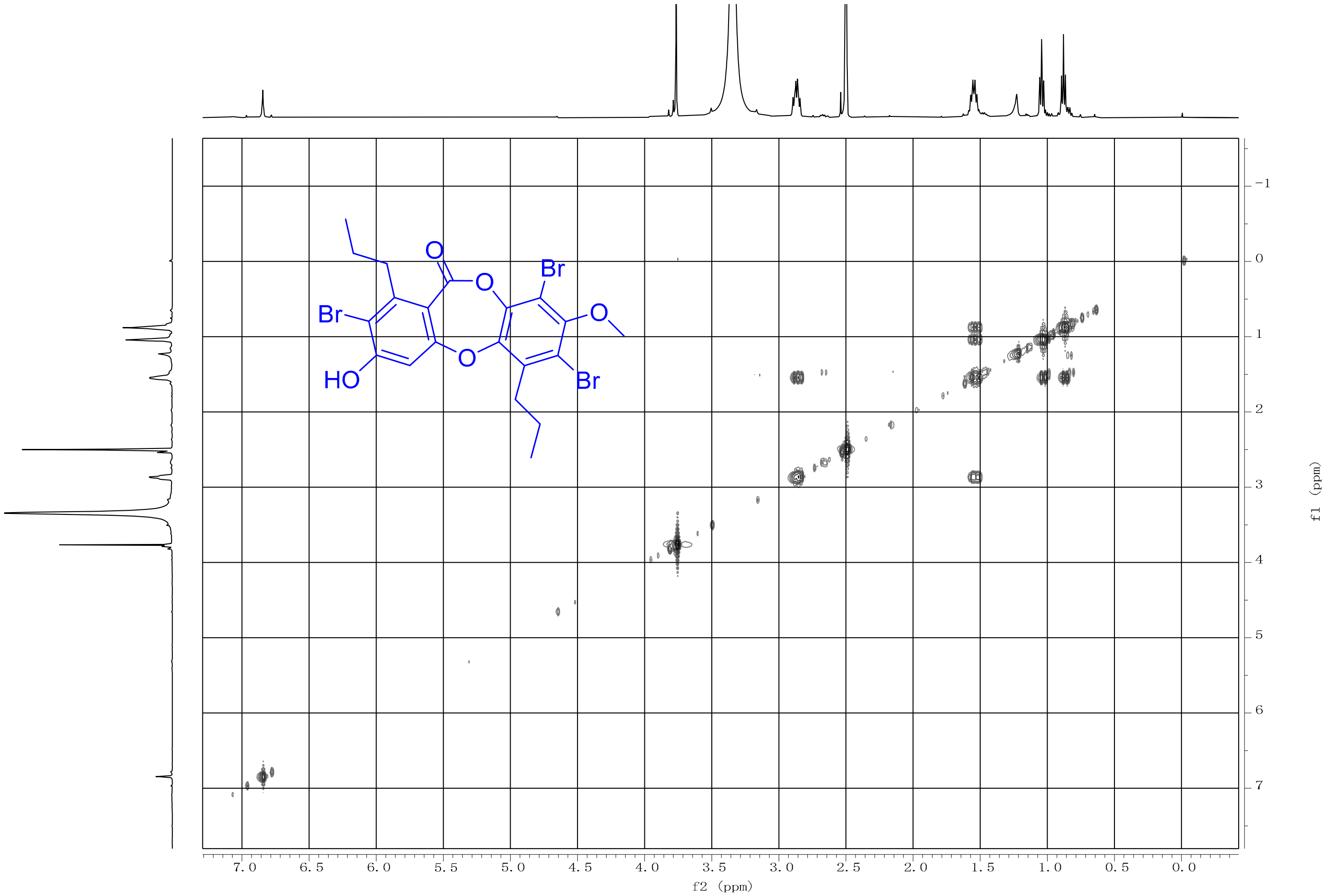

Figure S60. ${}^{1}H$-${}^{1}H$ COSY spectrum of **8** in DMSO-d_6

M-16-2 HMBC

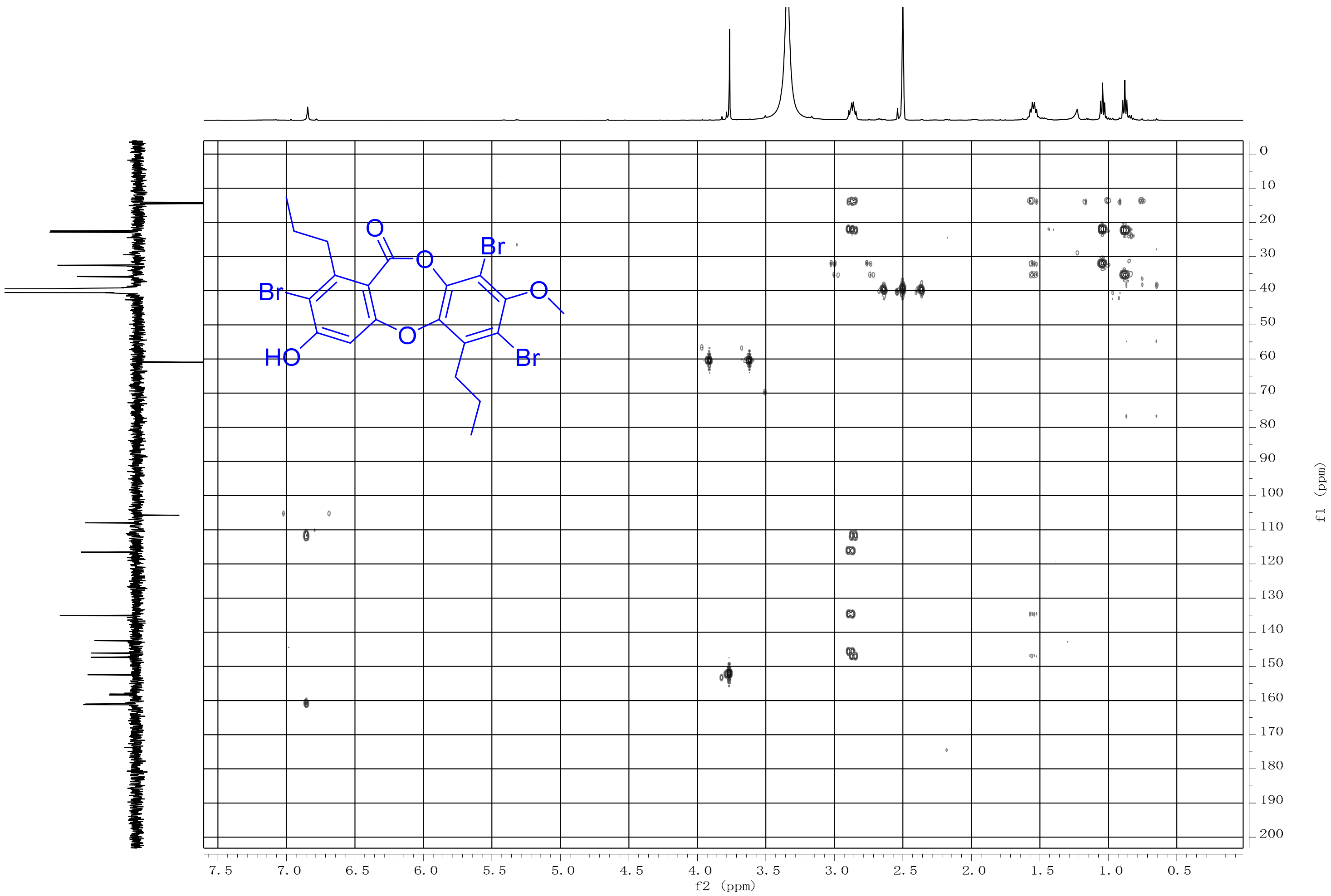

Figure S61. HMBC spectrum of **8** in DMSO-d_6

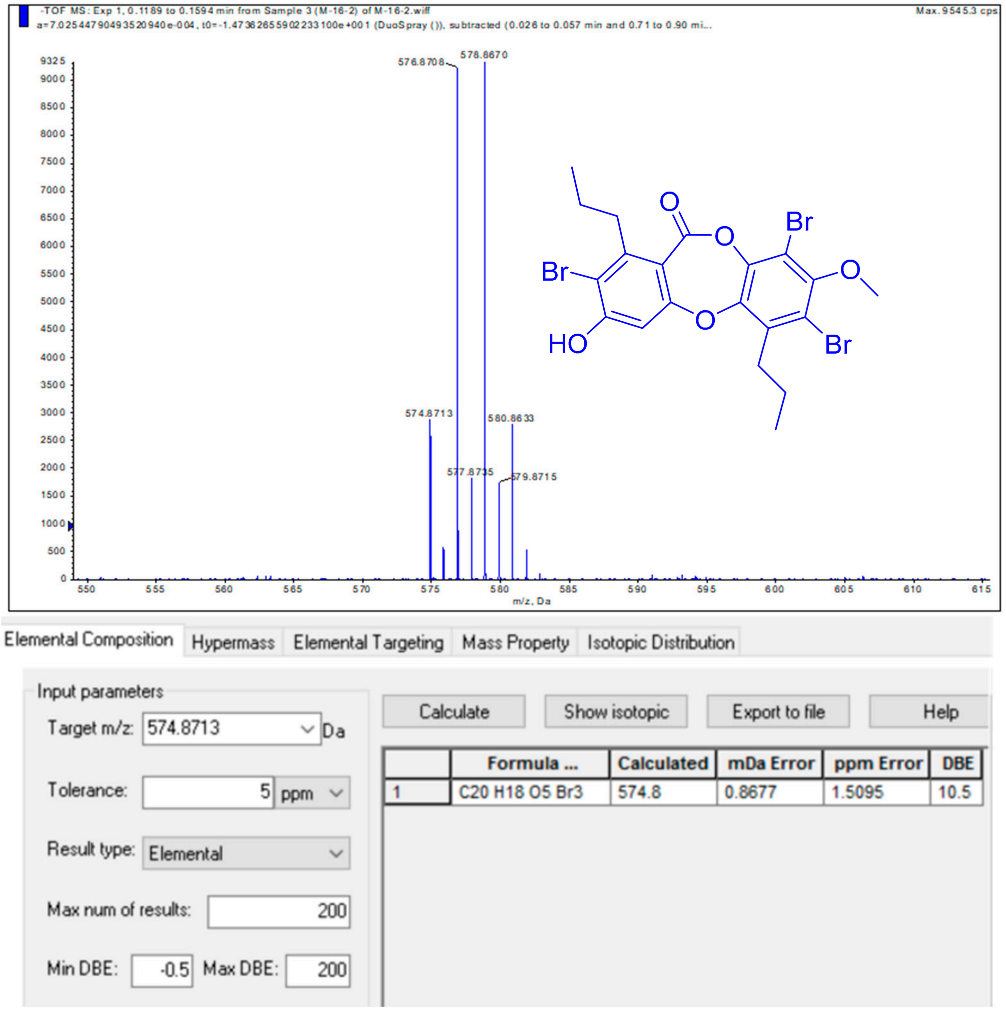

Figure S62. HRESIMS spectrum of **8**

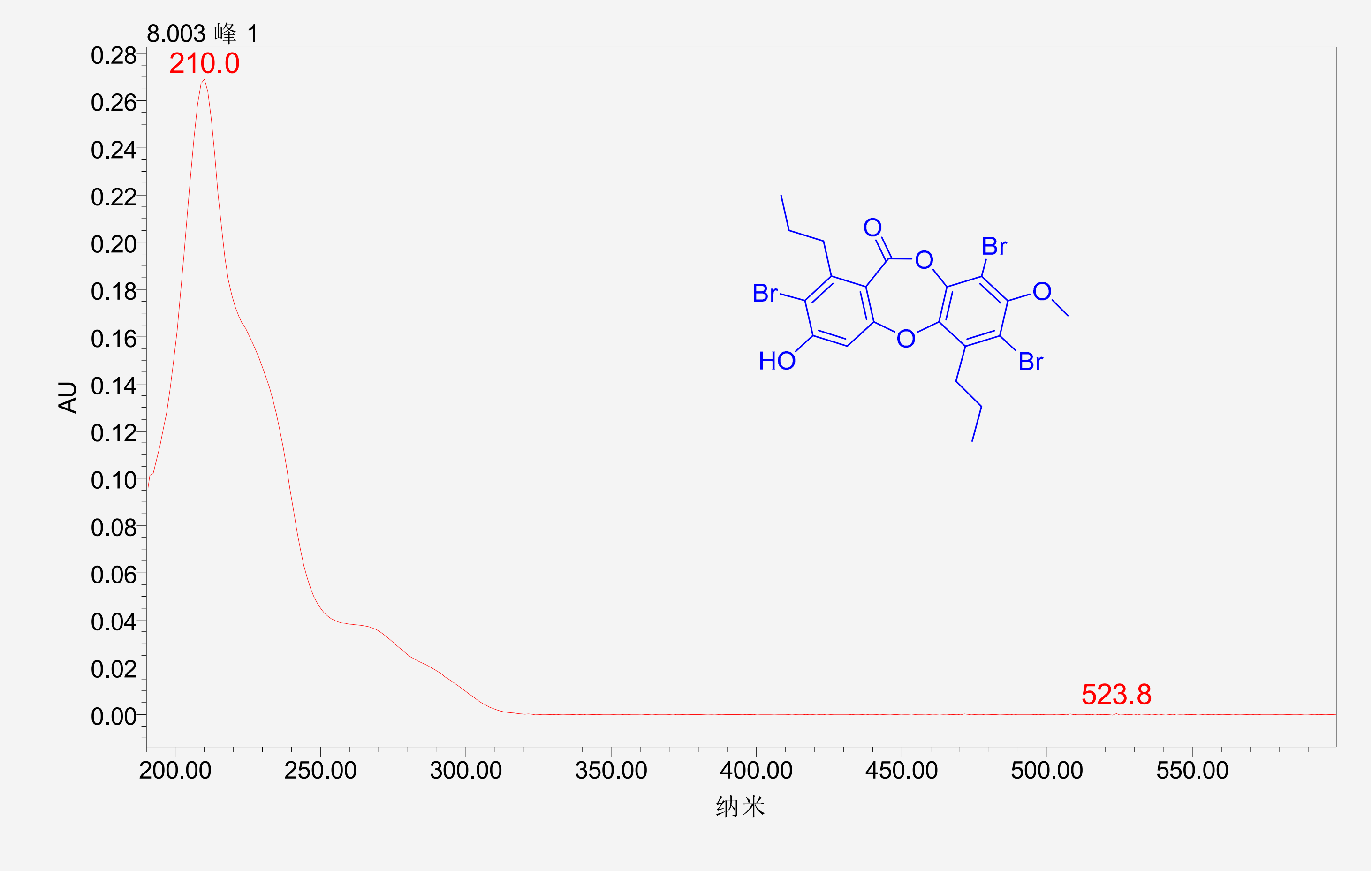

Figure S63. UV spectrum of **8**

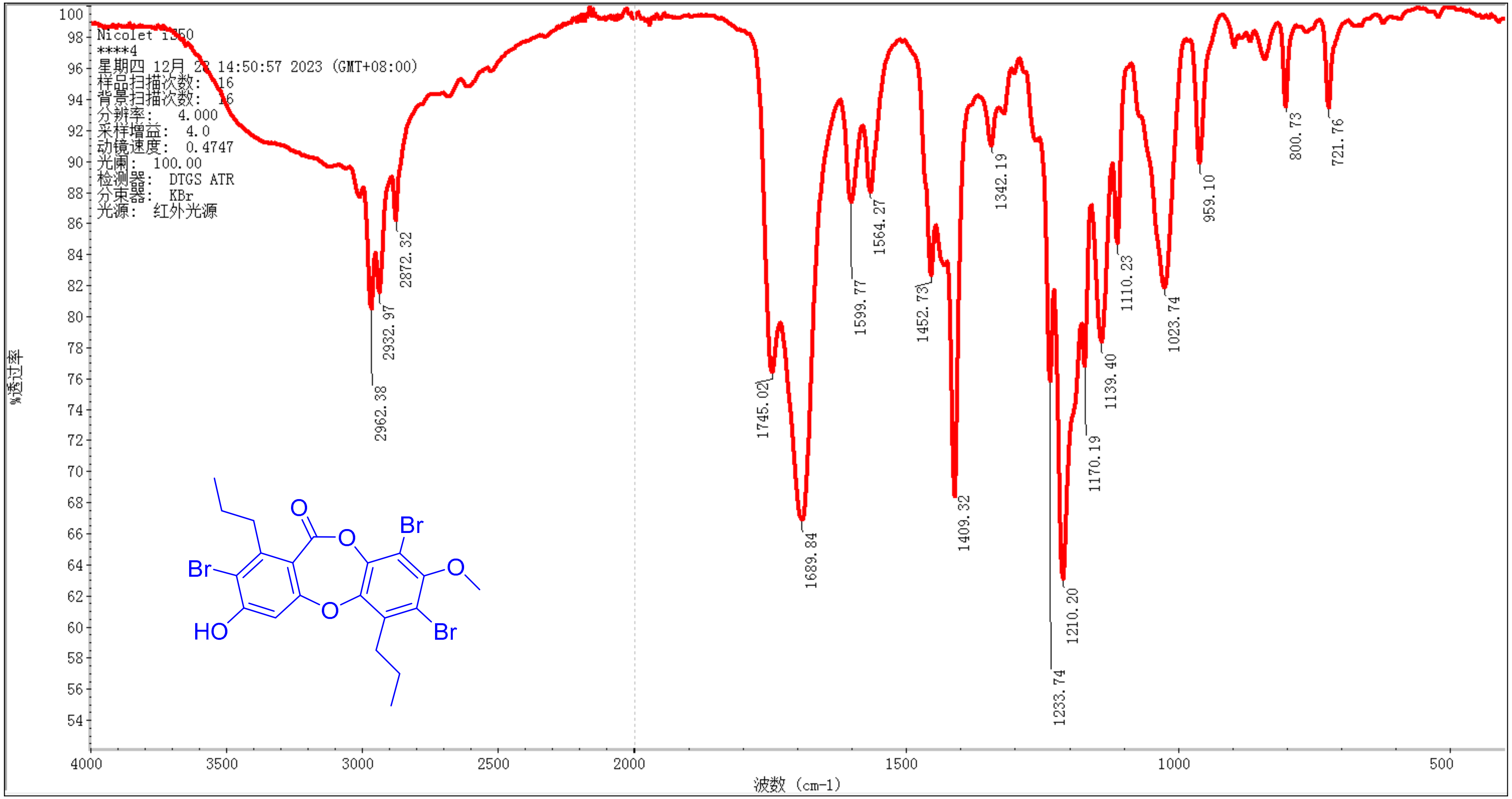

Figure S64. IR spectrum of **8**

Table S9. ^{1}H and ^{13}C NMR data and key COSY and HMBC correlations of 9

No.	δ_H	δ_C	COSY	HMBC
1		112.7, C		
2		158.8, C		
3		101.5, C		
4		158.8, C		
5		113.8, C		
6		146.1, C		
7		160.6, C		
8	2.81, t (7.7)	36.6, CH_2	H_2-9	C-1, C-5, C-6, C-9, C-10
9	1.58, m	22.7, CH_2	H_2-8, H_3-10	C-6, C-8, C-10
10	0.87, t (7.3)	14.1, CH_3	H_2-9	C-8, C-9
1′		146.6, C		
2′		142.2, C		
3′		108.3, C		
4′		152.9, C		
5′		117.2, C		
6′		135.5, C		
7′	3.15, t (7.9)	33.2, CH_2	H_2-8’	C-1', C-5', C-6', C-8', C-9'
8′	1.50, m	22.9, CH_2	H_2-7’, H_3-9’	C-6', C-7', C-9'
9′	1.02, t (7.3)	14.3, CH_3	H_2-8’	C-7', C-8'
MeO	3.78, s	60.8, CH_3		C-4’

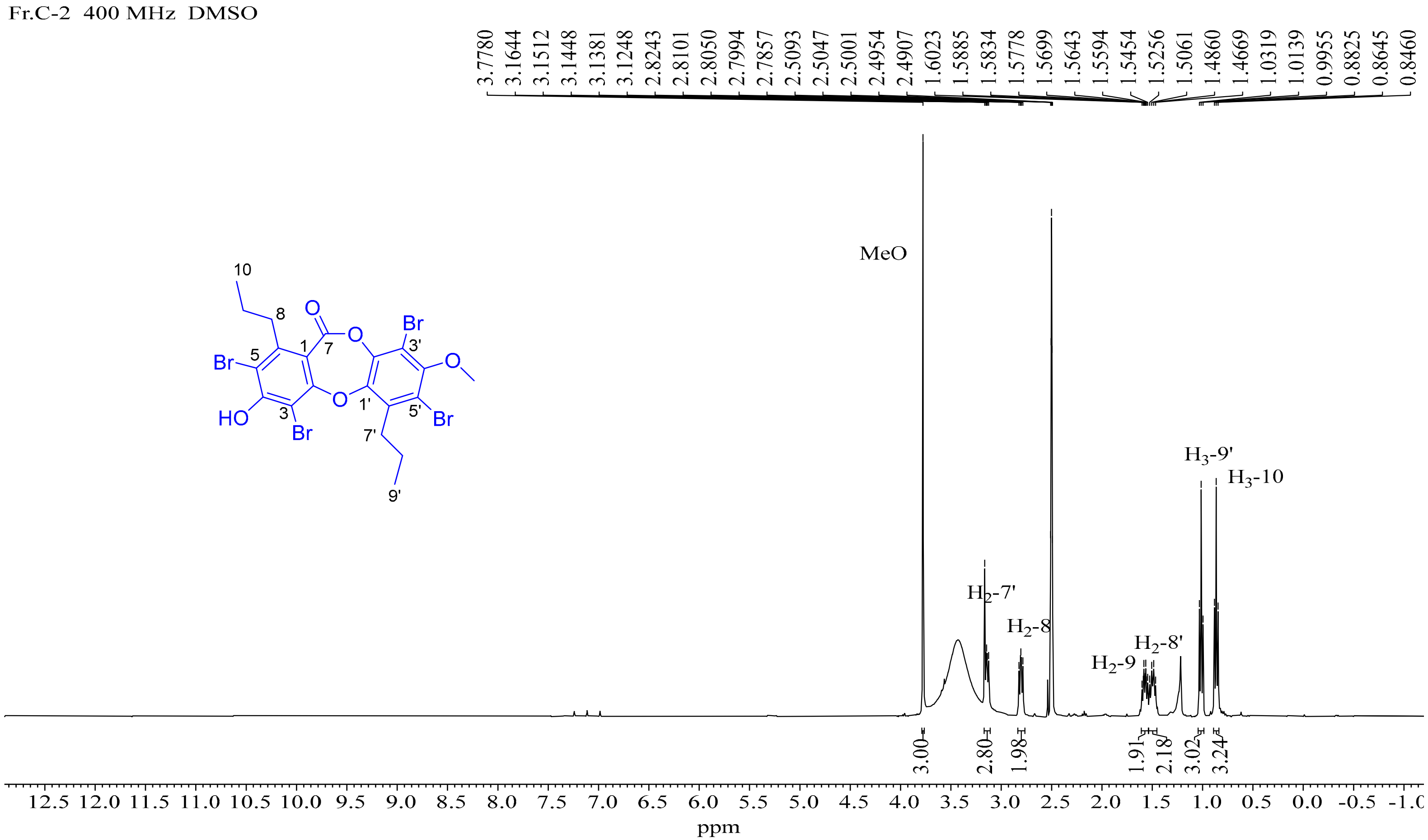

Figure S65. ^{1}H-NMR spectrum of **9** in DMSO-d_6 (400 MHz)

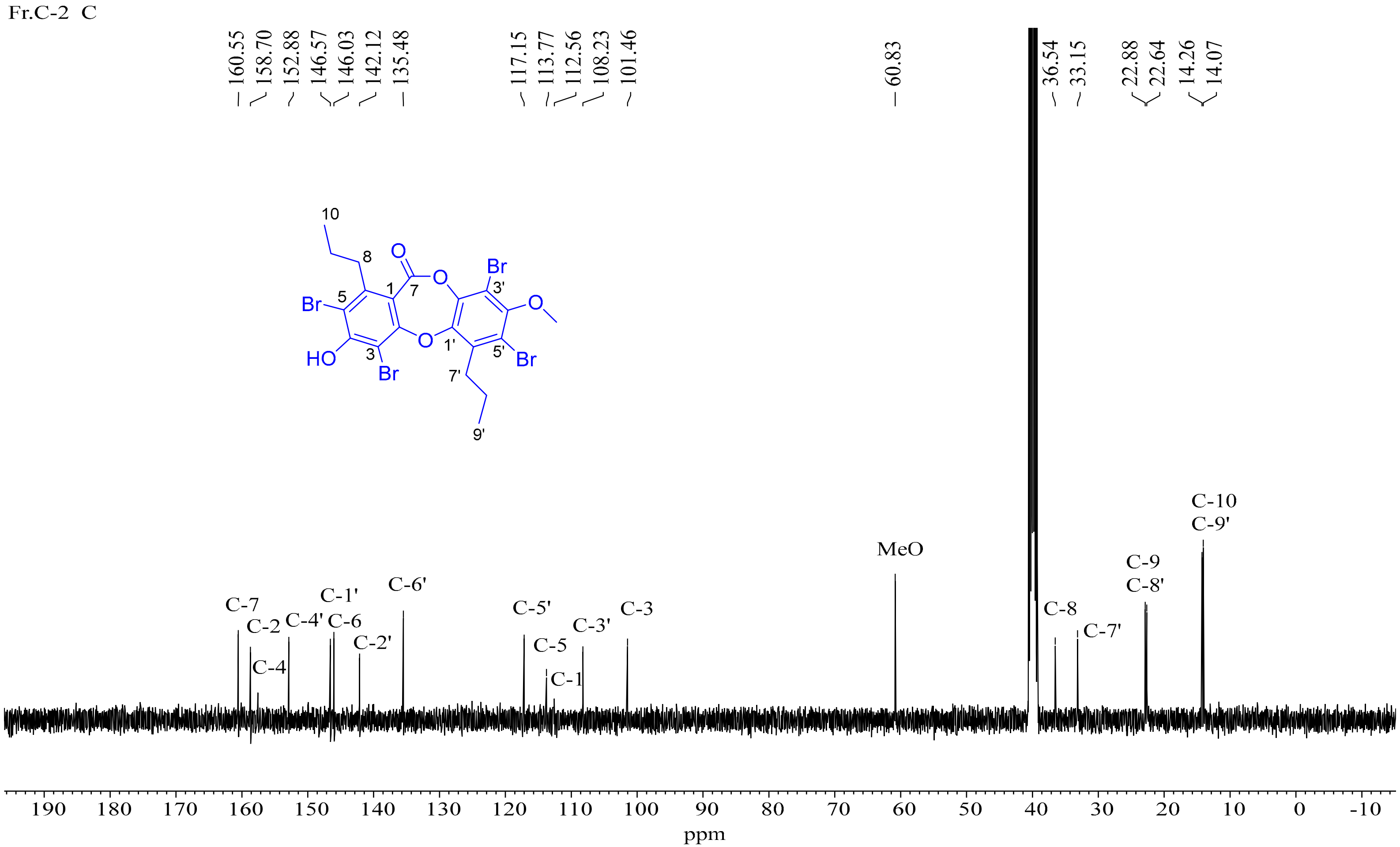

Figure S66. ^{13}C-NMR spectrum of **9** in DMSO-*d*6 (100 MHz)

Fr.C-2 HSQC

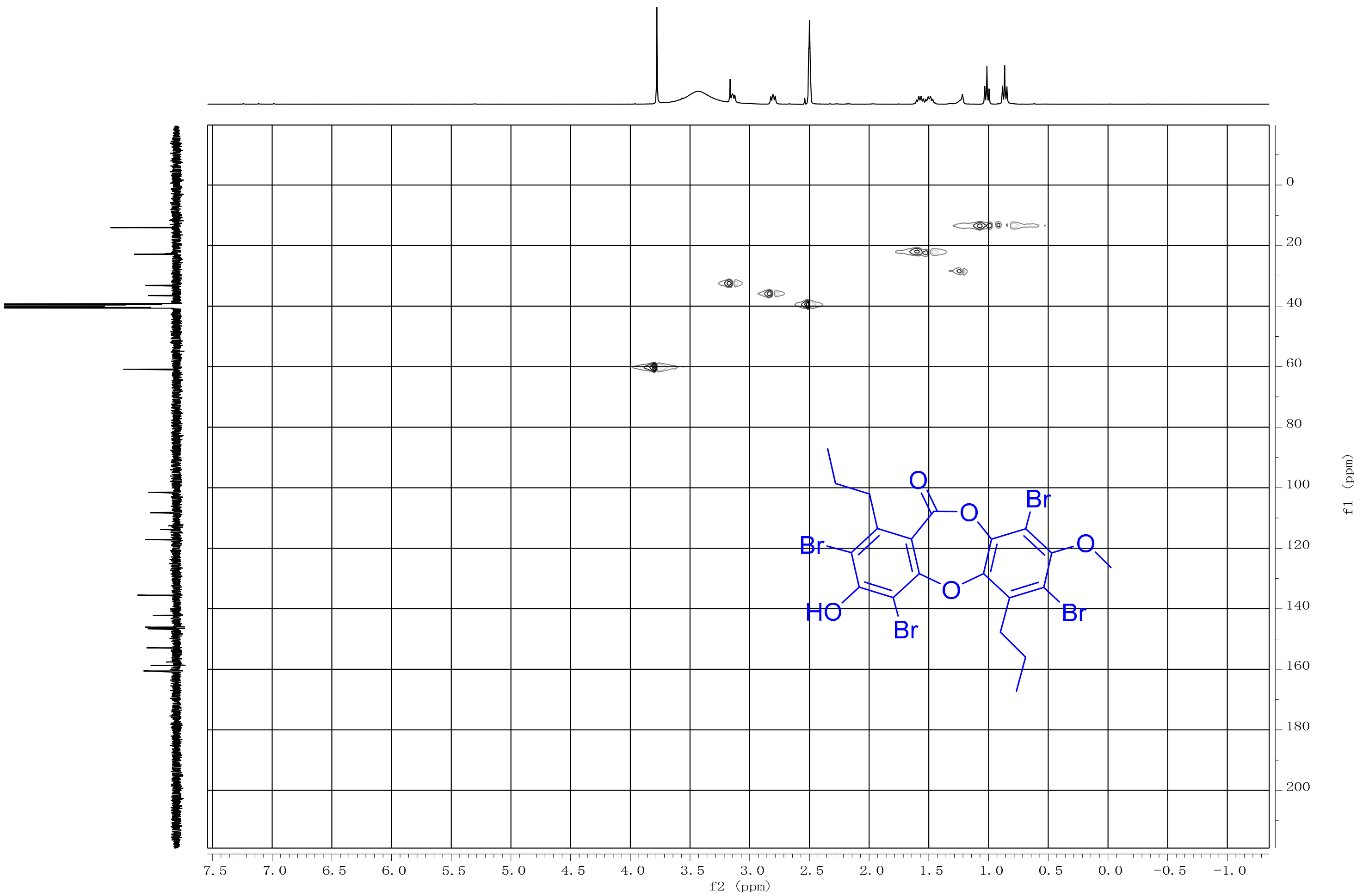

Figure S67. HSQC spectrum of **9** in DMSO-d_6

Fr.C-2 COSY

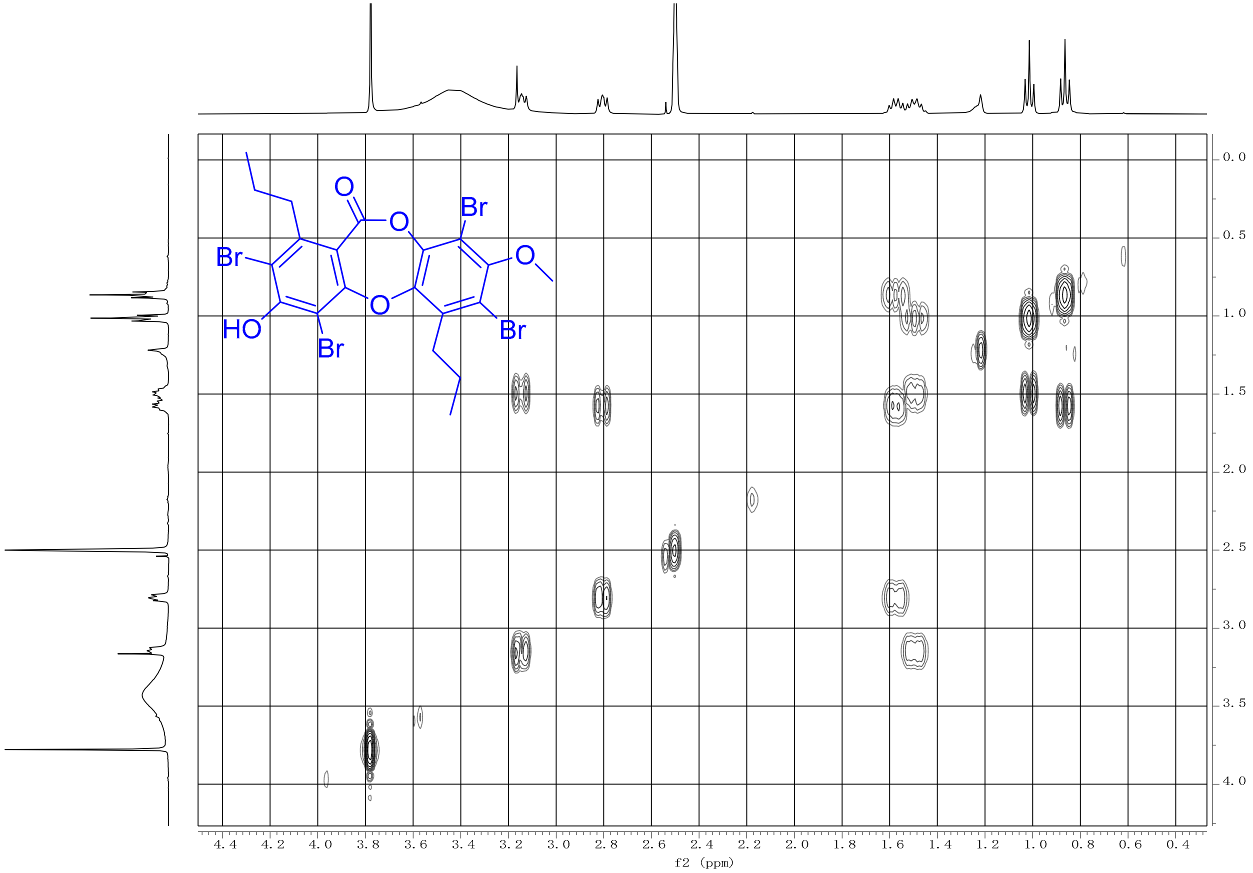

Figure S68. ¹H-¹H COSY spectrum of **9** in DMSO-d_6

Fr.C-2 HMBC

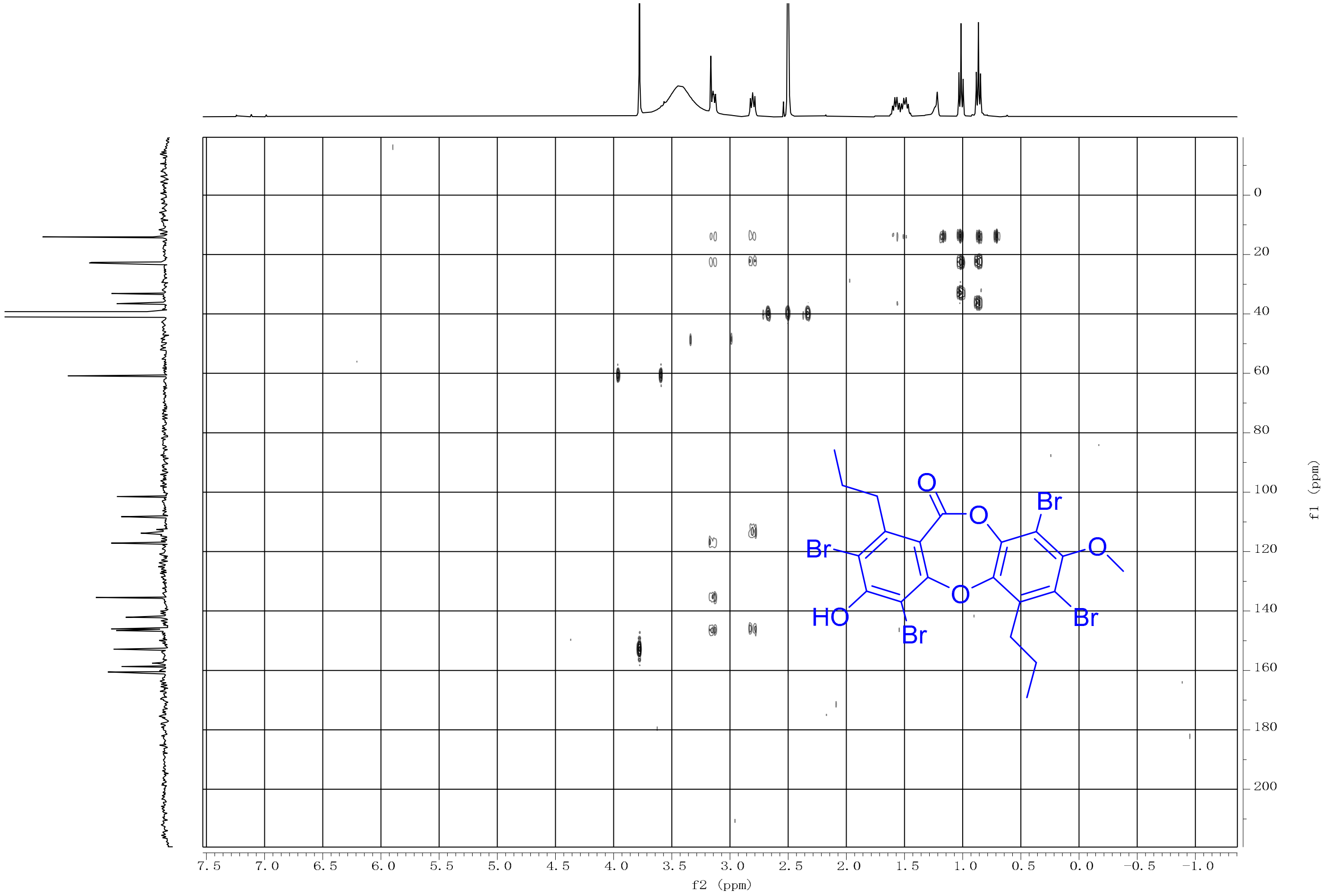

Figure S69. HMBC spectrum of **9** in DMSO-d_6

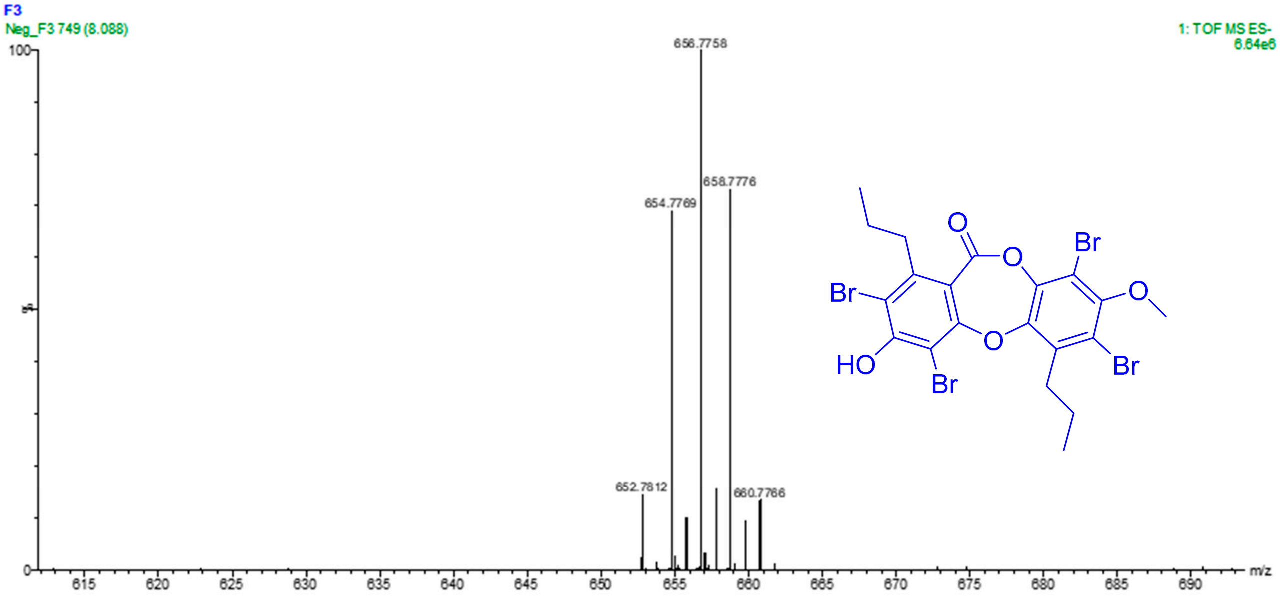

Single Mass Analysis
Tolerance = 5.0 mDa / DBE: min = -1.5, max = 50.0
Element prediction: Off
Monoisotopic Mass, Even Electron Ions
694 formula(e) evaluated with 5 results within limits (up to 50 closest results for each mass)
Elements Used:
C: 0-500 H: 0-1000 O: 0-200 Br: 0-8

Mass	Calc. Mass	mDa	PPM	DBE	Formula	C	H	O	Br
652.7812	652.7810	0.2	0.3	10.5	C20 H17 O5 Br4	20	17	5	4

Figure S70. HRESIMS spectrum of **9**

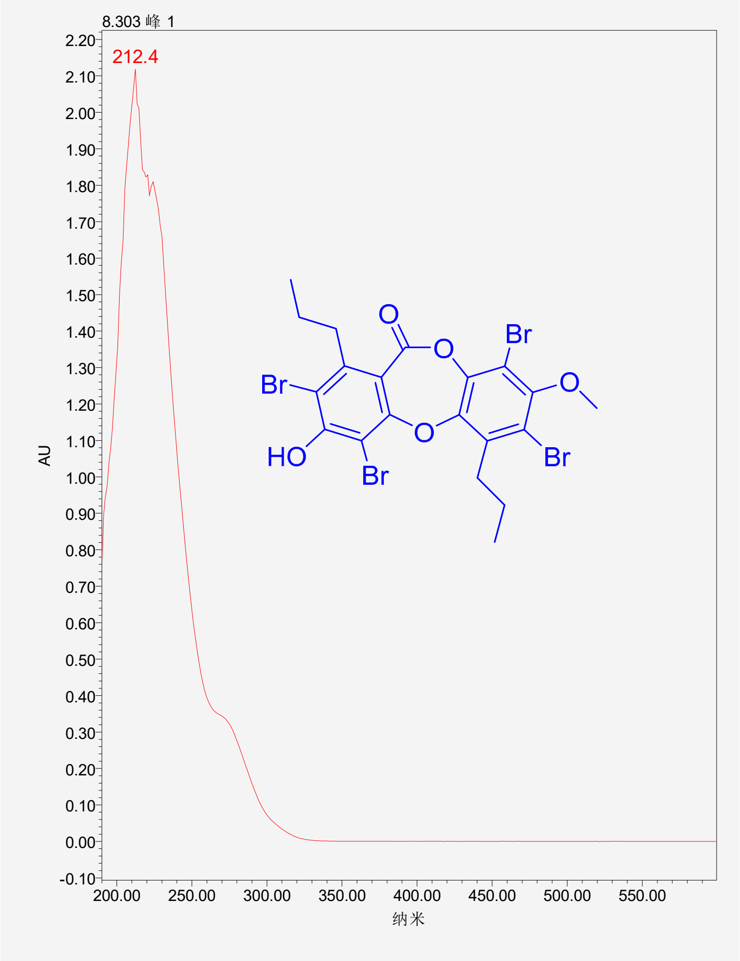

Figure S71. UV spectrum of **9**

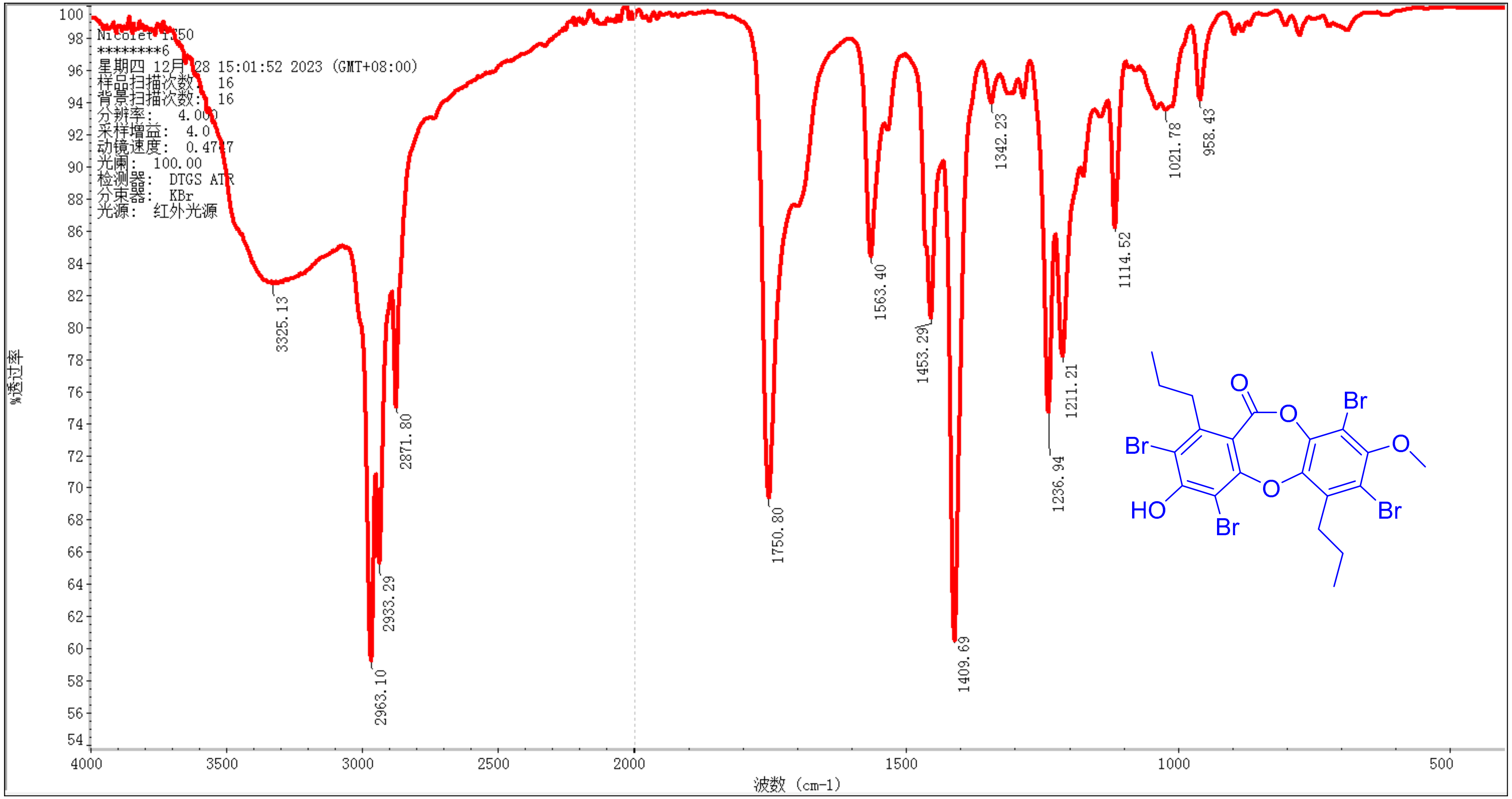

Figure S72. IR spectrum of **9**

Table S10. ^{1}H and ^{13}C NMR data and key COSY and HMBC correlations of 10

No.	δ_H	δ_C	COSY	HMBC
1		112.6, C		
2		157.6, C		
3		102.5, C		
4		157.6, C		
5		114.1, C		
6		144.6, C		
7		161.9, C		
8	2.79, m	36.1, CH_2	H_2-9	C-1, C-5, C-6, C-9, C-10
9	1.53, m	23.0, CH_2	H_2-8, H_3-10	C-6, C-8, C-10
10	0.90, t (7.2)	14.1, CH_3	H_2-9	C-8, C-9
1′		150.1, C		
2′		136.5, C		
3′		135.2, C		
4′		110.8, C		
5′		153.9, C		
6′	7.07, s	103.6, C		C-1’, C-2’, C-4’, C-5’
7′	2.81, m	32.3, CH_2	H_2-8’	C-2', C-3', C-4', C-8', C-9'
8′	1.50, m	22.1, CH_2	H_2-7’, H_3-9’	C-3', C-7', C-9'
9′	0.90, t (7.2)	14.4, CH_3	H_2-8’	C-7', C-8'
MeO	3.85, s	57.3, CH_3		C-5’

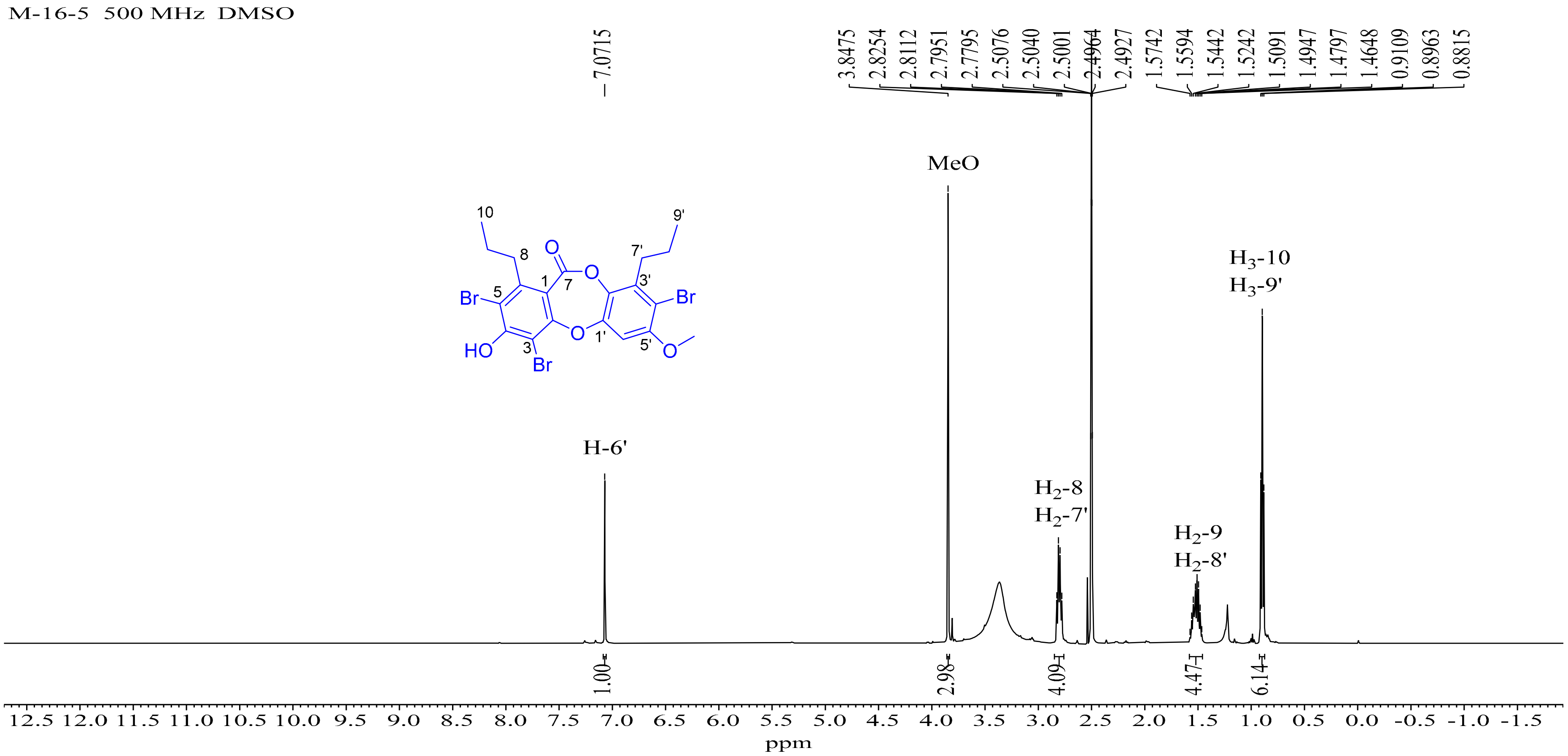

Figure S73. ^{1}H-NMR spectrum of **10** in DMSO-d_6 (500 MHz)

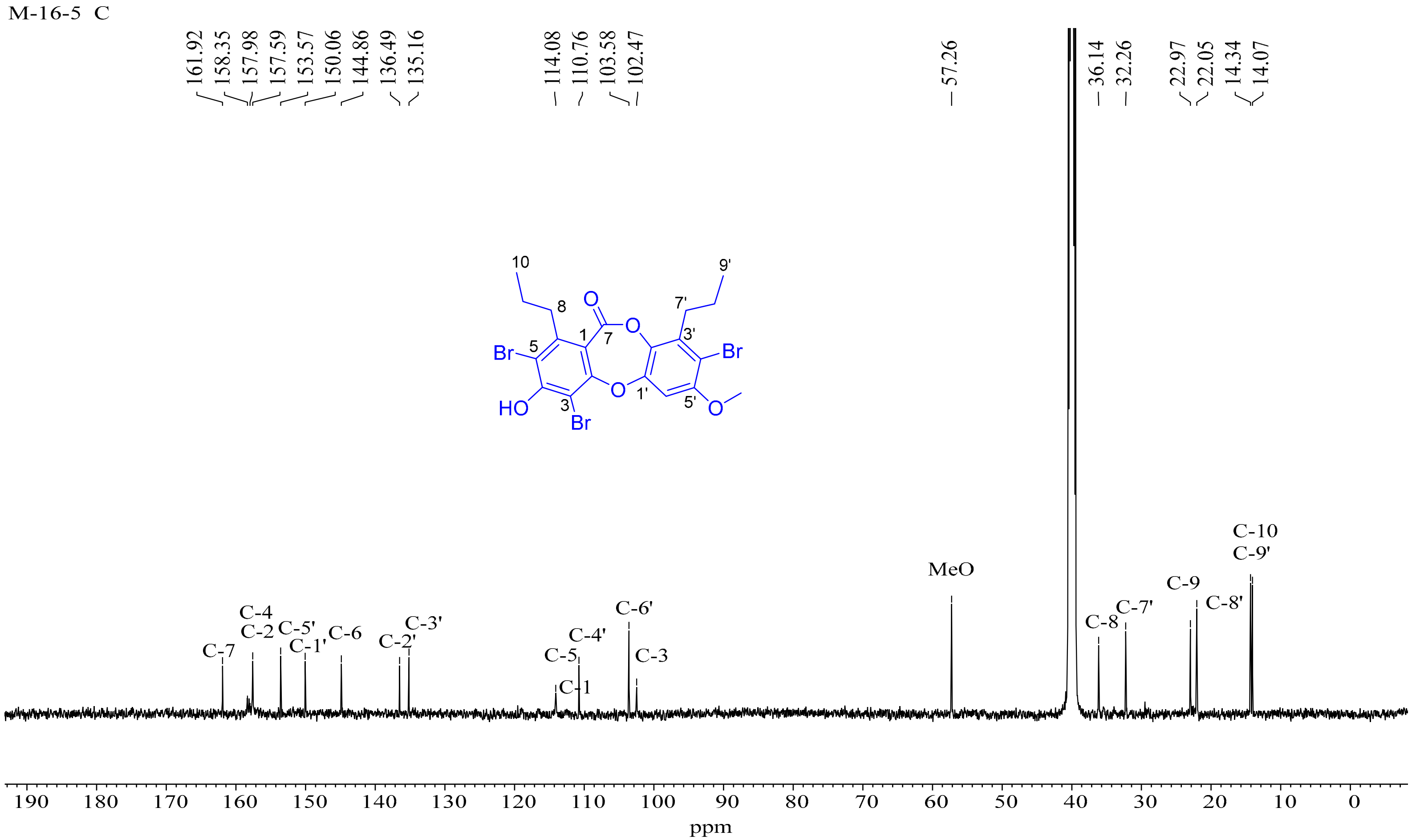

Figure S74. ^{13}C-NMR spectrum of **10** in DMSO-d_6 (125 MHz)

M-16-5 HSQC

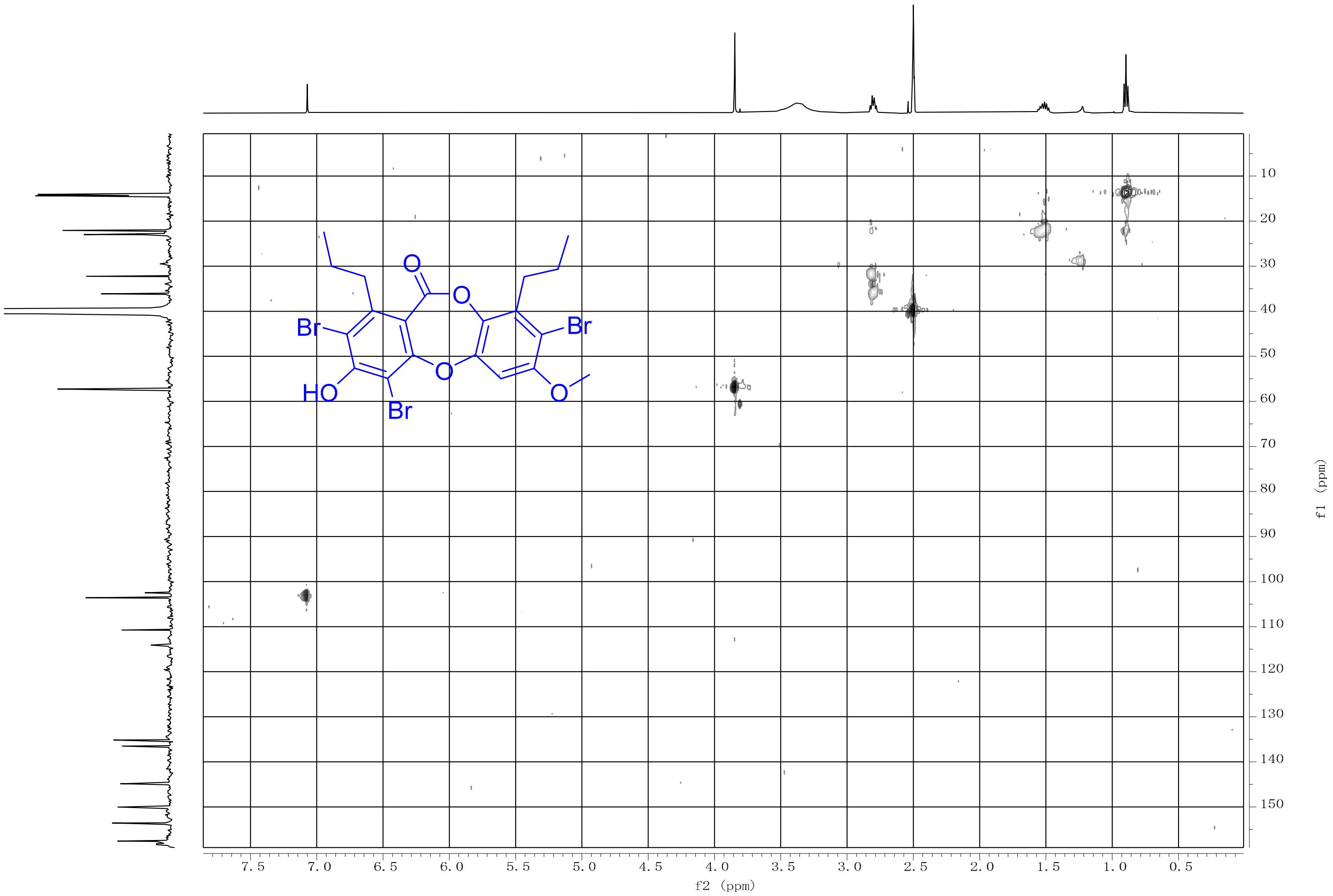

Figure S75. HSQC spectrum of **10** in DMSO-d_6

M-16-5 COSY

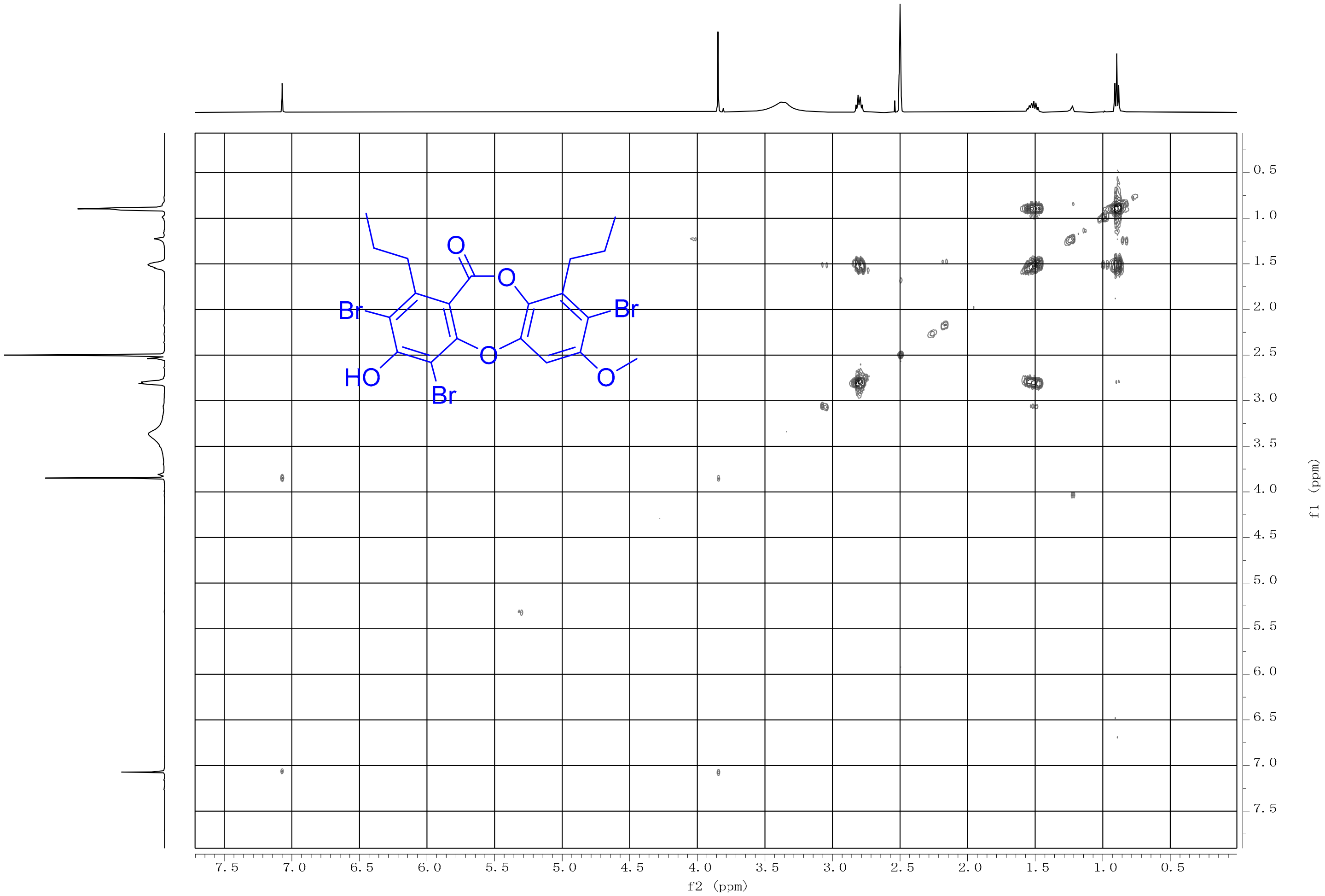

Figure S76. ^{1}H-^{1}H COSY spectrum of **10** in DMSO-d_6

M-16-5 HMBC

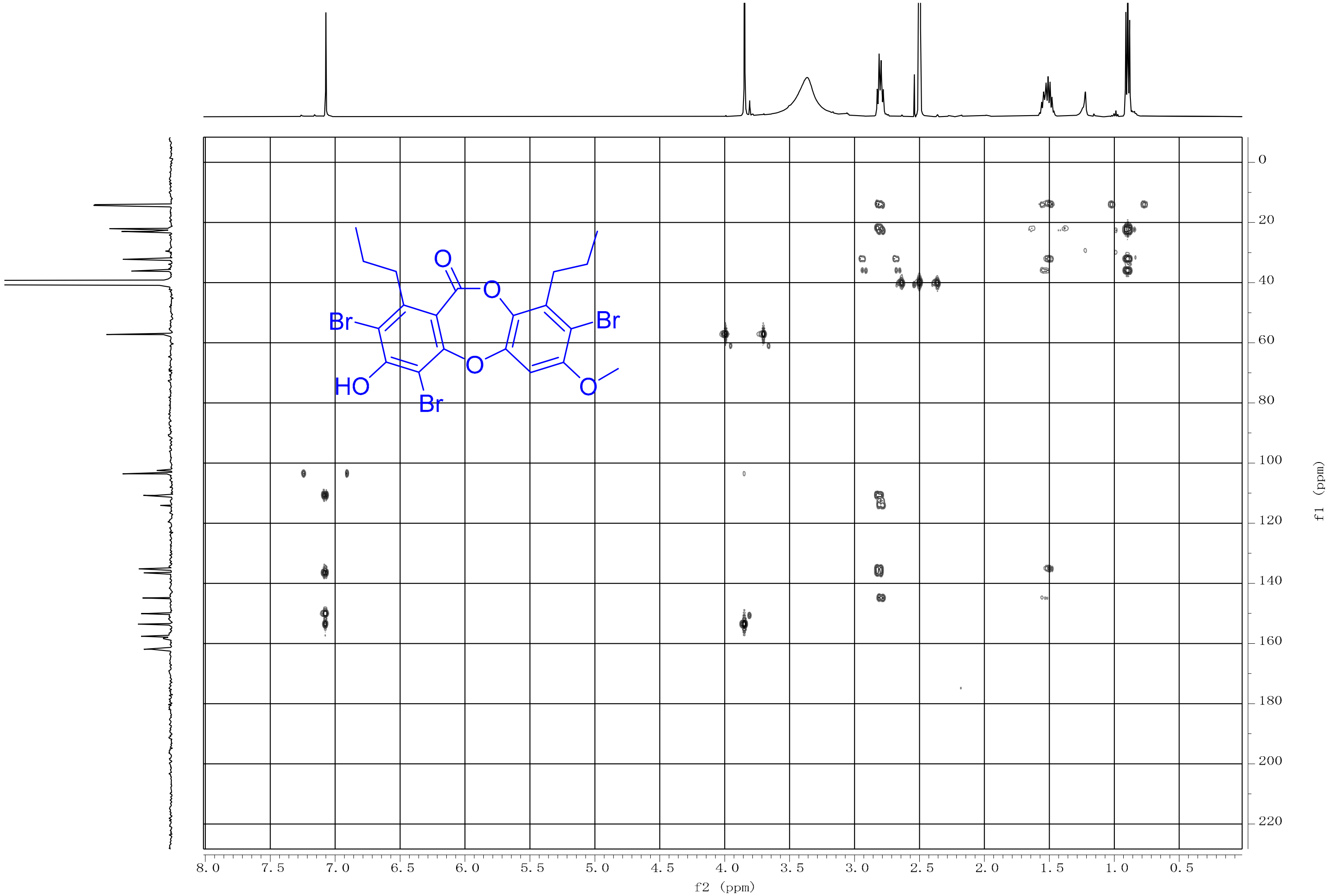

Figure S77. HMBC spectrum of **10** in DMSO-d_6

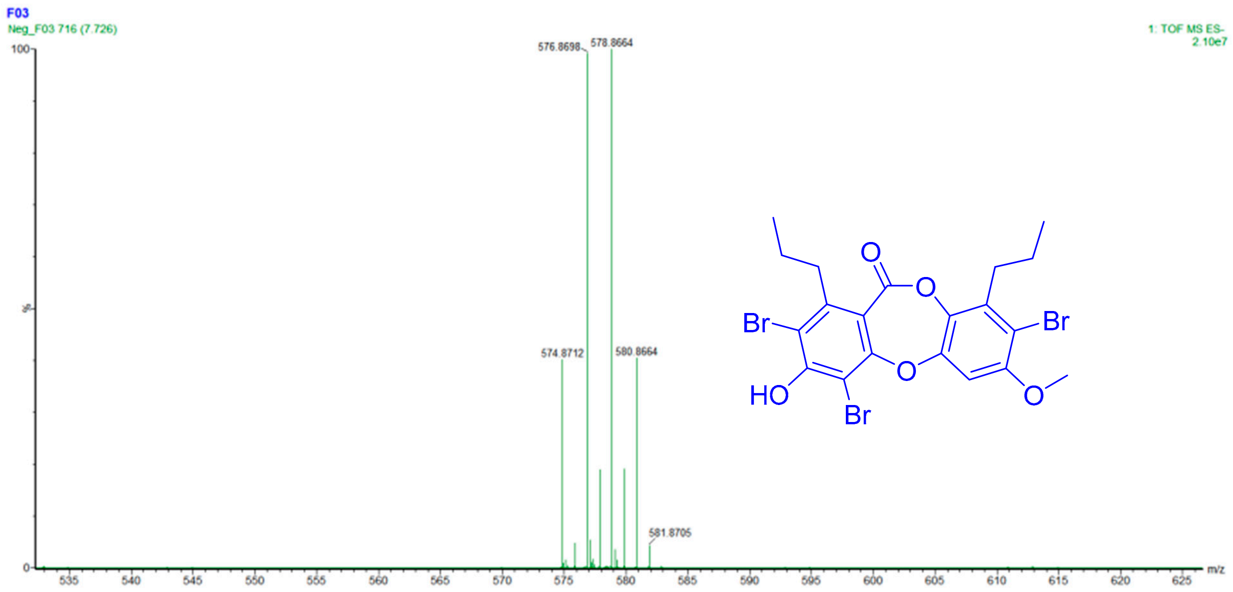

Single Mass Analysis
Tolerance = 5.0 mDa / DBE: min = -1.5, max = 50.0
Element prediction: Off
Monoisotopic Mass, Even Electron Ions
510 formula(e) evaluated with 7 results within limits (up to 50 closest results for each mass)

Elements Used:
C: 0-500 H: 0-1000 O: 0-200 Br: 0-8

Mass	Calc. Mass	mDa	PPM	DBE	Formula	C	H	O	Br
574.8712	574.8704	0.8	1.4	10.5	C20 H18 O5 Br3	20	18	5	3

Figure S78. HRESIMS spectrum of **10**

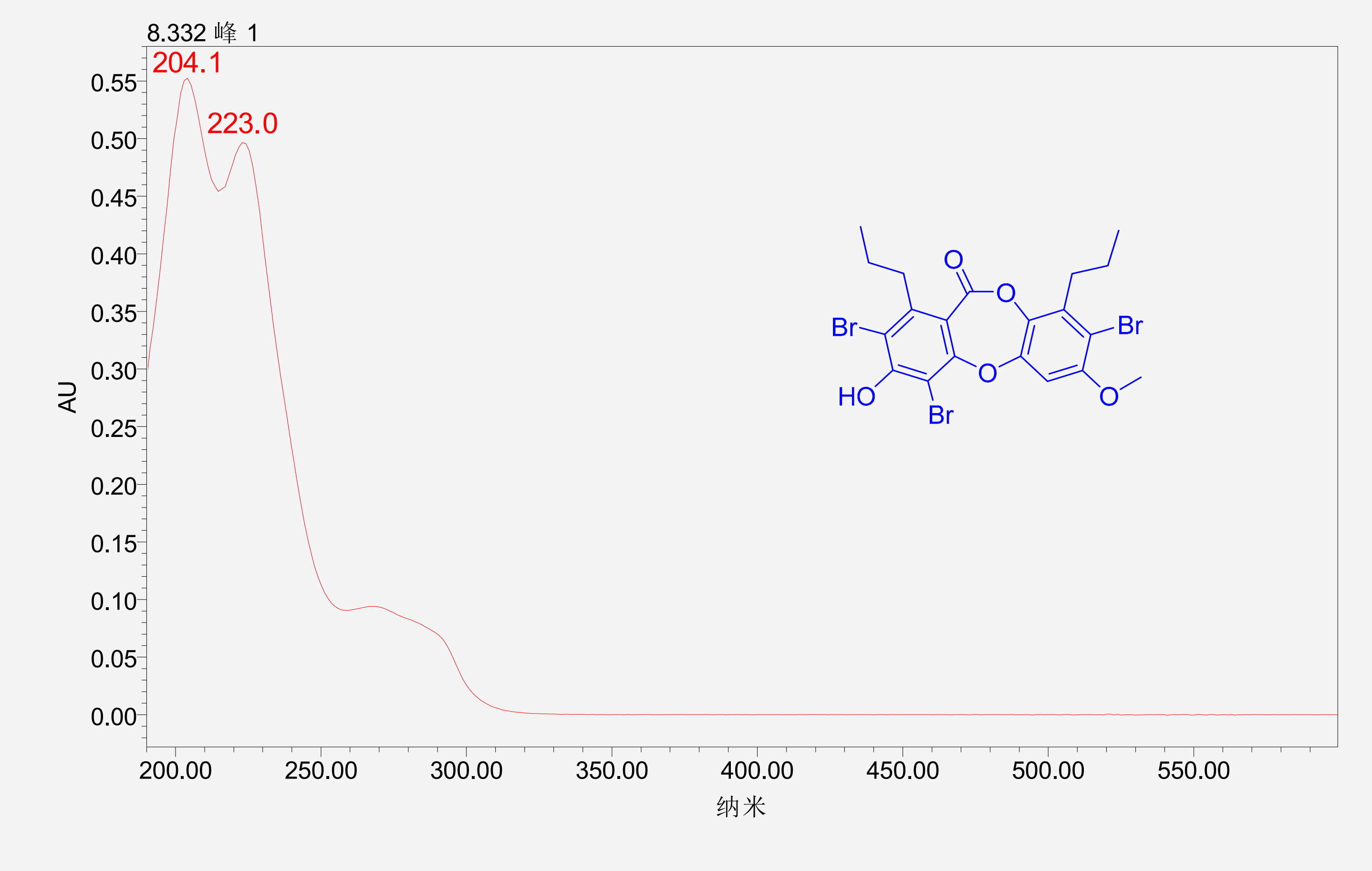

Figure S79. UV spectrum of **10**

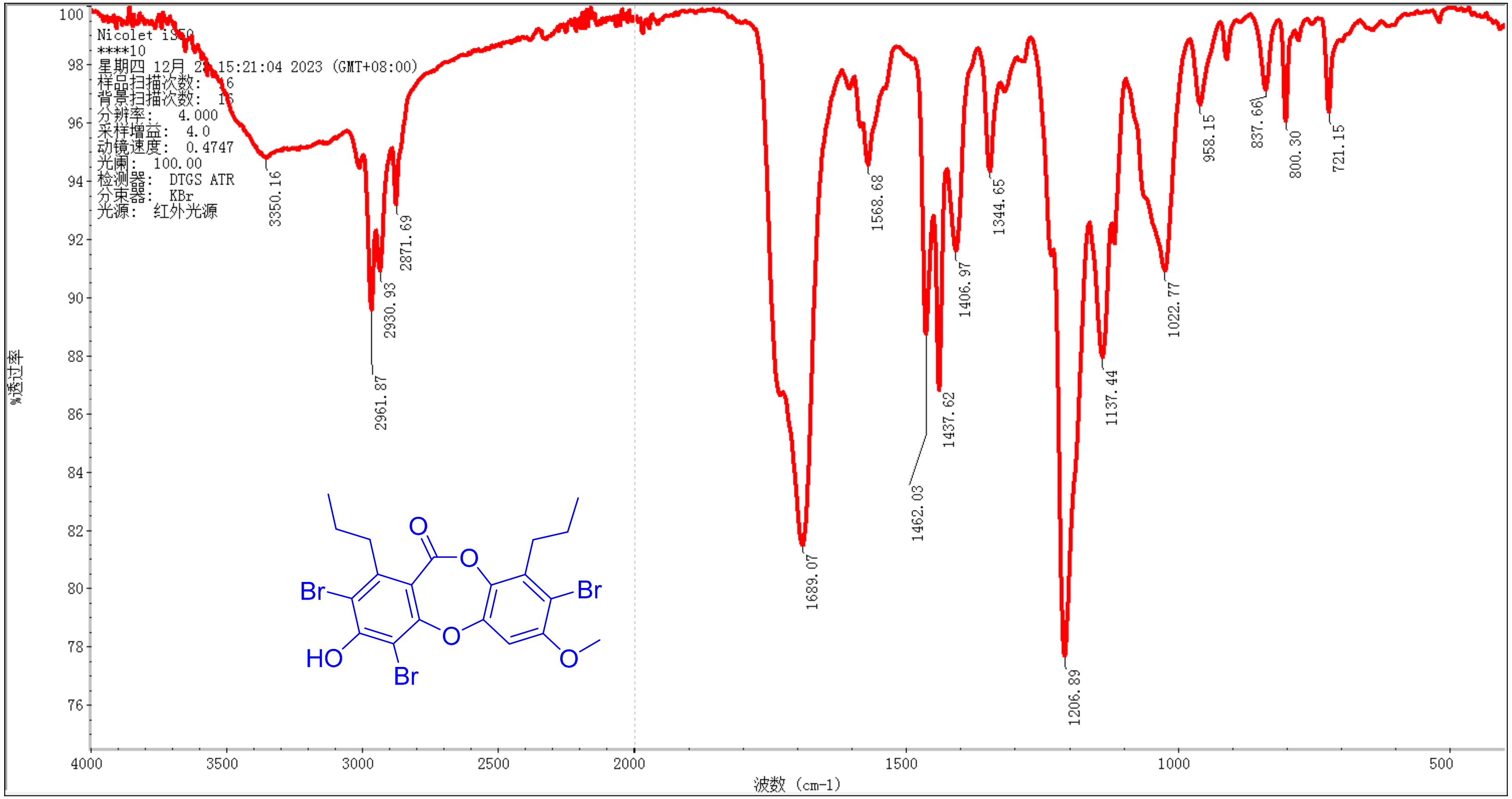

Figure S80. IR spectrum of **10**

Table S11. ^{1}H and ^{13}C NMR data and key COSY and HMBC correlations of 11

No.	δ_H	δ_C	COSY	HMBC
1		112.2, C		
2		158.9, C		
3		102.2, C		
4		151.1, C		
5		116.2, C		
6		145.7, C		
7		161.6, C		
8	2.72, t (8.0)	36.9, CH_2	H_2-9	C-1, C-5, C-6, C-9, C-10
9	1.56, m	23.0, CH_2	H_2-8, H_3-10	C-6, C-8, C-10
10	0.87, t (7.2)	14.5, CH_3	H_2-9	C-8, C-9
1′		149.1, C		
2′		141.9, C		
3′		134.0, C		
4′		117.5, C		
5′		152.0, C		
6′		108.3, C		
7′	2.83, t (7.5)	32.5, CH_2	H_2-8’	C-2', C-3', C-4', C-8', C-9'
8′	1.54, m	22.0, CH_2	H_2-7’, H_3-9’	C-3', C-7', C-9'
9′	0.92, t (7.2)	14.0, CH_3	H_2-8’	C-7', C-8'
MeO	3.78, s	60.8, CH_3		C-5’

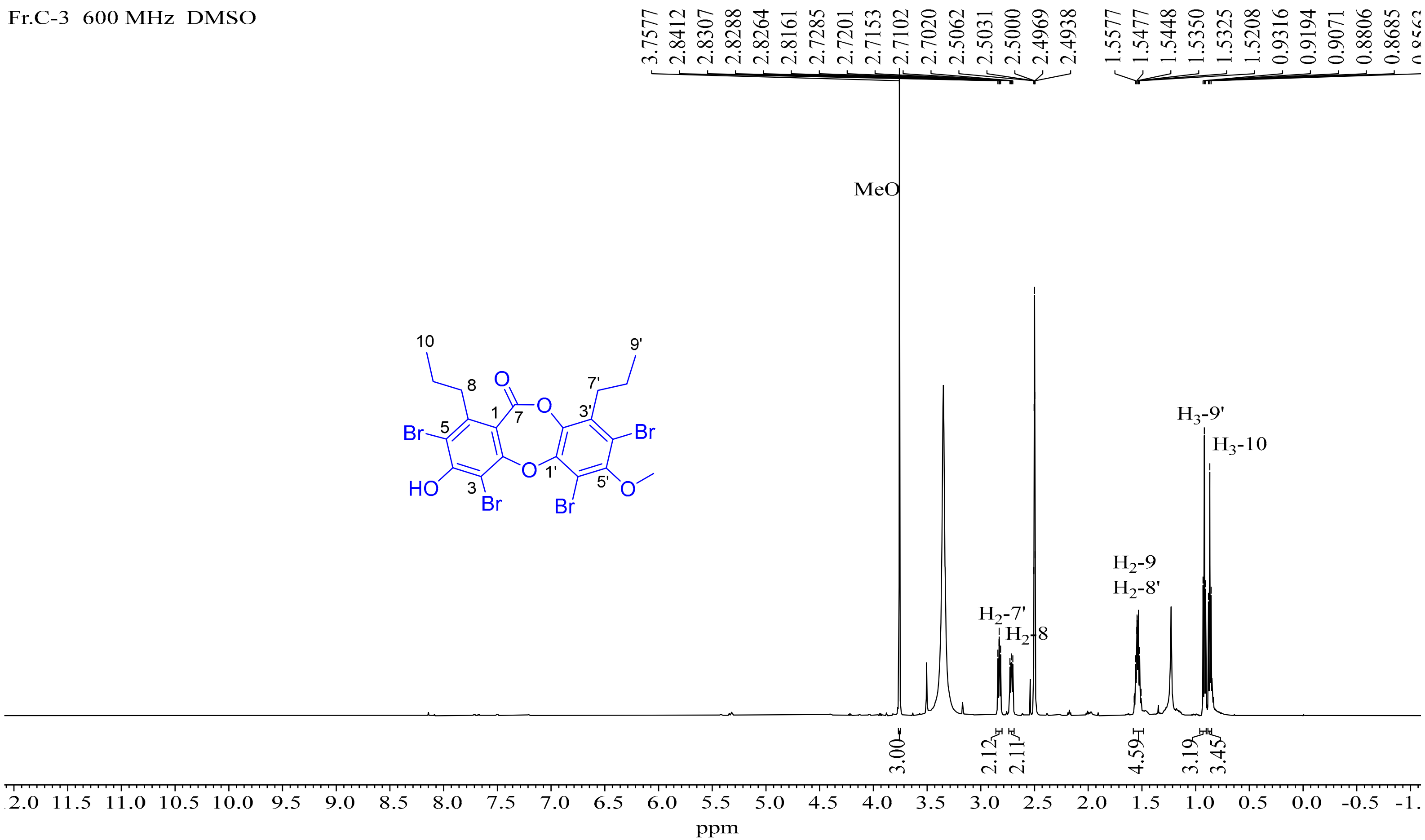

Figure S81. ^{1}H-NMR spectrum of **11** in DMSO-d_6 (600 MHz)

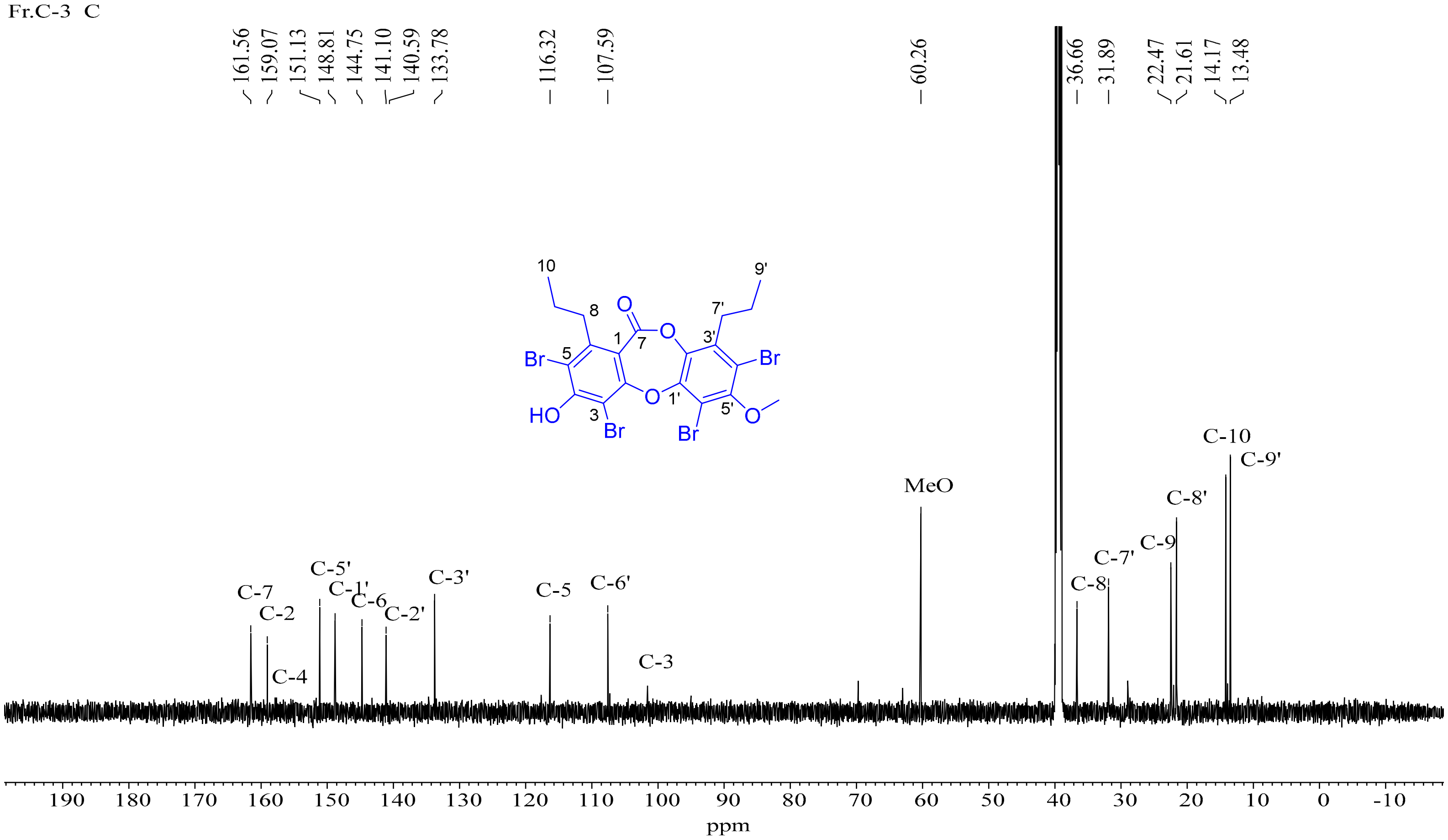

Figure S82. ^{13}C-NMR spectrum of **11** in DMSO-d_6 (150 MHz)

Fr.C-3 HSQC

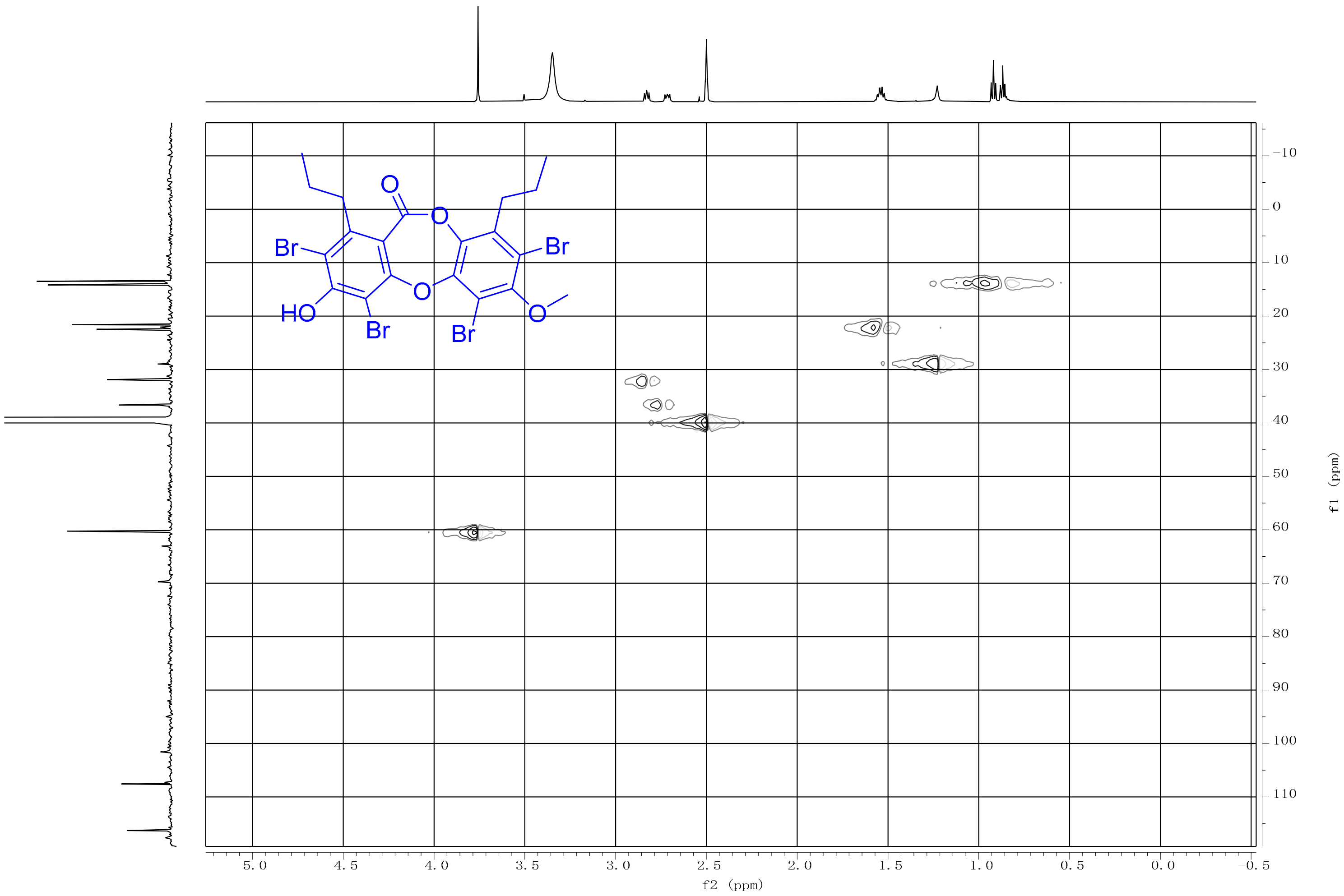

Figure S83. HSQC spectrum of **11** in DMSO-d_6

Fr.C-3 COSY

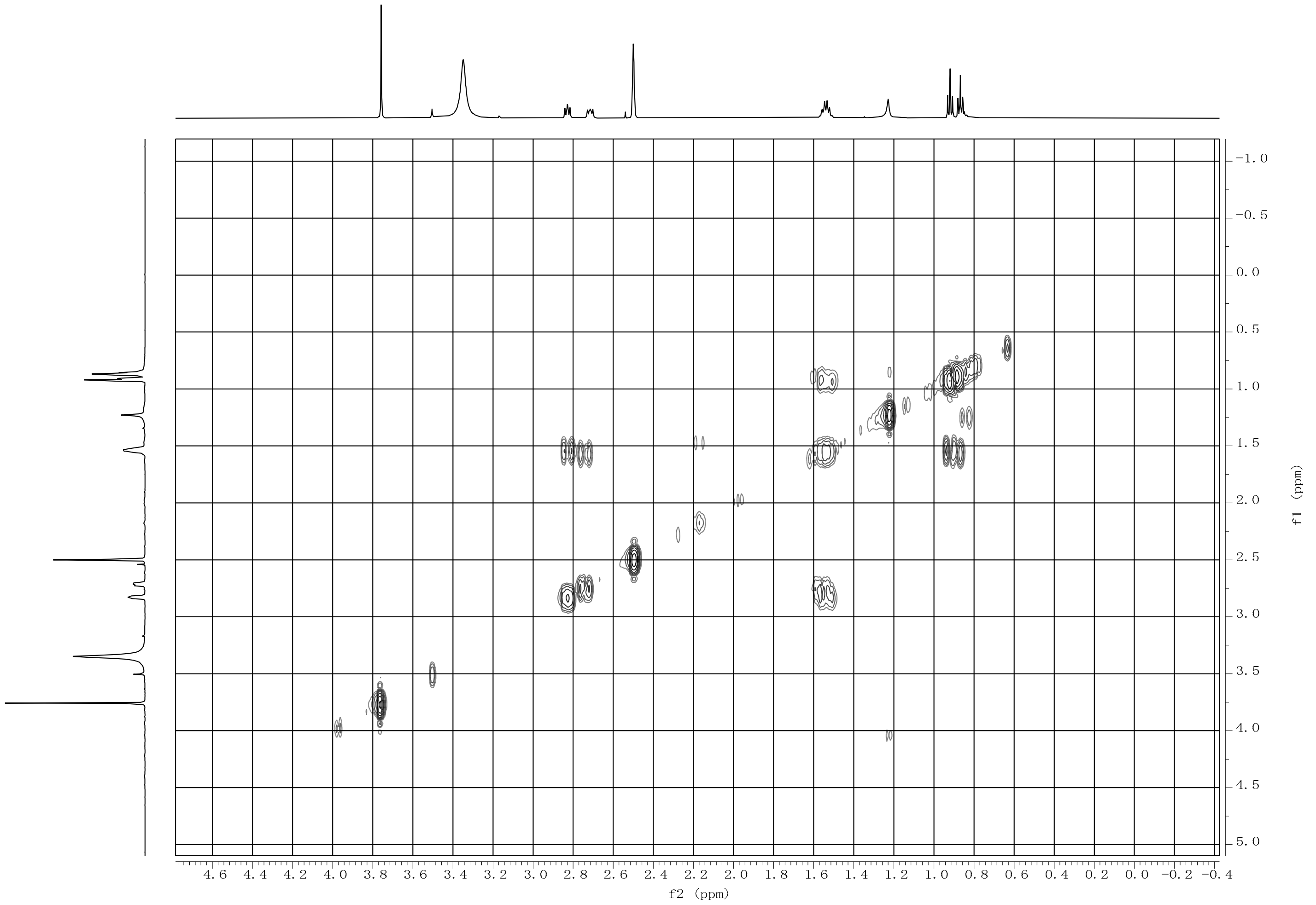

Figure S84. ^1H-^1H COSY spectrum of **11** in DMSO-d_6

Fr.C-3 HMBC

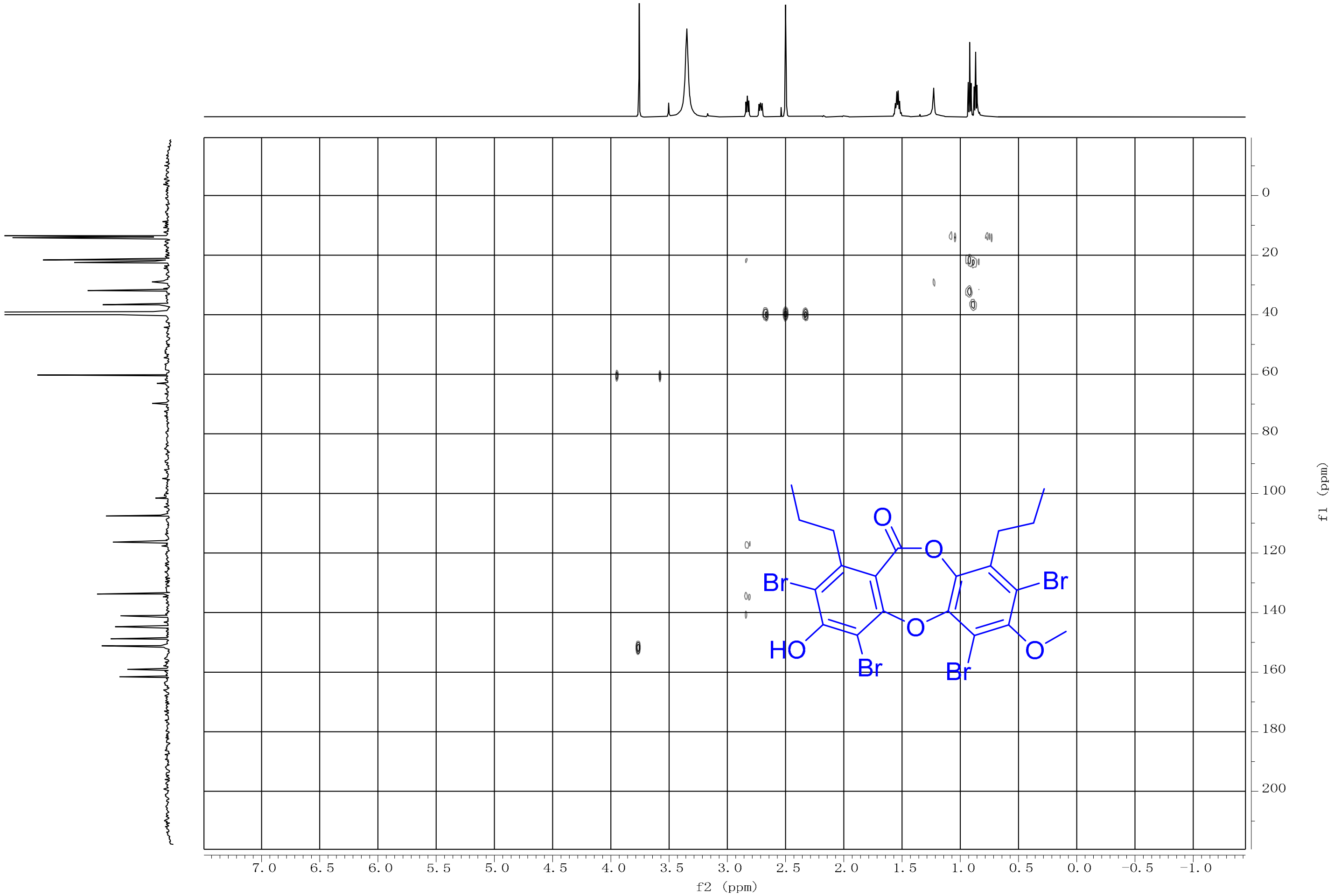

Figure S85. HMBC spectrum of **11** in DMSO-d_6

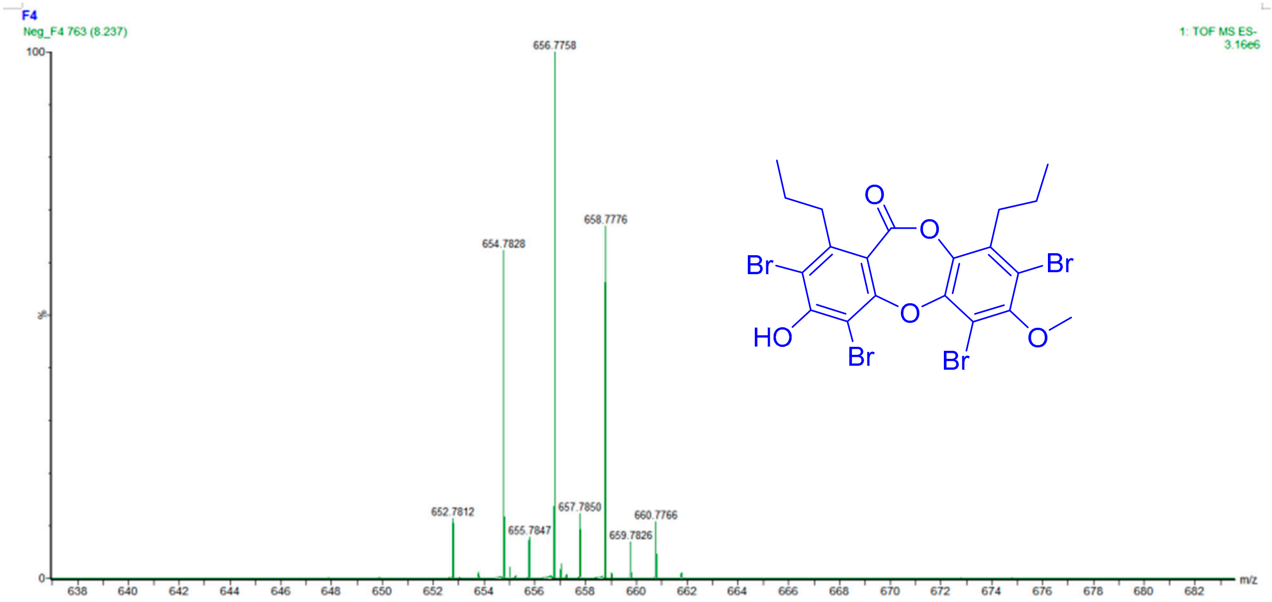

Single Mass Analysis
Tolerance = 5.0 mDa / DBE: min = -1.5, max = 50.0
Element prediction: Off
Monoisotopic Mass, Even Electron Ions
694 formula(e) evaluated with 5 results within limits (up to 50 closest results for each mass)
Elements Used:
C: 0-500 H: 0-1000 O: 0-200 Br: 0-8

Mass	Calc. Mass	mDa	PPM	DBE	Formula	C	H	O	Br
652.7812	652.7810	0.2	0.3	10.5	C20 H17 O5 Br4	20	17	5	4

Figure S86. HRESIMS spectrum of **11**

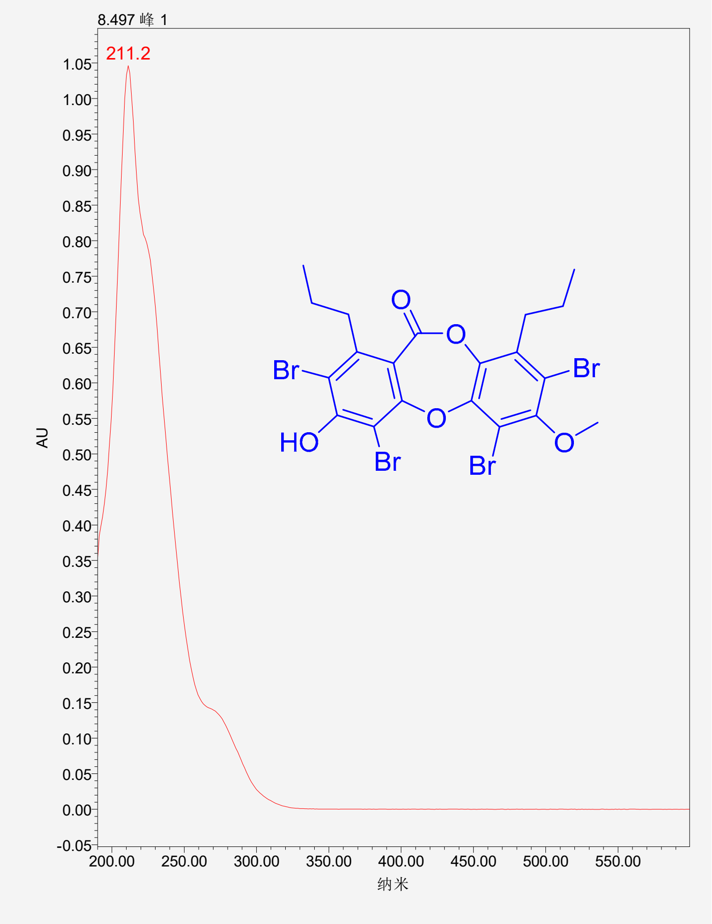

Figure S87. UV spectrum of **11**

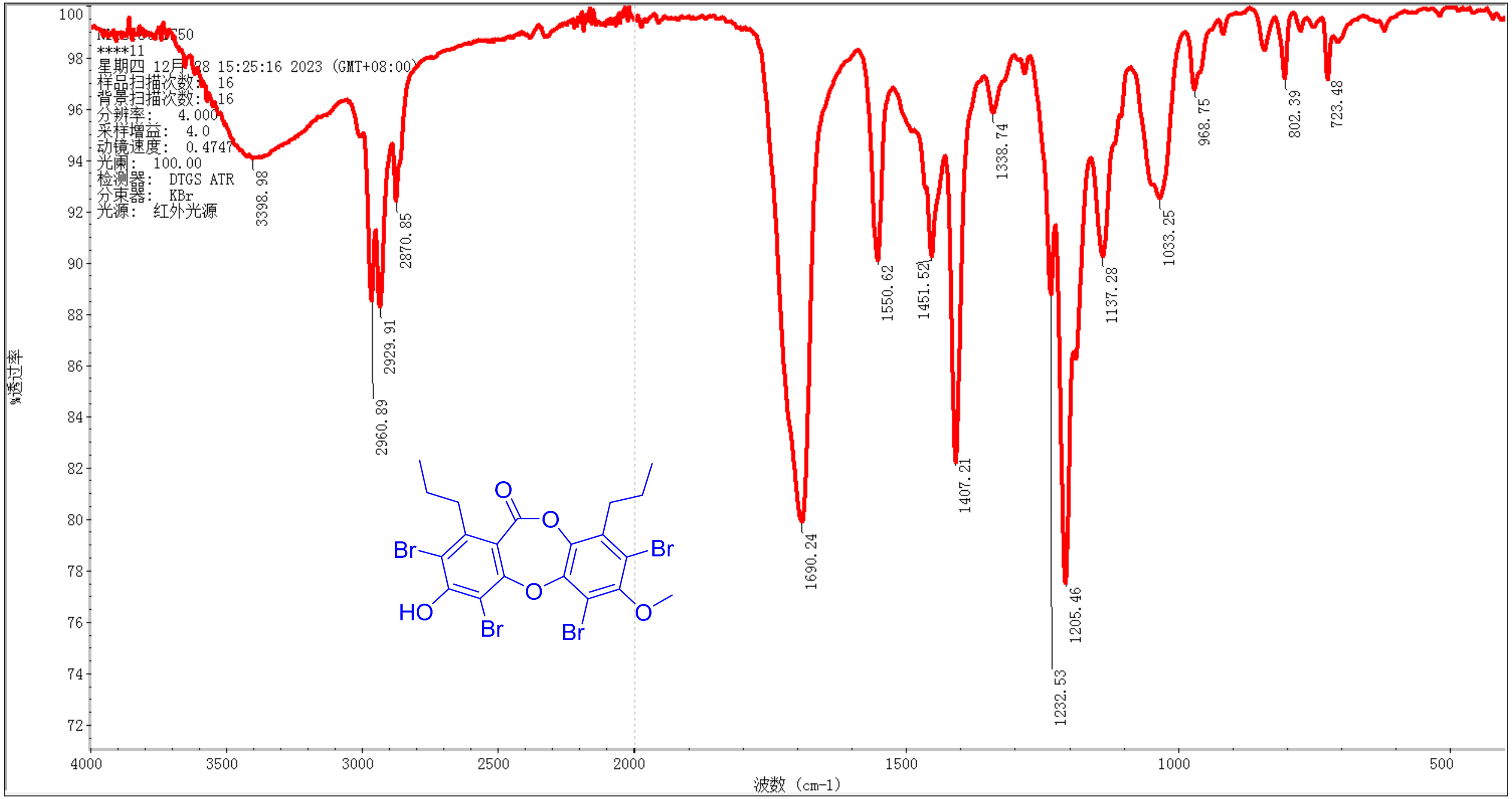

Figure S88. IR spectrum of **11**

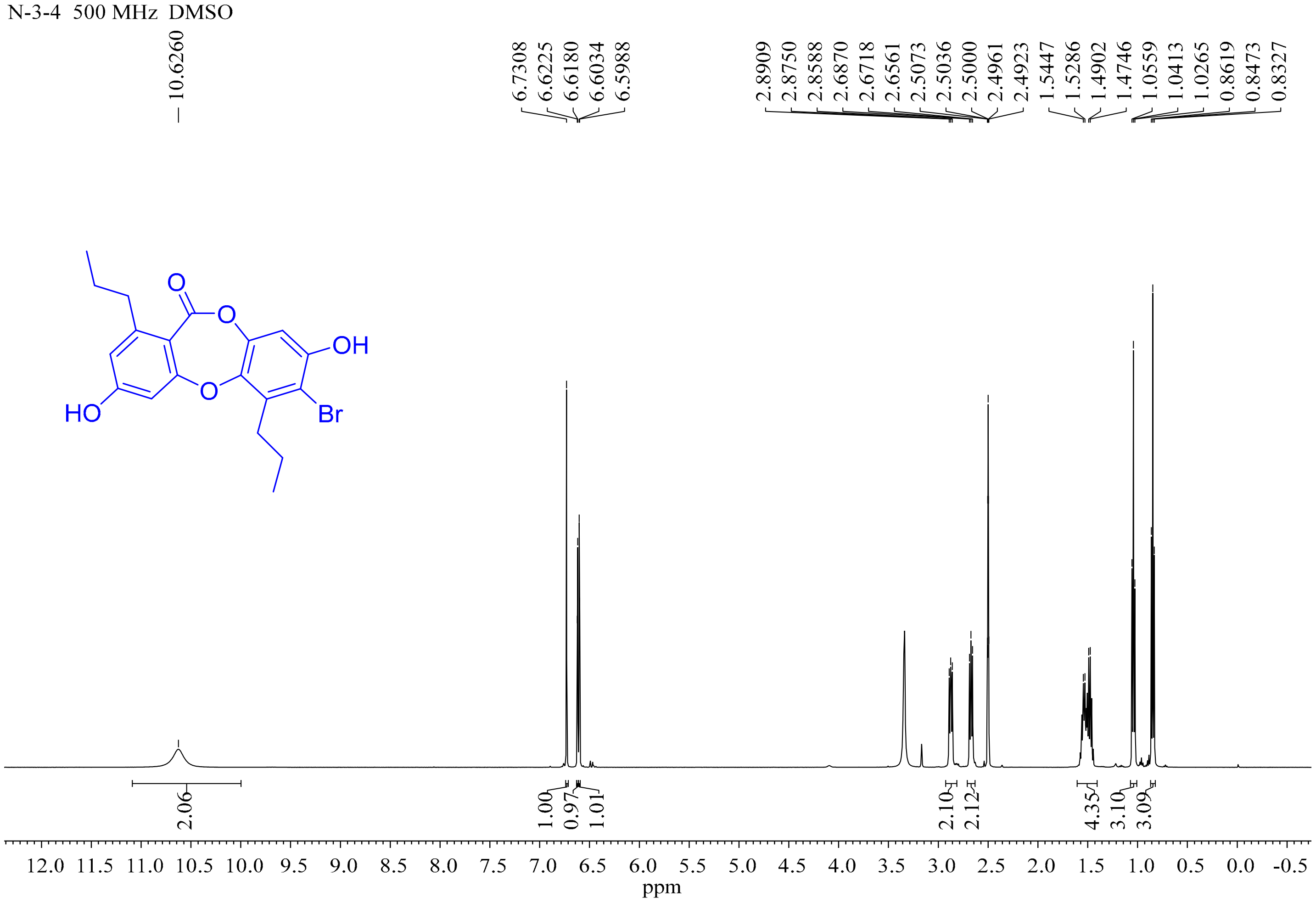

Figure S89. ^{1}H-NMR spectrum of **12** in DMSO-d_6 (500 MHz)

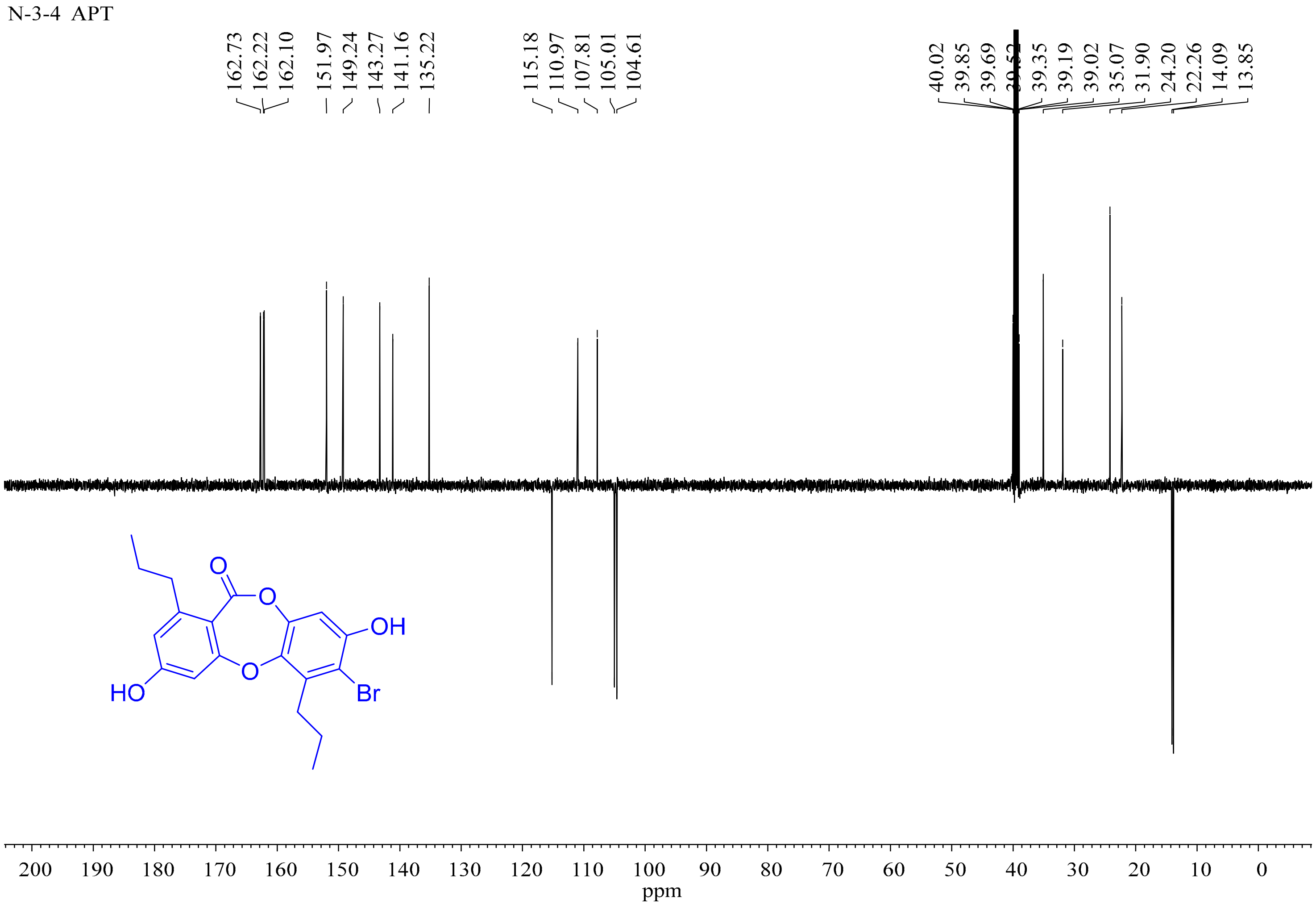

Figure S90. APT spectrum of **12** in DMSO-d_6 (125 MHz)

N-3-4 HSQC

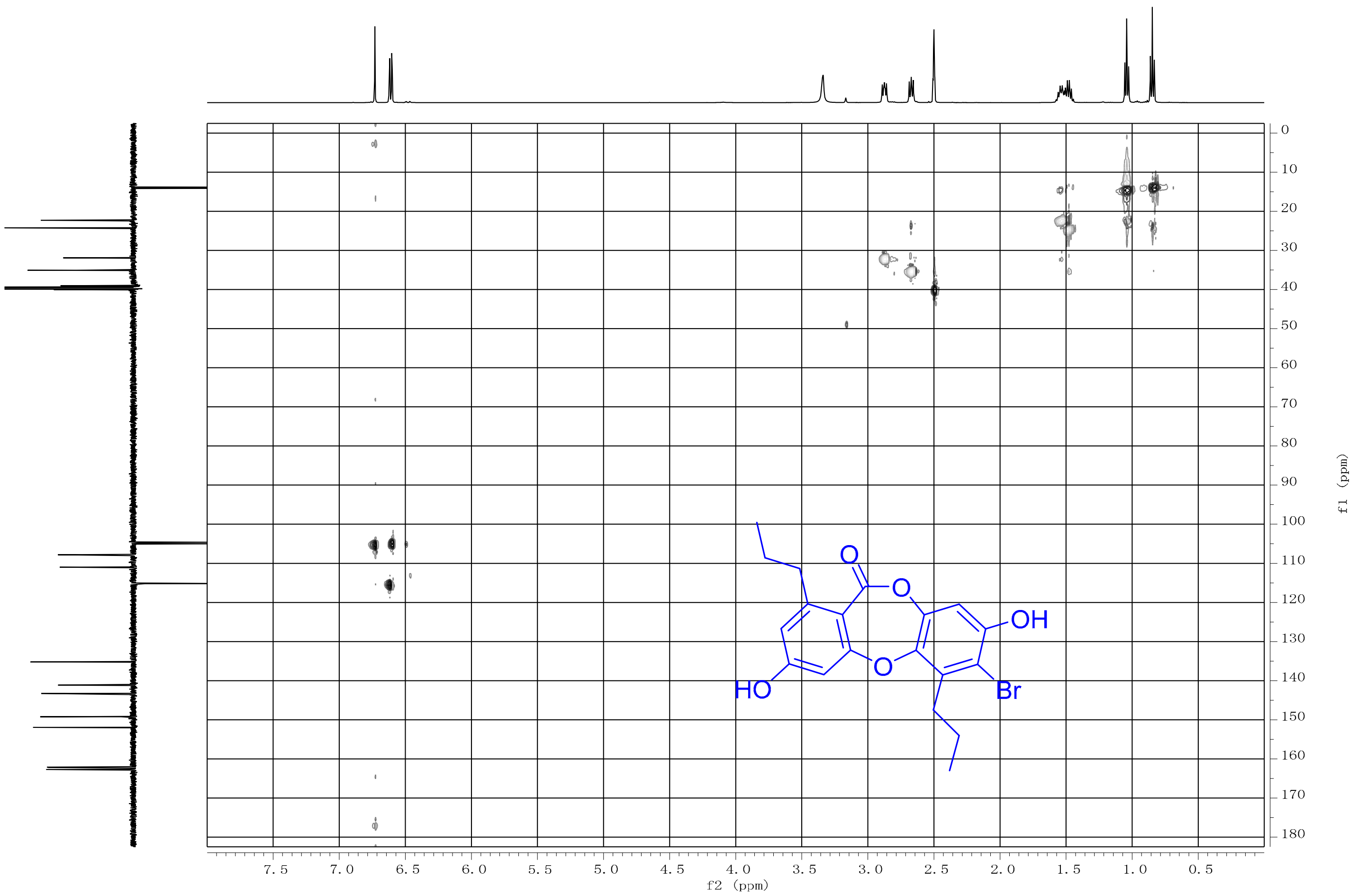

Figure S91. HSQC spectrum of **12** in DMSO-d_6

N-3-4 COSY

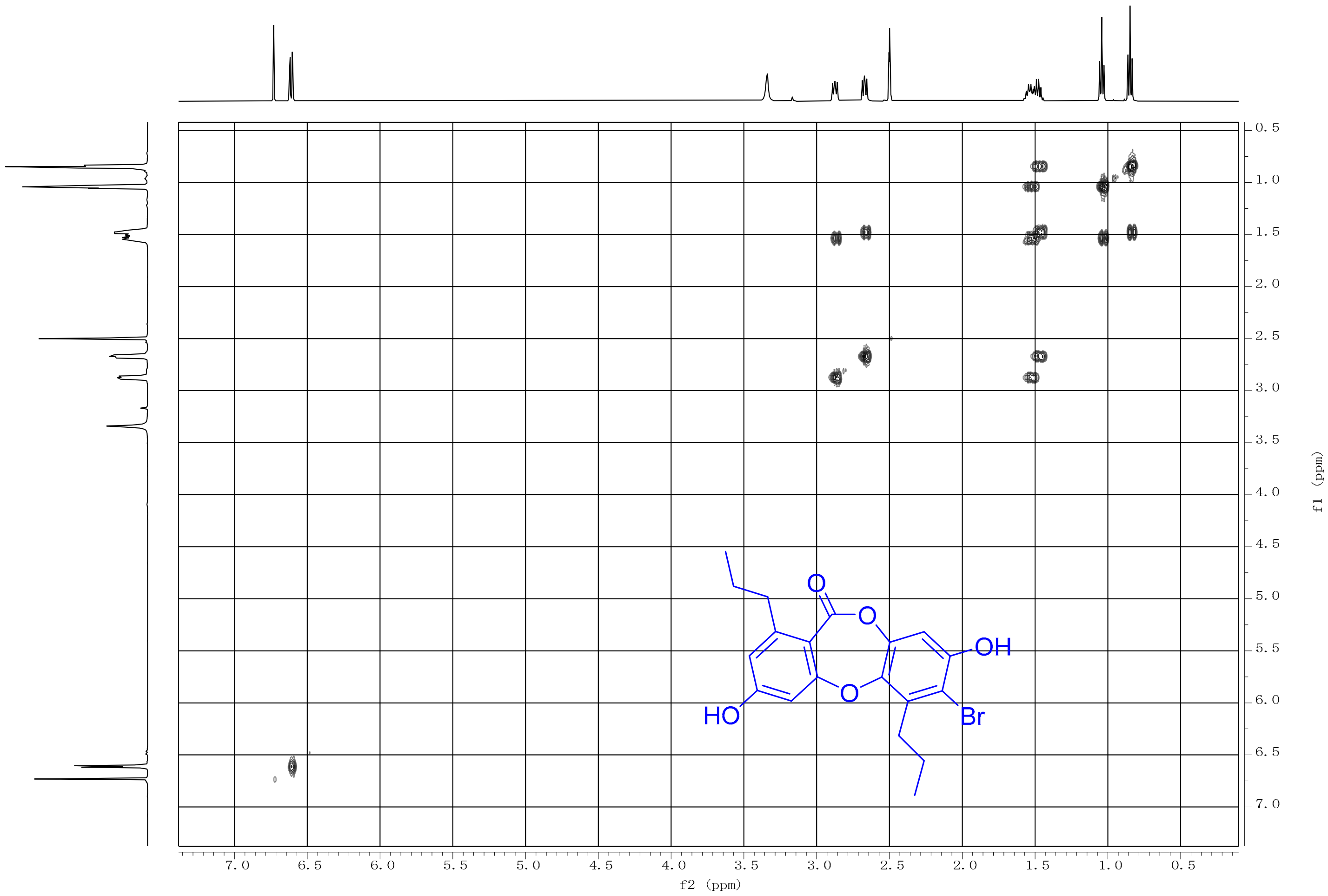

Figure S92. ^{1}H-^{1}H COSY spectrum of **12** in DMSO-d_6

N-3-4 HMBC

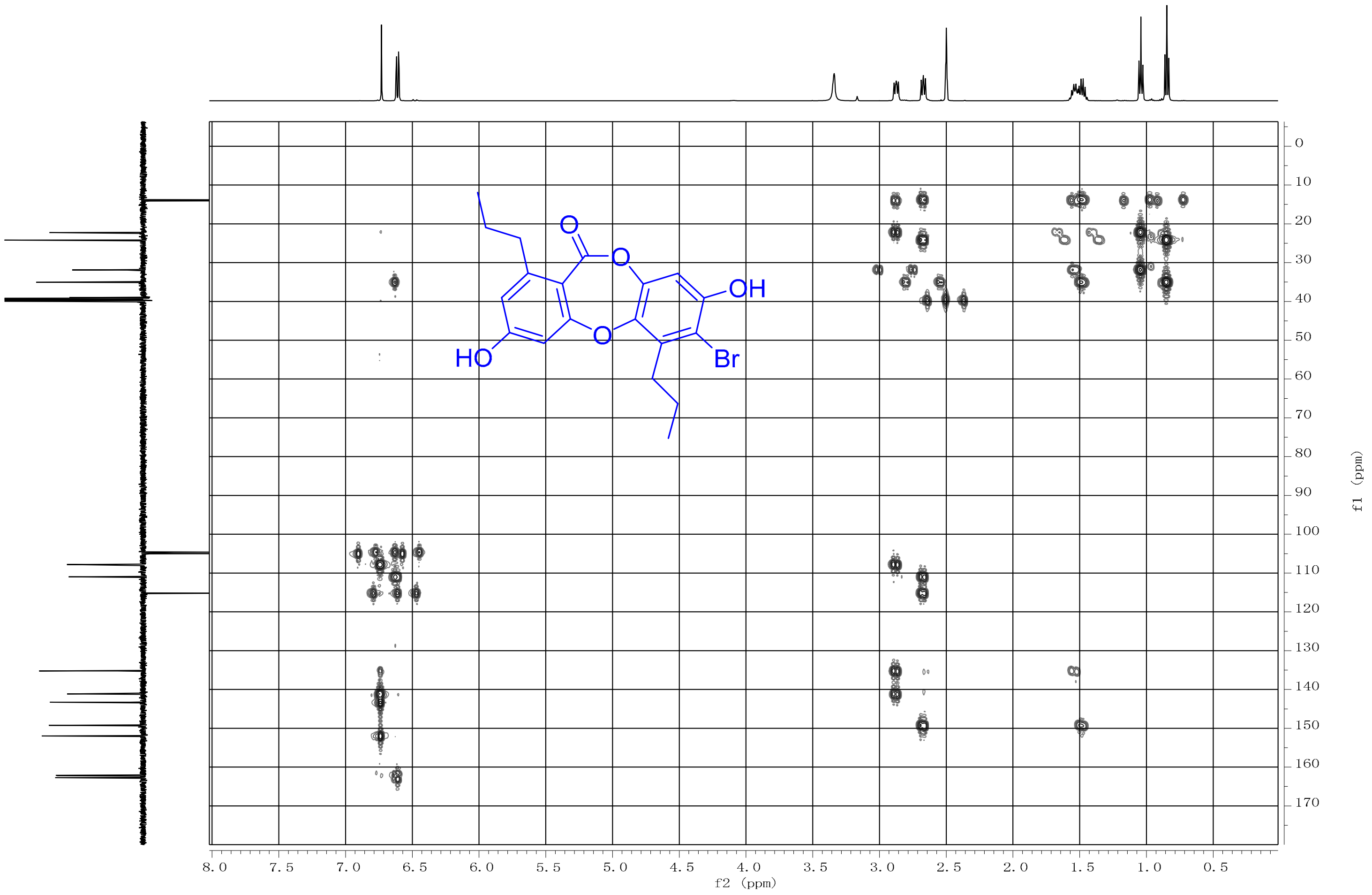

Figure S93. HMBC spectrum of **12** in DMSO-d_6

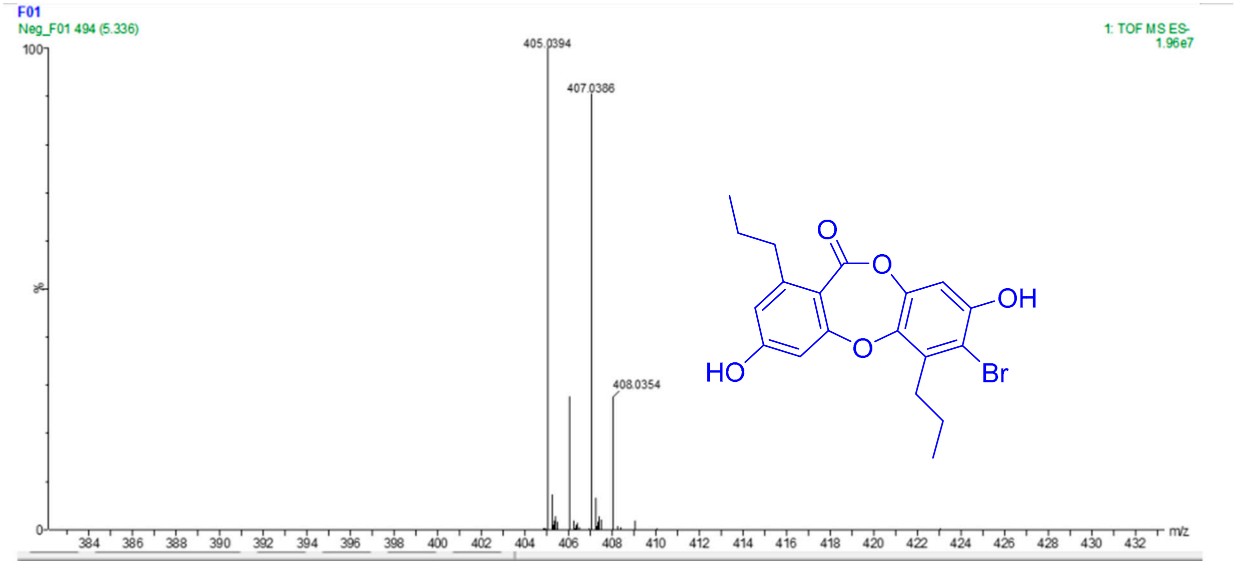

Single Mass Analysis

Tolerance = 5.0 mDa / DBE: min = -1.5, max = 50.0

Element prediction: Off

Monoisotopic Mass, Even Electron Ions

213 formula(e) evaluated with 5 results within limits (up to 50 closest results for each mass)

Elements Used:

C: 0-500 H: 0-1000 O: 0-200 Br: 0-8

Mass	Calc. Mass	mDa	PPM	DBE	Formula	C	H	O	Br
405.0348	405.0396	-4.8	-11.9	1.5	C12 H22 O10...	12	22	10	1
	405.0338	1.0	2.5	10.5	C19 H18 O5 Br	19	18	5	1
	405.0340	0.8	2.0	30.5	C32 H5 O	32	5	1	

Figure S94. HRESIMS spectrum of **12**

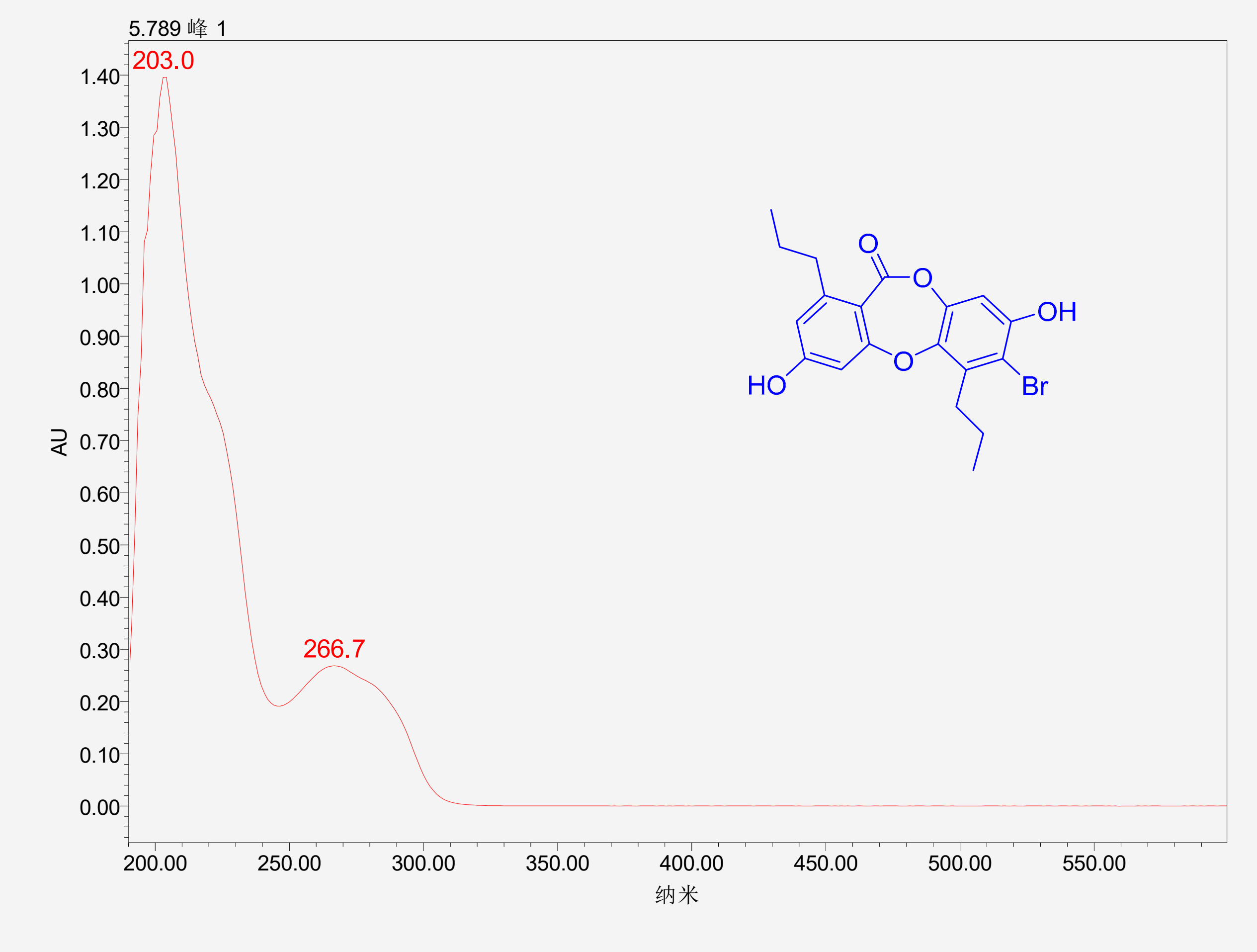

Figure S95. UV spectrum of **12**

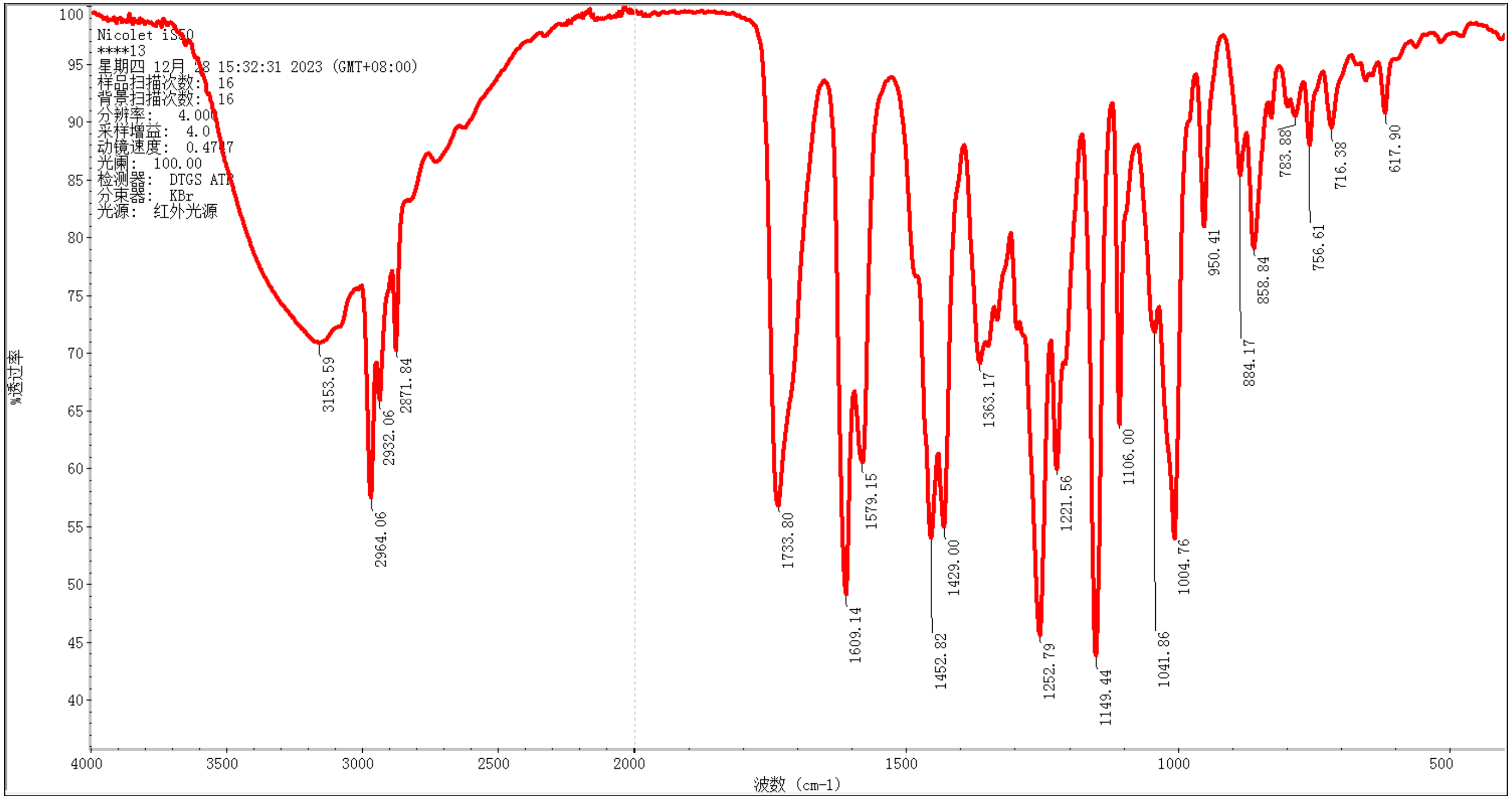

Figure S96. IR spectrum of **12**

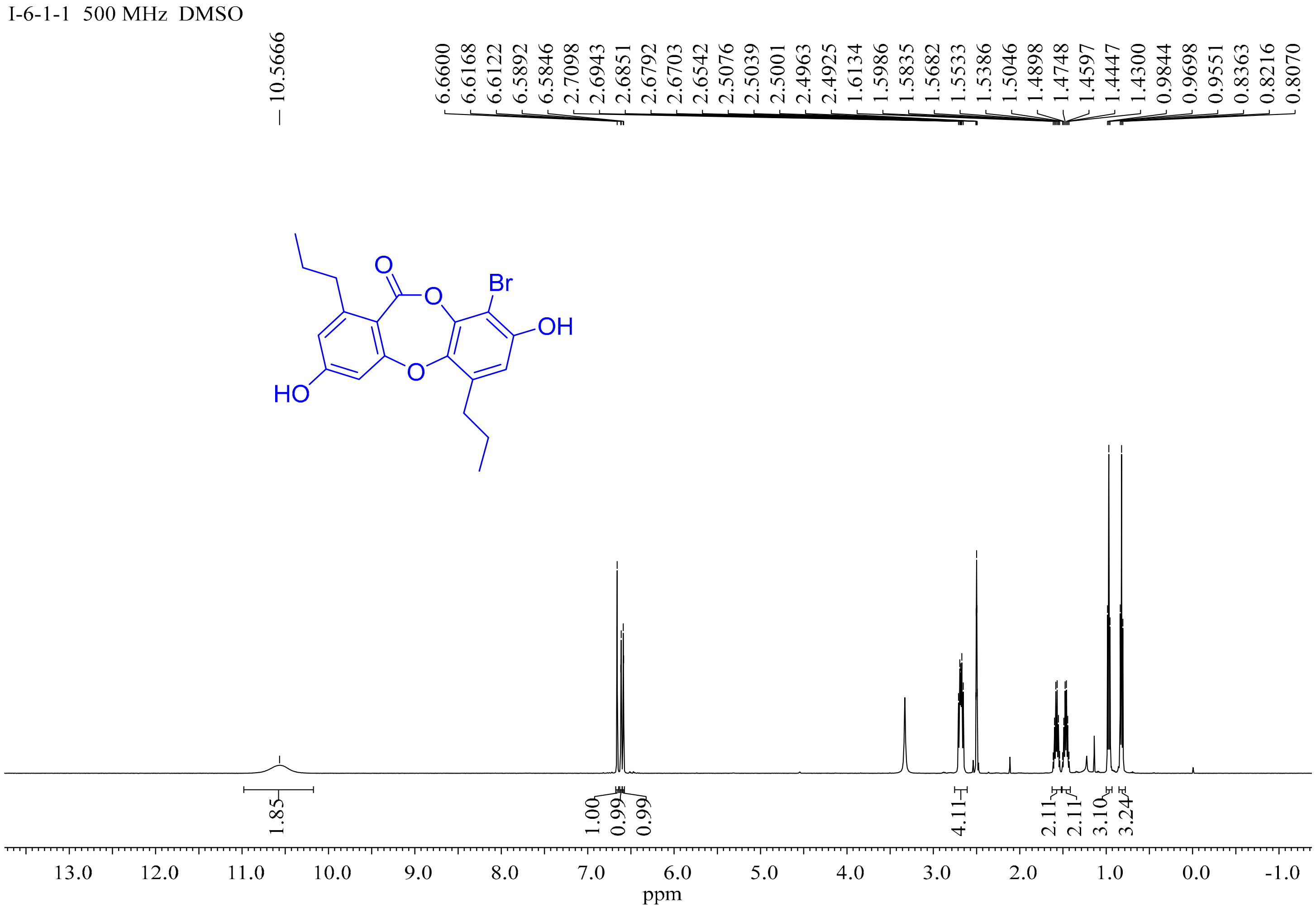

Figure S97. ^{1}H-NMR spectrum of **13** in DMSO-d_6 (500 MHz)

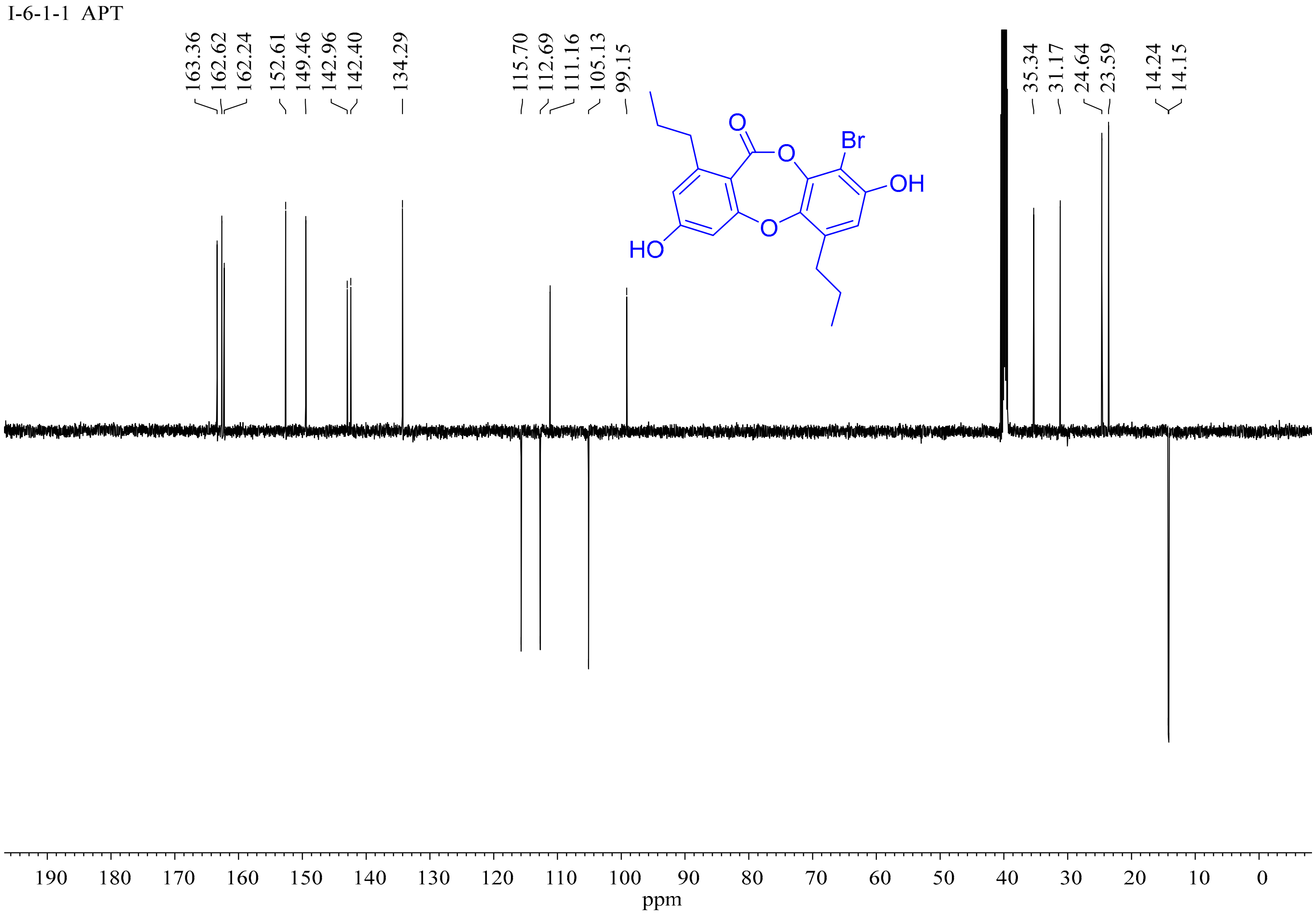

Figure S98. APT spectrum of **13** in DMSO-d_6 (125 MHz)

I-6-1-1 HSQC

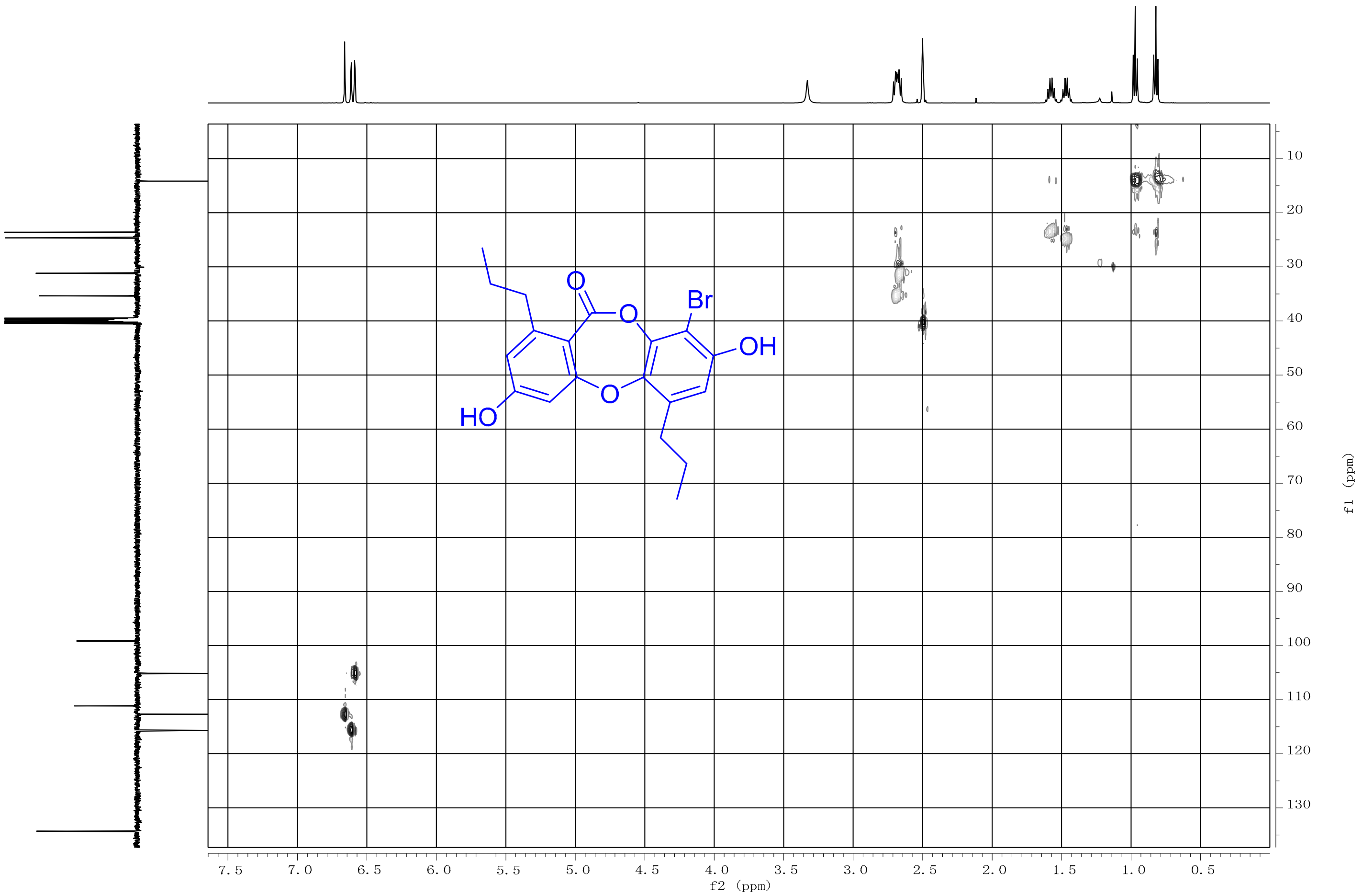

Figure S99. HSQC spectrum of **13** in DMSO-d_6

I-6-1-1 COSY

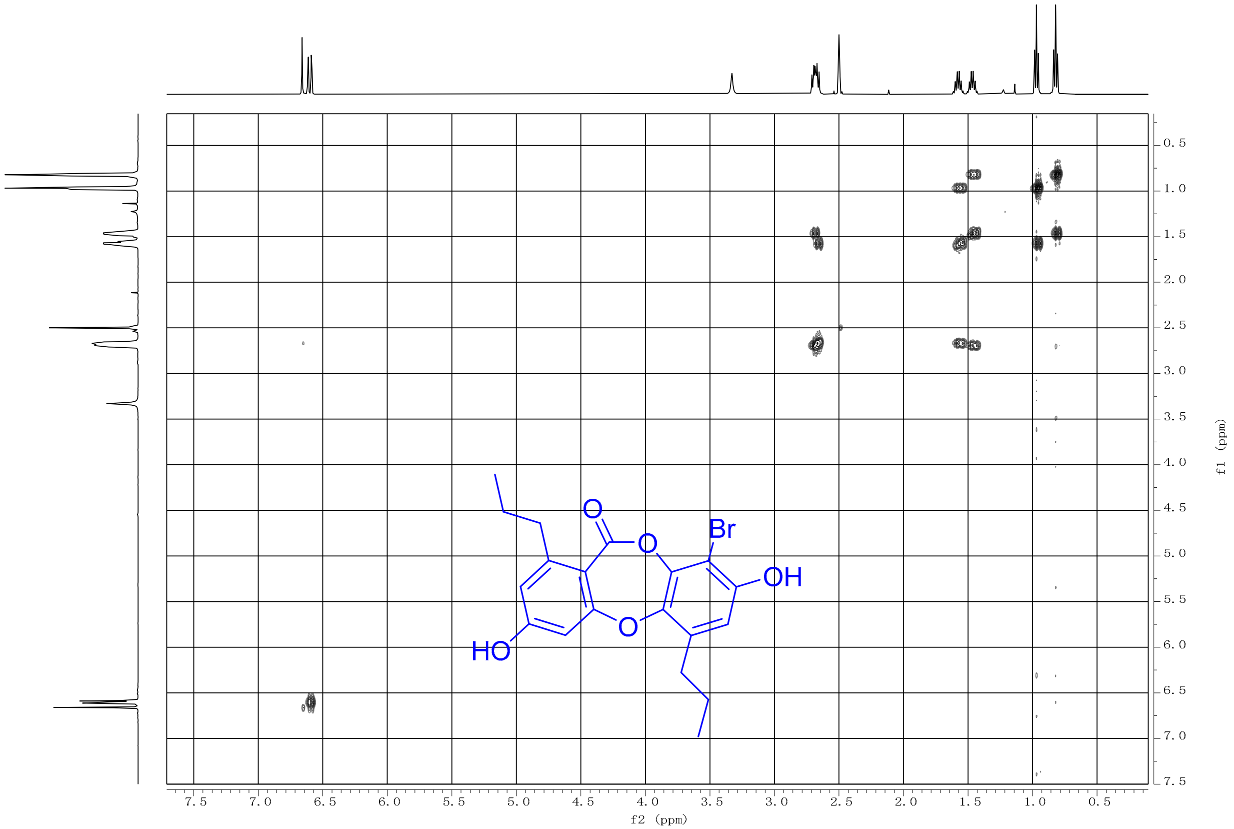

Figure S100. ^1H-^1H COSY spectrum of **13** in DMSO-d_6

I-6-1-1 HMBC

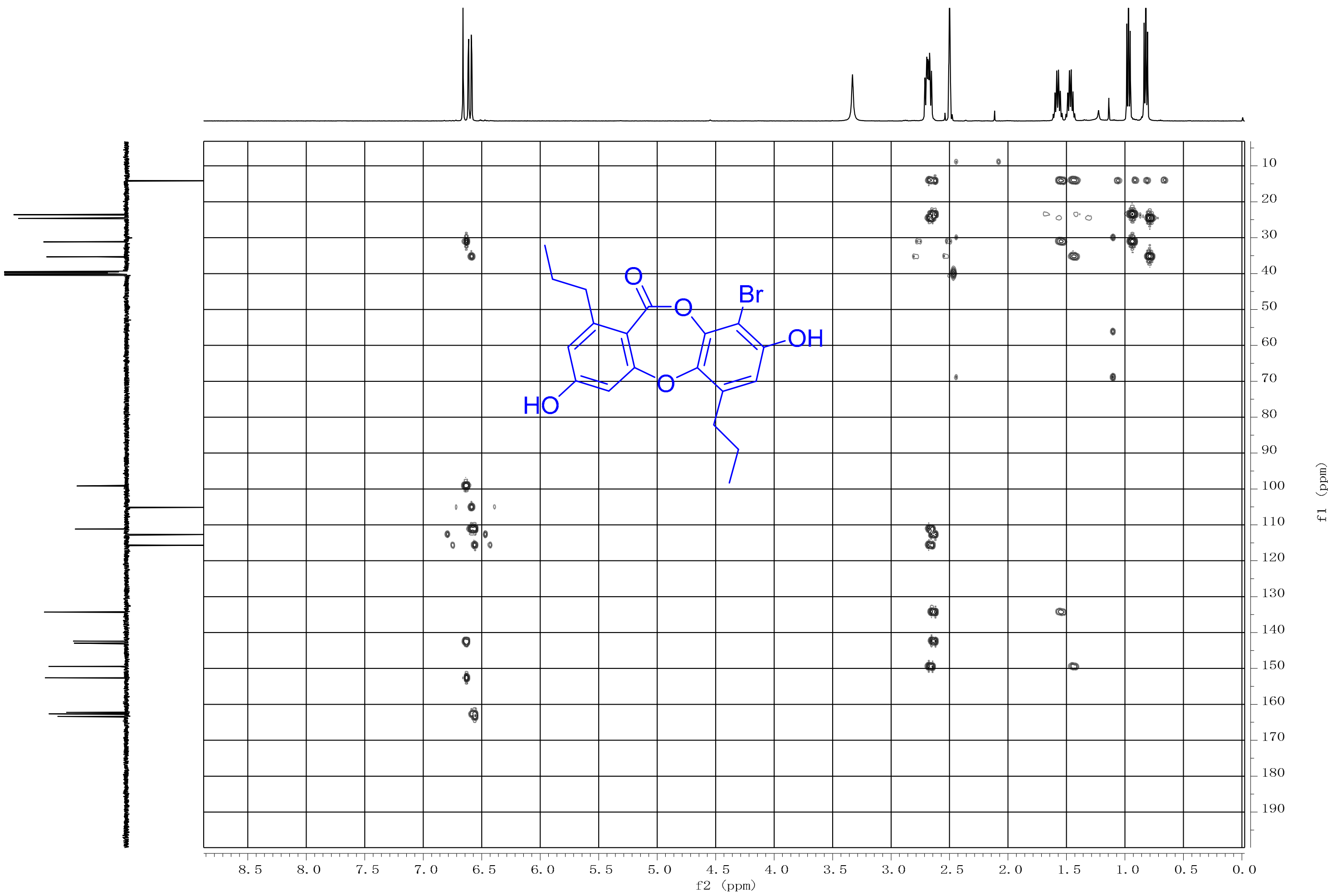

Figure S101. HMBC spectrum of **13** in DMSO-d_6

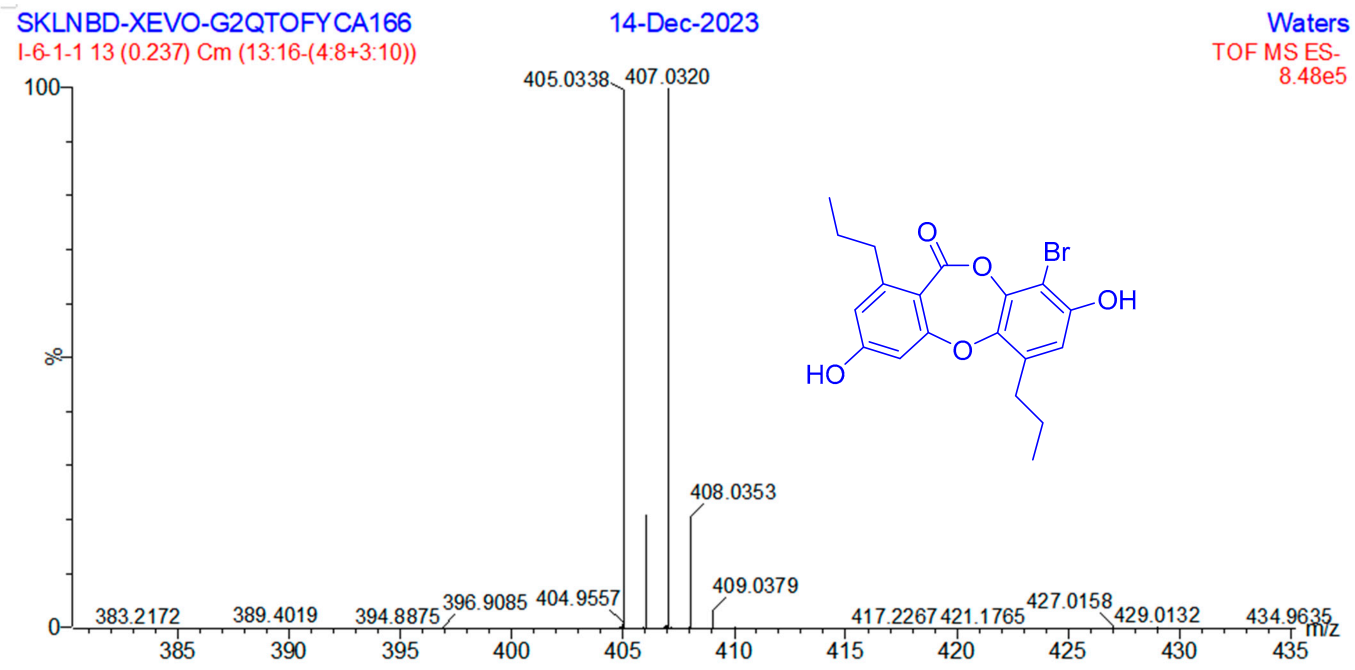

Mass	Calc. Mass	mDa	PPM	DBE	i-FIT	Norm	Conf(%)	Formula
405.0338	405.0338	0.0	0.0	10.5	476.0	0.000	100.00	$C_{19}H_{18}O_5Br$
	405.0340	-0.2	-0.5	30.5	509.1	33.139	0.00	$C_{32}H_5O$

Figure S102. HRESIMS spectrum of **13**

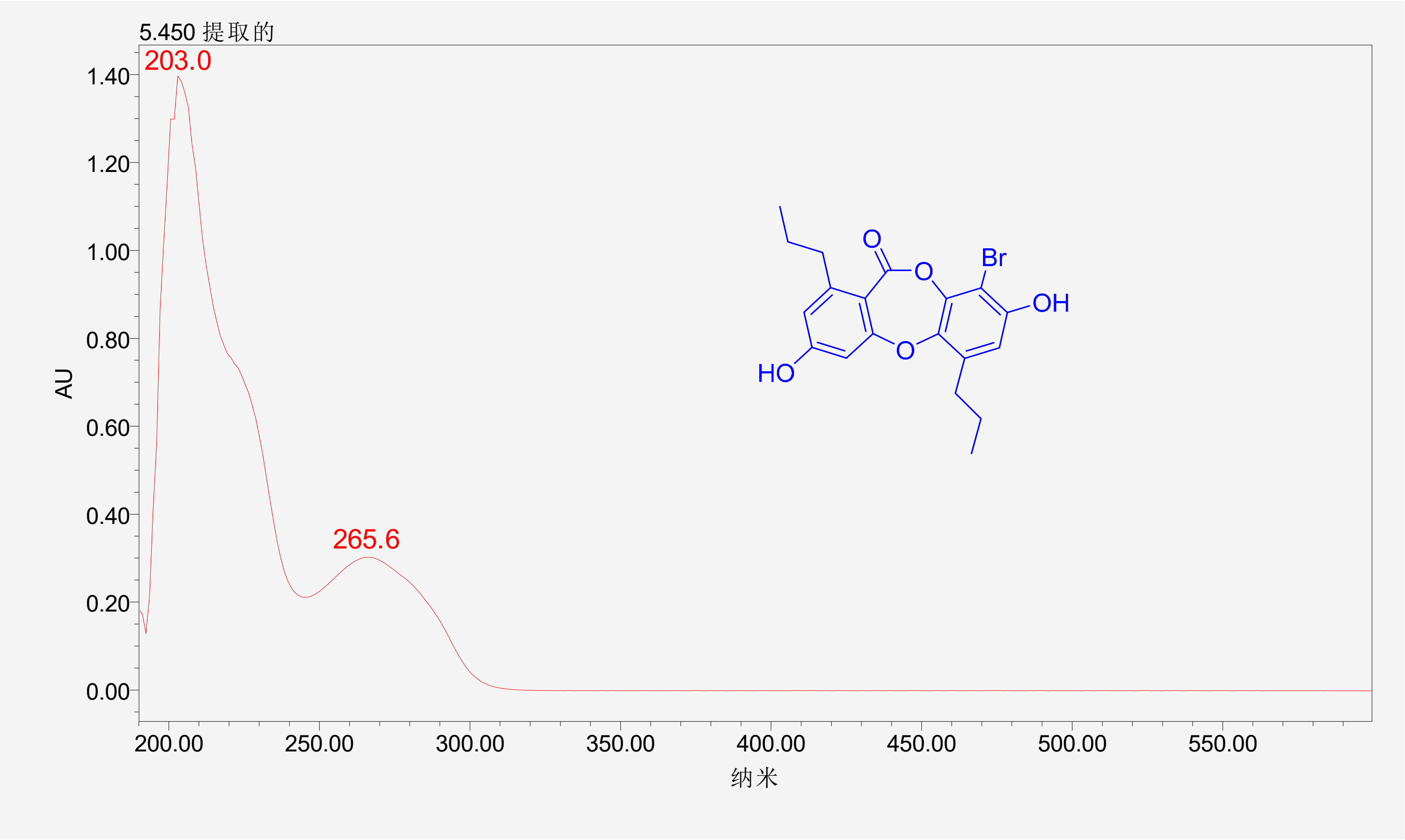

Figure S103. UV spectrum of **13**

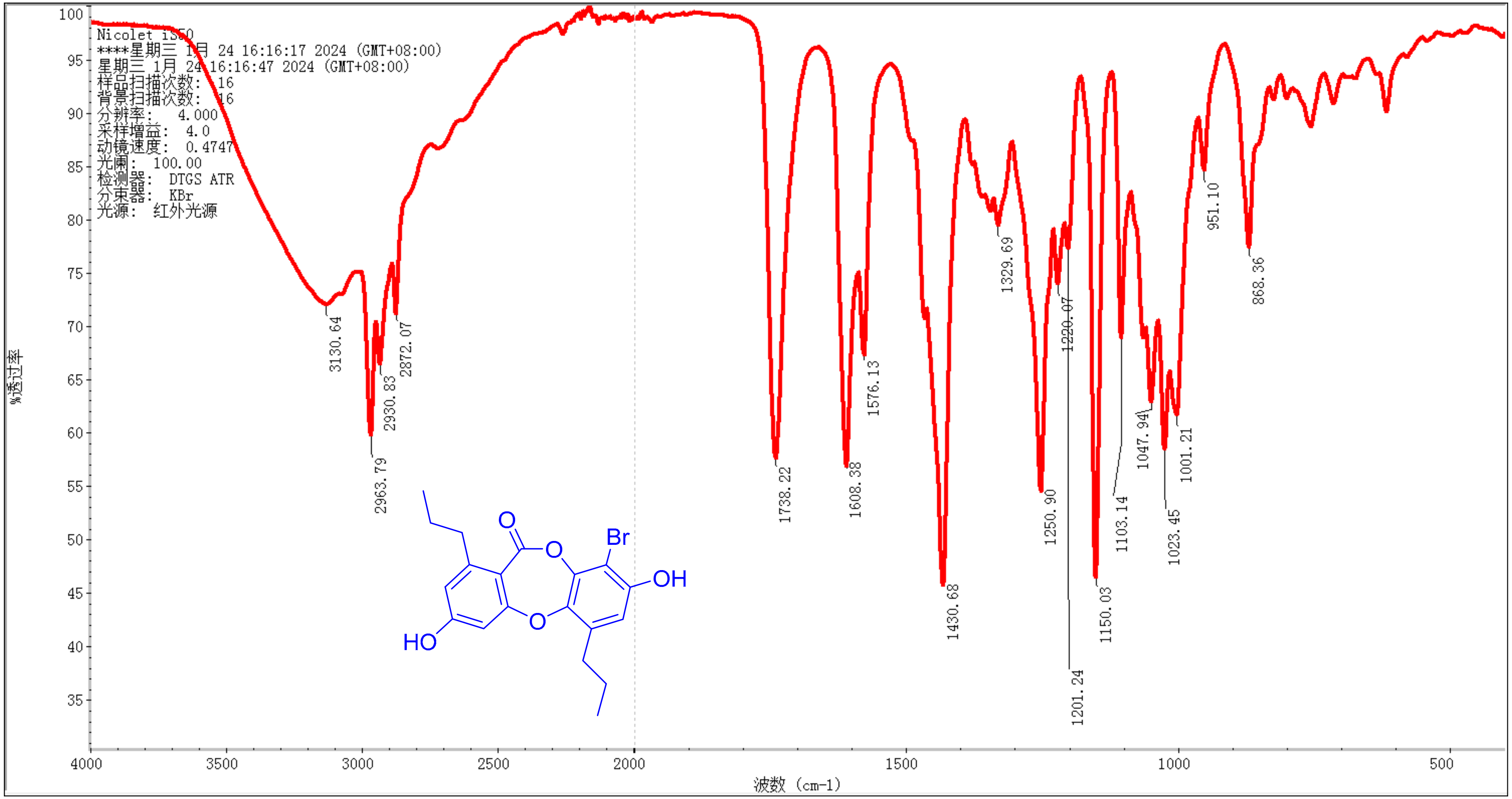

Figure S104. IR spectrum of **13**

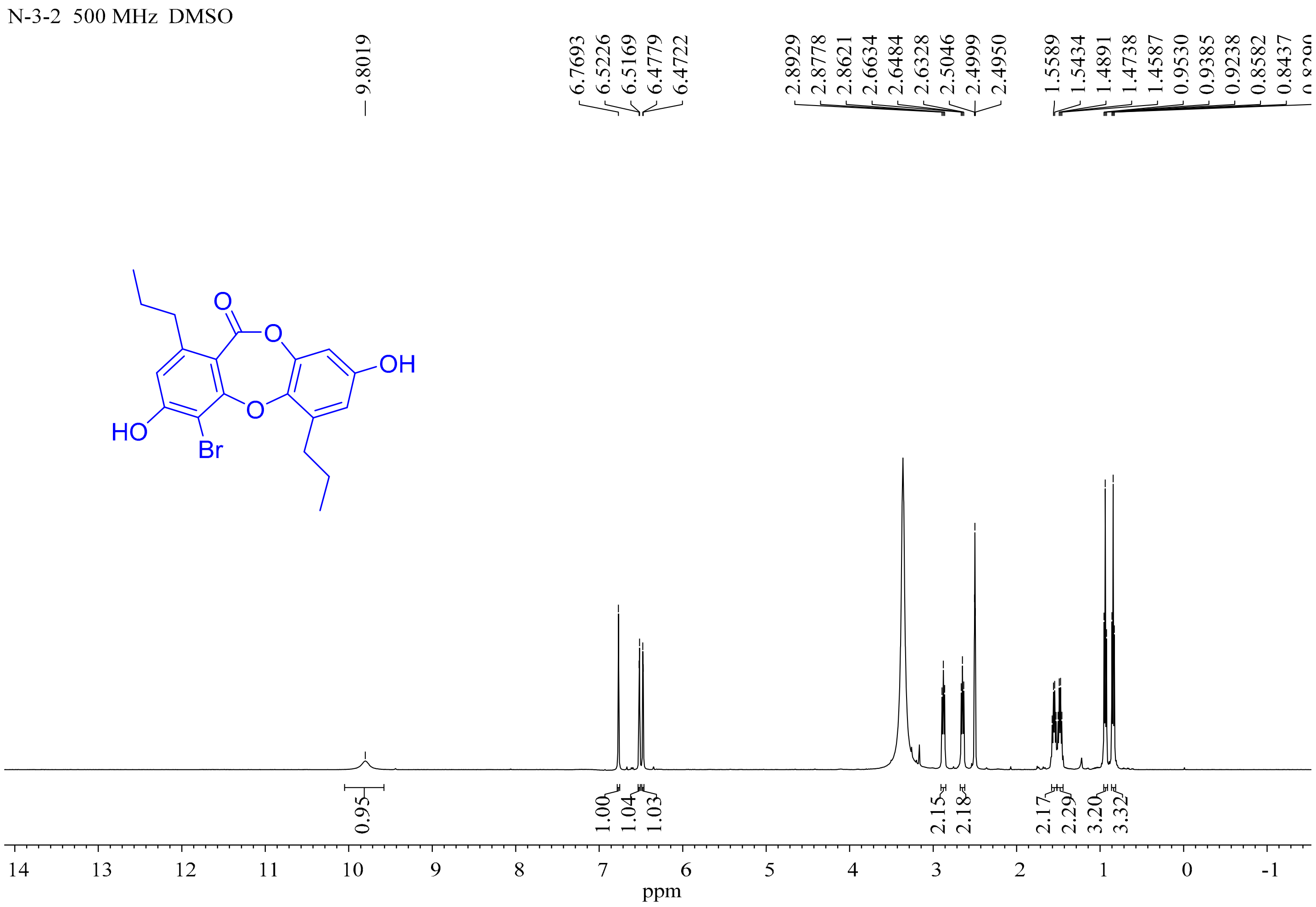

Figure S105. ^{1}H-NMR spectrum of **14** in DMSO-d_6 (500 MHz)

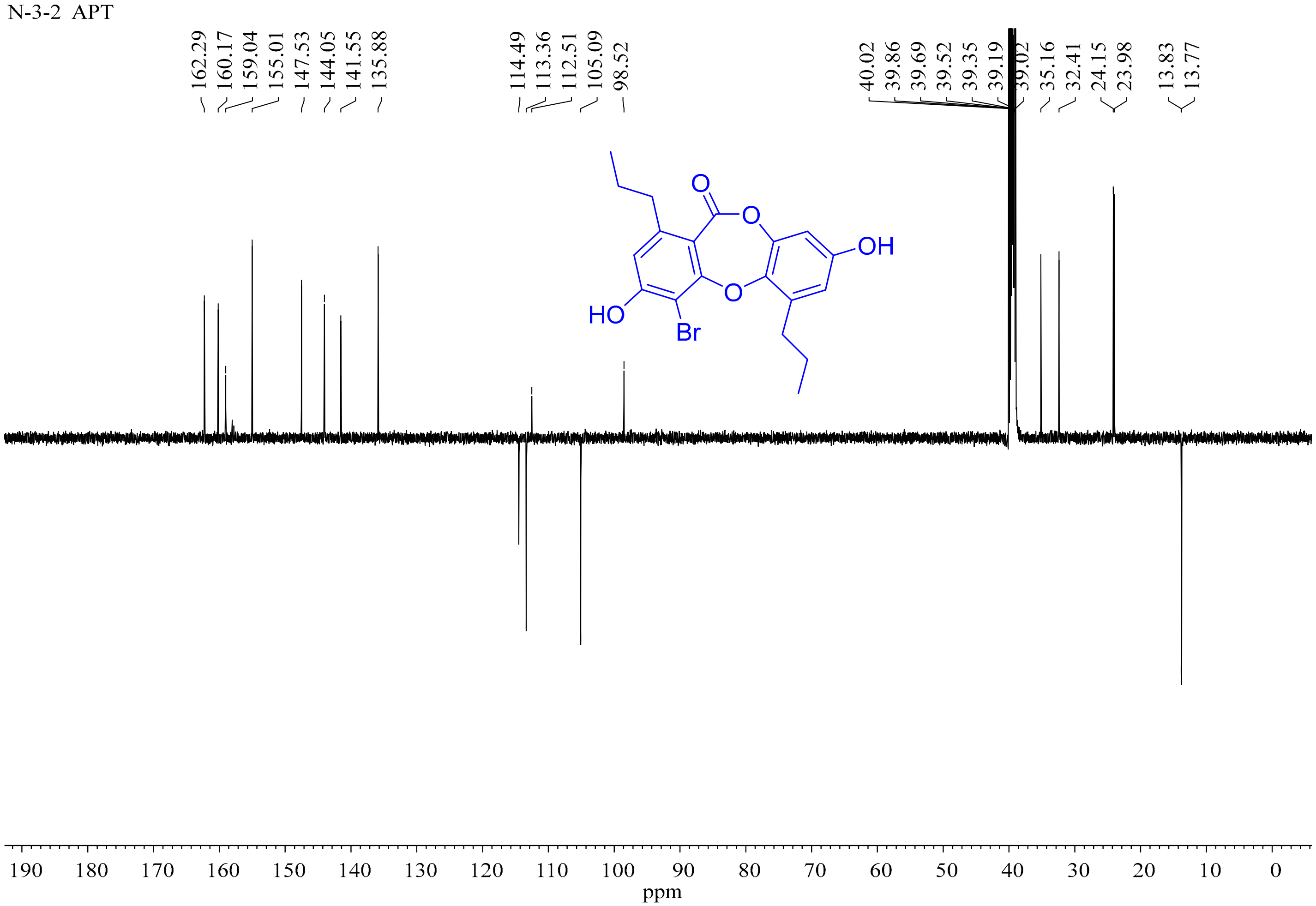

Figure S106. APT spectrum of **14** in DMSO-d_6 (125 MHz)

N-3-2 HSQC

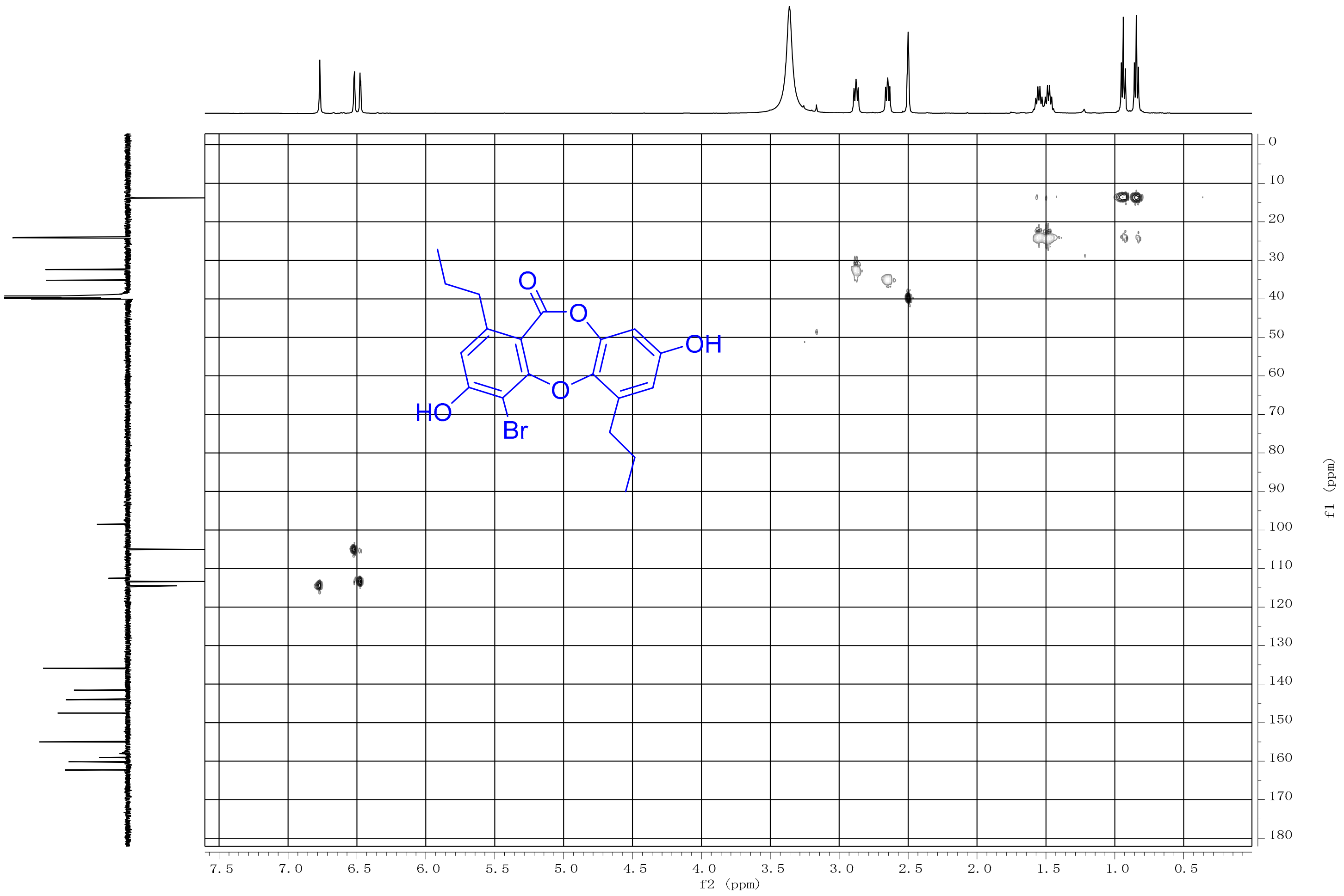

Figure S107. HSQC spectrum of **14** in DMSO-d_6

N-3-2 COSY

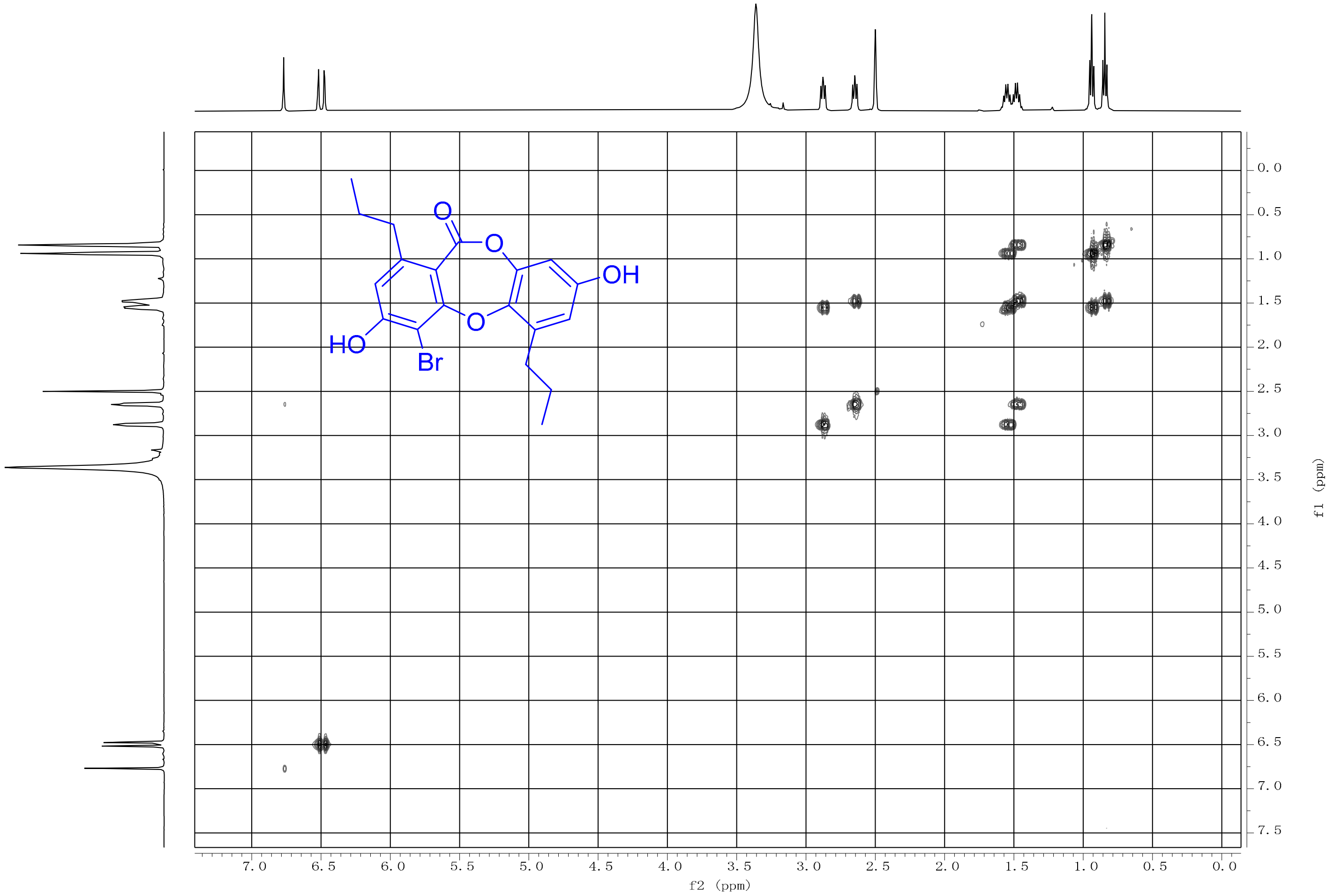

Figure S108. ^1H-^1H COSY spectrum of **14** in DMSO-d_6

N-3-2 HMBC

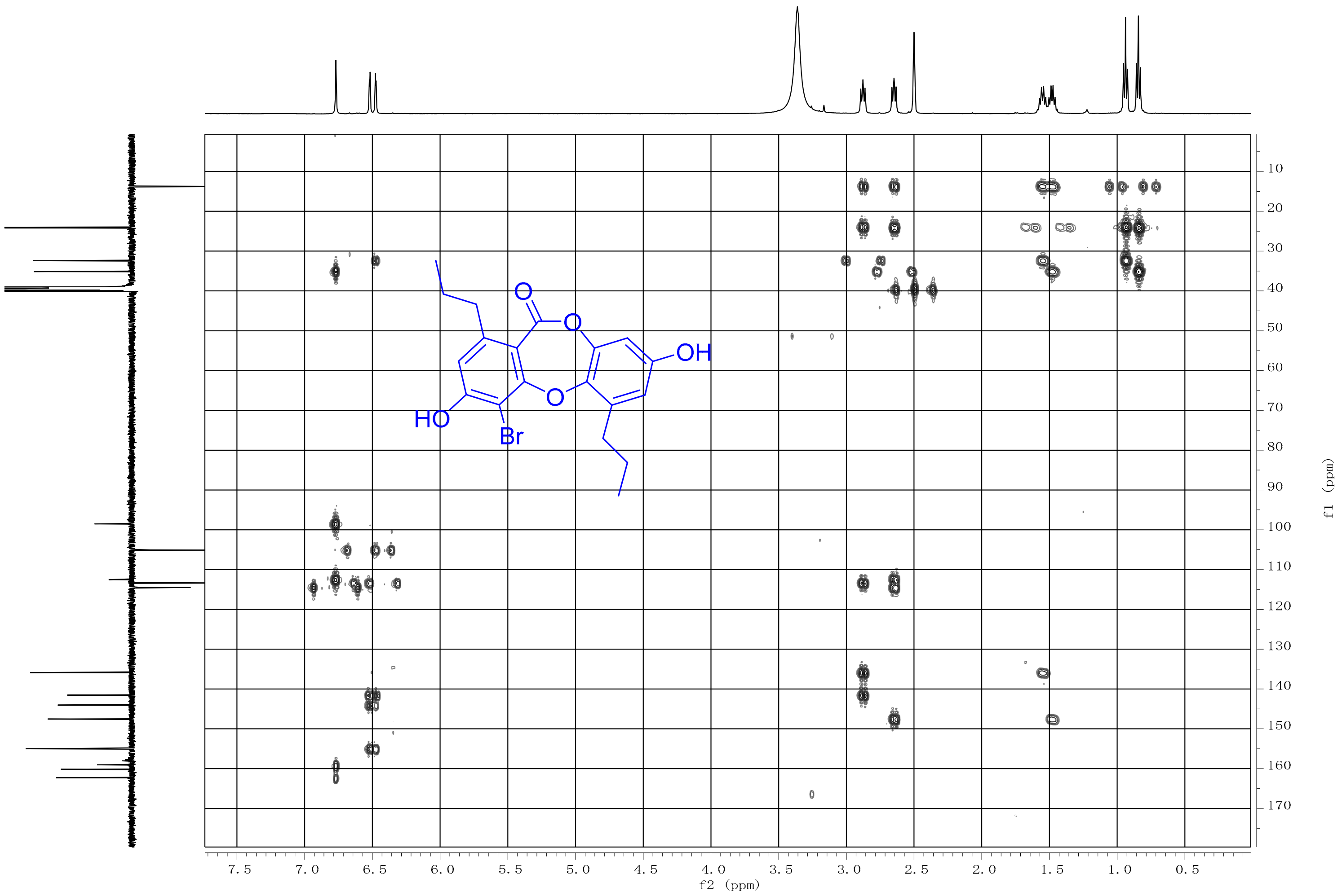

Figure S109. HMBC spectrum of **14** in DMSO-d_6

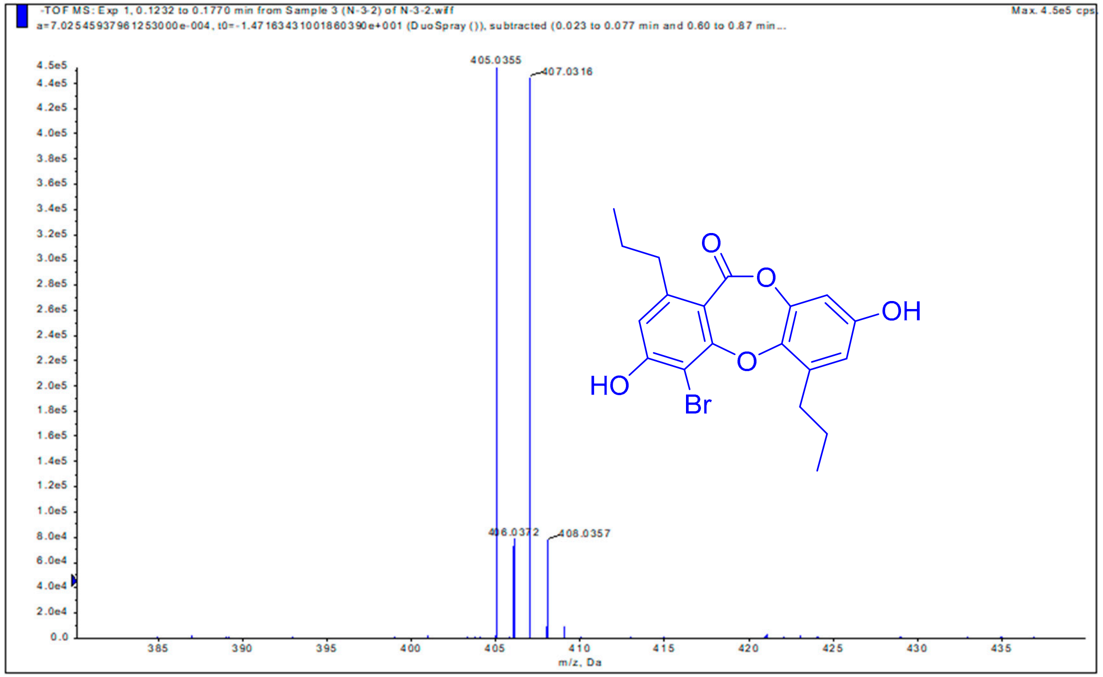

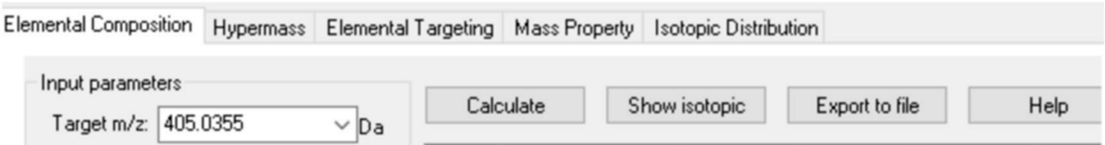

	Formula ...	Calculated	mDa Error	ppm Error	DBE
1	C19 H18 O5 Br	405	1.7399	4.2958	10.5

Figure S110. HRESIMS spectrum of **14**

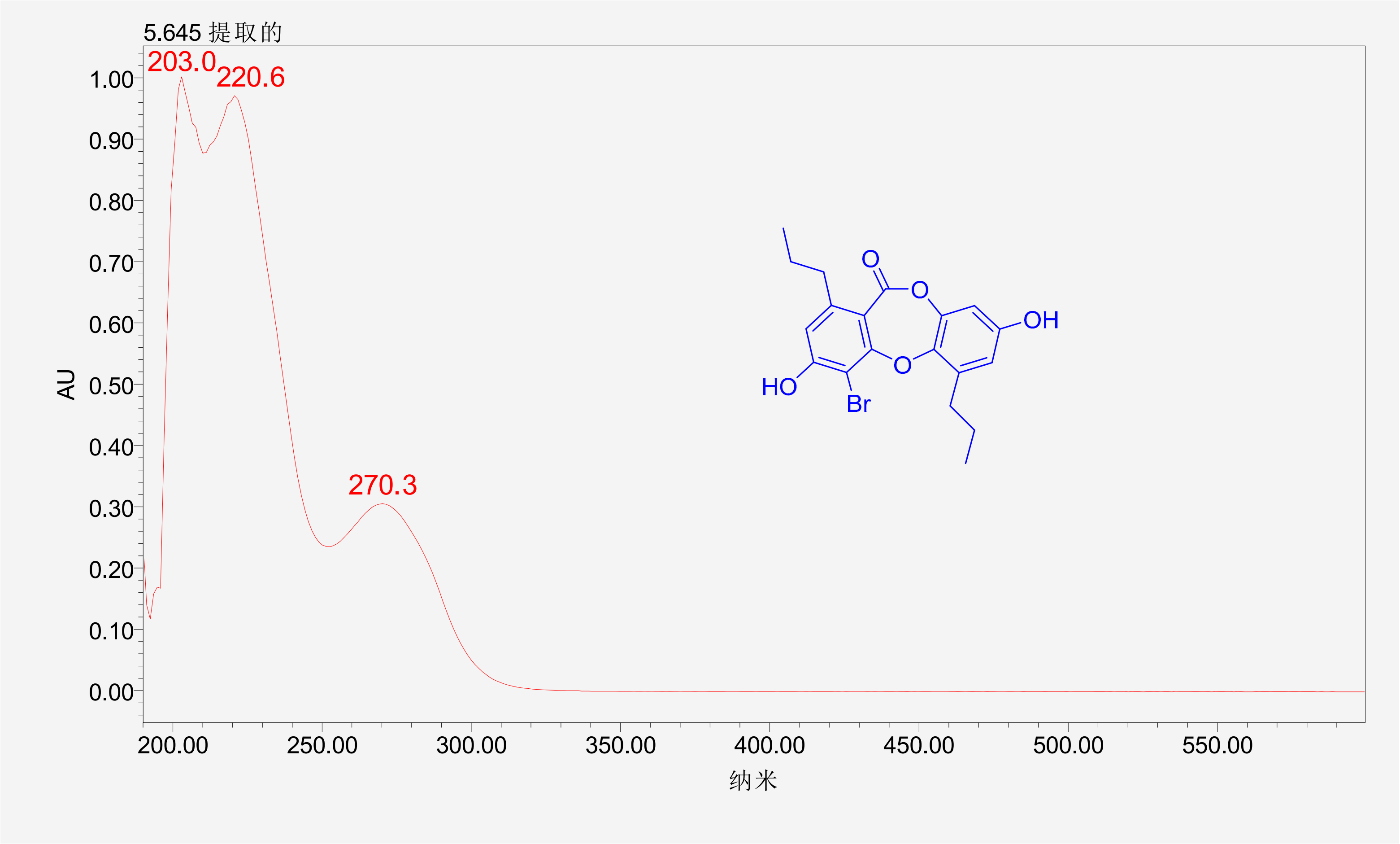

Figure S111. UV spectrum of **14**

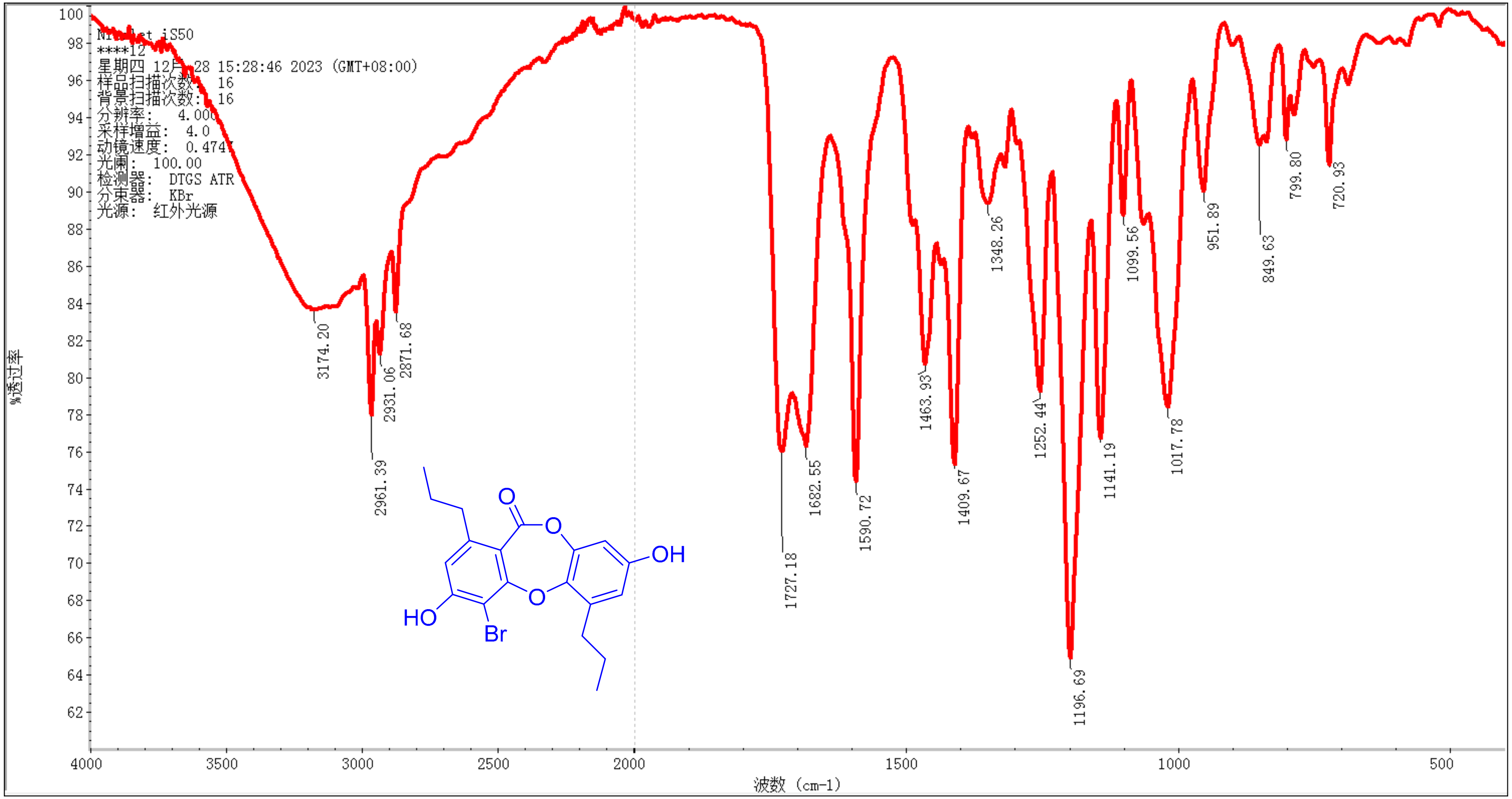

Figure S112. IR spectrum of **14**

N-5-4 500 MHz DMSO

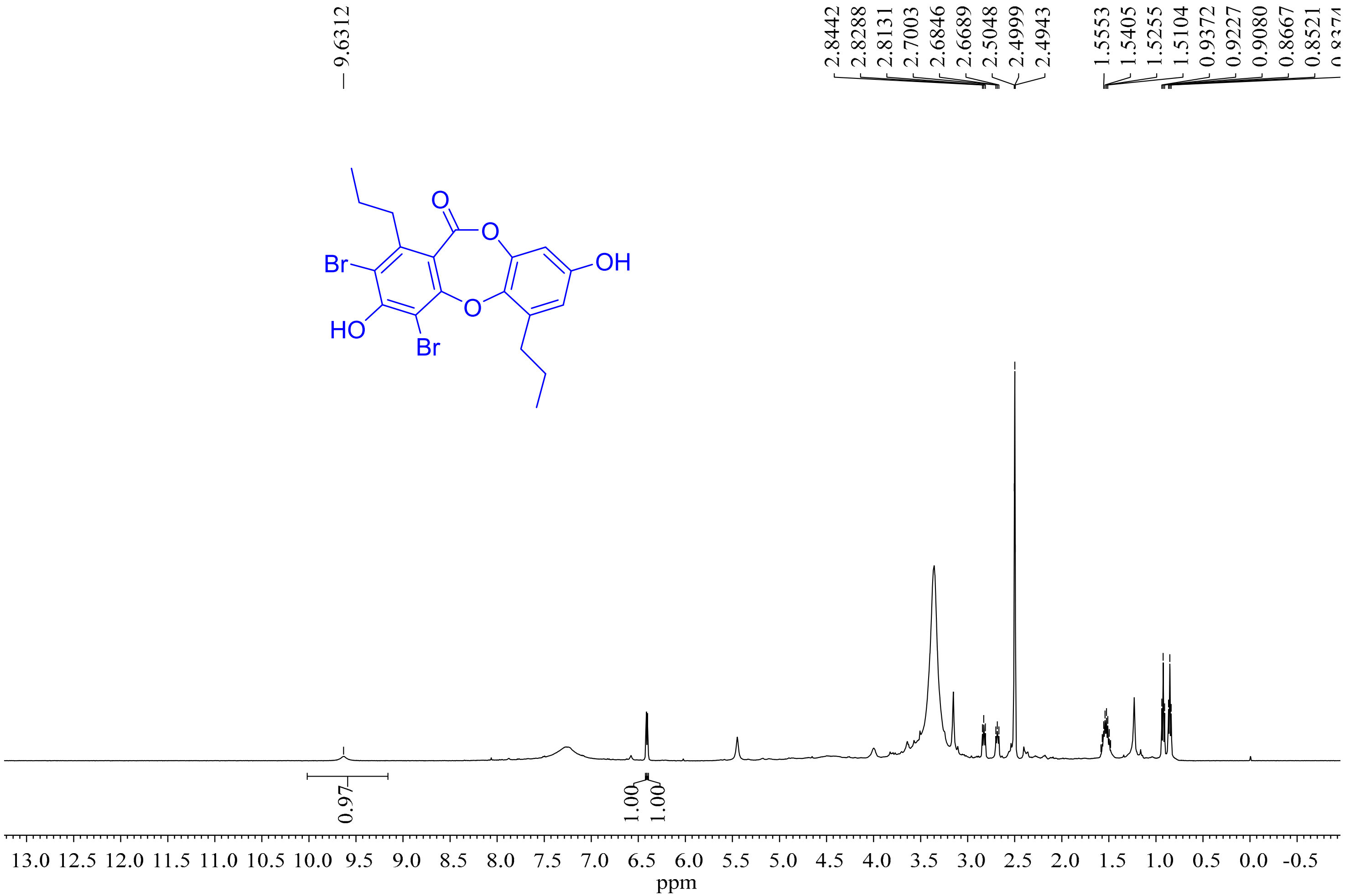

Figure S113. ^{1}H-NMR spectrum of **15** in DMSO-d_6 (500 MHz)

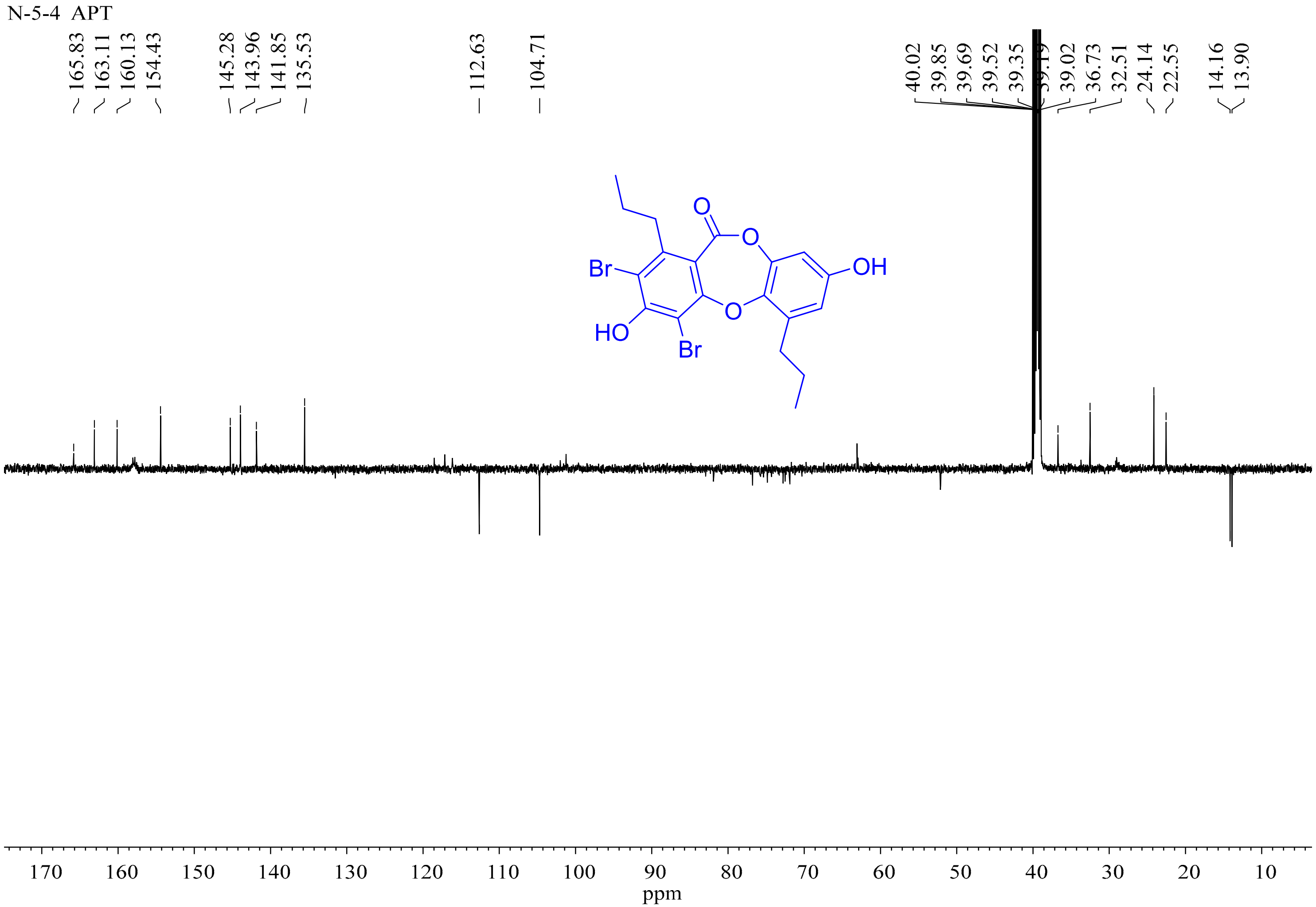

Figure S114. APT spectrum of **15** in DMSO-d_6 (125 MHz)

N-5-4 HSQC

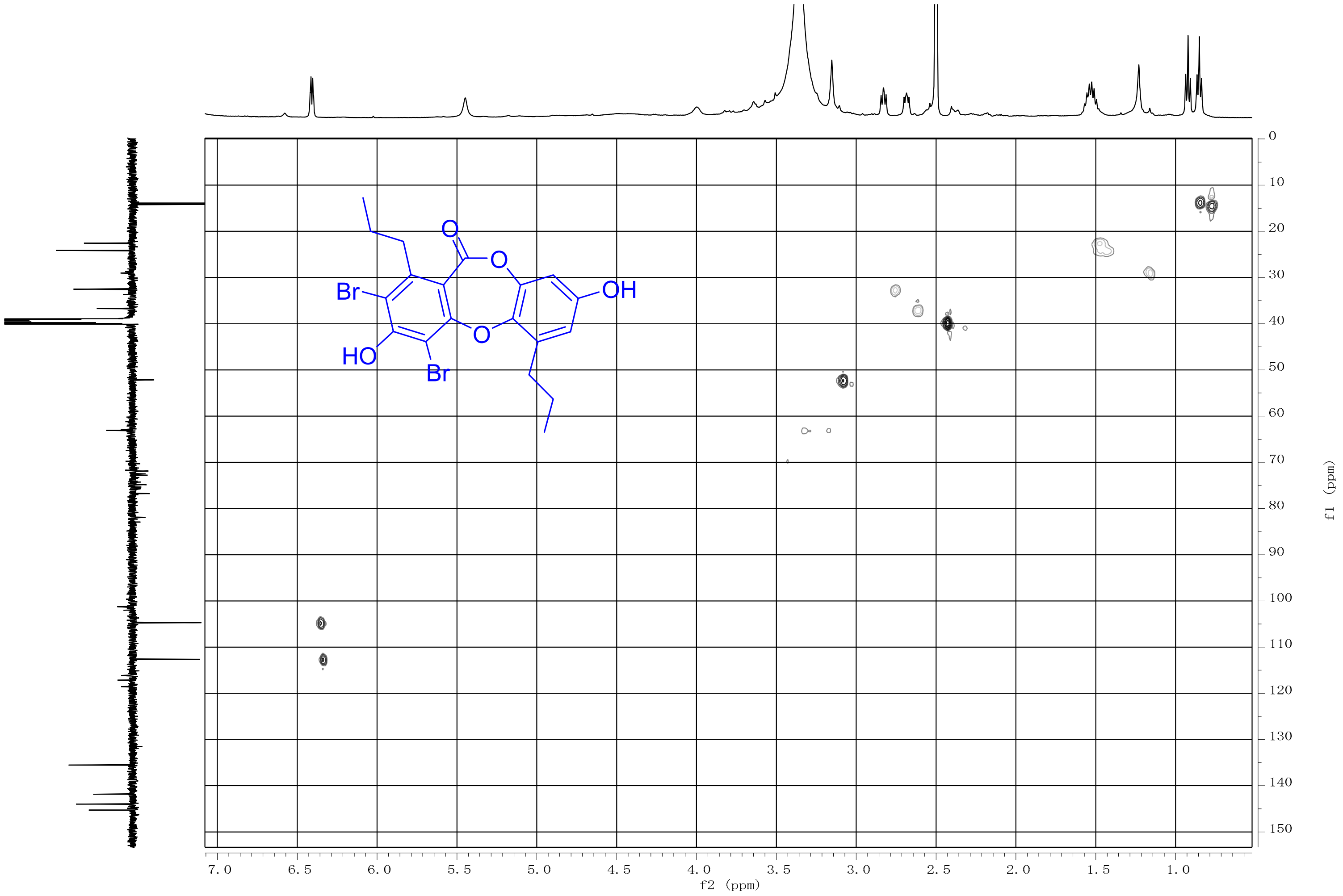

Figure S115. HSQC spectrum of **15** in DMSO-d_6

N-5-4 COSY

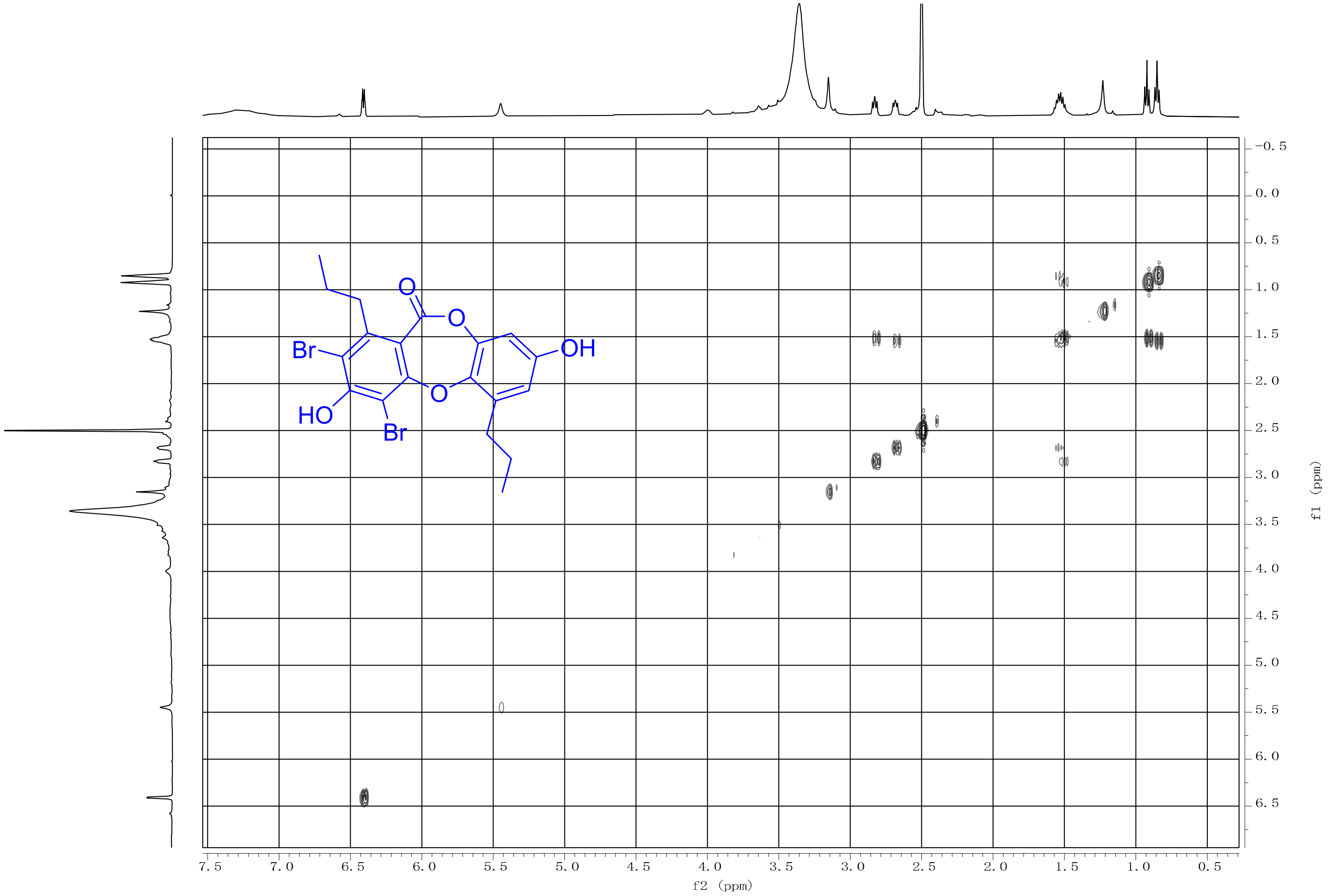

Figure S116. ^1H-^1H COSY spectrum of **15** in DMSO-d_6

N-5-4 HMBC

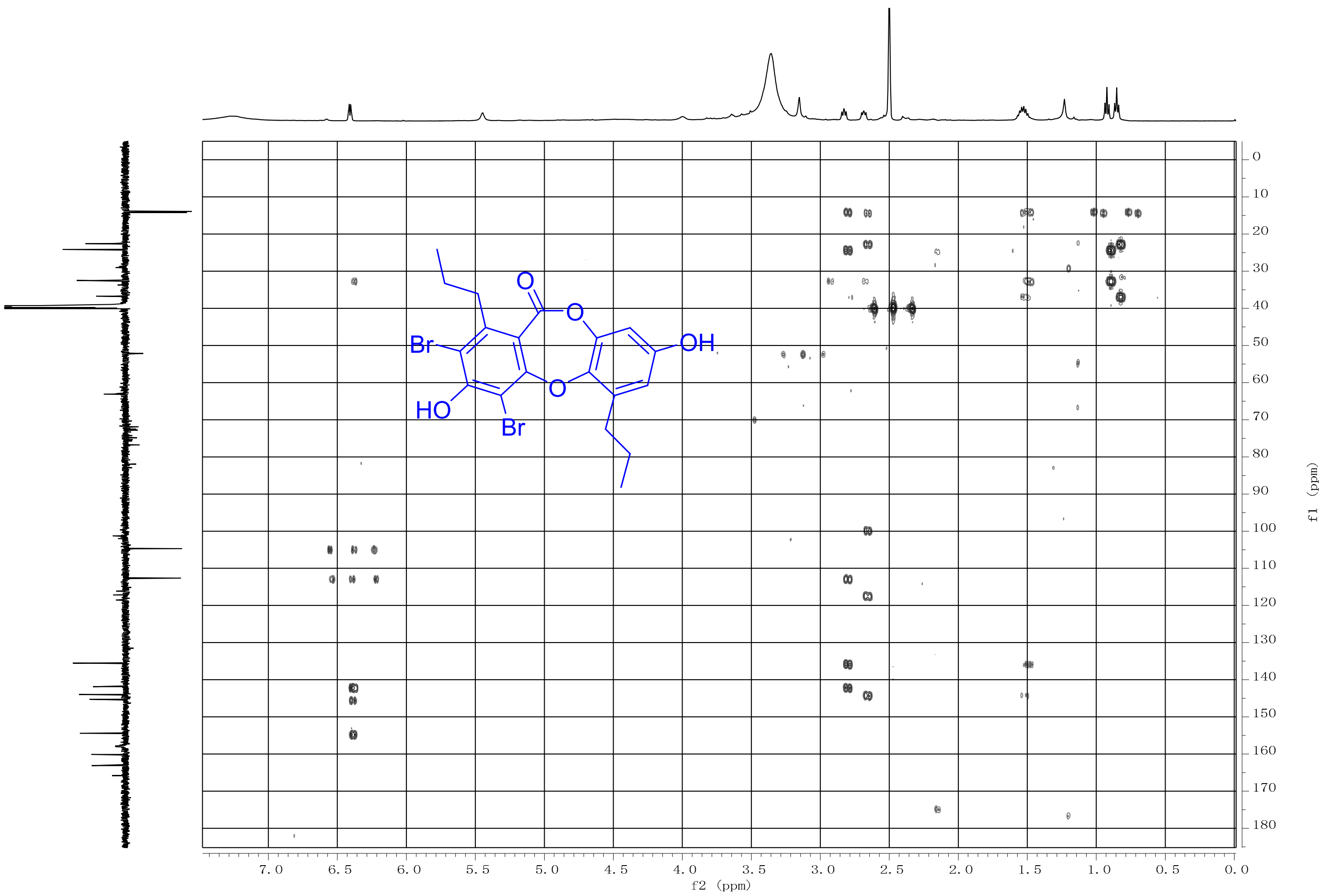

Figure S117. HMBC spectrum of **15** in DMSO-d_6

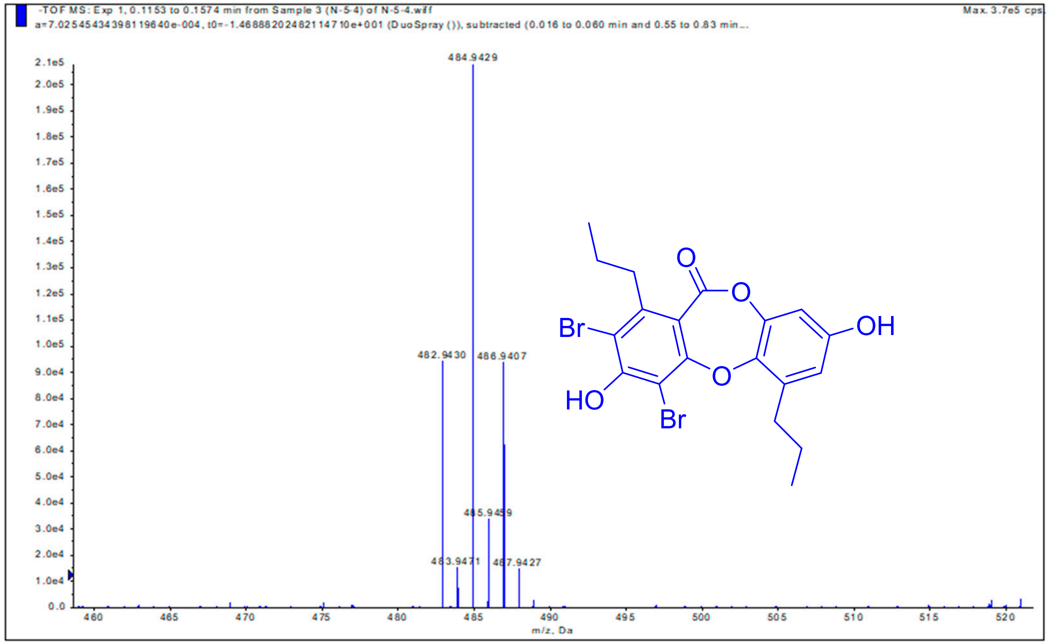

Elemental Composition | Hypermass | Elemental Targeting | Mass Property | Isotopic Distribution

Input parameters

Target m/z: 482.9430 Da

Tolerance: 5 ppm

Calculate | Show isotopic | Export to file | Help

	Formula ...	Calculated	mDa Error	ppm Error	DBE
1	C19 H17 O5 Br2	482.9	-1.271	-2.6319	10.5

Figure S118. HRESIMS spectrum of **15**

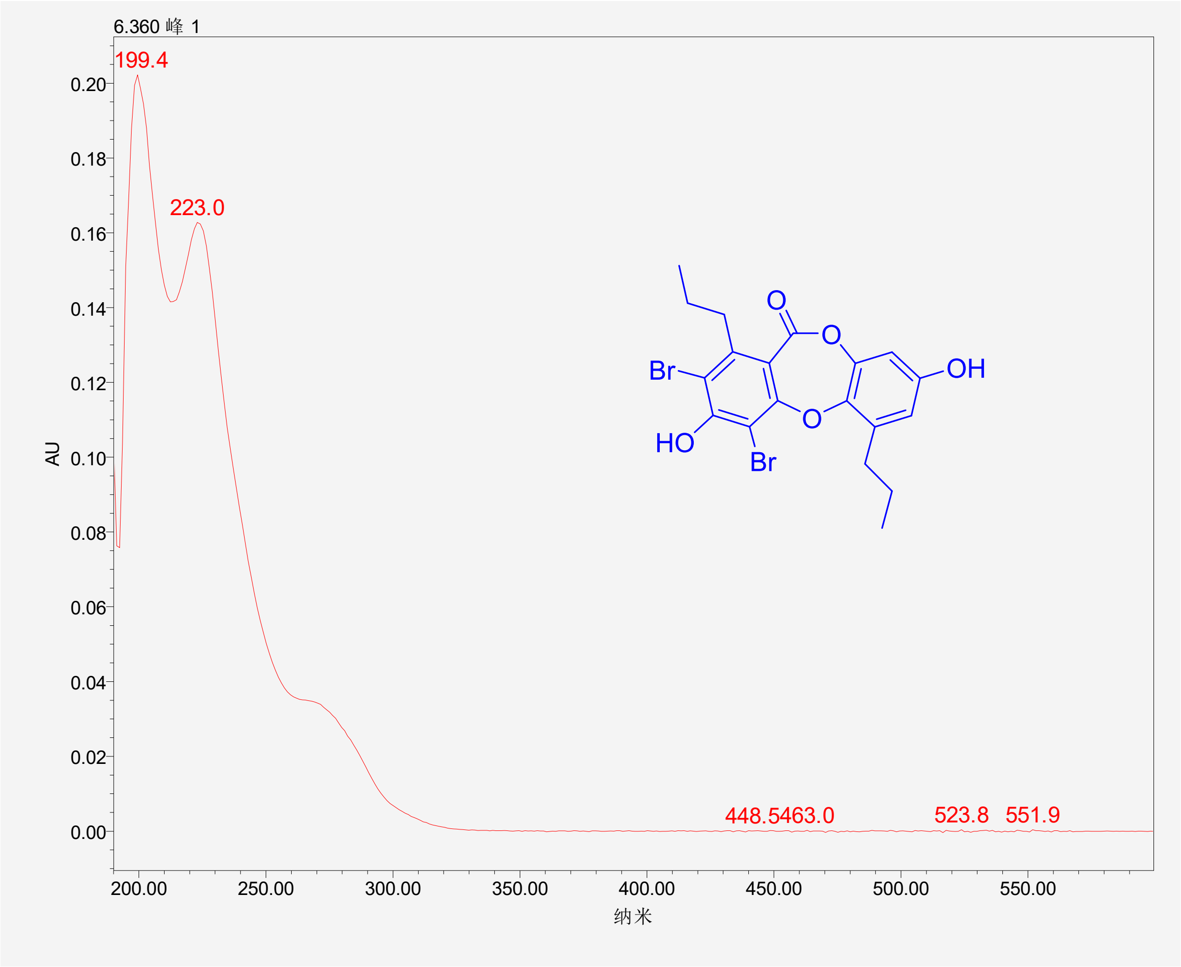

Figure S119. UV spectrum of **15**

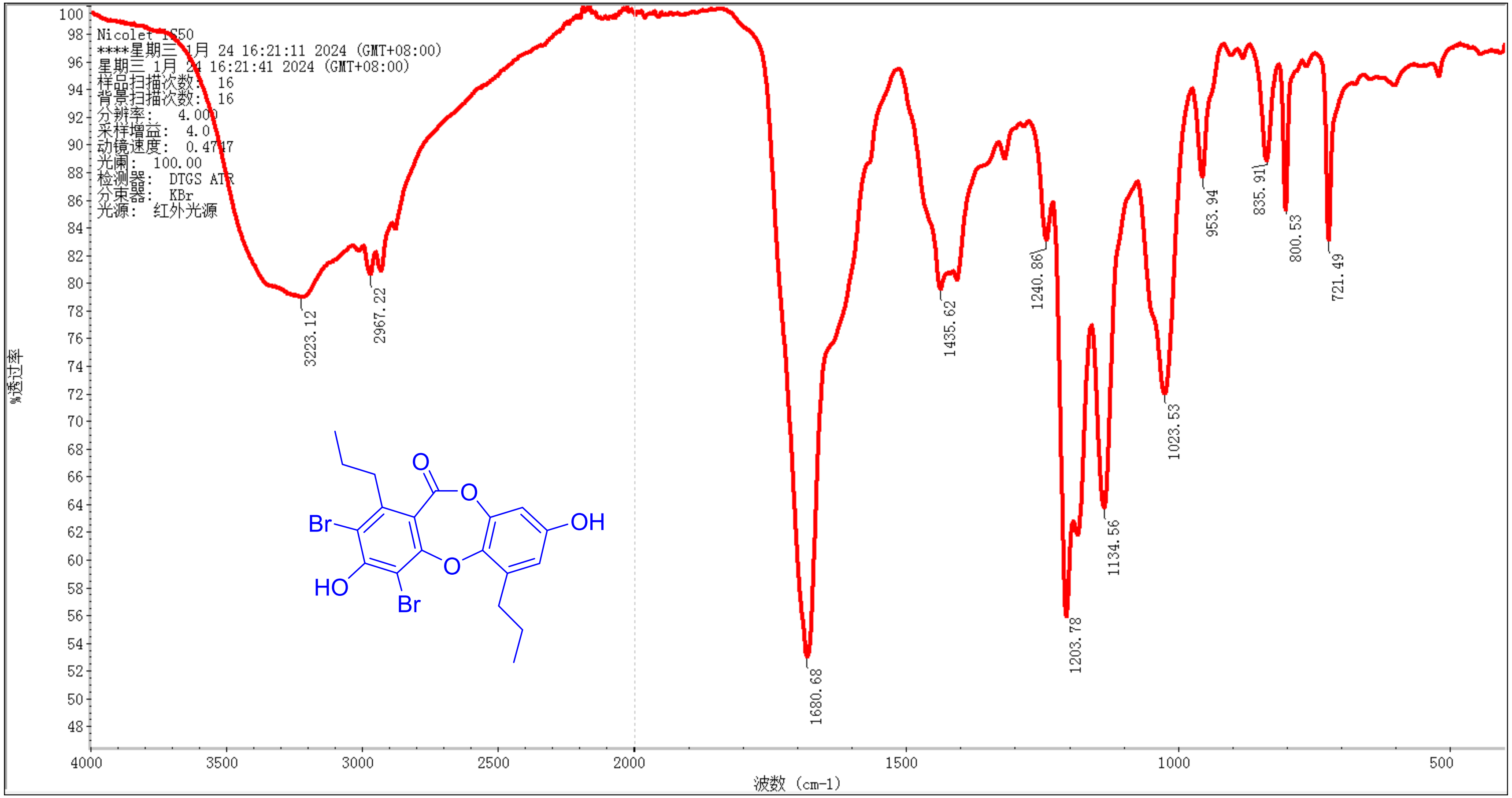

Figure S120. IR spectrum of **15**

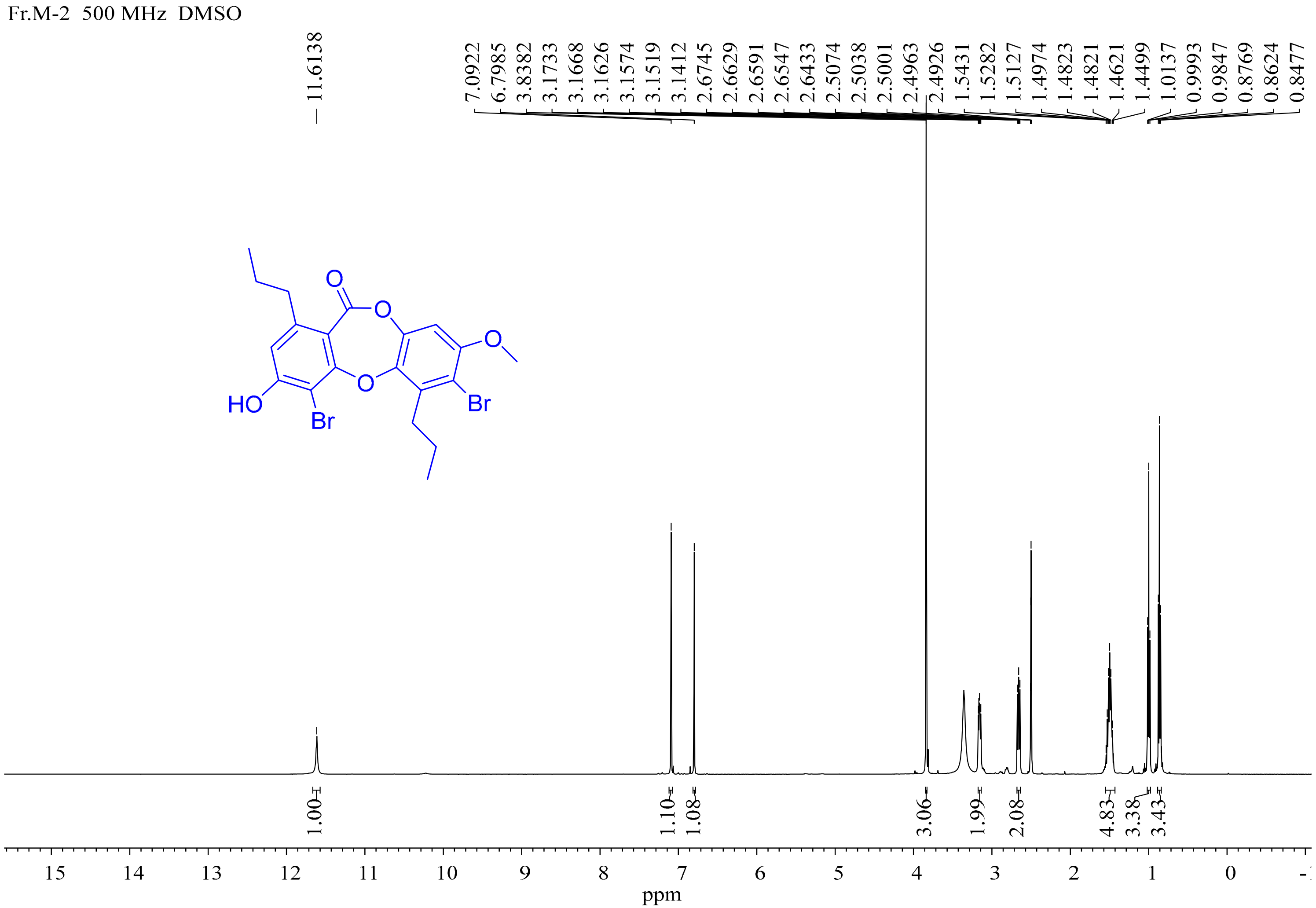

Figure S121. ^{1}H-NMR spectrum of **16** in DMSO-d_6 (500 MHz)

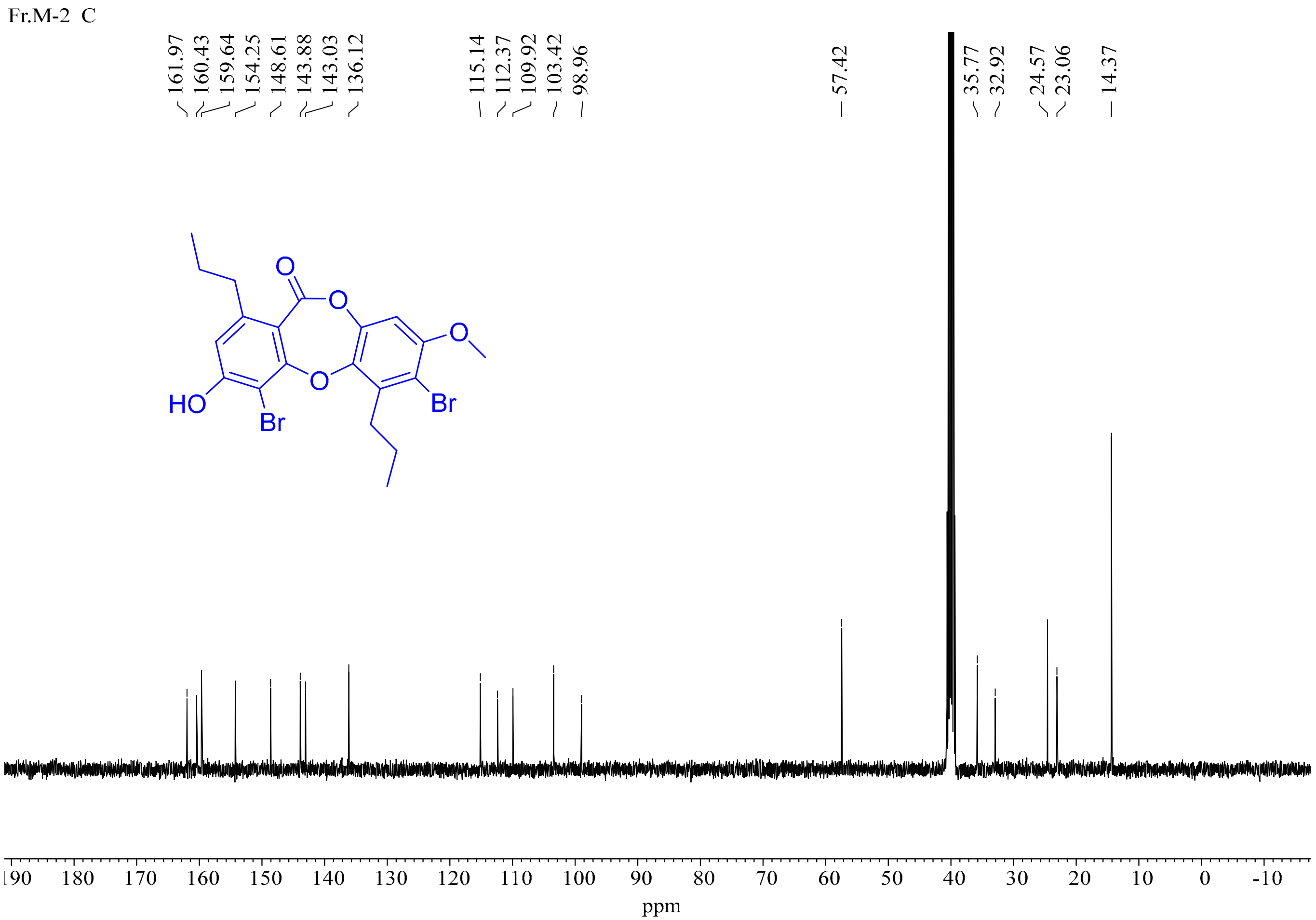

Figure S122. ^{13}C-NMR spectrum of **16** in DMSO-d_6 (125 MHz)

Fr.M-2 HSQC

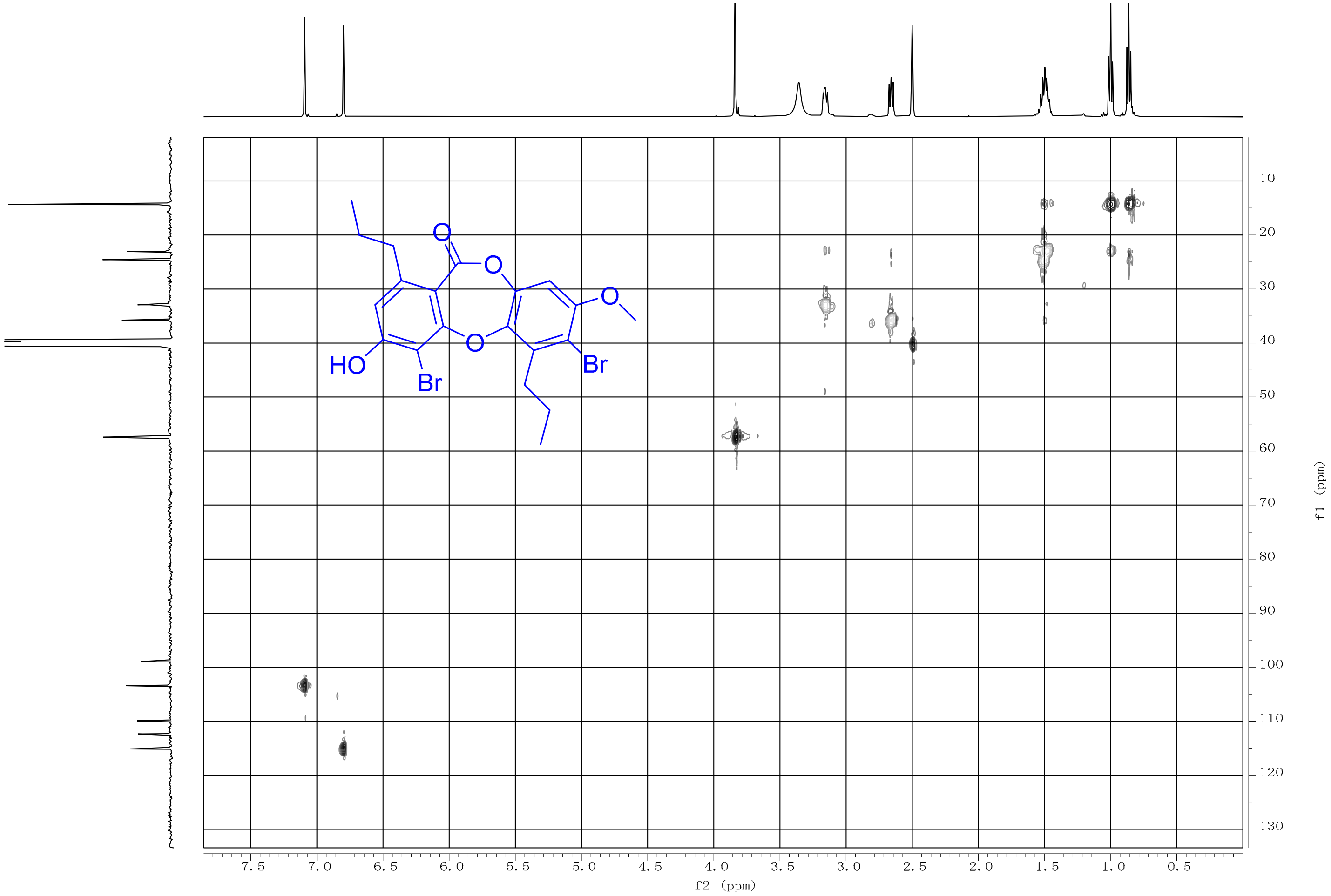

Figure S123. HSQC spectrum of **16** in DMSO-d_6

Fr.M-2 COSY

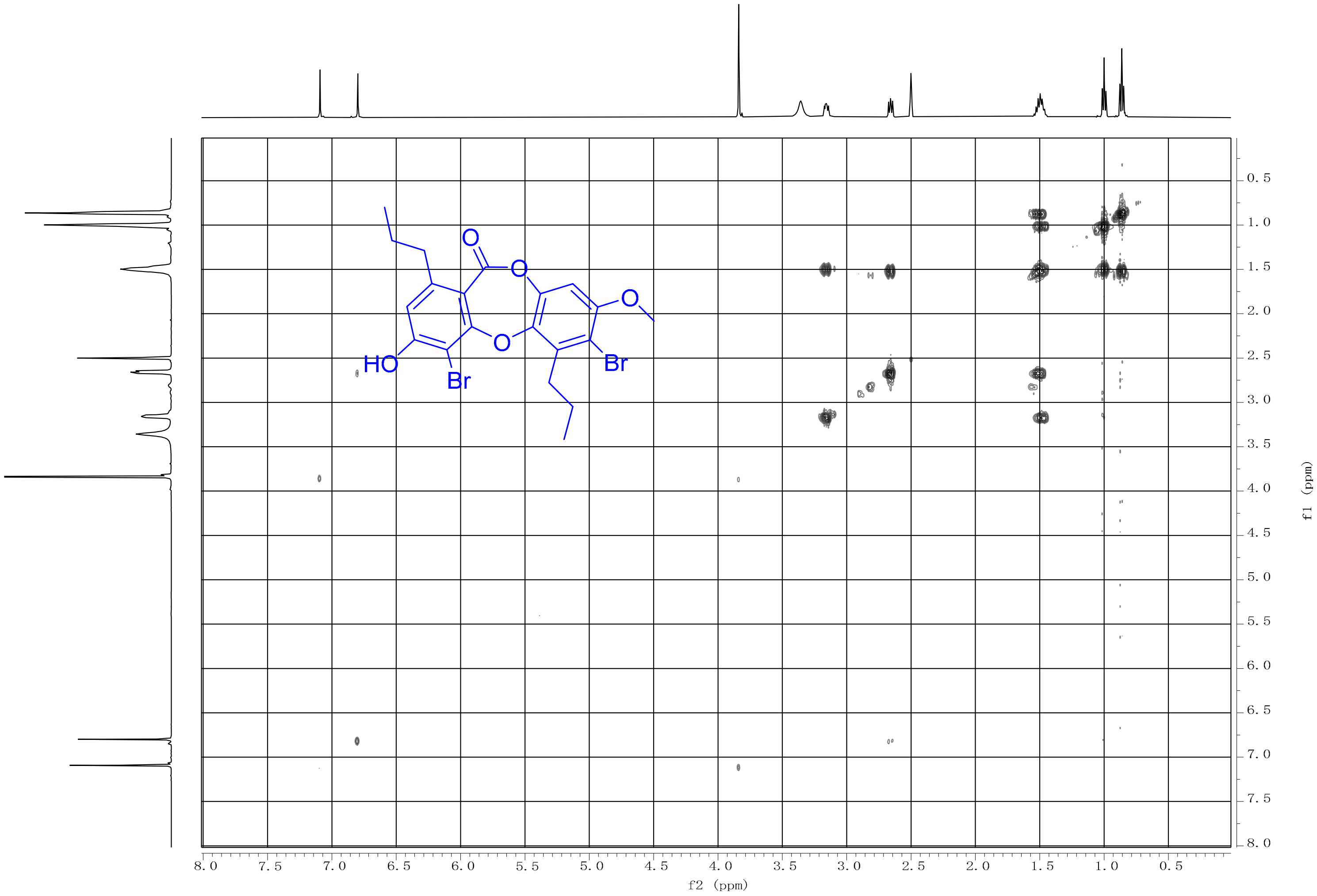

Figure S124. ^{1}H-^{1}H COSY spectrum of **16** in DMSO-d_6

Fr.M-2 HMBC

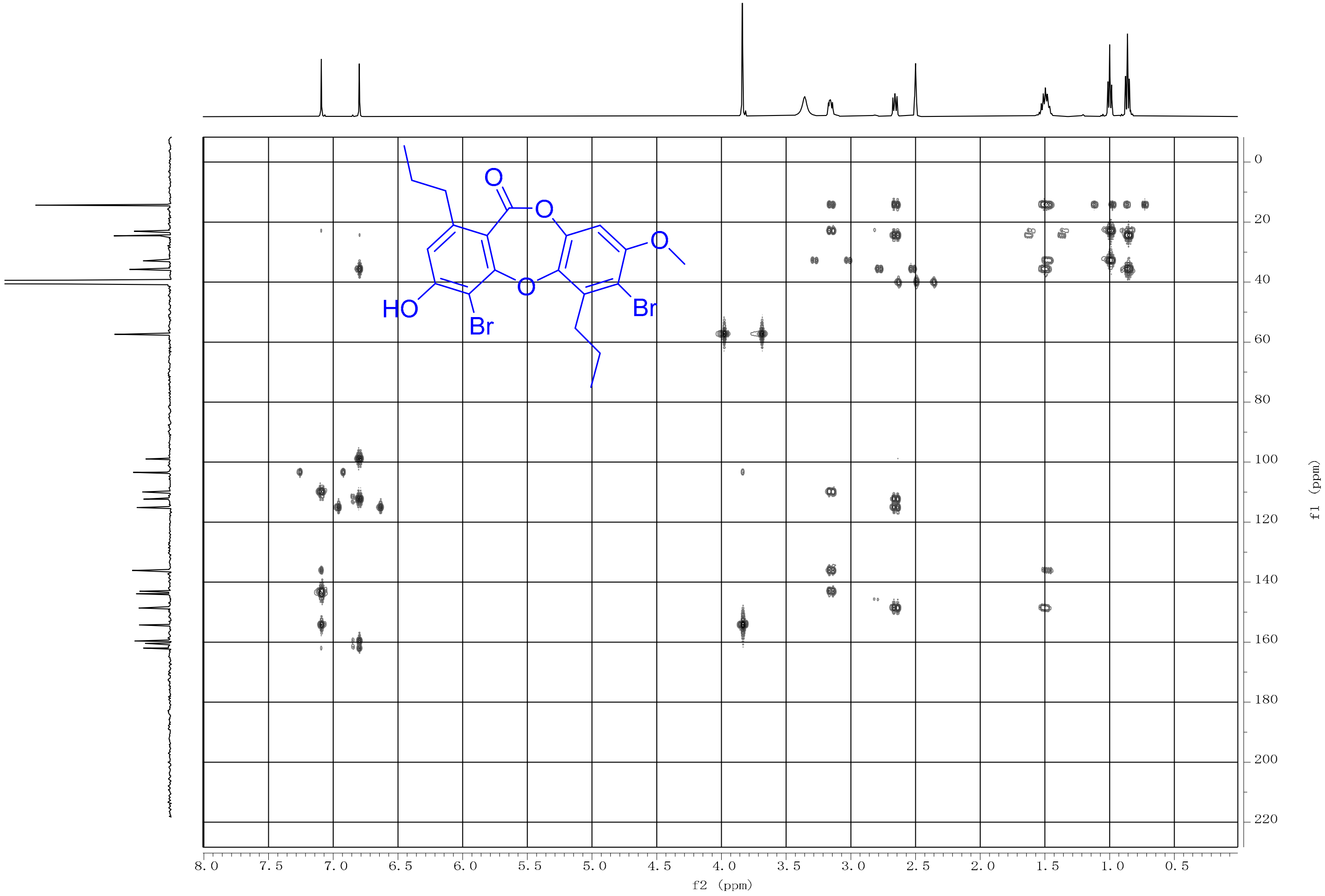

Figure S125. HMBC spectrum of **16** in DMSO-d_6

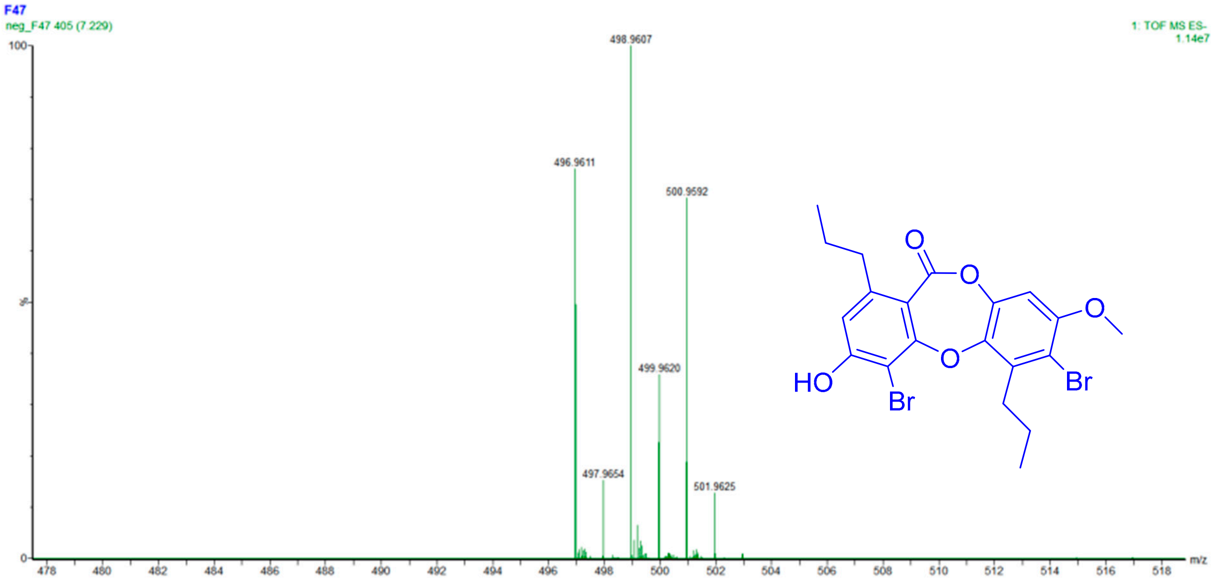

Single Mass Analysis

Tolerance = 5.0 mDa / DBE: min = -1.5, max = 50.0

Element prediction: Off

Monoisotopic Mass, Even Electron Ions

351 formula(e) evaluated with 8 results within limits (up to 50 closest results for each mass)

Elements Used:

C: 0-500 H: 0-1000 O: 0-200 Br: 0-8

Mass	Calc. Mass	mDa	PPM	DBE	Formula	C	H	O	Br
496.9611	496.9658	-4.7	-9.5	1.5	C13 H23 O10 Br2	13	23	10	2
	496.9599	1.2	2.4	10.5	C20 H19 O5 Br2	20	19	5	2

Figure S126. HRESIMS spectrum of **16**

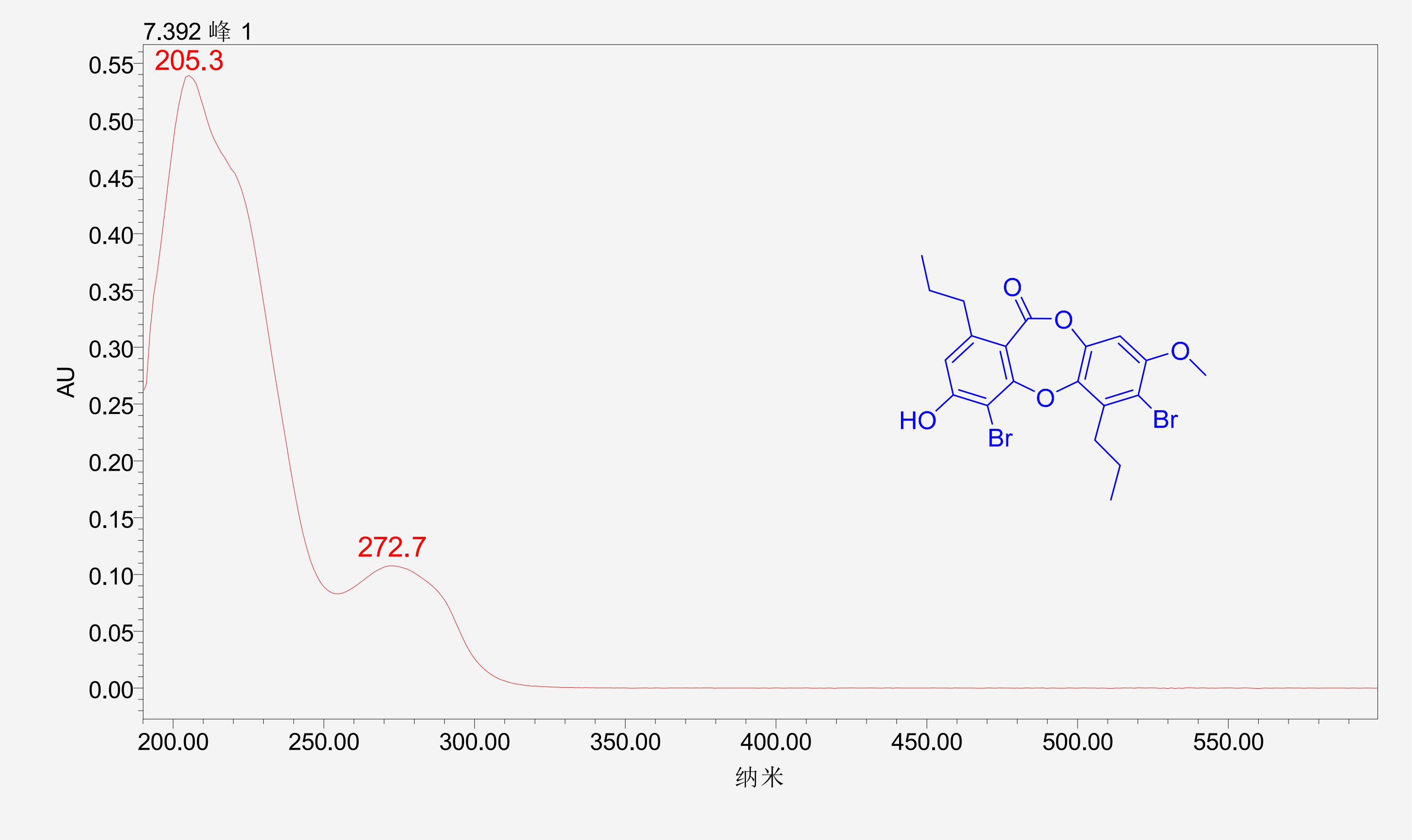

Figure S127. UV spectrum of **16**

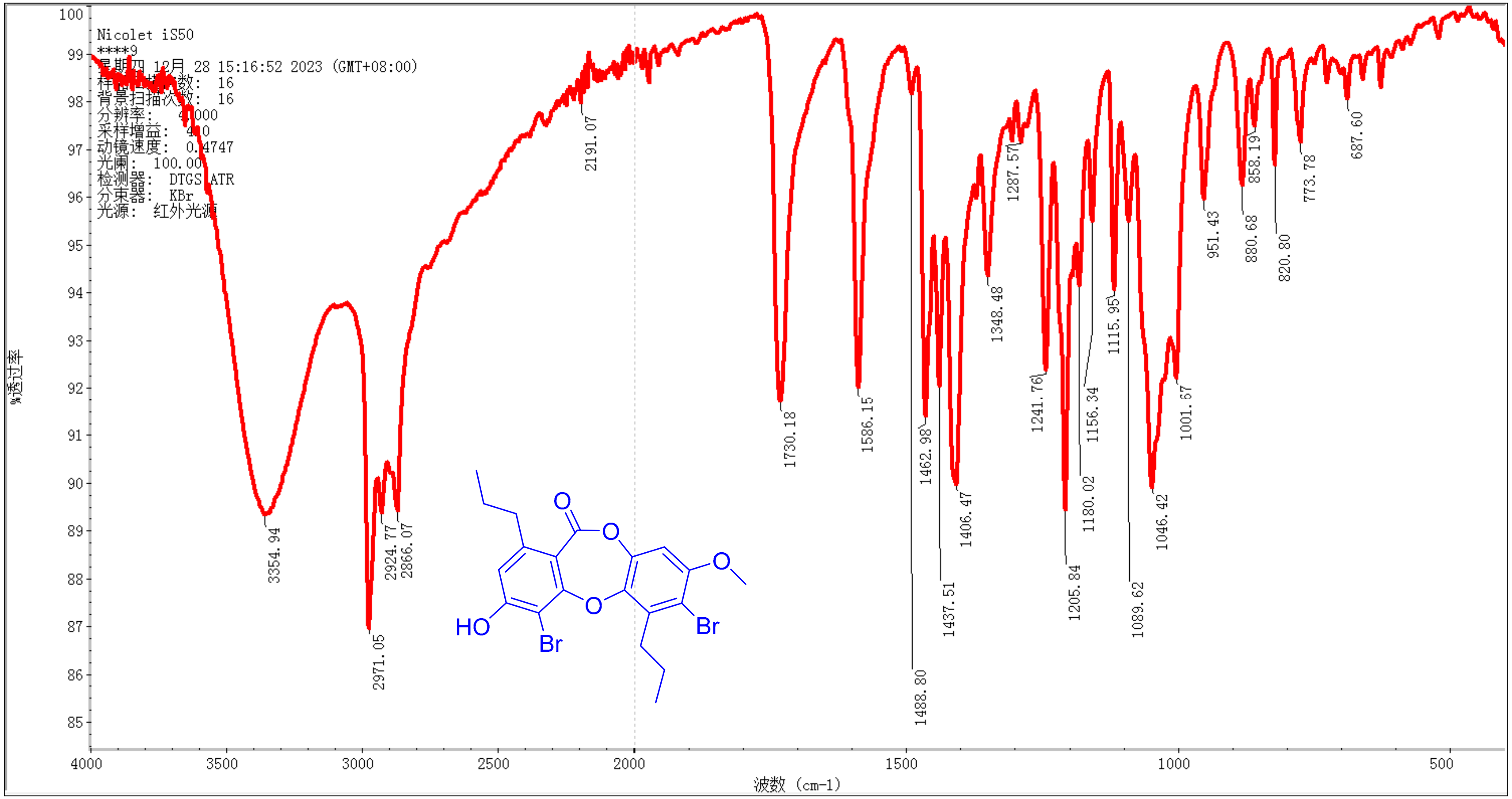

Figure S128. IR spectrum of **16**

Table S12. Comparison of the ^{1}H NMR data of **12-16** to spiromastixones P-T (δ_H ppm, *J* in Hz)

Position	**12**[a,d]	**P**[b,e]	**13**[a,d]	**Q**[a,e]	**14**[a,d]	**R**[b,e]	**15**[a,d]	**S**[a,f]	**16**[a,d]	**T**[a,e]
3	6.60, d (2.3)	6.73, d (2.0)	6.59, d (2.3)	6.72, d (2.0)	-	-	-	-	-	-
5	6.62, d (2.3)	6.72, d (2.0)	6.62, d (2.3)	6.71, d (2.0)	6.77, s	6.90, s	-	-	6.80, s	6.93, s
8	2.68, t (7.9)	2.76, t (7.8)	2.69, m	2.79, t (7.8)	2.65, t (7.8)	2.73, t (9.8)	2.68, t (7.9)	2.90, t (7.8)	2.66, t (7.9)	2.74, t (7.8)
9	1.48, m	1.57, m	1.47, m	1.57, m	1.48, m	1.57, m	1.55, m	1.64, m	1.50, m	1.58, m
10	0.85, t (7.3)	0.89, t (7.5)	0.82, t (7.3)	0.88, t (7.5)	0.84, t (7.4)	0.88, t (7.3)	0.85, t (7.4)	0.93, t (7.5)	0.86, t (7.4)	0.89, t (7.5)
3′	6.73, s	6.81, s	-	-	6.52, d (2.9)	6.57, d (3.0)	6.42, d (2.9)	6.49, d (2.5)	7.09, s	6.94, s
5′	-	-	6.66, s	6.79, s	6.47, d (2.9)	6.58, d (3.0)	6.40, d (2.9)	6.48, d (2.5)	-	-
6′	-	-	-	-	-	-	-	2.94, t (7.8)	-	-
7′	2.88, t (8.1)	2.99, t (8.0)	2.67, m	2.75, t (7.8)	2.88, t (7.9)	2.98, t (9.8)	2.83, t (7.9)	2.94, t (7.8)	3.16, t (8.1)	3.28, t (8.0)
8′	1.54, m	1.66, m	1.55, m	1.66, m	1.55, m	1.66, m	1.52, m	1.66, m	1.48, m	1.61, m
9′	1.04, t (7.4)	1.09, t (7.5)	0.97, t (7.4)	1.01, t (7.5)	0.94, t (7.4)	1.08, t (7.3)	0.92, t (7.4)	1.00, t (7.5)	1.00, t (7.3)	1.07, t (7.5)
OMe	-	-	-	-	-	-	-	-	3.84, s	3.92, s

[a]500 MHz; [b]400 MHz; [c]600 MHz; [d]Recorded in DMSO-d_6; [e]Recorded in acetone-d_6; [f]Recorded in methonal-d_4 P: spiromastixone P, Q: spiromastixone Q, R: spiromastixone R, S: spiromastixone S, T: spiromastixone T. The NMR data of known analogues are cited from the literature (Guo, Z.; Zhu, W.; Zhao, L.; Chen, Y.; Li, S.; Cheng, P.; Ge, H.; Tan, R.; Jiao, R. New antibacterial depsidones from an ant-derived fungus Spiromastix sp. MY-1, Chin. J. Nat. Med. 2022, 20, 627-632).

P

Table S13. Comparison of the ^{13}C NMR data of **12-16** to spiromastixones P-T (δ_C ppm)

Position	**12**^{a,d}	**P**^{b,e}	**13**^{a,d}	**Q**^{a,e}	**14**^{a,d}	**R**^{b,e}	**15**^{a,d}	**S**^{a,f}	**16**^{a,d}	**T**^{a,e}
1	111.4, C	113.1, C	111.2, C	113.1, C	112.9, C	114.8, C	100.0, C	116.7, C	112.4, C	114.0, C
2	162.7, C	162.8, C	162.6, C	162.7, C	160.6, C	159.4, C	160.6, C	156.9, C	160.5, C	159.9, C
3	105.1, CH	105.7, CH	105.1, CH	105.8, CH	98.9, C	99.4, C	101.7, C	101.1, C	98.9, C	99.4, C
4	162.6, C	163.1, C	162.3, C	163.1, C	159.5, C	161.5, C	160.6, C	160.8, C	159.6, C	161.4, C
5	115.6, CH	116.1, CH	115.7, CH	116.2, CH	114.9, CH	115.5, CH	117.6, C	112.2, C	115.2, CH	115.8, CH
6	149.7, C	150.7, C	149.5, C	150.4, C	147.9, C	149.0, C	144.4, C	147.2, C	148.6, C	149.6, C
7	163.2, C	164.3, C	163.4, C	164.3, C	162.7, C	163.1, C	163.5, C	164.1, C	161.9, C	162.4, C
8	35.5, CH_2	36.4, CH_2	35.3, CH_2	36.3, CH_2	36.6, CH_2	36.5, CH_2	37.2, CH_2	37.4, CH_2	35.8, CH_2	36.6, CH_2
9	24.7, CH_2	25.4, CH_2	24.7, CH_2	25.4, CH_2	24.6, CH_2	25.3, CH_2	22.9, CH_2	24.1, CH_2	24.6, CH_2	25.3, CH_2
10	14.3, CH_3	14.3, CH_3	14.2, CH_3	14.3, CH_3	14.3, CH_3	14.4, CH_3	14.6, CH_3	14.2, CH_3	14.4, CH_3	14.3, CH_3
1′	141.6, C	143.1, C	142.4, C	143.8, C	141.9, C	143.3, C	142.3, C	143.5, C	143.0, C	144.1, C
2′	143.7, C	145.1, C	142.9, C	144.1, C	144.5, C	145.6, C	145.7, C	145.7, C	143.9, C	144.9, C
3′	105.5, CH	106.2, CH	99.2, C	99.8, C	105.5, CH	106.0, CH	105.2, CH	106.0, CH	103.9, CH	103.4, CH
4′	152.4, C	152.8, C	152.6, C	153.0, C	155.4, C	155.9, C	154.9, C	156.6, C	154.3, C	155.3, C
5′	108.3, C	108.7, C	112.7, CH	113.3, CH	113.8, CH	114.2, CH	113.1, C	114.7, C	110.2, C	110.7, C
6′	135.7, C	136.7, C	134.3, C	135.4, C	136.3, C	137.4, C	135.9, C	137.7, C	136.1, C	137.4, C
7′	32.4, CH_2	33.1, CH_2	31.2, CH_2	32.2, CH_2	32.8, CH_2	33.8, CH_2	32.9, CH_2	34.2, CH_2	32.9, CH_2	33.8, CH_2
8′	22.7, CH_2	23.4, CH_2	23.6, CH_2	24.2, CH_2	24.4, CH_2	25.0, CH_2	24.6, CH_2	25.4, CH_2	23.1, CH_2	23.7, CH_2
9′	14.5, CH_3	14.4, CH_3	14.3, CH_3	14.2, CH_3	14.2, CH_3	14.3, CH_3	14.3, CH_3	14.2, CH_3	14.4, CH_3	14.2, CH_3
OMe	-	-	-	-	-	-	-	-	57.4, CH_3	57.3, CH_3

[a]500 MHz; [b]400 MHz; [c]600 MHz; [d]Recorded in DMSO-d_6; [e]Recorded in acetone-d_6; [f]Recorded in methonal-d_4. P: spiromastixone P, Q: spiromastixone Q, R: spiromastixone R, S: spiromastixone S, T: spiromastixone T. The NMR data of known analogues are cited from the literature (Guo, Z.; Zhu, W.; Zhao, L.; Chen, Y.; Li, S.; Cheng, P.; Ge, H.; Tan, R.; Jiao, R. New antibacterial depsidones from an ant-derived fungus Spiromastix sp. MY-1, Chin. J. Nat. Med. 2022, 20, 627-632).
